# Comparison of the cytoplastic genomes by resequencing: insights into the genetic diversity and the phylogeny of the agriculturally important genus *Brassica*

**DOI:** 10.1186/s12864-020-06889-0

**Published:** 2020-07-13

**Authors:** Jiangwei Qiao, Xiaojun Zhang, Biyun Chen, Fei Huang, Kun Xu, Qian Huang, Yi Huang, Qiong Hu, Xiaoming Wu

**Affiliations:** 1 Key Laboratory of Biology and Genetic Improvement of Oil Crops, Ministry of Agriculture and Rural Affairs, Oil Crops Research Institute of the Chinese Academy of Agricultural Sciences, Wuhan, China; 2 Genosys Inc., Shenzhen, China

**Keywords:** *Brassica*, Rapeseed, Cytoplasmic DNA, Maternal origin, Evolutionary relationship, Cytoplasmic male sterility

## Abstract

**Background:**

The genus *Brassica* mainly comprises three diploid and three recently derived allotetraploid species, most of which are highly important vegetable, oil or ornamental crops cultivated worldwide. Despite being extensively studied, the origination of *B. napus* and certain detailed interspecific relationships within *Brassica* genus remains undetermined and somewhere confused. In the current high-throughput sequencing era, a systemic comparative genomic study based on a large population is necessary and would be crucial to resolve these questions.

**Results:**

The chloroplast DNA and mitochondrial DNA were synchronously resequenced in a selected set of *Brassica* materials, which contain 72 accessions and maximally integrated the known *Brassica* species. The *Brassica* genomewide cpDNA and mtDNA variations have been identified. Detailed phylogenetic relationships inside and around *Brassica* genus have been delineated by the cpDNA- and mtDNA- variation derived phylogenies. Different from *B. juncea* and *B. carinata*, the natural *B. napus* contains three major cytoplasmic haplotypes: the *cam*-type which directly inherited from *B. rapa*, *polima*-type which is close to *cam*-type as a sister, and the mysterious but predominant *nap*-type. Certain sparse C-genome wild species might have primarily contributed the *nap*-type cytoplasm and the corresponding C subgenome to *B. napus*, implied by their con-clustering in both phylogenies. The strictly concurrent inheritance of mtDNA and cpDNA were dramatically disturbed in the *B. napus* cytoplasmic male sterile lines (e.g., *mori* and *nsa*). The genera *Raphanus*, Sinapis, *Eruca*, *Moricandia* show a strong parallel evolutional relationships with *Brassica*.

**Conclusions:**

The overall variation data and elaborated phylogenetic relationships provide further insights into genetic understanding of *Brassica*, which can substantially facilitate the development of novel *Brassica* germplasms.

## Background

The genus *Brassica* in *Brassicaceae* family is one of the most agriculturally important plant genera worldwide, which mainly comprises three diploid and three allotetraploid species, as described in the genetic model of U’s Triangle [1]. *Brassica napus* (AACC, 2*n* = 38), *B. juncea* (AABB, 2*n* = 36) and *B. carinata* (BBCC, 2*n* = 34) are thought to be generated by interspecific hybridizations between each two of the three basic diploid progenitors: *B. rapa* (AA, 2*n* = 20), *B. oleracea* (CC, 2*n* = 18) and *B. nigra* (BB, 2*n* = 16). The current abundant genomic and phenotypic diversifications have given rise to highly diverse crops of vegetable, oil, ornamental, fodder and fertilizer use types. To date, *B. napus* (rapeseed) has become to be the second largest vegetable oil crop worldwide [2]. Recently, the release of certain reference genome sequences has drived *Brassica* as an ideal model for studying polyploidy [3–7].

*B. napus* is supposed to originate from certain kind of hybridization between *B. rapa* and *B. oleracea*, which co-existed in European Mediterranean coastwise regions, at approximately 10,000 years ago [4]. Then it has diffused worldwide (mainly to Asia, America and Australia), and eventually formed several ecological and morphological types, which mainly include winter, spring and semi-winter ecotypes or oil-use, root-tuberous and leafy morphotypes. Recently, extensive resequencing and analyses on nuclear DNA concerning the mechanisms for the progenitors, evolution and improvement of this versatile crop have been performed. Phylogenomic analyses combining diverse *B. napus* and its potential progenitors revealed that winter type rapeseed might be the original form of *B. napus*, European turnip ancestor might donate the A subgemone, the origin of C subgenome is mysterious and it was currently supposed to evolve from a common ancestor of cultivated C-genome species (kohlrabi, cauliflower, broccoli, and Chinese kale) [8]. The A and C subgenomes evolved asymmetrically and higher genetic diversity was identified in A subgenome [9].

To date, the genuine originating mechanisms of *B. napus* remain largely unresolved. The frequent post-formation introgression events occurred during human breeding consequentially confused the recovery of the originating trajectory of *B. napus* at nuclear genome level. Cytoplasmic DNA in plant cell, especial for chloroplast DNA (cpDNA), are structurally simple with a small genome size (100–300 kb) and stably inherited mostly in a uniparental pattern with nearly none recombination [10]. Thus, it has been extensively employed in the phylogenetic studies [11–14]. Genotyping by using six chloroplast SSR primer pairs or TILLING analysis, one most prevalent cpDNA haplotype was identified in *B. napus* [15, 16]. While, the *B. napus* of this same cpDNA haplotype generally formed an ambiguous clade, which did not group with the investigated *B. rapa* or *B. oleracea* accessions [17], implying its mysterious origin. A few *B. napus* accessions were grouped with the majority of *B. rapa* accessions suggested another independent cytoplasmic origin from *B. rapa* [9, 18], indicating that has multiplex maternal origins. The mitochondrial DNA (mtDNA) of *B. napus* has drawn much more attention for the extensive application of its cytoplasmic male sterility (CMS) lines in the heterosis-driving hybrid breeding, mainly containing *polima* (*pol*), *cam* and *nap* mitotypes in the natural resources [17]*. Nap* mitotype is predominant in natural *B. napus*, However, it remains unsolved and were supposed to be from an unidentified or lost mitotype of *B. rapa* [19]. The *nap* mitotype was further judged to be derived from *B. oleracea*, since it was phylogenetically grouped with *botrytis*-type and *capitata*-type *B. oleracea* [20].

Apparently, the current above conclusions regarding the origin of *nap*-type *B. napus* are controversial and ambiguous. Previous cpDNA and mtDNA-based studies were separated and never been corresponded and integrated to accurately explore the multiply origin of *B. napus*. Cytoplasmic DNA and its corresponding cytonuclear interactions, are highly valuable for crop breeding not only due to its cause of cytoplasmic male sterility [21], but also in the association with certain agricultural traits, e.g., high seed-oil content in *nap*-type rapeseed [22] and plant resistance to adverse living environment. Here in this study, a well-chosen set of plant materials centering on *B. napus* have been synchronously resequenced at the cpDNA and mtDNA level, a systematic genetic investigation and an elaborate phylogenetic pedigree at intraspecific level have been constructed, with the purpose of improving our understanding of the whole *Brassica* genus.

## Results

### Sequencing of the diverse cytoplasmic *Brassica* DNA haplotypes

To distinguish the cytoplasmic DNA (cpDNA and mtDNA) haplotypes within *Brassica* genus, genotyping analysis through High Resolution Melting (HRM) method were performed in our germplasm collections (Figure S1). Primers were designed being targeted on a set of intra/inter-specific cpDNA polymorphic sites that were identified previously [16] (Table S1). Three major haplotypes were identified in approximately 480 worldwide *B. napus* accessions. Two major cpDNA haplotypes were identified in 180 *B. rapa* accessions, while 180 *B. juncea* accessions contain one major cpDNA haplotype. *B. oleracea*, *B. carinata*, *B. nigra*, *B. maurorum* (MM, 2*n* = 16), certain wild C-genome relatives and three *B. napus* cytoplasmic male sterility (CMS) lines were treated as each with a distinct haplotype for the subsequent genome sequencing. *B. cretica*, *B. incana*, *B. insularis* and *B. villosa* represent the wild C-genome relatives. *Polima* [23, 24], *nsa* [25] and *mori* [26, 27] are the CMS lines. Certain relative materials, i.e. *Eruca sativa* (2*n* = 22), *Raphanus sativus* (2*n* = 18), *Sinapis arvensis* (2*n* = 24) and *Moricandia arvensis* (2*n* = 28), were also included to enrich this study (Table S2).

Cytoplasmic DNA was synchronously isolated from 72 accessions that represent for all major cytoplasmic haplotypes and morphological varieties (Table S2), using an optimized organelle isolation procedure (Materials and Methods). This method can substantially help to remove nuclei and balance the proportions of cpDNA and mtDNA content. Reads mapping analysis demonstrated that the isolated total DNA contains an average ratio of 37.2% chloroplast DNA and 3.4% mitochondrial DNA, respectively, which is approximately 5–10 times higher than the ratio of cytoplasmic DNA in the total leaf DNA [28]. The cytoplasmic DNA mixture was then subjected to the high-throughput sequencing (with average sequencing depths above 500 x, Table 1). The obtained paired-end reads (150 bp) were directly mapped to a tandem sequence gather, which consists of 10 published chloroplast genome sequences across *Brassicaceae* family. The mapped reads were extracted and de novo assembled by SOAPdenovo software package [29]. Generally, two or three large contigs were eventually generated for the chloroplast genomes. Gaps were directly filled through manual jointing of the overlapping ends of each two contiguous contigs, and then verified by Sanger sequencing of the gap-spanning PCR fragments. All the obtained chloroplast genome sequences are provided in Additional file 3 (Appendix A).

**Table 1:** Sequencing information of the representative materials

Species (names)	Entry Number	Discriptions	Total Data (G)	Data of chloroplast genomes			Data of mitochondrial genomes		
				Data (G)	Rations	Average depth	Data (G)	Rations	Average depth
*B. rapa* ssp. *oleifera*	A22	oilseed use	3.20	1.74	54.44%	11,387	0.22	6.95%	1002
*B. rapa* ssp. *oleifera*	A173	oilseed use	3.34	1.91	57.11%	12,467	0.17	5.11%	769
*B. juncea*	AB81	oilseed use	5.69	1.21	21.18%	7877	0.12	2.06%	529
*B. juncea* var*. tumida*	AB180	vegetable use (Zha-cai)	3.47	0.82	23.78%	5386	0.06	1.60%	250
*B. napus*	AC32	*Cam*-type cytoplasm	6.90	1.90	27.54%	12,418	0.22	3.21%	998
*B. napus*	AC399	*Polima*-type cytoplasm	4.53	2.65	58.59%	17,347	0.12	2.66%	542
*B. napus* (Zhongshuang11)	AC457	*Nap*-type cytoplasm	9.37	3.90	41.60%	25,480	0.96	10.21%	4311
*B. napus* (Darmor)	AC489	*Nap*-type cytoplasm	8.14	3.31	40.70%	21,647	0.59	7.23%	2649
*B. napus* (*Mori* sterile line)	AC490	Recombinant cytoplasm	5.37	2.24	41.70%	14,637	0.41	7.58%	1834
*B. napus* (*Nsa* sterile line)	AC497	Recombinant cytoplasm	5.87	0.91	15.51%	5948	0.06	0.94%	250
*Brassica insularis*	C1	wild species	7.23	3.08	42.59%	20,111	0.21	2.89%	943
*Brassica oleracea var. oleracea*	C3	wild species	4.19	1.79	42.76%	11,710	0.14	3.24%	612
*Brassica cretica*	C5	wild species	4.36	1.67	38.41%	10,947	0.18	4.20%	825
*Brassica villosa*	C11	wild species	8.73	2.00	22.94%	13,090	0.19	2.15%	847
*Brassica oleracea var. italica*	C16	cultivar (Broccoli)	3.35	1.29	38.43%	8402	0.08	2.33%	352
*Brassica nigra*	B2	wild species	6.29	0.54	8.66%	3561	0.05	0.72%	204
*B.maurorum*	Maurorum-1	wild species	2.63	0.97	36.73%	6314	0.05	2.01%	238
*Brassica carinata*	BC2	cultivar	3.94	1.72	43.70%	11,254	0.23	5.76%	1022
*Sinapis arvensis*	Sinapis1	wild species	6.67	2.24	33.60%	14,649	0.32	4.76%	1431
*Sinapis arvensis*	Sinapis3	wild species	7.76	2.43	31.28%	15,866	0.12	1.61%	563
*Raphanus sativus*	Raphanus-1	cultivar	7.55	2.44	32.32%	15,951	0.34	4.49%	1527
*Moricandia arvensis*	Moricandia-1	wild species	7.23	2.95	40.83%	19,295	0.22	3.05%	992
*Eruca sativa*	Eruca-1	cultivar	6.55	1.78	27.14%	11,619	0.30	4.63%	1366

### Genome-wide cytoplasmic (cpDNA and mtDNA) variations in *Brassica*

**Fig. 1:**
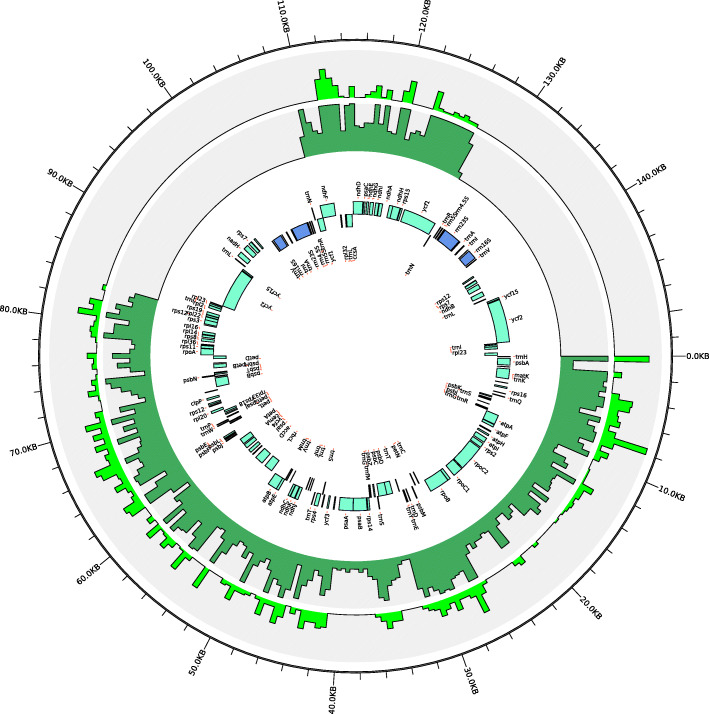
Genomic distribution of the basic cpDNA variants in the sequenced materials. The map was drawn using Circos (http://circos.ca/). The innermost circle represents for the chloroplast genome map of *B. napus* strain 51,218. The inner bottle-green bars and outer laurel-green bars correspond to the distribution of SNPs and InDels within nonoverlapping 500-bp bins across the entire genome, respectively. The length of each bar denotes the total number of basic variants in a 500-bp region, take the value as 30 if it exceeds 30. None variants appeared in two inverted repeat regions, IRa (83–109 kb) and IRb (126–153 kb)

**Fig. 2:**
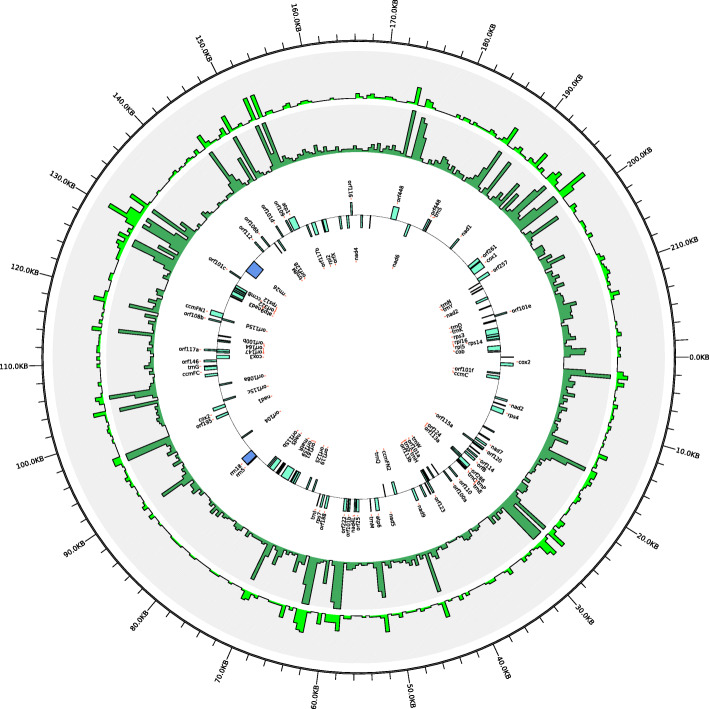
Genomic distribution of the basic mtDNA variants in the sequenced materials. The map was drawn using the same procedure as for Fig. 1. The innermost circle represents for the mitichondrial genome map of *B. napus* strain 51,218. The inner bottle-green bars and outer laurel-green bars correspond to the distribution of SNPs and InDels, respectively

The chloroplast and mitochondrial genome sequences of a *B. napus* strain 51,218 [22], which is an intermediate breeding material of *nap* mitotype, were respectively used as reference sequences to call the overall cpDNA and mtDNA basic variants. The calling was conducted by standard BWA/Genome Analysis Toolkit (GATK) pipeline with manual inspection [30], and then randomly verified by Kompetitive Allele Specific PCR (KASP) analysis. A total of approximately 4700 reliable basic polymorphic sites, including 3880 SNP and 820 InDels, respectively, were identified for all the sequenced chloroplast haplotypes in *Brassica* genus. While, approximately 3400 polymorphic sites (2700 SNP and 700 InDels) were identified for the mitochondrial haplotypes (Table S3). The average SNP density in the chloroplast and mitochondrial genomes was 25 and 12 SNPs per kilo base (kb), respectively. The chloroplast variants were uniformly distributed along the reference genome, except the two 26-kb large inverted repeat regions, IRa and IRb (Fig. 1), since these genomic regions were skipped due to the repetitive mapping of the same reads. The mitochondrial variants showed a comparatively even distribution pattern along the reference genome; however, their variation frequencies are obviously much higher at the regions containing the open reading frame (ORF) genes (Fig. 2).

Among the overall variants, 13.9 and 18.1% were identified as nonsynonymous for 47 cpDNA coding genes and 61 mtDNA coding genes, respectively. The materials of two *B. napus* mitochondrial haplotypes, below known as *cam*- and *polima*-types, possess approximately 300 basic variants when referring to *B. napus* strain 51,218 mitochondrial genome of *nap*-type. *Polima*-type is close to *cam*-type with a difference of only about 50 conserved cpDNA variants (Table S3). Consistent difference patterns were also found for cpDNA variants as for the three cytoplasmic types. KASP analysis using the primers targeted to the *B. napus* mitotype-corresponding mtDNA and cpDNA polymorphic sites detected that *nap*, *cam* and *polima* cytoplasms accounted for 87.1, 7.2 and 5.7% in the investigated *B. napus* population (Figure S2). Undoubtedly, *nap*-type is the predominant cytoplasmic DNA haplotype, as identified in previous studies [15, 16]. Most of the *B. rapa* materials are of the same *cam*-type in *B. napus*, another major haplotype accounting for a frequency of approximately 5.8% in the investigated *B. rapa* population has been identified and named as *sarson*-type hereinafter, since it mainly exists in *B. rapa var*. *sarson* accessions.

### The phylogeny of *Brassica* genus conducted based on the whole chloroplast genomes

Analyses based on the whole chloroplast genomes or genome-wide variations instead of partial cpDNA fragments can infer a phylogeny with much higher resolution and reliability, even at lower taxonomic levels [14]. To forecast the evolutionary trajectories of *Brassica* crops, all the above-obtained whole chloroplast genomes were subjected to phylogenetic analysis. The phylogenetic trees tentatively conducted using the Maximum Likelihood method, neighbor-joining method and Bayesian method were almost identical. To reduce the calculating amount and avoid a corpulent tree, the trees comprising materials throughout each intra-species, *Brassica* genus and *Brassicaceae* family, respectively, were conducted stepwise by Maximum Likelihood method [31].

Chloroplast genome sequences of *Raphanus sativus*, *Isatis tinctoria*, *Matthiola incana* and *Arabidopsis thaliana* in *Brassicaceae* family (Data from NCBI, Additional file 3) served as outgroup to root the intra-specific trees. The results indicated that 13 *B. rapa* accessions, 14 *B. juncea* accessions, 24 *B. napus* accessions and 13 C-genome species each clustered well and were separately integrated into a species-specific group. The *B. rapa* separated a little branch containing only two accessions, which were classified as *sarson*-type cytoplasm mentioned above (Figure S3). The *B. juncea* accessions did not diverge any secondary branches, indicating a lack of cytoplasmic genetic diversity (Figure S4). The *B. napus* cluster were split into two large branches, one branch containing the *nap*-type lines (e.g., the nuclear-genome sequenced cultivars Darmor/AC489 and ZS11/AC457), another branch further split into two little branches, containing *cam*-type (e.g., Shengli Rape/AC32) and *polima*-type (e.g., Jianyang Rape/AC399) lines, respectively (Figure S5). All the investigated cultivated *B. oleracea* (e.g., Cauliflower, Broccoli, Cabbage, Kohlrabi) and part of the wild *B. oleracea* were shown with one nearly identical chloroplast genome sequence. However, the C-genome wild relatives (*B. villosa*, *B. insularis*, *B. cretica* and *B. incana*) each contains a distinct haplotype. All the C-genome species demonstrated a hierarchically clear pedigree, from *B. villosa* stepwise to the cultivated *B. oleracea* (Figure S6).

**Fig. 3:**
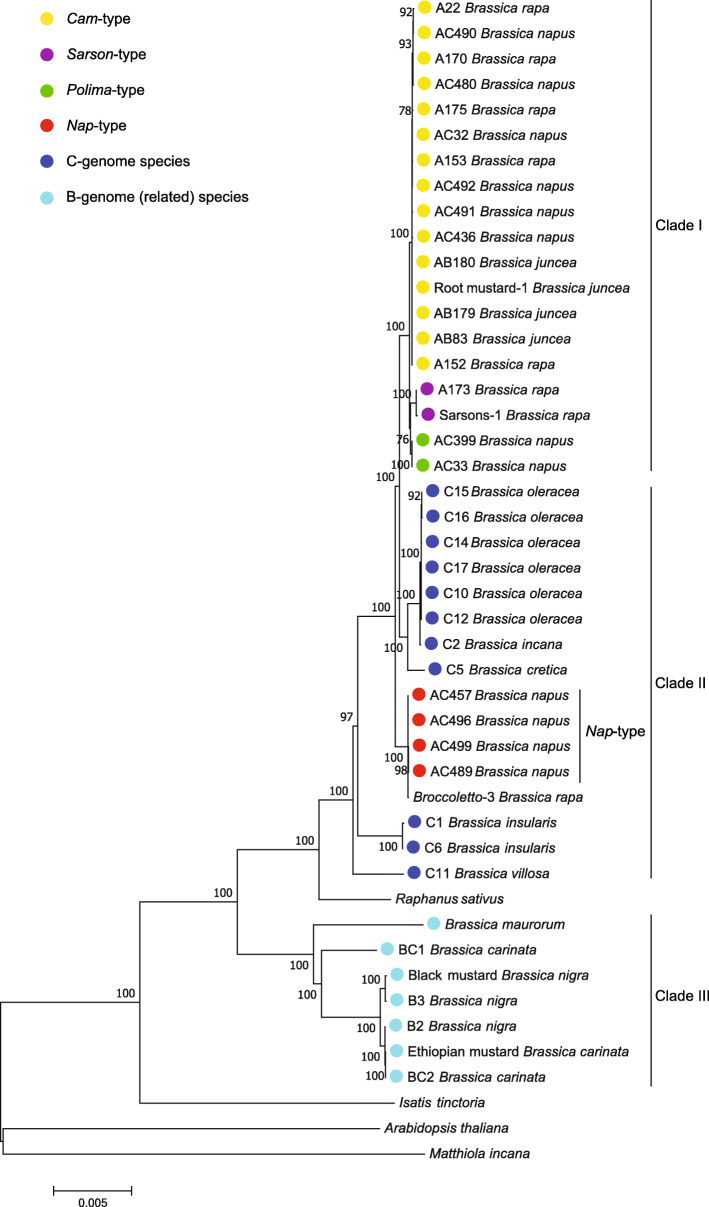
Molecular phylogeny of *Brassica* genus. This tree was inferred using Maximum Likelihood method based on 42 entire chloroplast genomes from representative materials centering on *Brassica* genus. The front letters A, AC, AB, C, BC and B of the entry name stand for the AA-, AACC-, AABB-, CC-, BBCC- and BB- genome species *B. rapa*, *B. napus*, *B. juncea*, *B. oleracea* (and other C-genome species), *B. carinata* and *B. nigra*, respectively. The numbers displayed in the corresponding branching nodes are the bootstrap values (%) calculated from 500 trials, supporting the reliability of the obtained tree structure. The length of branches indicates the evolutionary divergence according to the scale bar (relative units) at the bottom. The input materials with diverse cytoplasmic haplotypes were labeled with cycles of corresponding colors, the separated clades constitute the whole evolutionary pedigree are marked on the right

A part of the above intra-specific materials were selected capable of maximumly representing each their intraspecific genetic diversities, and then together with *Brassica nigra*, *B. carinata* and *B. maurorum*, were combined to construct a larger tree comprising of materials all over *Brassica* genus. The cpDNA sequence data for materials Root mustard-1 (*B. juncea*), Sarsons-1 (*B. rapa*), Broccoletto-3 (*B. rapa*), Black mustard (*B. juncea*) and Ethiopian mustard (*B. carinata*) were added from Li et al., [18] to enrich the whole phylogenetic tree. The results indicated that *Brassica* genus was mainly divided into three clades, from which the maternal origin of the three natural allotetraploid species can be clearly inferred (Fig. 3). All the *B. rapa*, *B. juncea* and quite a few *B. napus* accessions of both *cam-* and *polima*-type constitute Clade I, which further diverged two little branches containing *B. rapa* ssp. *trilocularis* (Sarsons) and *polima*-type *B. napus*, respectively. Three *B. juncea* accessions clustered only in Clade I without any further divergences from their co-clustered *B. rapa* accessions, thus indicating that the investigated *B. juncea* has a monophyletic maternal origin from *cam*-type *B. rapa*. Clade II comprises all the *B. oleracea* lines and other wild C-genome species, parallelly branched with Clade I. The branch, which comprises only the *B. napus* accessions with a same *nap* cytoplasmic type, is inserted in the middle of Clade II and separated certain C-genome wild relatives (*B. insularis* and *B. villosa*) from the remaining part, which contains all *B. cretica*, *B. incana* and the cultivated *B. oleracea*. Clade III comprises mainly *B. nigra*, *B. carinata* and *B. maurorum* accessions, indicating that the investigated *B. carinata* has a monophyletic maternal origin from *B. nigra*. The major cytoplasmic haplotype of *B. nigra* was designated as *nigra*-type cytoplasm. The wild species *B. maurorum* had been reported to be close to the B-genome species [32] and seems evolved earlier than all the remaining part in Clade III. The topological branches in this tree displayed a clear hierarchical pedigree, from Clade III to Clade I (Fig. 3). Taken together, different from *B. juncea* and *B. caritana*, *B. napus* was dispersedly distributed in the *B. rapa* and *B. oleracea* clusters, suggesting its multiple maternal origins from A-genome *B. rapa* or certain C-genome *Brassica* species (2*n* = 18).

### The evolution of *Brassica* tightly associates with a set of its close genera

**Fig. 4:**
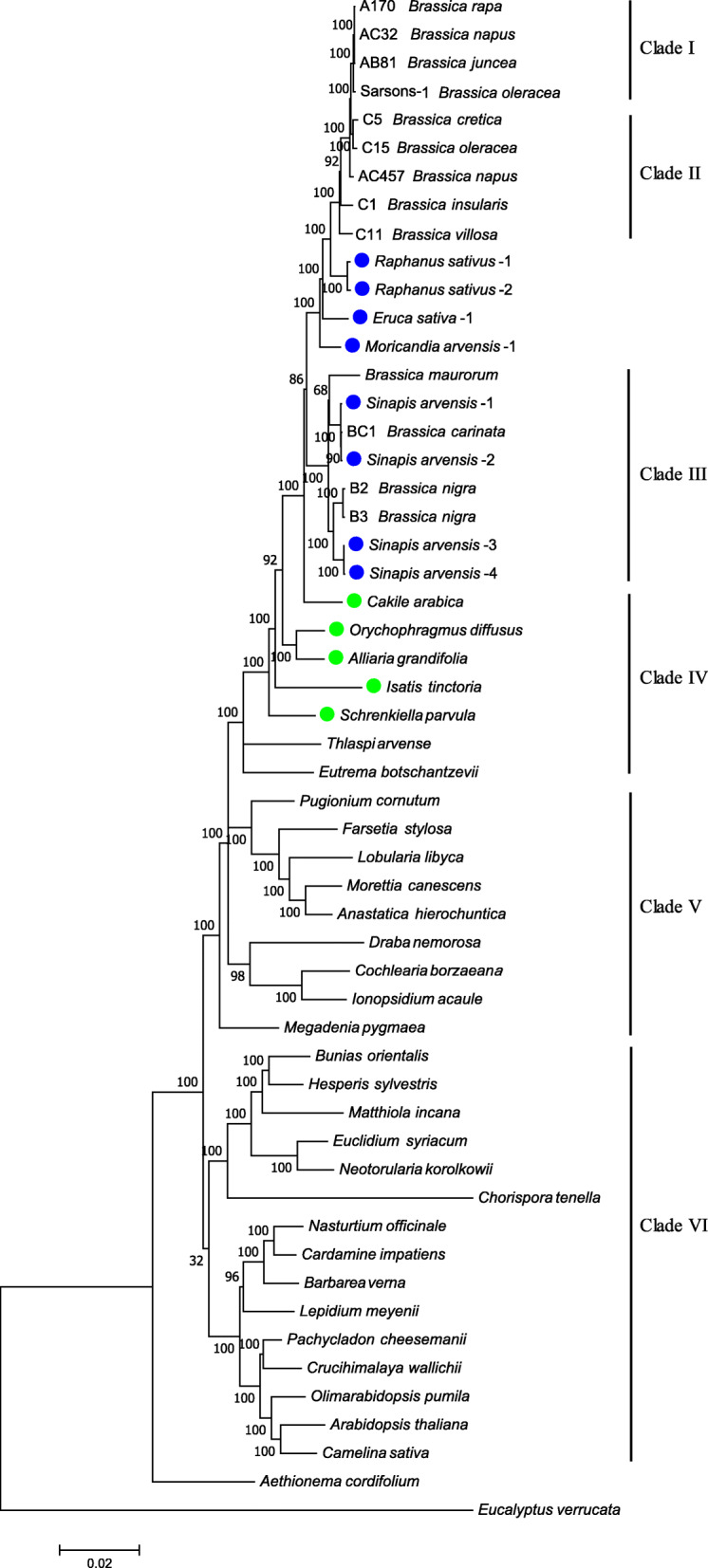
Molecular phylogeny of *Brassicaceae* family. This tree was inferred using Maximum Likelihood method based on the entire chloroplast genomes from representative materials based on 54 chloroplast genomes. This tree was conducted and handled the same as it in Fig. 3. Sequence information for the chloroplast genomes of other cruciferous species are provided in Materials and Methods. Accessions representing the genera integrated into the phylogeny of *Brassica* genus are labeled with blue cycles, Accessions representing the genera close to *Brassica* genus are labeled with green cycles

Intriguingly, *Raphanus sativus* was inserted between Clade II and Clade III and bidirectionally close to *B. villosa* and *B. maurorum* in the *Brassica* phylogenetic tree (Fig. 3), suggesting certain association between *Raphanus* genus and *Brassica* phylogeny. To explore whether any more other genus also mingle with *Brassica* genus, a phylogenetic tree containing 54 (Thirteen in and 41 beyond *Brassica* genus) chloroplast genome sequences in *Brassicaceae* family was constructed (Fig. 4). The tree displays an evolutionary pedigree with a clear hierarchical architecture. The *Brassicaceae* family was basically divided into two large lineages, containing *Arabidopsis*/*Matthiola* and *Draba*/*Brassica* genera, respectively, which is congruent with the previous studies [33, 34]. Another three materials, *Eruca sativa*, *Moricandia arvensis* and *Sinapis arvensis*, were also identified to be tightly integrated with the evolution of *Brassica* genus. *Eruca sativa* and *Moricandia arvensis* were located at the same positions as *Raphanus sativus*, while three herein sequenced and one public *Sinapis arvensis* (*Sinapis*-4) accessions displayed scattered distribution that is fully merged together with the B-genome containing species in Clade III. These findings imply a tight evolutionary association among *Brassica* and these relatives. *Cakile arabica*, *Orychophragmus diffusus*, *Alliaria grandifolia*, *Isatis tinctona* and *Scherenkiella parvula* in Clade IV were shown to be close to *Brassica* cluster at cytoplasmic DNA level. Successful germplasm development through inter-specific sexual or somatic hybridization between *Brassica* species with *Orychophragmus violaceus* or *Isatis tinctona* [35, 36] could partially support that the species in Clade IV are fairly close to *Brassica*.

### Uncoupled inheritance of chloroplast and mitochondrial genomes in *B. napus* CMS lines

**Fig. 5:**
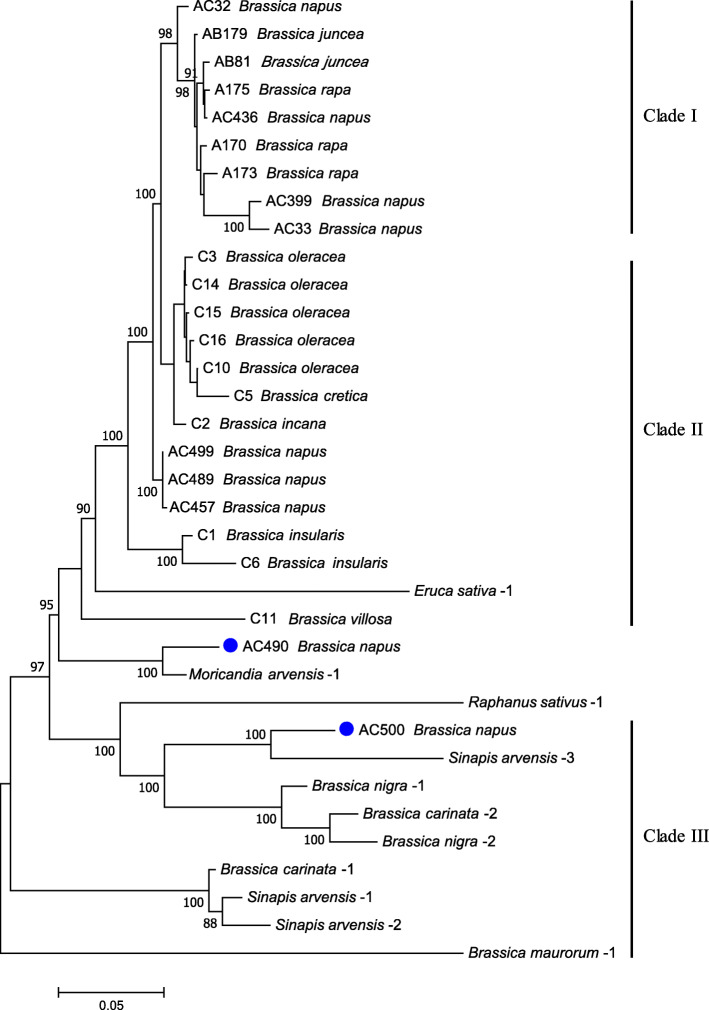
Molecular phylogeny of *Brassica* genus inferred using Maximum Likelihood method based on mitochondrial genome variations. The *B. napus mori* and *nsa* CMS lines were labeled with blue cycles

Mitochondrial genome represents another half set of cytoplasmic DNA. To ascertain how about the *Brassica* phylogeny if being inferred based on mitochondrial genomes, the segmented sequences containing the mitochondrial allelic variants from each corresponding material inside and around *Brassica* genus were extracted and concatenated as each separate intact sequence. All the assembled sequences were subjected to phylogenetic analysis according to the above same procedure used for chloroplast genomes. The obtained mitochondrial tree (Fig. 5) displayed a pedigree largely resembling the tree that was derived based on cpDNA (Fig. 3). Likewise, it also diverged into three clades, each of the natural *Brassica* materials possesses nearly identical evolutionary positions in both the cpDNA and mtDNA deriving trees, the same maternal origin relationships of the three *Brassica* allotetraploid crops were inferred. The location of four genera (*Raphanus sativus*, *Eruca stivus*, *Moricandia arvensis* and *Sinapis arvensis*) in the mtDNA derived tree were also integrated into *Brassica* genus, demonstrating that mtDNA evolved parallelly linked with cpDNA in *Brassica* genus. Nevertheless, differences happened for the *B. napus* cytoplasmic male sterile lines, i.e., *mori* [26, 27] and *nsa* [25] CMS lines, which have been successfully utilized in heterosis-driving hybrid breeding. *Mori* and *nsa* lines located in the *cam*-type and *nap*-type *B. napus* clusters, respectively, in the cpDNA deriving tree (Figure S5), and possess the identical natural *cam*-type and *nap*-type chloroplast sequences, respectively. However, they are clustered close to their mtDNA donor species in the mtDNA deriving tree (Fig. 5), i.e., the *B. napus mori* and *nsa* sterile line each clustered together with *Moricandia arvensis* and *Sinapis arvensis*, respectively. The coupled inheritance of mitochondrial genomes and chloroplast genomes in the *B. napus* CMS lines has been disturbed.

### Estimation of divergence times of *Brassica* crops

The phylogenetic tree containing 54 chloroplast genome sequences in *Brassicaceae* family (Fig. 4) was subjected to estimate the divergence time for these investigated *Brassica* species, the timetree was conducted by Reltime [37]. It was calibrated referring to two previously estimated divergence times: 30–35 million years ago (Mya) which dated the speciation of genus *Aethionema* and 25–30 Mya which dated the separation of two large *Brassicaceae* clades including *Arabidopsis* and *B. napus*, respectively [33, 38]. *Eucalyptus verrucata* was set as the outgroup. The obtained timetree (Figure S7) indicated that *Aethionema* might be an ancient cruciferous genus and there were two major periods for species radiation in *Brassicaceae* family. During 25–18 Mya, certain genus emerged and separated from each other; and then during the second radiation period (15–6 Mya), most of the genus speciated and formed several large clades. *Brassica* genus emerged approximately at 4.85 Mya, and began maybe as a kind of *B. nigra* or *B. rapa*. *Moricandia arvensis*, *Eruca stivus*, *Brassica maurorum*, *Raphanus sativus* and *Sinapis arvensis* speciated at 3.15 Mya, 2.85 Mya, 2.17 Mya, 2.05 Mya and 1.42 Mya, respectively. The *Brassica* C-genome species (e.g., *B. villosa* and *B. oleracea*) separated from A-genome species (*B. rapa*) since 1.12 Mya. Three allotetraploid species (*B. juncea*, *B. carinata* and *B. napus*) speciated during the period 0.17–0.01 Mya or much later, which are consistent with the estimated originating time of ~ 7500 years ago for *B. napus* [4] and the cultivation beginning time of ~ 7000 years ago for *B. juncea* [7]. The *Brassica* tetraploid species are much younger than certain other polyploidy crops, e.g., the emerge of cotton (*Gossypium hirsutum*) at 1–2 Mya [39, 40] and the emerge of soybean (*Glycine max*) at 0.8 Mya [41].

## Discussion

### The comparative genomics of cytoplasmic genomes provides insights into the *Brassica* phylogeny and the origin of *nap*- type *B. napus*

As mentioned above, *B. rapa* mainly contains a predominant haplotype (*cam*-type) and a newly identified *sarson*-type cytoplasm, which presents merely approximately 50 cpDNA and 20 mtDNA basic variations (Table S3). Fourteen *B. juncea* aceesions including different vegetable and oil varieties possess only one chloroplast and nearly one mitochondrial DNA haplotype almost identical to the corresponding *B. rapa cam*-type haplotype. *B. carinata* (BC2) clustered next to *B. nigra*. None cytoplasmic DNA types of BB-genome and CC-genome diploid species have ever been detected in the germplasm collections of the natural *B. juncea* or *B. carinata*, respectively. These results ascertain that *B. juncea* and *B. carinata* each has a monophyletic maternal origin from *B. rapa* and *B. nigra*, respectively.

Three major haplotypes were identified in our natural *B. napus* collection. Of the 24 sequenced *B. napus* accessions, 7 lines tightly clustered with the majority of *B. rapa* in Clade I (Fig. 3) and thus are recognized as *cam*-type. They contain nearly none cpDNA SNP differences from their co-clustering *B. rapa* and *B. juncea* materials (Table S3), indicating a direct maternal origin of these *B. napus* accessions from the *cam*-type *B. rapa*. Two previously known *polima*-type lines (Xiang5A and 20A) also clustered in Clade I but adjacent to *sarson*-type *B. rapa* (Sarson-1) with minor divergence, suggesting that the *polima* haplotype may inherit from certain *sarson*-type like *B. rapa*, which is different from the previous assumption that *polima* haplotype likely diverge from *Cam* haplotype. *Nap*-type cytoplasm, which is predominant with a population frequency of 87.1% in our *B. napus* collection, resides in numerous elite rapeseed cultivars worldwide (e.g., Darmor and ZS11). The cluster of *nap*-type *B. napus* is inserted in the middle of C-genome Clade II, appears like a separate haplotype that is parallel to *B. cretica*, *B. incana*, *B. insularis*, *B. villosa* and *B. oleracea*. This was also supported by our mtDNA-based phylogeny (Fig. 5), and also by the recent cpDNA-based phylogenetic studies that included other three more C-genome species: *B. rupestris*, *B. montana* and *B. macrocarpa* [9].

**Fig. 6:**
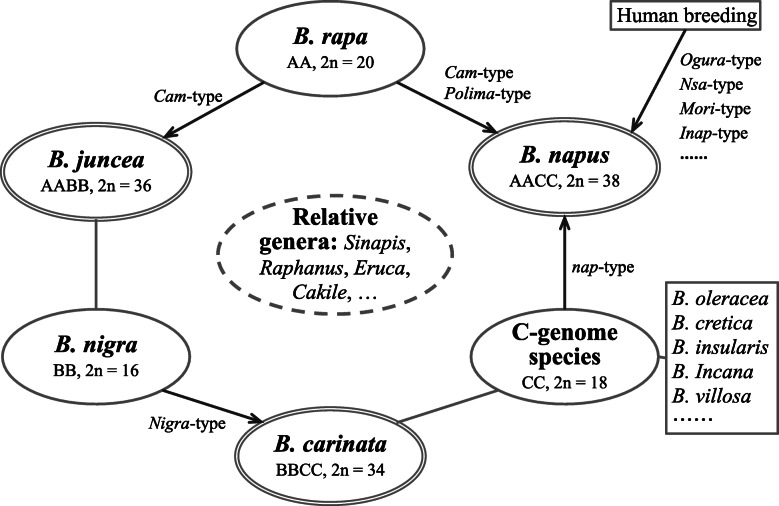
A refined model of U’s Triangle. Ellipses of single and double lines represent three basic diploid and three tetraploid species in *Brassica* genus. Diffusion of the corresponding cytoplasmic haplotypes were indicated by the arrows. Ellipse of dashed lines represents the close (diploid) species in other genera which can be used to create extensive germplasms with novel allotetraploid genomes and various cytonuclear combinations

Since *nap*-type cytoplasm is highly divergent from the known cultivated C- genome materials, there is a great possibility that one certain C-genome wild species rather than *B. oleracea* may have donated the *nap*-type cytoplasm to *B. napus*, and also the corresponding nuclear C subgenome. Judging from the cytoplasmic inheritance, the current natural *B. napus* may have three maternal parents, two of A genome *B. rapa* and one of C genome species, possessing higher cytonuclear diversity than *B. juncea* and *B. carinata*. A refined model of U’s Triangle delineating the diffusion of cytoplasmic haplotypes in *Brassica* genus has been proposed in Fig. 6. Unexpectedly, the *B. rapa* variety broccoletto had been previously identified possessing identical *nap*- cpDNA haplotype [15, 18]. Whether broccoletto is the original female parent of *nap*-type *B. napus* yet need further evaluation. The investigated broccoletto accession collected from Italy were generally cultivated alongside multifarious wild *Brassica* species [18]. Whereupon, the presence of *nap*-type haplotype in these *B. rapa* accessions may result from as yet unidentified introgression events, i.e., the stepwise transfer of *nap*-type cytoplasm from *B. napus* into *B. rapa* through natural hybridization and consecutive backcrosses.

### Strong parallel evolution among *Brassica* and several relative genera

As clearly demonstrated in both the cpDNA and mtDNA based phylogenetic trees (Fig. 4 and Fig. 5), *Raphanus sativus*, *Eruca sativa* and *Moricandia arvensis* located between *B. villosa* and *B. maurorum*, namely between the *B. oleracea* wild relatives and B-genome species. *Sinapis arvensis* converged with the B-genome containing species in Clade IV (Fig. 3). These results suggest a potential co-originating (and co-evolving) relationships among *Brassica* and these relative genera. Comparative analysis of genomic framework using 22 genomic blocks (GB) demonstrated that most GB associations in *Brassica* species could be detected in *Raphanus sativus* [42], suggesting that *Raphanus* and *Brassica* species potentially shared a common hexaploid ancestor after whole genome triplication (WGT). Common translocation Proto-Calepineae Karyotype (tPCK)-like ancestors were deduced to be the likely common ancestor of all current *Brassiceae* species that had undergone WGT and repetitive chromosomal rearrangements. Phylogenetic analysis based on 32 mitochondrial protein-coding genes suggested that *Eruca sativa* is closer to the *Brassica* species and *Raphanus sativus* than to *Arabidopsis thaliana* [43]. The U’s Triangle theory were accordingly revisited and extended into a multi-vertex model [42], which should include not only *Raphanus*, but also *Eruca*, *Moricandia* and *Sinapis* species as basic diploid species as suggested herein by our studies (Fig. 6). Future determination of nuclear genomes of the representative *Eruca*, *Moricandia* and *Sinapis* species would provide detailed insights into the genomic and evolutionary association among these genera.

### Heavy interspecific recombinations of mitochondrial genomes caused the coupled inheritance of both cytoplasmic genomes

Generally, mitochondrial and chloroplast genomes demonstrate consistent evolutionary relationships in higher plants, because of their coupled inheritance in a uniparental manner. The inconsistent locations of *B. napus mori* and *nsa* sterile lines, in the mtDNA and cpDNA based phylogenetic tree (Fig. 5 and S5) revealed their uncoupled inheritance of mitochondrial and chloroplast genomes. *Mori* sterile line (AC490) was primarily obtained by protoplast fusion between *Moricandia arvensis* (MM, 2*n* = 28) and *B. juncea* [26, 44], and then the CMS phenotype was transferred into *B. napus* through several rounds of sexual hybridization. *Nsa* sterile line (AC500) was developed primarily also from protoplast fusion between *B. napus* and *Sinapis arvensis* [25]. Sequencing analysis of *Ogura* sterile line, which was also developed through somatic hybridization of *Raphanus sativus* and *B. napus* [45, 46], revealed that rearrangement happened extensively in its mitochondrial genome [47]. Nine regions were identified to be unique to the all the published *Brassica* mitochondrial genome sequences belonging to U’s Triangle. Therefore, both the *mori* and *nsa* lines ought to contain plenty of mitochondrial genome regions from their incipient donor parents, thus clustered close to *Moricandia arvensis* and *Sinapis arvensis*, respectively, in the mtDNA based phylogenetic tree. It seems that somatic hybridization through protoplast fusion is an effective means to induce the recombination of mitochondrial genomes. Intergenomic recombinations and DNA rearrangements had been frequently identified within mitochondrial genomes [48, 49], suggesting that there must be a stronger variating dynamics in mitochondrial genomes than in chloroplast genomes.

While, it is notable that none recombination happened with the chloroplast genomes, since both the *nsa* and *mori* lines possess the identical *nap*- and *cam*-type chloroplast genomes from each of their recipient *B. napus* and *B. juncea* lines (Figure S5), respectively. This may result from lower interspecific recombination frequencies for cpDNA or strong artificial selection during the breeding process. Similarly, recombination of parental mitochondrial genomes rather than chloroplast DNA has been identified in a cybrid (cytoplasmic hybrids) obtained by protoplast fusion of *Nicotiana tabacum* and *Hyoscyamus niger* [50]. Thus, this phenomenon also would be potentially existent in other *B. napus* cybrid materials, e.g., the recent *inap* [51] CMS lines containing mtDNA components from *Isatis indigotica*. Collectively, these results indicated that recent human breeding activities have drastically disturbed the evolutionary accordance between cpDNA and mtDNA in a mass of cybrid lines.

### Potential application of the *Brassica* cytoplasmic genetic resources

The diversified *Brassica* relatives stated above have been identified possessing desirable elite traits. *Eruca sativa* (2*n* = 22) is a diploid edible plant and its medicinal properties have various promoting effects to health [52]. *Moricandia arvensis* (2*n* = 28) is reported to be a C3-C4 intermediate species, transferring this feather of strong photosynthetic efficiency into *Brassica* crops have been tried by means of hybridization [53]. *Sinapis arvensisis* is a wild weedy plant of the genus *Sinapis*, both *Sinapis arvensisis* and *Sinapis alba* (2*n* = 24) possess high resistances to drought, leanness, multiple diseases, herbicides and pod shattering [54]. Certain *Raphanus* species were identified to be immune to clubroot [55]. The inter-specific evolutionary relationships (Fig. 6) of *Brassica* present a potential guidance for improving the current *Brassica* crops and even the development of certain novel allotetraploid gerplasms by intercrossing the corresponding diploid species.

Natural cytoplasmic variations could interact with nuclear genomes to shape a large proportion of phenotypic traits that contributed to adaptation [56]. The cytoplasmic genetic diversity in most of the current *Brassica* populations (e.g., *B. juncea*, *B. carinata* and cultivated *B. oleracea*) remain rather low, which may be a key limiting factor for crop improvment. To create extensive germplasms with various novel cytonuclear combinations may be of great significance for both the fundamental studies and the crop breeding in the future.

## Conclusions

Compared with the huge nuclear genomes, cytoplasmic DNA is a primary and easy means to evaluate the evolutionary relationships. Meanwhile, it is also highly effective to dissect the maternal origins and to infer the primary originating events. Herein, the overall genetic diversity of *Brassica* cytoplasmic genomes has been systematically identified by the synchronous resequencing of the chloroplast and mitochondrial genomes. The whole *Brassica* phylogeny has been refined and enriched, providing further insights into the understanding of the origin of the important *B. napus nap*-type cytoplasm. Human interference has remodeled the cytoplasmic inheritance in *B. napus*. The obtained genetic resources can substantially support the further research on the *Brassica* evolution, the development of novel germplasms.

## Methods

### Plant materials

A set of 480 worldwide *B. napus* accessions were collected from the National Mid-term Genebank for Oil Crops of China, it has been repeatedly used as a core rapeseed collection in our previous studies [57]. The *B. rapa* and *B. juncea* populations contain primarily landraces, which were collected across China. The cultivated *B. oleracea* inbred lines were obtained commercially from market, the wild *B. oleracea* and other C-genome wild species which are native to coastal southern and western Europe were collected from rocky Atlantic coasts of Spain (Bay of Biscay) and the Centre for Genetic Resources, The Netherlands (CGN). Detailed information in regard to all the above materials and other materials in *Brassica* genus and its relative genera are provided in Table S2. Plant materials were planted in the experimental fields or greenhouses of Oil Crops Research Institute of CAAS in Wuhan (114.31°E, 30.52°N), from October 2015 to May 2017. The collection, identification, reproduction and conservation were conducted by the Rapeseed Germplasm Team in our institute, under the long-term support of Chinese national projects regarding species conservation and germplasm development. All the plant materials investigated here were deposited as seeds in the National Mid-term Genebank for Oil Crops of China.

### Genotyping analysis

Leaf total DNA of the corresponding accessions were directly extracted using the cetyltrimethylammonium bromide (CTAB) method described by Lutz et al., [58] and then subjected to genotyping analysis. High Resolution Melting (HRM) experiments were performed in 98/384-well plates using the Roche LightCycler 480® High Resolution Melting PCR Master Mix and analyzed by the LightCycler 480® Gene Scanning Software. Kompetitive Allele Specific PCR (KASP) analysis used for variant validation and haplotype dissection were performed using KASP Master mix according to the company’s protocols (LGC Genomics, Teddington, Middlesex, UK) on the Roche LightCycler 480® System.

### Isolation of the cytoplasmic DNA

Isolation of the cytoplasmic DNA was performed according to Hao et al., [59] with minor modifications. The newly developed young leaves were picked from 5 to 10 representative plants of each accession, and then homogenized thoroughly by Dounce homogenizer in isolation buffer [25 mM MOPS-KOH, 0.4 M mannitol, 1 mM EDTA, 10 mM tricine, 8 mM cysteine, 0.1% BSA (w/v) and 0.1% PVP-40 (w/v), pH 7.8]. One centrifugation step (300 g, 5 min) was performed to remove the unwanted whole plant cells and cell debris that mainly contain nuclear DNA contaminants. Another following centrifugation step (1500 g, 10 min) was added to remove a large proportion of chloroplasts to keep a proportionable ratio between cpDNA and mtDNA content. Then, the mixture of chloroplasts and mitochondria were collected by a further centrifugation step (20,000 g, 20 min) and then subjected to DNA isolation, using the CTAB method.

### Sequencing, genome assembly, variant calling and validation

High-throughput sequencing of the cytoplasmic DNA was performed according to our previous study [16]. The DNA was randomly ultrasonically sheared and prepared into paired-end (PE) libraries with insert sizes ranging from 300 to 400 bp, and then subjected to an Illumina Hiseq2500 (Illumina, San Diego, CA, USA) sequencing platform for sequencing at both single ends. Clean reads were directly mapped to a tandem sequence gather consisting of representative public cruciferous chloroplast genomes using Burrows-Wheeler Aligner (BWA) MEM program [60] under default parameters. The mapped paired-end reads were extracted and de novo assembled using the SOAPdenovo software package [29]. The obtained contigs were located on the yet published *Brassica* chloroplast genomes through BLAST alignments, and then sutured by manually jointing the overlapping ends between each two contiguous contigs. Basic variants (SNP and short InDels) were called using standard BWA/Genome Analysis Toolkit (GATK) pipeline [30], the chloroplast and mitochondrion genomes of *B. napus* line 51,218 (GenBank: KP161617.1 and KP161618.1) were used as the cpDNA and mtDNA reference genomes, respectively.

### Phylogenetic and molecular clock analysis

Chloroplast genome sequences were trimmed with aligned beginning sequences, and then subjected to alignment, which was conducted by ClustalW [61]. Maximum Likelihood trees were conducted by MEGA7 [62] based on Tamura-Nei substitution model. Timetree analysis was conducted using the RelTime method [37] based on original Newick formatted phylogenetic tree files, according to the guided procedure inplanted in MEGA7.

## Supplementary information

**Additional file 1 Figure S1.** Representative genotyping results by HRM analysis. (A) The normalized and temperature-shifted difference plot indicated that three site-specific haplotypes were identified in a plate of 96 plant DNA samples using HRM407 primers. (B) The normalized and temperature-shifted difference plot showed that two site-specific haplotypes were identified in a plate of 96 plant DNA samples using HRM727 primers. **Figure S2.** Representative genotyping results in a plate of 384 plant DNA samples by KASP analysis for primers mP1858 (A) and cP1225 (B). **Figure S3.** Phylogenetic tree of *Brassica rapa*. This tree structure was inferred using Maximum Likelihood method based on the entire chloroplast genomes from representative *B. rapa* materials. The sequence data for materials Zicaitai-1, Turnip-3 and Sarsons-1 from Li et al. (2017) were added. **Figure S4.** Phylogenetic tree of *Brassica juncea*. This tree structure was inferred using Maximum Likelihood method based on the entire chloroplast genomes from representative *B. juncea* materials. **Figure S5.** Phylogenetic tree of *Brassica napus*. This tree structure was inferred using Maximum Likelihood method based on the entire chloroplast genomes from representative *B. napus* materials. **Figure S6.** Phylogenetic tree of *Brassica* C-genome species. This tree structure was inferred using Maximum Likelihood method based on the entire chloroplast genomes from representative *B. oleracea* materials. **Figure S7.** Timetree analysis using the RelTime method. The timetree was computed based on the phylogenetic tree of *Brassicaceae* family in Fig. 4 using two calibration constraints labeled with blue stars and displayed only with topology. *Eucalyptus verrucata* labeled with lightgray was set as outgroup.

**Additional file 2 Table S1** Primers used for HRM and KASP genotyping analysis. **Table S2** List of plant materials investigated in this study. **Table S3** Total cpDNA and mtDNA variant data.

**Additional file 3 Appendix A** The dataset for chloroplast genome sequences of 72 *Brassica* accessions. **Appendix B** Accessions of the public sequence data.

## Data Availability

The chloroplast and mitochondrial genome sequences of *Brassica napus* strain 51218 can be found in GenBank under KP161617.1 and KP161618.1, respectively. Information for the cruciferous cpDNA sequence gather and chloroplast genome sequences used in phylogenetic analysis can be found in Additional file 3. The obtained chloroplast genomes were provided in Additional file 3 and deposited at Mendeley Data (DOI: 10.17632/skfwfrwgjs.1).

## Abbreviations

cpDNA: Chloroplast DNA
mtDNA: Mitochondrial DNA
*pol*: *Polima*
CMS: Cytoplasmic male sterility
KASP: Kompetitive allele specific PCR
