# Supplementary material for: Comparison of the cytoplastic genomes by resequencing: insights into the genetic diversity and the phylogeny of the agriculturally important genus Brassica
Source: BMC Genomics. 2020 Jul 13;21:480. doi: 10.1186/s12864-020-06889-0 (PMC7359470; doi:10.1186/s12864-020-06889-0)
Supplement: Supplementary file 1 — Additional file 1 Figure S1. Representative genotyping results by HRM analysis. (A) The normalized and temperature-shifted difference plot indicated that three site-specific haplotypes were identified in a plate of 96 plant DNA samples using HRM407 primers. (B) The normalized and temperature-shifted difference plot showed that two site-specific haplotypes were identified in a plate of 96 plant DNA samples using HRM727 primers. Figure S2. Representative genotyping results in a plate of 384 plant DNA samples by KASP analysis for primers mP1858 (A) and cP1225 (B). Figure S3. Phylogenetic tree of Brassica rapa. This tree structure was inferred using Maximum Likelihood method based on the entire chloroplast genomes from representative B. rapa materials. The sequence data for materials Zicaitai-1, Turnip-3 and Sarsons-1 from Li et al. (2017) were added. Figure S4. Phylogenetic tree of Brassica juncea. This tree structure was inferred using Maximum Likelihood method based on the entire chloroplast genomes from representative B. juncea materials. Figure S5. Phylogenetic tree of Brassica napus. This tree structure was inferred using Maximum Likelihood method based on the entire chloroplast genomes from representative B. napus materials. Figure S6. Phylogenetic tree of Brassica C-genome species. This tree structure was inferred using Maximum Likelihood method based on the entire chloroplast genomes from representative B. oleracea materials. Figure S7. Timetree analysis using the RelTime method. The timetree was computed based on the phylogenetic tree of Brassicaceae family in Fig. 4 using two calibration constraints labeled with blue stars and displayed only with topology. Eucalyptus verrucata labeled with lightgray was set as outgroup. [file 12864_2020_6889_MOESM1_ESM.docx]

**Supplementary Figures:**


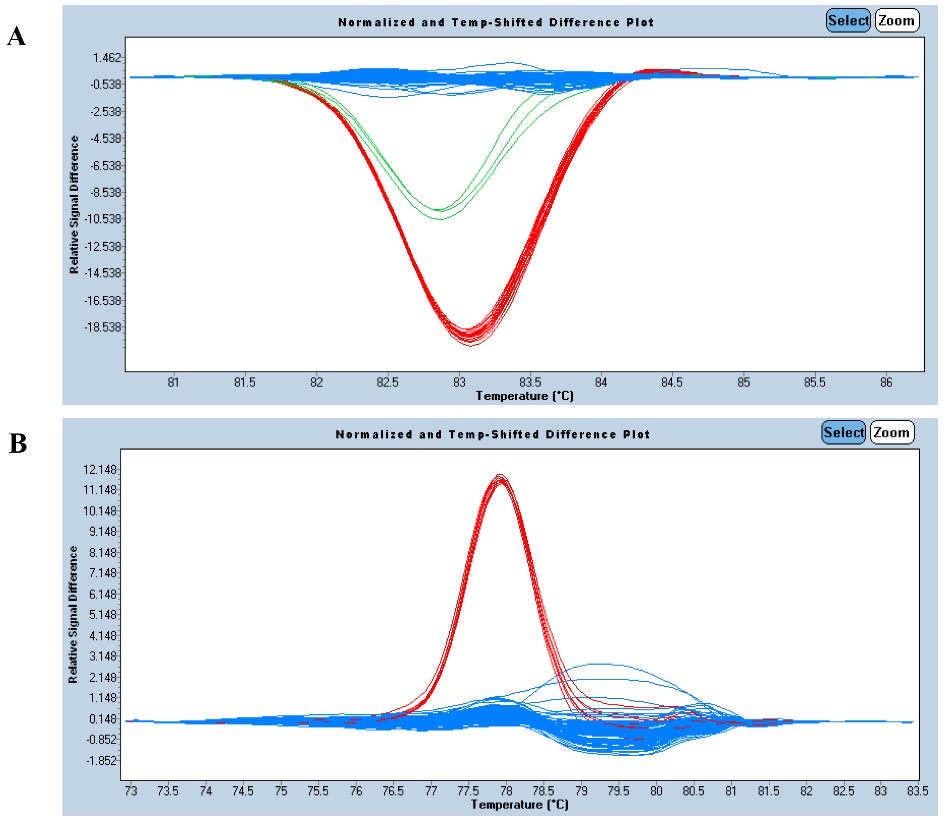


**Fig. S1.** Representative genotyping results by HRM analysis. (A) The normalized and temperature-shifted difference plot indicated that three site-specific haplotypes were identified in a plate of 96 plant DNA samples using HRM407 primers. (B) The normalized and temperature-shifted difference plot showed that two site-specific haplotypes were identified in a plate of 96 plant DNA samples using HRM727 primers.


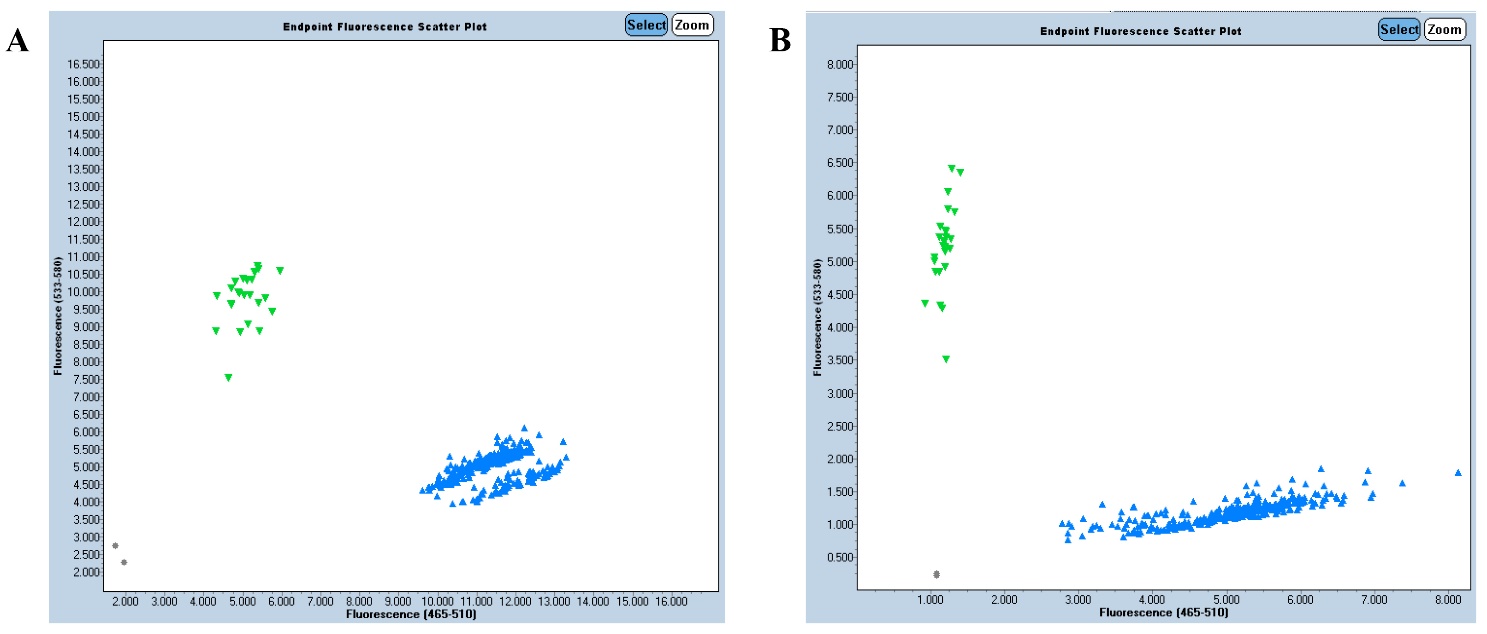


**Fig. S2.** Representative genotyping results in a plate of 384 plant DNA samples by KASP analysis for primers mP1858 (A) and cP1225 (B).


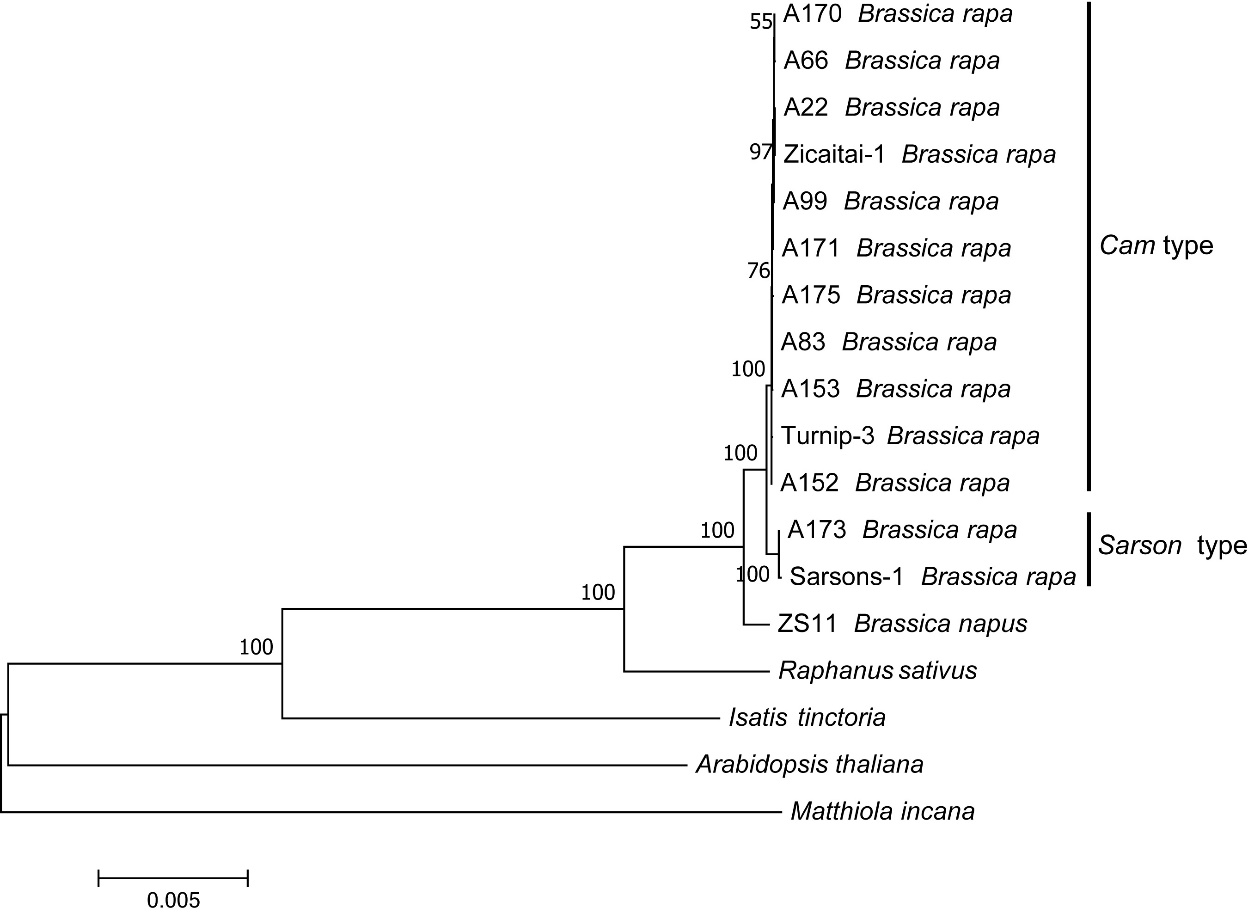


**Fig. S3.** Phylogenetic tree of *Brassica* *rapa*. This tree structure was inferred using Maximum Likelihood method based on the entire chloroplast genomes from representative *B. rapa* materials. The sequence data for materials Zicaitai-1, Turnip-3 and Sarsons-1 from Li et al. (2017) were added.


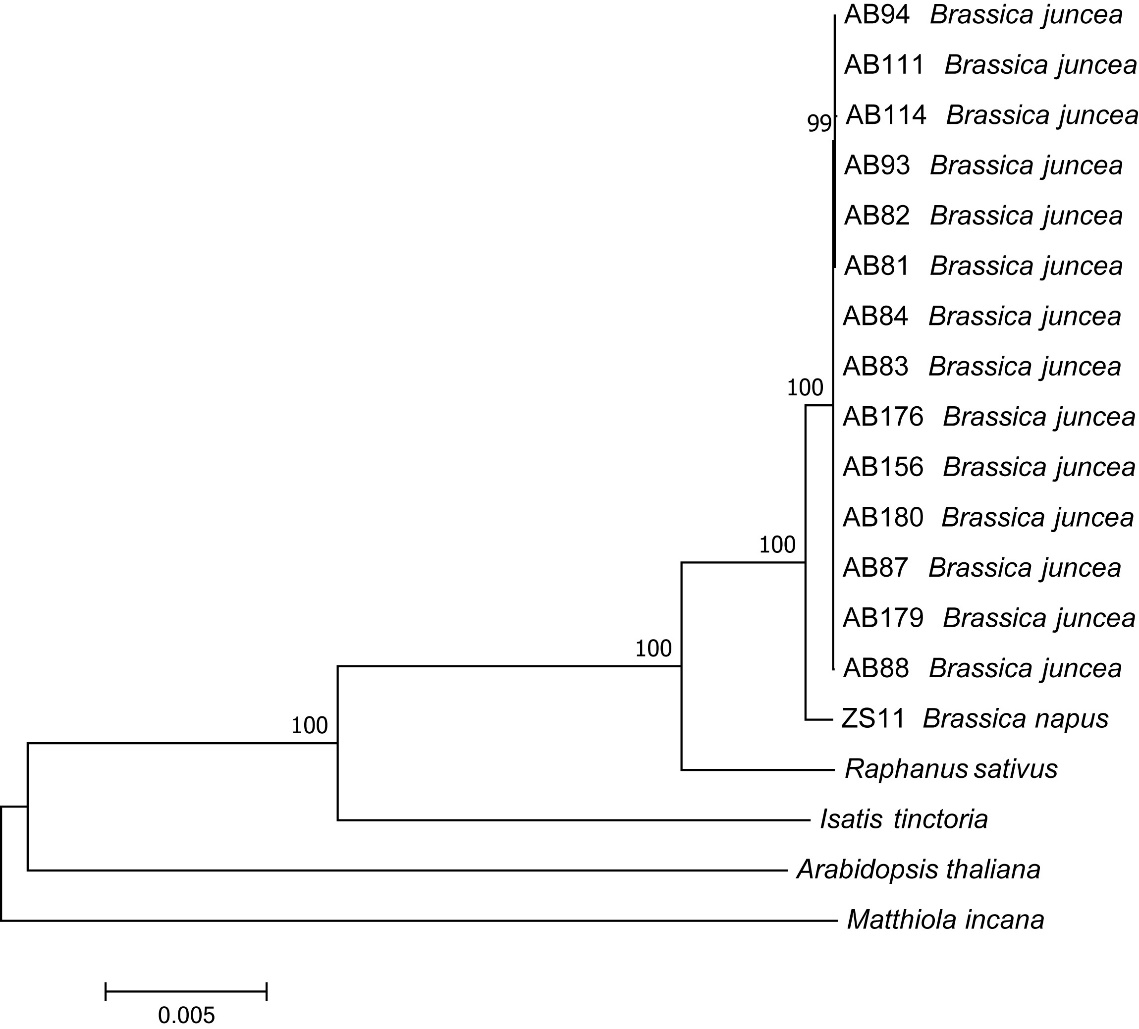


**Fig. S4.** Phylogenetic tree of *Brassica* *juncea*. This tree structure was inferred using Maximum Likelihood method based on the entire chloroplast genomes from representative *B. juncea* materials.


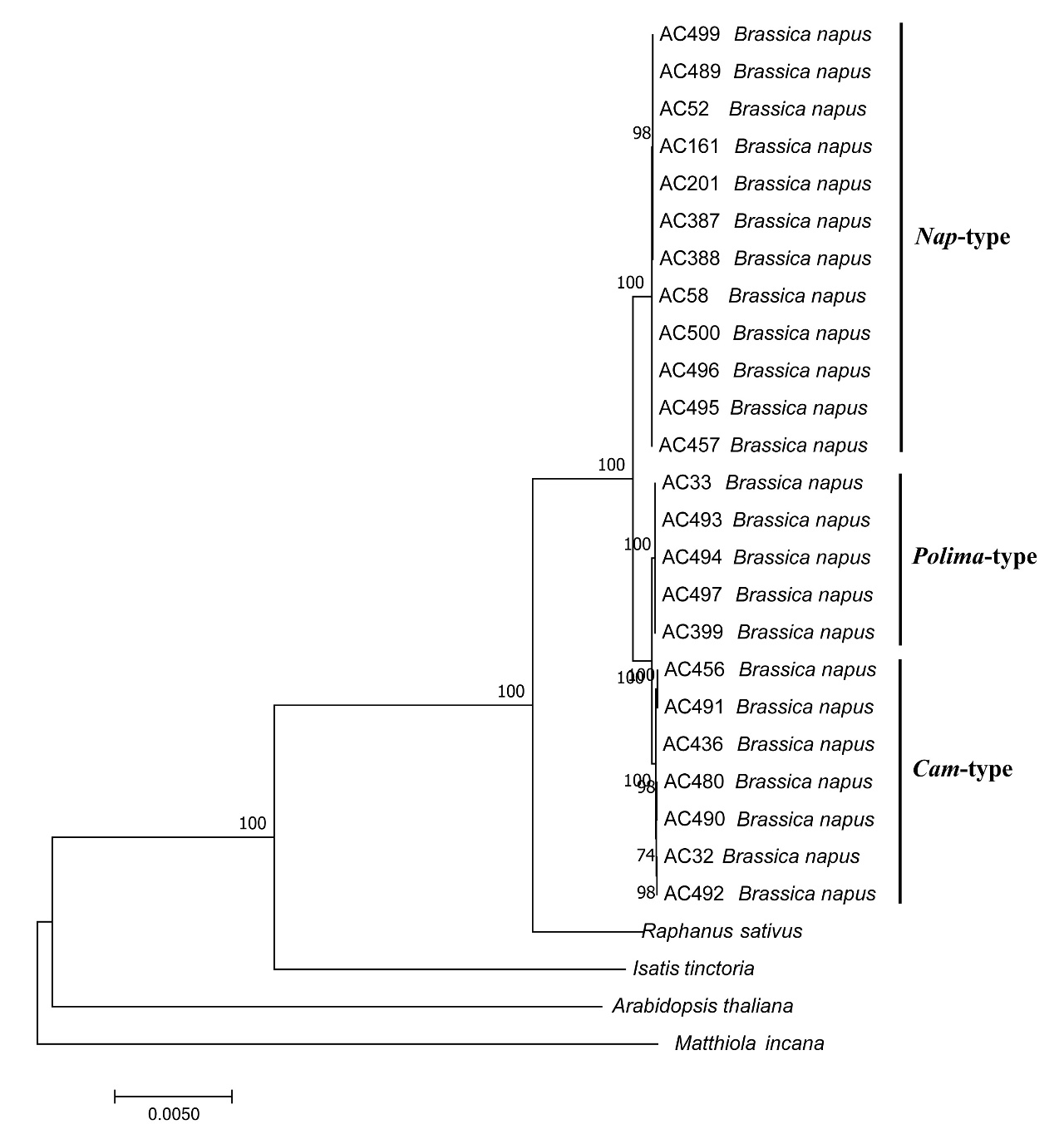


**Fig. S5.** Phylogenetic tree of *Brassica* *napus*. This tree structure was inferred using Maximum Likelihood method based on the entire chloroplast genomes from representative *B. napus* materials.


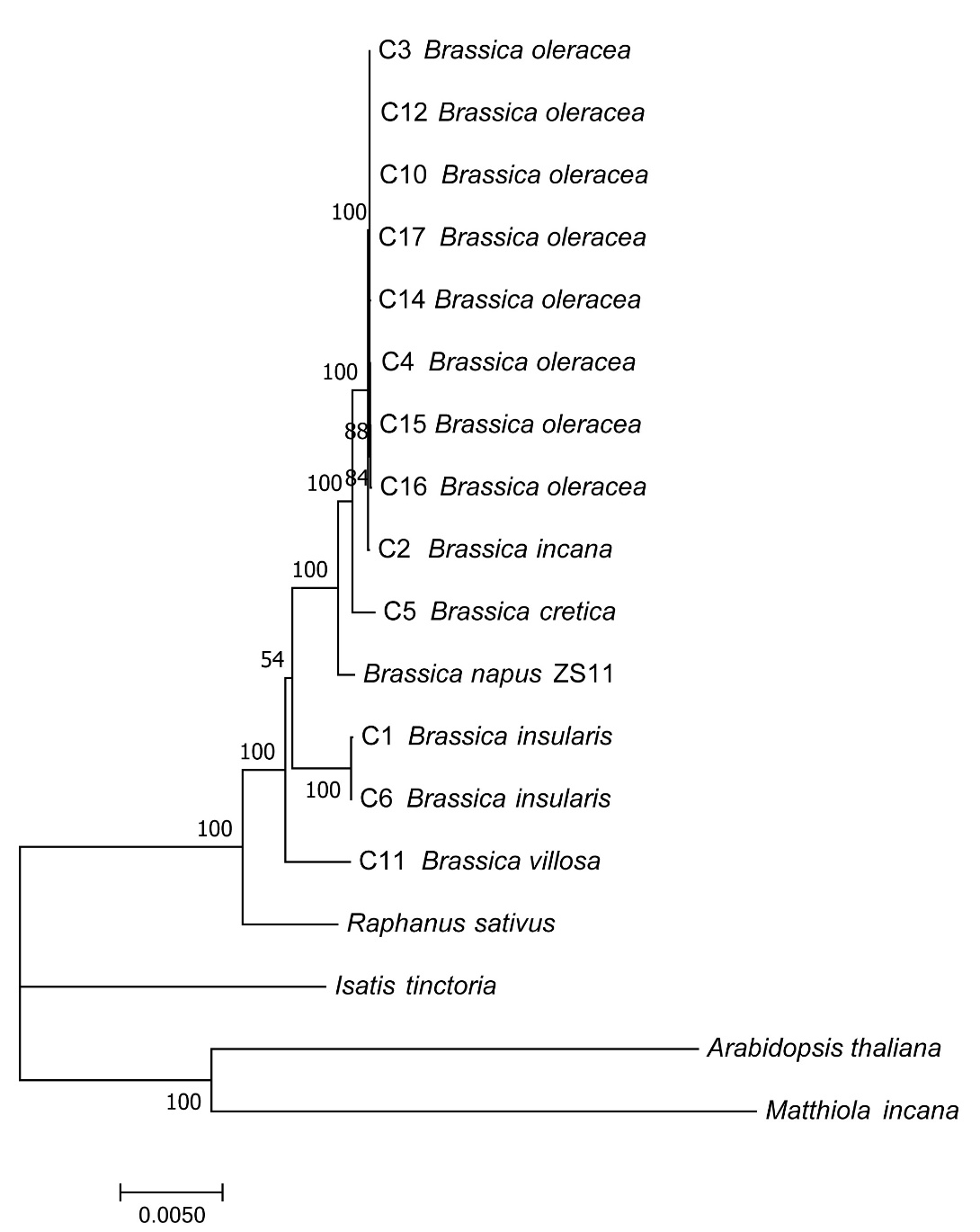


**Fig. S6.** Phylogenetic tree of *Brassica* C-genome species. This tree structure was inferred using Maximum Likelihood method based on the entire chloroplast genomes from representative *B. oleracea* materials.


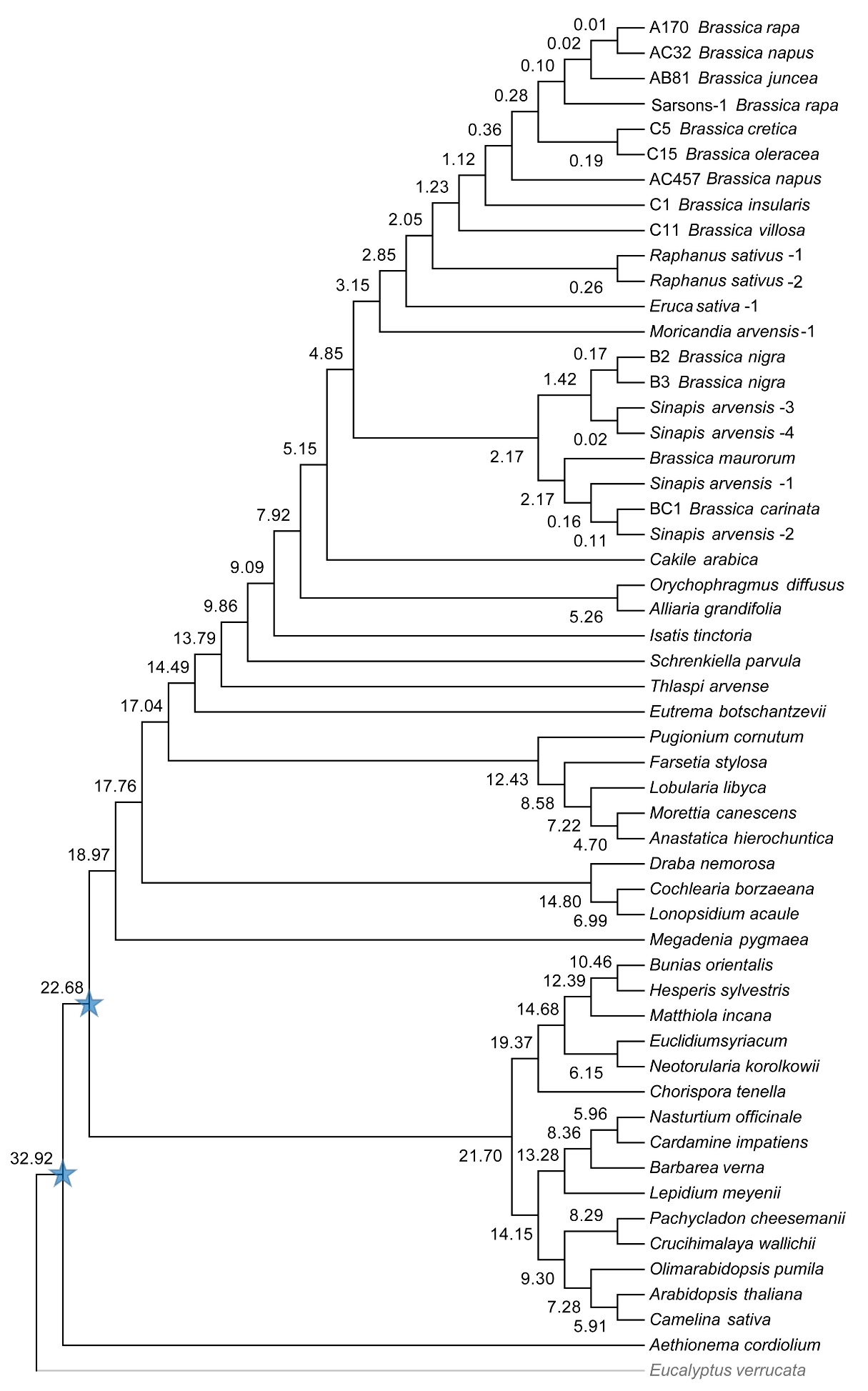


**Fig. S7.** Timetree analysis using the RelTime method. The timetree was computed based on the phylogenetic tree of *Brassicaceae* family in Figure 4 using two calibration constraints labeled with blue stars and displayed only with topology. *Eucalyptus verrucata* labeled with lightgray was set as outgroup.

**Additional file 3: Appendix B：**

**Accessions of public sequence data:**

Sequence data of the chloroplast genome sequences in the cruciferous cpDNA sequence gather can be found in GenBank under the following accession numbers: KJ872515.1 (*Brassica napus* strain DH366), KT581449.1 (*Brassica juncea*), DQ231548.1 (*Brassica rapa subsp. pekinensis*), KR233156.1 (*Brassica oleracea* var*. capitata cultivar* C1176), KT878383.1 (*Brassica nigra*), KJ716483.1 (*Raphanus sativus*), KT270358.1 (*Eutrema heterophyllum*), KR029093.1 (*Capsella rubella*), LN877386.1 (*Camelina sativa*) and LT161916.1 (*Arabidopsis arenosa*).

Sequence data of the chloroplast genome sequences used in phylogenetic analysis can be found in GenBank under the following accession numbers: KJ716483.1 (*Raphanus sativus*-2), KU050690.1 (*Sinapis arvensis*-4), KX343072.1 (*Cakile arabica*), KX756548.1 (*Orychophragmus diffusus*), KX342847.1 (*Alliaria grandifolia*), KT591187.1 (*Isatis tinctoria*), KT222186.1 (*Schrenkiella parvula*), KT962847.1 (*Eutrema botschantzevii*), KX886351.1 (*Thlaspi arvense*), AP009373.1 (*Draba nemorosa*), LN866844.1 (*Cochlearia borzaeana*), LN866848.1 (*Ionopsidium acaule*), KT844941.1 (*Pugionium cornutum*), KY912025.1 (*Farsetia stylosa*), KY912029.1 (*Lobularia libyca*), KY912031.1 (*Morettia canescens*), KY912021.1 (*Anastatica hierochuntica*), KX371593.1 (*Megadenia pygmaea*), AP009376.1 (*Nasturtium officinale*), KJ136821.1 (*Cardamine impatiens*), AP009370.1 (*Barbarea verna*), KY231152.1 (*Lepidium meyenii*), JQ806762.1 (*Pachycladon cheesemanii*), AP009372.1 (*Crucihimalaya wallichii*), AP009368.1 (*Olimarabidopsis pumila*), KX551970.1 (*Arabidopsis thaliana*), KX886352.1 (*Camelina sativa*), KY912024.1 (*Euclidium syriacum*), KX886350.1 (*Neotorularia korolkowii*), KY912030.1 (*Matthiola incana*), LN877376.1 (*Bunias orientalis*), KY912027.1 (*Hesperis sylvestris*), KY912028.1 (*Chorispora tenella*), AP009366.1 (*Aethionema cordifolium*) and KC180772.1 (*Eucalyptus verrucata*).
