# Supplementary material for: Comparison of the cytoplastic genomes by resequencing: insights into the genetic diversity and the phylogeny of the agriculturally important genus Brassica
Source: BMC Genomics. 2020 Jul 13;21:480. doi: 10.1186/s12864-020-06889-0 (PMC7359470; doi:10.1186/s12864-020-06889-0)
Supplement: Supplementary file 3 — Additional file 3 Appendix A The dataset for chloroplast genome sequences of 72 Brassica accessions. Appendix B Accessions of the public sequence data. [file 12864_2020_6889_MOESM3_ESM.docx]

**Additional file 3:**

**Appendix A** The dataset for chloroplast genome sequences of 72 *Brassica* accessions

# A22 chloroplast genome

ATAGAGAAGCTTAATACAAAGGCGGAAAAAGAAATCATAATAACTTGGTCCCGGGCATCACGGGCGAACGACGGGAATTGAACCCGCGATGGTGAATTCACAATCCACTGCCTTAATCCACTTGGCTACATCCGCCCCTACTATACTATACATCATATCTTGTTTGTATTGTCTAAAATAAAAACGCAGCAATATTTTTTTTGATAAAAAAAAAATTATAAATTATAATAGTATTTTTTTCTATTTTATATATAATAGAAAAAATTATATAAAAAAAATGATTTGTTCCGTTTTATAGAAAAAAACGAGCGATATAAGCCTTATTTTTAAGGCTTATATCGCTCGTTTTTAATATTACTAAACTAGGTCTAGACTAACACTAAAGAATTATCCATTTATAGATGGAGCCTCAACAGCAGCTAGGTCTAGAGGGAAGTTGTGAGCATTACGTTCATGCATAACTTCCATACCAAGGTTAGCACGGTTAATAATATCAGCCCAAGTATTAATAACACGTCCTTGACTATCAACTACTGATTGGTTGAAATTGAAACCATTTAGGTTGAAAGCCATAGTACTAATACCTAAAGCAGTAAACCAAATACCTACTACCGGCCAAGCCGCTAAGAAGAAATGTAAAGAACGAGAATTGTTGAAACTAGCATATTGGAAGATCAATCGGCCAAAATAACCGTGAGCAGCTACAATGTTGTAAGTTTCTTCTTCTTGACCGAATCTGTAACCTTCATTAGCAGATTCATTTTCTGTGGTTTCCCTGATCAAACTAGAAGTTACCAAAGAACCATGCATAGCACTAAATAGGGAGCCGCCGAATACACCAGCTACACCTAACATGTGAAATGGGTGCATAAGAATGTTGTGCTCAGCCTGGAATACAATCATAAAGTTGAAAGTACCAGAGATTCCTAGAGGCATACCATCAGAAAAACTTCCTTGACCAATTGGGTAGATCAAGAAAACAGCAGTAGCAGCTGCAACAGGAGCTGAATATGCAACAGCAATCCAAGGACGCATACCCAGACGGAAACTAAGTTCCCACTCACGACCCATATAACAAGCTACACCAAGTAAAAAGTGTAGAACAATTAGTTCATAAGGACCACCGTTGTATAGCCATTCATCAACGGATGCAGCTTCCCAGATCGGGTAAAAATGCAAACCAATAGCTGCAGAAGTAGGAATAATGGCACCTGAAATAATATTGTTTCCGTAAAGAAGAGATCCAGAAACAGGTTCACGAATACCATCAATATCTACTGGAGGAGCAGCAATGAATGCGATAATAAAAACGGAAGTTGCGGTCAATAAGGTAGGGATCATCAAAACACCAAACCATCCAATGTAAAGACGGTTTTCAGTACTAGTTATCCAGTTACAGAAGCGACCCCATAGGCTTTCGCTTTCGCGTCTCTCTAAAATTGCAGTCATGGTAAAATCCTTGGTTTATTTAATCATCAGGGACTCCCAAGCACACAAATTCTCTAAAACTATAAGTAGATAATTGAGAGCTTGTTATTGAACAGTATAACATGACTTATATAGCCATGTCAACCAATGTAAAATGGCTAAGATCCTTTTAGTTTAGATTCATAATAATTTTTTTATCGAGGAGAGAAATTATAAACGAATCTATATACATAGAAATATAATTTCTCTATGAATATTATTTCAAAATCATATGAATATGATCCATAGTGGGTTGCCCGGGACTCGAACCCGGAACTAGTCGGATGGAGTAGATAATTTCCTTGTTAAAATGAAAAAAAAAGTAAAAAACCCCTCCCCAAACCGTGCTTGCATTTTTCATTGCACACAGCTTTCTCTATGTATACATAGAAAACTCAGTTTCTTTGTTTCCTTATAAATAGGACTGCGAATTCAATACTCAGTAAATTTCATCTTAGTCTTACTGTATGAACATTTAATAATAGAAATAAATGACTTTTGATAATACAAAATAATTAATTTTTTTGTTATCTCCGCATTCCGTTTACGTTCTGATAAATTTTGATTTAATTTATGGAGCCTCAGAACCCCATTATTCATGATTGACTAAATCATTAAGATAAAGAATATCCAAATACCAAACCCGCACTCGATATAATCTTTTAGAAGCATAATCACTTCTTGGGAAGATTAAAGAAAGAACTTGGTCTTCCCCCGTAAGGAATTCTTCTAATAAACCCGAGCCCAACCTTTTTAAAAAAGCGCGTACAGTACTTTTGTGTTTACGAGCCAAAGTTTTAACACAACAAAGACGAAGTATATATTTTATTCGATACAAATTCTTTTTGTTTGAAGATCCGCTGTAATAATGCGAAATATTTCTGCATATACGCACAAATCGGTTGAGAATATCAGAATCTGATGAATCCGTCCAGGTCGCTTTACTAATCGGATGCCCTAATACATTACAAAATTTATCTTTAGCCAACGACCCAATAATAGAAGAAATTGGAATGTTGCTATCCAATTTTATTCTAACATTATCTATTAGAAATGAGTTTTCTAGCATTTGACTACGTACCACTAAAGGGTTTAGTCGCAAACTTGATAGATAACCCAGAAATTCTAAATTATCTTTAGATAATTGATTTATATTAACCTTTTGCGATTGAAACCATACGGAAAAATAACATTGCCATAAATTAACAAAATAATATTTCCATTTATTCATCAGAAGTGGCGTATCCTTTGTTGCCAGAATGTATTTTCCATGATATCGAACATAATGTAGGAAAGGATCCTTGAGCAACCCTAAGAGCGCCGAAAAATTATTAACAAAGACTTTAAAAAAATGTTGTATTTTTCCATAGAATAAAATTCGCTCAAAAAAGACGTCATAAGATGTCGATCGTAAATGAGAAGACTGCTTGCGTAGAAAAAAAAAGATGGATTCGTATTCACATACATGAGAATTATATAAGAACAATAAAAATCTTGGATTCAAAATTGATTTTTTTTTACTATCAAAATTCTTCCAATTGCAATACTCGTATAGACAGAACCGAAAAAAATGCAAAGAAGAGGCATCTTTTACCCGGTAACGTAGGGTTTGAACCAAGATTTCTAGATGGATGGGGTAAGGTATTAGTACATCTAATACATAATTAAAATGTGAGAGTTTGTCTTCTAAAAAGGGAAATATTGAATGAAGTGATTGTAAATTGTAAGATTTTTTTACATTTTTTCCTTCGATAGAGGATCCCAACCTTAGGGAAAATGGAATTTCTACAATCACTGCAAATAAAACAGATATCATTTGATAATAGAAATTATTGGTATGCCCCAGATTTTTGTTCAAATCCTTAGTGGGAATAATCAAACGATTCTGTTCGTACATTCGCAAAATTAAGCGTTTCACAATTAGTGAACTATATTTTTTGTCATAATCCGCATTTTCCAAGAAAATAGGGCGGTTTCTATTTAATCTATTTAAACCGTGATCATAAGCAAGTACATAAATATAGTCCCGAAAAAAAAGTGGATATAGAAAACTCTGTTGACGAGCCCCATCGAACTCTAAATATCCTTGAAATTTCTCCATTTGGATTAAAATTCGATTTGAACTAAAAGTAAAGTCTTTATTTTCTTGAGTTCTGAAATGACACATAGTGCGATACAGTCAAAATAAGGTATTAGATTACGAAAGCACTAAATACCTCATAAACAGGTAGACTGCTAACTGGATTCTCTATCTTTAATAGGTTTCTGTTCGTTATATTATAAAATAACAAAACAAGATGATTAGAAATCCTTTATTTTTTTAACCTAATCGCTCTTTTGATTTTGGAAATATATATATATATATTTTTTTTTATCAATATACTGCTTCTTTTACACATCCATCTACAACCTAACCCAAACGGACTAGGGAAAAAATAATTAGGACTCACGAAAAAATTGATAATAACACGCAAGAAAAAAATTCCTTCCCATACCCGTATTAGGTACTAATCTATTTTTAACATTTAATTAGATCGGGTAATTTTTCAAATTACGAATGGAAGCTCGTTTCTTTTTTTTTCTTAGAATAAGGAAAACTTGTTTTTTATCCATCCATTTATATTTATTCACTCGACCCAAATTGGAATTCTTCTTTTTTTTTTTTTTTTCGACAAGGTTGTACCGATTAGAAAATAAAAAAAATTTTTATCTGAATTCTCCCTTGATACGACATGCTATTTTTTCCGTTCATTCCTTTCAGGATCAGTCGTGGTCTTACAAACTCTACCGCGGATCTGGACGAATCCTTTTCTTCATACAAATGTGTAAAAGATGCTAGTCGCACTTAAAAGCCGAGTACTCTACCGTTGAGTTAGCAACCCCCCCCACAAAAAAAAGCAAGTACTGCAAATATGTAGATACAACCAGAATAAAGAAAAAAAGAAAAATCCAGTCATGTGTGCGTCAGGGAGAAATAGATCTATTTCTCTATGAGAGAATTATATTTGGTCCATACACTGTTGTCAATATGATTGTAAATTTTTAATATAGCGAAAAGAATAGAAAAAATAAAAAAGTTTAACCCCCTGGTTTGTGAGTTCATACAAGGAATGAAAACTAAGCCGAATAGGATAATCTCAAATCTTTCTATTTCTATATATATTATGATTCAGAATTAATATATTATTTATTATATTAATATATTATTTATTATACAGTATGATTTTATATATTTTCTATACAATAATTCTATACAATAAAATTTTGTATTTATACAAAATTTAGAATTTCTATAAACCCAAAAATTTTTTAATAAATTTGTTTTTTATTATAAAACATGGTAGTTTTTAGCAGGATATTTGTTAGTTTTCATACCTTTAGGAAGAATACTAATAATAAATGGAAATTCTAATAAATCAAAATAAATATGATGGAAACGAAAGAGGAGGAAAGAAAAGAGTAGATCAAATTTGATACCAAGCTATATATGAGTCTTTAACATCCTCTTTTTTATAGTTCATTAATTCAATTTCGTTTTATTAAGACTTAATTCCGTAAAAATCCCTGCCTTCTTTGAAATATCATGAACTGTTCTTGTTGGTTGAGCGCCCTTTTTAAGGAAATCGAGAATAGCAGGAAGATTTAAATAAGTTTGATTAGTTATCGGATCATAAAACCCCACCTTCCGAAGATCTCTTCCTTCTCTTCGGGATCGAACATCAATTGCAACGATTCGATAAATGGCTCATTGGGATAGATGTATATGAATAATACCCCCCCCCGAGAAACGTATACGAGGCTTTGGCCTCATACGGCTCGAGAAAAAAATGCAATGAGTATAAGTTAACTCTCTTTATAAAATTCAAACATTAAATTAATTAGCCAGTATTGAGTATTGAAACCCTAACTATTTTTTTCATAAAAAGCGTTCGTAACATTCGTACTCTCGTAACTCAAGTTAAATAACTCTCAAATATCTCAACAGAGACTCCTTAAGTACTCTTTTTATTGAGTAGTCTCTAACCTTTTTTTGTTTGTCTCATTTTTTCGAATCAATTTTGATTCTTCATTCTGATCTAGTTGTTCAAACAATTGAAAAAGGGATTTCCTTGTTTCAGGATTCTTTATCCTTACTTTGAATCTTTGGGTTTAGACATTACTTCGGTGATCTTGATCGTTTTATTAAAAAAGGGCAGCAACAAGCCCCTTATTTTGTTTATGATTTCTTTTCTTTCTATCAAAGAATCATACAAACGCTTGATTCACGCATGATAGACTTTTAATTCAAAGAATTTTAAAATTTTACGAAAATTTCCTTTTCCATTGTAAAATTACTTGAAAGGGCTTTTTTTTCAATATAAAAATAAAAAGACTTACGAAGTTGTTCCAACTTATTGATTCGCACTAACCCTAGATCCTTACTCCGGCGAAAGGAATAAAAACTTTCTATTCTCCTCGAGCTCCATCCTGTACTCTTTTTTATATTCAAAAAGGTGTAGGACTCTCGTAAAATAGAACACAAAATGTCGAGCCAAGAGCACCTATATTCCTAATATAAAAGGTGGCGGATCAAAACATCCACAGCAGATCATGTCCTTCAATTCAAGTCGCACGTTGCTTTCTACCACATCGTTTTAAACGAAGTTTTACCATAACATTCCTTTAGTTTGTGTAATTGATTCAATTATGGAATCATGAATAGTCATAGTTCAGTCAGTATATCGTAATCTATACTTTTTCTTTCTCTATGAATGGAATAGTGAATCTATGCGTAAAAGGTTCAGTCAGAATTCAAATGAATCCCACATTAAATTCTATATATGTAAAAATATGTAAAATCGAAATTTGAATAGAAATCTATATTTCTATATATAAATATATTTTTTTTTATTAAAACTCGTAGAATCTACGGTTCTACCTTACTTACCTACATCACACACAACTAAAAAAAGCAAATAGATTTTTTGTAATTTCGGGTGAAATAATGAAAAAGAAGTTTATTCTTCTTTTCATTTCAATATTTTATTCTTAAAAAATATTGTTTTTTTAAACAGAAAGAAAAAGATGGTGTACAAAGGCAATAGAAGATTTATTTCGTAATGACTGGACTCTGGGACGGAAGGATTCGAACCTCCGAATAGCGGGACCAAAACCCGTTGCCTTACCGCTTGGCTACGCCCCATTTTTATTTTTATTCAAGACTACTAAAAGAGTAATATTGCTATTGGTTGTTCGTCAATTCAATTTCAGCCCAAATGAAATATAGATTACATTGGTGCTATAGTTTTGACACGTGTAGATAGCAAATCAAACTTACTTTATTGATCATTACATAGAATTCAATTAAGATATTGTATGAAAATATTATTTCTTTCATTCTCTTATGAGAATGAAAGGATTTTTGATTGAGTAAGTTCAACAAAGTCTTTTTAGACTATCTTTCTTTATTTATTTATTTTTTTCCTTATATAAAAAATATATTAATAACTCAATCAAAATTAAATTATCCACAAGAACACCAATTTTTGTTATGCTTAATATATTTAATTTGATCTGTATTTGTTTTAATTCGGCCCTTTTTTCAAGCACTTTTTTAGTCGCCAAATTGCCGGAGGCCTACGCCTTTTTGAATCCAATCGTAGATGTTATGCCCGTAATACCTCTTTTCTTTCTTCTCTTAGCCTTTGTTTGGCAAGCAGCTGTAAGTTTTCGATGAAATTATTAATACTGTCTTAGAAAAATTCACGATTTTGATTCTTCCAACAATTCAAATCAAAAGATCAAAAAATCTTGACGTAGGAAGGAACTCTCAATTCAAACATTGAATTTTTTTGGTAGCCATACTAAAATCTGGATCATTTGATTTCCTCAGTTTTATCCTCTTTTCTCTAAATGAAAGAACTTAATTAGATTCGAGTTCACTCACAAAAAAAGTATCTAGATATTTAGTATAAAAATAGAGAATCTATTCTCTTTTTTTTTTTTTGAAAAAAAAAAAGTAAGATCTTGGAGATTGTGTAATGCTTACTCTCAAACTTTTTGTATACACTGTAGTTATATTCTTTGTTTCTCTCTTCATATTTGGATTCCTATCTAATGATCCAGGACGTAATCCGGGACGTGAAGAATAAAAAAGAAAGGTTTTTTATTACTTTAATTTAATATTTAAATAGTGGAAATGTGCGAATTTTATTAGGATTTTATCTATTTCACATCATCAAAAAGGGGAAGGGAAAGAGAGGGATTCGAACCCTCGGTACGATTAACTCGTACAATGGATTAGCAATCCAACGCTTTAGTCCACTCAGCCATCTCTCCTAATCGAAAAGGAATACTTTTTAGGTTCCATTAGACAAAAAAACGGCTTAAAAAAAAACTTTCTCCACTTTATTCTTAAAAAACTTTTTTTTTTTTAGATTATTCTTTAATAATACTTTAATTATATATATATTTTCATTTTCTATATTTTATTATATATATTATTTTATTATATATAAAAAATATAATATTATTTTTTCTTTTTTATTATCTAAATAATATTTATATAATATTTATATATAATTAATATATATATTACTATTATATAACCTTTTTTATAGAACTTTCTCAGTAATTCTATTTACATAAAAACTGTAAATAAAGATTCAATAAAGAAAAGGCTCGAAAGAGAAATAAATAAAATCACAAAAATAGAAATAGAGAATCCTTTTTGATTTTGTCTCGTCCAAACACAAATAAAAGATCTTTTTTATTTTAATAGCCTGGCCTGGTCAGTCCCCAGCCGGGCCTTTTTTTGTTAAAGTTAAAAAGACCCATCCGATGGGTTTTTAGACAAAAAAGATCTGAAATAAAAAAAAGGAATCCTGCTTTGCCTAATTTTATTAAGTCTACGCTAGAATTTTCTCATTTTTTTTTTCAGATTTTTTTTCTCCCGATTACTTTGTTCGACAAAAAGTAAATTTATATACAATAATTGGATTGTAGCGGGTATAGTTTAGTGGTAAAAGTGTGATTCGTTCCTTTAACCCCTTTAATAGTTAAAGGGTCTCTCGGTTTGATTAATCTTCCGATCAAAAACTTTATTTCTGAAAAGGATTTAGTCCTTTACCTTTCAATGAAAAATTCAAGGAAGATTATAGATTCTCGTAATTTGTATCCAAAGACTCTAATTAATTGTCAATTTGGATTATGAAATTTCGAAACATAATTTTTGAATTGGATGACTATTTACAATTCAATAAGTATAACAAGAGGATCCATGGATAAAGCCAGAAAAGTTTCTTTCTAATCGTAACTAAATCTTCAGTTCTATTTTTTGTTTGGTATAGAAAAAATTGAAGCAAAATAGCTATTAAACGAGAACTTTGGTTTACTAAAGACATCGACATATTATATTGTTTTAGCTCGGTGGAAACAAAATACTTTTCCTAAGGATTCCGTTAAATAGAAATAAAGAACGAAGTAACTAGAAAGATTTTTTGAGTTCGCCTTTTCTATCTTCTAGAAGGATCATCTATAAAGCAAAATTTTCTGTGAAAGCCTCCAAACGGGAAAAAAGCTAACATAGATGTTATGAGTCAAATTTTGATTTCGTTCCCATCTTATTTTATTTGGGAATTTCGCCATCCATCATAAAGGAGCCGAATGAAACCAAAGTTTCATGTTCGGTTTTGAATTAGAGACGTTAAAAATATAAAACTGATCGATCGACGTCGACTAAAACCCTTAGCCTTCCAAGCTAACGATGCGGGTTCGATTCCCGCTACCCGCTCTAAATTCTAAATTGTCCCCTTTTTATTAGAGACAATTTTCTCTATTAGAATTGTCTAATAGCAATTGTGTAGTGAATTCACTACACAATTGCTAAAAAGATTTCGCACATTTAACAAATGGGAAGTTAAAAAAAGCGAAAAGCGTCCATTGTCTAATGGATAGGACATAGGTCTTCTAAACCTTTGGTATAGGTTCAAATCCTATTGGACGCAATATCAATATAGATATAAATATATTGATATTTATCTATTATTTCCATTTCTATATATTATAAGAATATTTTTATTCTGTTTAGAAAATTTATAAAAAAGAAATATAAGTAAGAAATAAATTCTGAATGCTTTAAGATTTAATATTAAACATATACAGATAATTAGTTCTATACTTATATATATTATATATAGACTTAACTTAAATATACTTCTATTTTTTATAATTTCTCCTGAAGTAGAAAACGTTCCAGTTGCTCTTTAATACCTTCTTTCAAAAAGCTTTCTGCTTCAGCGGTTAATGTCTTGGTAGAGGCTATTATTTCTTGGAACTCAGGTTTATTTGTTTTTAAATAAGTGCGTAGCTGAACGAGAAATTTTCTTACTTGTCCAATTTCTAATCCATCCAGATAACCATTTGTTCCGGTATAAATGGTCATTATCTGTTCTTCCACTGTGAGAGGGGCTGATTGGGATTGTTTCAGTAACTCACGCAATCGTTGACCTCTTGCCAATTGATTCTGAGTAGCTTTATCGAGATCAGAAGAAAATTGGGAAAAGGCTTCTAATTCCGCGAATTGAGCCAATTCCAATTTTAATTTTCCAGCTACCTGTTTCATAGCTTTAATTTGAGCGGCAGATCCTACTCTCGAGACAGAAATCCCTACATTAATAGCAGGTCTAATTCCAGCATTAAAAAGATCAGCGGATAAGAATATTTGTCCATCTGTAATGGAAATTACATTAGTAGGAATATAAGCTGAAACATCTCCTGACTGGGTCTCGACGATTGGTAAGGCAGTCATACTTCCTTCACCTAATTCAGAGCTTAATTTAGCGGCTCTTTCTAAAAGACGTGAATGTAAATAAAAAACATCTCCTGGATAAGCTTCACGCCCGGGCGGTCTTCGTAATAGAAGAGACATTTGTCGATAAGCTTGTGCTTGTTTGGAAAGATCATCATAAATGATTAAAGTGTGTTGTTCACGGTACATAAAATATTCAGCCAAGGCGGCTCCTGTATAAGGCGCGAGGTATTGTAACGTAGCTGGGGAATCAGCCGTTTCAGCTACCACAATAGTGTAGTCCATTGCCCCTCGTTCCTGTAAACTAGTCACTACCTGAGCCACGGAAGAAGCTTTTTGACCAATAGCCACATAAACACATATTACATTTTGACCTTGTTGATTGAGAATTGTATCTGTGGCTACTGCTGTTTTACCGGTCTGTCTGTCACCAATAATTAATTCCCGCTGGCCGCGTCCTATAGGGATCATGGAATCAATAGCAATAAGTCCTGTTTGAAGAGGCTCATATACAGAACGTCTCGAAATAATACCTGGGGCAGGAGATTCAATTAACCGAGATTCAGAAGCTGAAATCTTACCTCGACCATCAATAGGGTTAGCCAAGGCGTTTATAACACGCCCCAAATAAGCCTCACTCACGGGTATCTGAGCAATTTTTCCCGTAGCTTTGACTGAACTTCCTTCTTGGATCATCAAACCGTCACCCATTAATACAACACCAACATTATTTGATTCTAAATTAAGGGCAATACCTATAGTACCCTCCTCAAATTCTACTAATTCACCTGCCATTACTTCATCAAGACCATAAATCCGAGCGATGCCGTCGCCCACTTGAAGTACGGTACCGGTATTTACAATCGTCACTTCTCTATTATATTGCTCAATACGTTCACGGATAATATTACTAATTTCATCGGCTTTAATGGTTACCATGAGTATTGTCCTAATTCTTTTTTAGAAGAAAAAAAAAAATAATGCCTATCATAATCGTAAGGAAAGGGCTAATCAGTAATTTCTTTCATCGTACCAAACATACCAATATTTGCATTAATAGTACGTAAATGTAACTCATTACTCAAACAACTATTTAGGGTTCCTATAGCTCCCTGTAAAGCTTGTTGGAAAACCCGTTCACGGACTTGATTAATTGTTCTTTGTTGCTCAAAAAGAATGGTTTCGTTTTTGTAATTTTCTAATTGTTTCAAAGTCCTAGAAGTTGAATTAATCAAATTGACTTTTTCTCGTTCGATTTCAGAGTATCCATTTACGCGAAACTGATCCGCCTCCATTTCTACTTTACGCAGGCGAGCTCGGGCGTTTTCTAATTGTTGAATAGCTCCTTCACGTAGTTCTTCTGAATTTCGAATAGTATTTAATATCCTCTGCTTTCGGTTATCTAATAAATCATTTAATGAAAGTAGATTATTCAGTAAAAAAAAAGTTCTATGATCCCTTCCCGAACCAAACATGAATCTTTCGATTCATTTGGCTCTCATGCTCACGTATTCCAATCATTTATCAATTATGTATGAGACTTTCATTCCCATATTTTTCATGTAATGAGCCTATCCTCTCCCAATTTTGTTGTATTCAATTCATATTCAATATATATTTCTATCGAAAAAGATCACCAATCCAAGACAAAACTATTTGGAGGATTCTTCTGACCAATAAAAAATTGATAATTGTCAGCAAAGTTGTTTCTTTTTTTCTTGAAATCCAAAGAATTTTTATTACTTTATACGTAGGTTATCAATTCTGCATTATACAAAAAGACTCAAAAATTTTTATCGACATGAGTGTTTTATATCGAAAAAAGCCGAACTATTCTTTTTGAAAATCTTATTCATTTTTTAATTAGACTACATATGGTAGAAAGAGTACCATGTTGCATCTGAACTTCAAACGGTTTAGTTTTAACCATGTTAATTAATGGTCCCAAATTTTTGGTTGATAGAGAATCAAAGTCAAGTAGACTTACCAAAGAATAACGAAATGCTATGGTTCTAAAATATGATTTTTTATTGAATTTTGTATTCAGAAGTAATTCGCGGGATTAGGCACTCTTTGCTAGTTATAGTGCCACTGGACGAATCCAGCCTATTCTTGAAATGAACAACTCACACACACTCCCTTTCCAAAAAAGATCAATACACCGAAGACTACACTTAGATTTATTGGATTTGTTGCTAAAATATCGGTATTAAACCCGAAACTCCCGGCGGATGGCCAGTGACCCAAGTAAACGAAAGAATCGGTTAAATTTTTCATATAATCTCCTCTTCTAGCTAAACTATAAAAAAAAAGAACTCTGTCCTTTTTTTTTTTATTCTTTGTTTTTTTGAATAAAAAGAAAATTTCGTTTAATAATTTATAATTTAATTTACCTATTTGGATATTTATAAACAGAATCAAAAACCTATTCTATTTACAAATTTATTTTCCAAAAATTTTTAATTTTCAATAATAATAATGAGACTTAATTAAAATTAAGCTAGAATTTGAGACCAAGTTTTATATCAATTTTAAAAAACCTAAACCTCCTTTTTGCGCAACACTCCTTAAAAAAAAATTTCCATTAAACTAAAAAGAATAAGGGGAAGGAAGAAAGCGAATCGATGTGTTAATTCCCCATCCTCAAATTAGTCCTTCCCAAGGGTTGTTGTCTCAATGAATAATTGTAGGAGTGAAATCTTGATAGAATAAAAAAAACTACGAAAAAAAAAATTCCTAATTTTATTATTTCTAGGATTAAACAAAAGGATTCGCAAATAAAAGCGCTAATGCTACAACTAGGCCATAAATTGTTAAAGCTTCCATAAAAGCCAAACTAAGCAATAAAGTACCTCGTATTTTTCCTTCTGCCTCAGGTTGTCTCGCGATACCTTCGACAGCTTGACCCGCAGCTGTACCTTGACCAACCCCAGGTCCAATAGAAGCAAGCCCAACAGCCAACCCAGCAGCAATAACCGAAGCAGCAGAAACCAGTGGATTCATGATAAGTTCCTCACACCAAAATAAAGAAATAGTTAATGATACAATCATCCAACGACTTAGGACTTAATTATAATTAAGTCATCGCTAAGATTCATCCAGCCAAAATAACCAAAAACTTGATAAGAATTACTTTGATATTAGTTCCTATCCACGGGATTTTGAAAAATGCATAATATATATATATACGACTTTTTTATGCCGTTTCTTTTTTGTGAACCATTCTTTTCTTTTAATTCTTCGTTCTTTTTTTGATCGTTTTTTTCAGCCAATTAACAGATAAAAAGTAAGAACTTATAATCGAATCGTTATCTAAATAGAAATTCACAAAAATAGTGGGGCAGATTATATAGATCTTTAACTTATATATACCTAGTCAATATCAAATATGACATATACAAGTGTTTCTTACATAACGTAAACCAACTATTCGATAATTGGGCTAACCTAAATTTGAAAAAAAAAATAGTTAATGATGACCCTCCATAGATTCACCTATATAAGCCGCAGCTAAAGTGGCAAAAATGAGAGCTTGAATCCCGCTTGTAAATAATCCAAGGAACATGACAGGTATAGGAACCACTAAAGGTACTAAAGAAACAAGAACAACAACTACTAATTCATCGGCTAATATATTTCCGAAAAGTCGAAAACTCAGTGATAGGGGTTTTGTAAAATCTTCTAAGATGTTAATGGGTAAAAGAATTGGAGTTGGTTGAATGTATTTACTGAAATACCCTAATCCTTTTTTGCTAAGACCCGCATAAAAATATGCTACTGATGTGAGTAAAGCTAAAGCAACCGTCGTATTTATATCATTCGTTGGTGCTGCTAACTCCCCTTGAGGTAACTGGATAATTTTCCACGGTAAAAGGGCTCCTGACCAGTTAGAAACAAAAATAAATAAAAACAGGGTTCCAATAAAGGGAACCCATGGACCGTATTCTTCTCCAATCTGGGTTTGACTCACATCTCGAATGAATTCAAGGACAAATTCAAAGAAGTTTTGGCCGCCAGTTGGAATGGTTTGTGGATTGCGAACCGCTAGAGCTGCGGAACCTAATAAGATAGCAATTACAACCCAAGAAGTAATAAGGACTTGCGCATGGACTTGGAACCCCCCTATTTGCCAATAGAAATGTTGGCCTACTTCTACACCAGATATCTCATATAACCCTTCTTTTATTAGTGTATTGATGGAACATGATAAAACATTCATATTGCCCTCTGACAGAAATAAGAACTTTAAATTATTTTGATTCAAGACCCCCCCTTTTTTTTTACTTATTTACTTGAATTTTCTATTTTAGTTTTGGATACCAACTAAACGAATCACACAATATACCCAGTTTTTTATCTCTTTTTCTTTTGTATGATTCAGGAATAGTAACCGATTTTATAAATCGAAATACAGGGAGCCCCTCCCTCAAAAAAAATTGATTTATTTATCTTATTATTAATCAAGAATTTTGTATATAGCTAGAACGACCCTCACAAATTGCGAATACTAATTTGTTAAGAATGAATCGAATTGAAGCTATAGCGTCATCATTTGCTGGAATAGAAATATCCGCGAGATCGGGATTACAATTTGTATCGATTAAAGAAATGGTTGGAATTCCCAAAGTTATACATTCTCGAAGAGCCGTATATTCTTCTTGCTGATCGATGATGATTACAATATCAGGCAATCCCGTCATATATTTAATCCCGCCTAGATATGTTTCCAAGCGAGATAATTGTCTCTTCAACACAGCTGCATCCCTTTTCGGAAGACGGTTGAATCCCTCTGTCTTTTGTTCAGTTCTCAAGTCCCTAAACTTATGAAGTCTTTTTTCTGTAGTAGACCAATTTGTTAACATGCCGCCGAGCCACTTTTTATTAACATAATGACACCGAGCCCTTATTGCAGCCCGCGACACTAAATCAGCTGCTTTATTTTTTGTCCCAACAATTAAGAATTGTTTTCCCCTACTTGCTGCATCAAAAACTAAATCACAAGCTTCTGATAAAAAACGAGCAGTTCTAGTCAGATTTATAATATGAATACCTTTACGCTTTGCAGAAATATAAGGTGCCATTCTAGGATTCCATTTCCTAGTACCATGCCCAAAATGAACTCCTGCTCTCATCATCTCTTCCAAATCGATGTTCCAATATCTTTTTGTCATTTCTTTTCACACTTAAAAGGGGGGTACCCAAAACTAAAATAAAAATTTGTTCCAATGGAACCTTCTCTTGTCCGTTTATGCACGAGCCGAGCCATTATTTTGTATTCATTATTATCTTTATTAGTGTTAACAAATTATTAAAGCAAATGACTACAGCAAACAATAAAACATGAAATTCAAAACAGGAATCTGCTATTAGGAATTATTCAATTCTAGAAAAGGCAGATTTGTAAATAGAAGAGTCACAAAATTCCCTGTGATAAAATAAAATATCTCTCATATCTCCCTCTAATAAAGATAAATTCTTTGTTTTTTTTTCAAAAAGAATATTGGTATGTTGCCGTGAACAATGCACCAATCCTTTGTTGAACCCGGTCCCGGCGGGGATCACACCCCCTAGAACAACATTTTCTTTCAGGCCTTTCAACCAATCGATACGACCCCGAAGAGCAGCTTTTGCTAAAACTCTAGCAGTTTCTTGAAAACTTGCTTCGGATATAAAACTTTGAGTATTCAAAGATGCTCGAGTTATTCCTAATAAAACGGCTCGATAACAGATTGCTTCTTCTAAAGCACGCCCCGTGCGTTCTGCTCGTAACAATCCAATCAATTCTCCAGGTAAAAAAACATTAGACATTCCCTCTTCTGAAACCAAAACTTTTGATGTTATTTGACGTACAATAATTTCGATATGCCTATTATGAATCTGCACCCCCTGGGATCGATAAACCTTTTGAATCTTATTAACCAAAGAAATACGACTTTGCACTATAGTTAGCTCAGCACCAATCAAGAATCCCCAAGGAATTCCAAGAATTCTTGTTATACACCTGTTCCAACCCTTAATCCGCTTTTCTAAGTTCAGTGATATTGAATCAATCGAGCGGACTTCTAACACCTGTTCTACTTTTGGAAGACCTTGGGTTATATCACCGGATCTCGATTTTTCATATATAAATGTAACTAATGTATCCCCTTCGTAAAGAATTTCTCTATAATGCCCGTGAACTTTTGCTCCCGGAGTAGCCAAATAGGGCTTAGCGGATCTTATTACTACAGAATCCCTTTGAACAATTAAAACTTGACCCGATTTTAGGTATGGTTCTTTTTTGGCTATACATAGATTTTCACAAAAAAATTGTCCAAGACTTATTATTGTGGACGTTTCCTCACAATAATAATTATTATAATTTTGATGAAGAAAATACCAATTCAATTTGAATGGATTCAAAACAAGGTTACTGTATGGATCTAGATTAAAAATTCTTCCGTTTTCATCTATTAAATAAGAGTGAATTATTTGAAAAATATATTTGAAGTTATCAAGTTGCAAATATTTAATTACAGAGATCTGATTATAAGTTAGTAAAGGCAAAAATGAATAAAAATTCGAAATTTGAATGGCTGTTCCTAAGGGGCCCGACGAATTTTGAATTGTAATTAGAGGTTTTTTTTTTATTGATTGGTTTATAACATTGTGATATTTTACATGATTAAATGGACCGATTCTAAAACAATTAGAGGATGATAAAATTAACAAAGATTGGGATTCCTTATTTCTGAACATACGAATAGTTCCGTGATTTTGTCTAAGCGATTGTTGAAGAATGCCAGCCTTGGGAGAAAGCGAATAAAACGGATTCACGGAATCTGCAGAGATCAATCCCGAATCCGGCGGATTATTCCTTTTTCTTATATACGAAATATGGGATTTCACTAAGCCAATTCTTATGAAATCTCGAATCAAACCCTTTGTACTTACTTCAACAACGAAAGCGCGGACCTCCTCGAGGGAAGAATTTTTGTTGTCTTGGTCCCAATTCAAGACTAAACAAGTGCGAACCAATTGAATACTTGTGTCAGAAATTCCTCGAGTTGGTTTACCATTTCCATAAAGGATATAGTTGAAAACTCGAAGTTGAATATTATCCTTTTCCCGAAAGAGATCTTGTGGGAAGAGTGTTGCTAAATTTATACTGTCCATTATCTCATAGGTGGCTACGGGCCGCACCAAAACAAAAAACTTTTTCTTGGTTGGTGTGATCCGTTGGGCATAAATCCAATTTTTTAAATTTTTTGATTCTTTAGAGTTTGTTTTTCCCCTTCCTGGCGGTATCAAGATGCCACTATGTCGGGATATCTTATCTGTCTTGTCCGGAAAATGGATATCCCCCGAAAATATTTTGAGTTCAATCCTTTTTTTTTTTCTCTCCACTCGGATCAACCCGCCGACTTGGCTTCTTATATTTAAAGTGATTCGTGTATCGACTCCAATGATACTATAGTTCTGTACCATTATGGCGGAGGATTCGGGTAAAATATGCACTTCCTCAGGAATGAAAAAAAAGCGATCTACTTTCATTTCGTATTTTGTCTTAAATTTTTGGACTCCTCGATACTCAATCATATCCTCTTTTTGGACGATTGAGTCCGCCTTTAGAGTTCCATATTTAAGAATTCCGGAACTCTTTCTTCTGTATCTAGGATCATCAAAAAAAGCAAAAATACTGTTTCTACGGAAAATACCATTTATGGGTATTTCAATCGAGATACCTGAATGTGGTATGAACTCTTTCGCTTGCTCTTGAATCGATTGGAATGGAATGAGAAATCTATTTCTTCGCCTTTTTGCTAATAAATCCGAATTCTCATGAAAAATAGCAGAATATATGAAATTATAATGACTAGTACCTACGATTCCATTCAATTCTGAATAATTGGGAATCCCAGATTTTTTTTTATCAGAAAAATCTGAACTGAAAAATTTTTTGCTCACTTGATCATTATTCACTGAGAGGCTAGAAATAGATTTTCTTTCGACGGAAAGAAAGGGTATGTTCATTTGATCTTGATCTTTGTGGATCGAAAAAAGAATTAGACTAGATCCACAAGAACCTCCTGATAATATCCATAAATGACTTGTTTTTGGTAAAAGATGGACATTACTATATGTAAATTCGGGTGCATGGGATACATCAGTACTCCAATGCATTTCGCCCTCGGAGTCAGAATAAATATATTTTCTAACCCTCTCTTTAAAATGAAAAGTGGATGTTCCCTCGCGAATCTCAGCAATCACTTGTTCTGATTCCACATATTGATCATTTTGAACTAAAAGAAAACTTTTTGGTGGAATAGTCACGCTATGTATAATATCTTCGCTCTCAATAATTACAGACAAGTCTATATAACATAGAAAGGCAGGATGCCCGTGACGTGTACGTGTAGGATGAACCAAATCCTCATTAAATTTGATTTTTCCATTATAAGGGGCTCGTACATGTTCGGCAGTACCTCCTGTAAATACTCCACCGGTATGAAAAGTTCTTAATGTTAGTTGAGTCCCCGGTTCGCCAATAGATTGACCCGCAATAATACCTACAGCTTCCCCCAATTCAACTAGGTCACCATGAGTGGGACTCCGGCCATAACATAATCGACAGATCCAAGATGTACTCCGACAAGTAAAGGGAGTTCGAATAGATATTGATTGTGTTCCAAAGGTTATGAATCGATTGACAAGTCCAATCCCAAGATCTTGATTTCGAAAGGCGACACATCGGGAACCTATATATATATCGTCTGCTAAGACACGACCAATTAATGTTTGGATAAAAATTCTTTCTGACATCATCCGACTTTTATTTCGAGGACTCACAGAAATCCCTCGGATAGTGCCACAATCCGTTCGACGTACAACAATATGTTGAACTACTTCAACAAGTCGACGCGTAAGATATCCAGCATCTGATGTGCGGACCGCAGTATCTACAACTCCTTTACGGGCTCCATAGCAAGAAATAATATATTCTGTTAAAGACAGTCCTTCGCGTAAATTGCTTTGAATAGGTAAATCAATCATTTGTCCTTGGGGATCCGACATTAATCCTCTCATACCTACTAATTGATGTACTTGAGATGCATTTCCTCTAGCTCCCGAAAAAGACATCATATGGACTGGATTGAAAGGGTCCGTCATCCTAAAATTAGGATTCATTTCCTGTCGCAAATATTCACTTGTAGCATACCATATCTCAATAGATTGGCGTAATTTTTCTACCGCATGTACATTCCCATAATGATGGTGTTTTTCCAAAATCAAACTTTGTTGTTCAGCATCTTGGACAAGCCAGCCCTTAGAAGGTATCGTTAAAAGATCATCAATTCCTAATGAAATGGATGTAGCAGTTGCTTGCTGGAAACCCAGAGTCTTTACTTGATCTAGGATGTGTGATGTATATGCCATCCCGAAGTGATCTATTAATCGGCTAATAAGTCGTTTAATAGCAGTTCCATCTATCACTTTATTGTGAAATACCAGATTGGCCCGTTCCGCCATAAGTACCTCCATATTCTGCTGAATGGGATTCGACAATGAGTTTGAGTCAATGATTGCAAAACTTCCTTTTCTCGATCTTGATTTTTTAGGTCAGGAACTATGTCCGAGTTGACTCGGAGAGGTCCGAATTCACACGGGTGTCCTATAATTCTTTTTTATGAATACCATATTATTAGGTATCATATGAACAAGCTTGAGAAAAACCTTGTATAGCTTCCTCGATTTCTCGATAAAAAGAAATATGACCAACTGTGGTTCGAATATATATAAAAAAAGTTTGTTTTTTTACACTTCTTACTATCAGATAGTGTGCATAAATCTCATGATAGTTACCAAAAGATTCATAGTGAACTTCGATAGGAACTTCTTTTGAAGCAATAACGCGTTGATCTAATTGCCACCGAAGCCACAAAGGACTATCTAAATTGATTCTTTTCTGCCGATAAGCTCCAATTGCATCATAGGAATTGCAAAAAAAGGGTTCTTTCATATACTTATAGTTTGTTTCGTAAATTCTTTCATTTTGATAGTTTTTTCGATTACATGGATTATATCTGTTTGCACAAATACCTCGACGAGTGCCGCTCGTTAATACATAGAGTCCAATCAGCATATCTTGAGTCGGTACAGAAATGGGATCTCCAATAGCTGGAGATAAGAGATTCATATGAGAAAACATAAGTAAACGAGCCTCTGCTTGAGCTTCTAAAGATAAAGGCACATGAACAGCCATTTGATCCCCATCAAAGTCTGCATTGAACCCCTTACAAACTAATGGATGTAAACAAATAGTGCGTCCTTCCACTAAAATGGGTTGGAATGACTGTATGCCTAATCTATGTAGAGTAGGTGCTCTATTCAGTAATACGGGATGCCCCTGCATAACTTCTTGAAGGATTTCCCAGACAATCGGCTTTTTTTCACGAATTTGACTCTTAGCAACTCCTATGTTCGAAGCCAGATGTTGTCTAATTAGACCACGAATTACAAATGTCTGGAAGAGCTCTATTGCTATTTCGCGAGGCAATCCACAGCGATGTAATGAAAGTGAGGGTCCAACGACAATCACCGAACGCCCCGAATAATCGACCCGTTTGCCAAGCAGAGTCTCGCGAAATCTTCCCTCTTTTCCTTCAATTACATCTGAAAATGACTTGTAAACCTTATTATGACCATCCCTCATGGGTTGTCCACGGATTCCATTATCAAGAAGTGTATCCACGGCTTCTTGTACCAATTTTTCCTGACACATTACTAATTCCCCTGGTGTAGATCTACTTGTTGTTAATAGATCAGTAAGAGTATTGTTCCGATAGATAACTCTTCTATAGAGTTCATTAATATCTGAACTCATCAGTTTACCCCCTTCTATCTGAATGATGGGTCTCAACTCGGGAGGCAGAACCGGTAAGAGACATAAAACCATCCATTCCGGTTCTATATTTGTTCGAATAAAATGCTTAGCTAATTCCATACGTCTAACTAAAAAATCTTTTCTTCTTACAATTTTTCGATCTTCCCATTCATTCCCCGTGGGACCTTCTTCTCCTAATTGTTTCCATTCTACCAACGAATTTTCTATAATAATTCGCAAATCTAAATCGGCTAATTGTTCTCGGATAGCACCCGCCCCAGTAGAAATTTCTCGATTTCTAAATATATCGAAACCTTGAGTAGTAAAAAAAAGTGGGATGCTGTATTTCCAGGATTGAATTTCATATTCAAATGAACCTCGTAATCGTAAGAAAGTAGGTTTTTTCGTTATGGGCCTAGCAAAAGAAAAATTGGGATAGGGTCCACTATATGATCTCCCCCCCTCAAAACCGGACATGAAAGTTTCCTCTCATCCGGCTCAAGTAGTTATATCAAATAAAGATAAAGAAAGGGGTCGCACTTTCCAATTGTATTTTATAAAATCAAGTGAAAACCCAAAAAGAATCTACGCCTTACTCAAGTTCTCAGTGCAAACCAACCACCATTTCATTGATTCAATTAATTCTTCTTTGATTTCTATTTAGATTCTTTAGTGAATTCAAAATTACGACAGAAAAAAGAAAAAAATGTCAAATTCTTGAGTAGTCTACTTCCCTTCGAATGCCGGAATACTTTTTACCTTAAGTGAAAGGAATGCCTTAGAATTCATACGGGATTTATTTGTCTATGTATTGTTCCATTCGATCTTTTAGGTCCTGCGTTACCTCGATGGTTATGCCACAATATTCTTAAAGCTTATATGCGATGTATAGACTTCTCCAACCATGACATATTTGTTTACTTCAATATAAAAAACCAAATTTCTTTTCGTTTAGAAAGATAAGGGAATGCTTAATTCGACAAAAAAAAGGTCTTCTTTTCACGAGGTACGACTATCAATTTGAAGTTACTTTTTTTTTACTGAATCGACCATAGACCAATCGCCCTTGTTATTTGGGAGTATTGAATACACCCACAAGTCTGAGCTTCATGTTACTCTTTTCAAGAGACATGTCAGATCGAGGGCATCCCAAATTGATTGAAGGGGATGAGAGTTTATCATTCTTAAAAATAAAAATTTCGATCAAATCACACATCGCAGTATACTAGACCTTCTAATTCTTTAAGAGGTTTATCTAAAAGATTCGCAATATAACTAGGAAGACGTTTCAAATACCATACATGAGTTACAGGACATGTCAGTTTTATGTATCCCATTTGATATCTTCGTATCCGAGAATCAACAAATTCAACTCCACATTGTTCACAAAATTGCGAGTCTTCTTTTTCATCTCCGATCACTCGATAATTTCCACAAGCGCAAATTCCACTCTTTATAGGCCCAAAAATCCTTTCACAAAATAATCCATCTTTTTCCGGTTTATTGGTTTTGTAATGAAAAGTATAGGGTTTTGTCACCTCTCCAACTATCTCTCCATTAGGTATTTTTTTAGTGGCCCAAGCACTTATTTGCTGAGGAGAAACTAATCCAATTCGGAGTTGTTGATGTTTATACCGATCGATCATATAAGAAATTTTGTGATTCATTCCGATTAAACTTCCTTCCTATTAATCTGGAAATTCTTCTCAGATACAAGGAAATGATTCAGTTCCAGAGCCAAAGATCGTAGTTCTCGAACAAGTAATCGAAAAGATTCTGGAGCATCTTCTGGTTTAGGTATTGCTCCTCCAATGATAGTGGTACCAAGTACTTCTTGGCGAGCTCTAATATGATCAGATTTATAAGTAAGCATCTCTTGTAAAATATGAGCAACACCAAACCCCTCTAGAGCCCAAACCTCCATTTCGCCTACCCGCTGCCCCCCCTGCTTAGAACGGCCTCTAAGGGGTTGTTGTGTAACAAGTGCATAATGTCCACTAGAACGTCCGTGTATTTTATCATCAACCTGATGAATTAATTTCAAGATATAGGGCTTTCCTATTATCACAGGCTGTTCAAAAGGATCTCCCGTTCTTCCATCAAAAATGCGGCTTTTTCCTGGATACTCGGGTTCAAATACCCATGGATTGGCTGTTTGCTTACTAGCTTCATATAATTCAGAAAATACGAGTTTTCTCGAAGCCTCTTGTTCATATCTCTCATCAAAAGGGGCTATTCGATAATGTCTATCTAGCAAACTTCCCGCTAATCCAAGCGAGCATTCAAATATCTGTCCTACATTCATGCGTGAGGGTACTCCTAATGGGTTGAAGACCATATCCACGGGTCTCCCGTCTTGCAAATAAGGCATATCCTGTCTAGGCAAAATTTTGGAAATGATACCTTTATTTCCATGTCTTCCGGCTACTTTATCACCTACTTTGATTTCACGTTTCTGTGAAATATATACACGAATTATTTCGGGGTTATAACTTGAACCCCCCTTTTTCTGAACCCATCTCACATCAATAACTCGACCTCTACCACCTATAGGCAATTTTAAACAAGTTTCTTTTGAAGTCGATACCTGAATGCCAAGTATGGCCCGTAATAATCTATCTTCCGGAGCATACGAGGATTCTTTCGCCATCTGAGGCGTTAATTTACCTACTAAAATATCACCCGTTTCAACCCACGATCCTAGCATCACAATTCCATTTTTGTCTAAATTTCGGAGTAAACGGCCCTCTAGATGCGGTATTTCCTTAGTGATCCTTTCAGGACCTTGGGTTGTCACATGCGTCTGAATTTCATATTTCCGTATGTGGAAAGAAGTATAAATATCACCATATACTAGACACTCACTAATGAGTACCGCATCTTCAAAATTGTATCCTTCCCATGGCATATAAGCCACTAATATATTTTTCCCCAAGGCGAGTTCCCCACCAACTGTAGCAGCACCATCCGCTAAAATCTGTCCCTTTTTAATACATTTACCCCGGCGAACCTGAGGTTTTTGATGCATACAAGTATTTTTGTTTGAGCGTTGATACATAATTAATGGAATACTTAAAGTATTCTCATTTCCCGATAAAATTATCTTCTCAGTGTCAGTATAAAGGATTTTTCCCTCGTGTTCGGCTATAGCGGGAACCCCCGAATCTAAAGCCACTTGGCGTTCCAATCCAGTTCCAACAATGCACTTTTCGGACCGAGAAAGTGGAACTGCTTGACGTTGCATATTAGAACTCATTAAAGCTCGATTCGCATCATTATGTTCGATAAAAGGAATTAGGGAAGCTCCAATGGAAAAATATTGGAAAGGAAAAATGCTTCGAAGATGAACCTCTTCCCATGCGATAGTCAAAAATTCTTGGCGGTATCGAGCTGGTACAGCCTGTTCTTCTTGAATGCCCCGATTAAGAGCCAAAGAATTTCCTGCCGCTATCATATAATATTCATCTTGACTTGGTGATAAAAAAAGCATCCGTATCCGCGCCTTTTTTGATTTCTCAACGAGTTCATAAAACGGACTTTCTAACGACCCCCAATCACCAATCCTGGCATGAATTGATAAAGATCCAATAAGTCCCACATTGATTCCTTCAGACGTGTCAATGGGGCAAATACGCCCATAGTGACTAGGATGGATATCTCGTATTCGAAAATTAGCAGTTCGCCCTGTTAATCCGCCAGGGCCCAAATAACTCAACTTTCTCCCATGAACGATTTGTGTCAATGGATTAGTGCGATCCAAAACTTGAGATAATGGATGTAATCCGAAAAAGGATTCATAAGTAGTTGTTAACGGAGTTGAAGTTACCAAATTCTGAGGAGTCGGTATCAATTTATGCCTAATTGCTCCGCCTATAGTTCCCTTAACTACATTTTCTAAACGAGCCAGAGCCAACCCGAGCTGGTCTTGTAAAAGATCCGCTACAGAGCGAATACGTTTATTTTTCAAATGATTCATATCATCAAGTGTACCCATTCCAAATTTCATCCCAATCAAATGATCGGCAGCTGCTAATATATCTCGTGGTAACAAAAATATATTGTTCTGAGGTATATTAAGATTCAGTCTCCAGTTAATATTTCGGCGACCAATCCTCCCCAATTCACACCTTTGGTGAAAGAATTTTTTTTGTAATTCCTTACATAAGGATTCAGAAAATATTGGATCCCCACCTACACAAGAAAATTGTTGATAAAACTCCAAAATAGCATTTTCTTTTGACCCAATTTTTTTTTTCTCCTTATCGGTTAAGAAAGATAAGAAAATTTCAGGGTAGCAAACATTCTCTAGAATTTCTCTTAGATTCGAACCCATAGCTGATGATAGAACTAGAATAGATATTTTCTGTTTCCTACTCACACGAGCCCATATTCTTGCTTTTTTATCAATCTCTAATTCTAGCCTGCCCCCCCAATCTGATATTATGGTGCCGGTATAGACCGAAATCCCGTTATGATCCAATTCTGACTGGTAATAGATACCAGGACTTTGTAATATTTGATTGATCACAACTCGGTATATTCCGTTTACTATAGAAGTTCCAAGGGAATTCATTAAAGGAATGTTTCCAATAAAAATTCTTTGTTCTTGCATATTCCTATTGGTTTTCCAAATTAATCCCGCGGATACATATAATTCAGAAGAATATGTAAGTAATTCATAGACAGCATCTCGTTCTTTTATCAGAGGTTCTACCAATTGATATGTTTCCACAAATAATTGAAATTCAATTTCGTGATCTATATCTTCAATTTTTGGAAATTGCGAAAGTTCTTCTATTAAACCCTGATCAATAAACCGATAAAACCCTTCAAATTGTATCTGATTAAATCCGGGTATTGTAGATGTTCCCTCTTTTCCATCCCCGAGCATCTTTTTTGAATTTATCATTTATCCGTTTATTTTAAAAATCCCATATCTCATTCTTCACCGAATCATATAGATAGAATTCGATCTAGCAATAATGGAATTTCTATTCTGTTTACTGAATCACATGAAATTTTATCCAACTCCAAGATATATGGAATGTATGAAATCCGTATGAACGGAGACTAGATTCAATTGGAATTTTTTTTATAAGAAAGAGATCCAAATGGAACAGAATTTAGAAATACCGCTGGAACTTATGGAGTTTTGTAACGACTAGAAAAAAAGTAATTTCATTTTCACCTATGATATTACATATTCCAATTCGATCGCATACCATAAAAAACGGTATTCATGATAGGATCTGTTCGAGCAGATAAACATATAAGAAATAGAAAACTTTTTTTTAAACACTTTACTTTTTCATGTATTTGTATTTCATTGTTCAAAAAAATAGTTGCAGAAAAAAGATGGATTTTTACCTATTTTGAATAGAATATTTAGAATATCATTGAATTGAAGTAGGTAAGAAAACGTATGTTTTTTTATTTATTAATTTTTATTATTATTAAAATAAAAAAGAATGCACAGGTATATATATATGTCTCTTTTTCTTCTTTTATTGTGGTACAGTTCTATTTGGAACAGCACATGCTGTGCTCTACCAAAAATTAAAAATTTTTTTTCAATGTATTCAATGAAAAATTTCAATACAAAAATTTATTGAGAATTACTCCTCAAAAGCATCCCTAGAGAGATAAAATACCCCATTATAGAGCTATACAAGGTAACGTATGTTCTGATTCTGGGGTTTACATATACTCATTATTAGTGTTATAATTCAAATGGAAGAAGATTTCTTTTTAATTGAAAAAACTCAATATAGATTAGTTATAAATCTATTTCTAATGATTTTCTTATCTTATATTATTAGAAATAAAAAATGTAAATTTGAATTCAAAAAAGGTCATGAATTTACAGTCAATAGTTAATGGTTCTGATTTGTACTAGATTCTATATTTTGTGACTGAAAATCTATATTTTTTTCGGAGTTGAAAAAAAAAACAAGAGAAAATTTGAATCTAGTACAAATCATTTTGGCGGCATGGCCGAGTGGTAAGGCGGGGGACTGCAAATCCTTTTTCCCCAGTTCAAATCCGGGTGCCGCCTCAACAGGAGACTTGAAATCTCCTGTTATAAAACTATAACAAACGTAGGAAAAGACTCTTGATACTTTCTTTTCGTGATTCTAAGCCCCTGGCTCTCGAGGTTCTATTCTCTAACCTAAAGTTTTACCTATCAGATTAGAGGAAAACTAAAACGAGTGGAGGGAAATCCATTAGATTGGATAGGCAGAGAGGGAATTAAATTAATAGTTTTGGAAAGGATCTAAGATACTTTGGATATAGACTCATGAAAGTGGATATAGACTCATGAAAGTGGATATAGACTCATGAAAGTGTCGGAATGCTCAGACATTCAATCAATATTAGATTAGATGAAGAATTGCCTTTCGTTTTACTTCAAATAAAAATAAAAAACGATAAAAGAAAGAAAAAAGATTATTCTTTCTACATATGAGTCAGATTTTTTGGATACTTCGAAAAGTATCTGTTTACTTGTGTTTACATCTTGTCGATTCTACTAGAAATTCTATAATTAAGAATAACTCATTATAAGATAAGTGGATTTTTTGGAGTAGTTCATCAATGGTGACCAAATATCTCTCCCTTTTTTTGACTCTGCACCAGTGATTTCACTATTATTAGTGAACAATAATGGAAAAGTTTCTTCATATTCATAGGGGACAGAATTCACATGGATATAGTAAGTCTCGCATGGGCTGGTTTAATGGTAGTTTTTACATTTTCCCTCTCTCTCGTAGTGTGGGGAAGAAGTGGACTCTAGAAGTACTCCTAATTGCGATAATAATCAAACTCTATCAACCTGTATCAATTGTTTTAGTTTTCTAGACCGGCCGGCAATTTTTTTTAAGATCTTTTTTTAGAAATTGGATTTATGTTTTGTTTTATTGACTCATTTTTTTTTTTGATATCAGAGTTTATACCGTTAACCATTCATGGGATAACCCCCTTTCGAAATCTCAAGAGGTTTCCATCGAATTCGGATTATCCGTATTAAATGGATCAAACAAACAAATGAAATTGAGAAAGTATGTACATAGATTTCATATTCTATATTAATTTATATTTACATTTATAAAGAAAAAGAGAGATATGGGTGGATTCCTTTATATTAAAATATTTCACTTGTATCTTTATACATTACAATAACCATAATGGCTAGTATGGTAGAAAGAGATCTCTTTCTACCATACTAGCGGGCCCCTTAGGATACTACTGAATCTAATGCATTCCTTTCATTTAAGACGAGAAATTGACATCCTTTTTTGTCATTGATAGTCAAATTGTATTCAAATTAATTATTTTGACTAACCGTTTTTACGTAAATTATAAGCAAAAAAGCAGTAGGAACGAGAATGAAGAGTGCAGTAGCAATAAATGCAAGAATATTGACTTCCATAATTAAATCGTTTATTATTTATTTTTTTTTCTTTGGAATATCTCGGGATTTAATCCCATAGAGATGAGAAATCTTTCGCTTGTAAACTCACTCAGATGAATTAGATTTCGATGATATCGAATGAAAGAAATATCATGAATAACAATATCGGAGCTATAAAATCGATTCATCGTCAAGAATTTAATAGTATAACATAGGAAGATCTTTTATCCACACCAAATACATAATGAGATTCCTGATCCAATAAAAAACTATTTATTTATGATTCTTTTTCACCGCTTTCTTTTCTACAACCTAGTACTTTCCTTGTACAATCATCTGATGAAATATCATAAAAAACCTTTTCTACTTCGATTGTTTATAAAAAGAGTTTCTAAAGAACCTTAAATAAACAATAGAAATCAAATAGAGAAAACAAGTACGAAATTTCAAATTGAAATTTTCAAAATTTTGTTTTTGTAAGGGTCTATGATCTTTTTGTAAAACAAAGGAAATGTGATAAAGACGAGTCCCGATAAAAAAAAACTAAAATATTCCAAAAAATTAACTATATTAAATTTTCTTACGAGTTTTTCTTCGACATCGACTCTAATCTTTAAAAAGAGCATATTCATTAGGGAAGACTAATTTGATCTTTTTTTTTGAAACATCCTCTTTACTTGGTTGGATTCGAACTATTTTCACTTCCTTGACTTCATAGAAACAAAAGTATATATAGGTACTCTTGGCAAACGTATTATACGCTATCCTATTTGATTTTCCTACACGAGTTAATGGGAGATTAATTGACAAAAAGAGGAAACCCCATACAGTATCTCGTTCTTGAAGTGGTGAATGCTCTCAATAATTATAATTATACTAATTTACATATGTCTTTAAATTGGTGCTACAGAAATACCCCTTTCTTTTGCTTGATGAAAAAATAAAAATAAAACAAAAAGATAACCGAAACCATTTTGATCCCCTTGCCCAGAAACAAAAAGGGGAGTTTATGTCTTTTTTTTTTAATTGAATCCGCCGGGACTGACGGGGCTCGAACCCGCAGCTTCCGCCTTGACAGGGCGGTGCTCTGACCAATTGAACTACAATCCCATGGAAATAAAGCGGGTAGCTTACATATTCCTTCTTATGATTTCATTTGAATCATTTCAATTTTAGATTCAAATTAGTGTTTTGTAACAAAGAAAATCACAAGTAATATATTGATATCTATATGGATATCACTAAAGTGATATCAAGGCCGATTACTAGTAATCCTTGCATTATTCTAAAATCGATTGATAATCTATTTTTTATTGTCATTTTTTATGGAAACAAAAAGTAACGAGCCACAAGAAATAAAGAAAAAAGTAAAGTCGAAATATACCCAGATATTTGACTTTTTTCTTACCCTTCTCTGTCAATTATGCAAAACAAAAAAGGTTATGTAGACAGCGAATTATTGGGCCGAGCTGGATTTGAACCAGCGTAGACATATTGCCAACGAATTTACAGTCCGTCCCCATTAACCGCTCGGGCATCGACCCAGGAAGACTCTATTCGAACTTTATGGATAATCCATAATCAACTTCCTTTCGTAGTACCCTACCCCCAGGGGAAGTCGAATCCCCGCTGCCTCCTTGAAAGAGAGATGTCCTGAACCACTAGACGATGGGGGCATACTTGCTCAACCGCCATCATACTATGATCATAGTATGATCAGTTTTTTAAAATTGTCAATATAATCAAATGGTATGACTAGCTTATAAGATTTTTGATTTTTTTCTATAGCATTCTATATCATTTTTTTATTTTACATTTATATTCATATTCTAATCACAATTCTATAAAAAAAATCGATATATTTTCTTTTTATATTTGAAGTGGAAATATGAAAAAAAAAAAAAAATCGAAAAAAAGTCTAGTCTAAAATCTTTTAAAGAAGTGATTGGTCTGACAGAAAAAAAATAAAAAAGAGGGTTAAGTTTCGTTTTTTTTACTTTACTTCATAGATTGCCTCATCTCATTGTTAAGAAATAGTAGTGTCCCTATCTAACACTAACCCAAGAAAGTCAGACAGAATCCATCTTTCTTCCCTAATTAGACGATGGATTGATAAGTTAAGTTATCGATTCTCGCTTCTAGTTGCGAAATGAGCTACTAACCACTATGCGTCTATTGTATATATATTTAATATATATATATATTTGATTTACCTATCGACTCAGTCAGGAATTAAATCAAGACGGCCCTTTTAACTCAGTGGTAGAGTAACGCCATGGTAAGGCGTAAGTCATCGGTTCAAATCCGATAAGGGGCTTTTACTTTCTTTAACTTTCTATATAGGAAAAATTTCATTCGAAAGTCTATAATTTCGAATTTTTTGAATTTCATTCTAATGAATTTCATTTTAATAATAACTAATAATAAAGTGAGAGAGTAATTTAGAAAATCAAATTGAACATTTTTATATTATAATACAATGAATAATAATAAGTCGGCTTTTGAATCGCCAAATAGATATTCGTTGTTTCCCTTTTTCGATAGATTAGAAATCAACAAATCCAAAAGAAAAAGTAAGTGGACCTAACCCGTCGAATCATGACTATATCCACTATTCTGATATTCAAATTCGATAGAGATAAAATTGAAACAGTAGATTTGGTTTATTTCATATTTTTTTATTCGGAAATCTGTCGATATCTCTTATTTAATCTTCTTGTTTCTATATTTCATAGGAAATATATTGCGTTCCTGCCTAGAGAAAGAAAGTCTTATTCCAAATTTTTTAATACCTAAAGGGTATTTCAATATCTTGTTTTGATTCCAGAACATAACAAGAGCCTAAATTCTAGTTGTATAAGAATCAAATTGTATTAAGAATCAAAAAATCGAATCATAAAGAATGGCTTCAGATATCAATCAAATATTTCCATATTGATGCTTACAAGATGACAATGTAATGGGATTGAAGGTGTATGTGAGAAAGAAACTCTCATTTACAGTTTGCTATTATTTTATTTAAATATTGTATTGAATTAGATATAAATAATAAATTTTCCCTTTTTTTACCGGCATGGACATGTAGATATCAAATAAAATAGAAAAAAAGATTTCTTTATCTGAGTAATGAGTCATCTGACAATTCATGATTTAGATTCAACTACTTATTAAGAAACTAATAGCAAGGAAGAAACAATTTGAGTTGATGCGTTTACCTAAGTAAGGACCAATAAAATCAAATATTTTGATCTTCGAAACCAATTAAATGAAATTCTAAAGGTTAAATTTTATGGGGCAGTGCGCGAGAAATCAAATCATAAATAAATGATAGAATTTTGAGCGTCCTGAACATAATATATAACATTAAGATATATAAAGGTGTTCGGAAATGGTTGAAGTAGATGAATAGGAGGATCGCTATGACTATAGCCCTTGGTAAATTTACCAAAGACGAAAAAGATTTATTTGATATTATGGATGACTGGTTACGGAGGGACCGCTTCGTTTTTGTAGGTTGGTCTGGTCTATTGCTCTTTCCTTGTGCCTATTTCGCTTTGGGGGGTTGGTTCACAGGTACAACCTTTGTAACTTCATGGTATACTCATGGATTGGCTAGTTCCTATTTAGAAGGTTGCAATTTTTTAACCGCTGCAGTTTCTACTCCTGCTAATAGTTTAGCGCATTCTTTGTTGTTACTGTGGGGTCCTGAAGCACAAGGAGATTTTACTCGTTGGTGTCAATTAGGCGGTCTGTGGGCTTTTGTTGCTCTCCACGGTGCTTTCGCATTAATAGGTTTTATGTTACGTCAATTTGAACTTGCTCGATCTGTTCAATTGCGACCTTATAATGCAATCGCATTCTCTGGTCCAATTGCTGTTTTTGTTTCTGTCTTTCTAATTTATCCACTAGGTCAATCTGGTTGGTTCTTTGCGCCTAGTTTTGGTGTAGCGGCTATATTTCGATTCATCCTCTTTTTCCAAGGGTTTCATAATTGGACATTGAACCCATTTCATATGATGGGAGTCGCTGGTGTACTGGGCGCGGCTCTGTTATGCGCTATTCATGGTGCTACTGTAGAAAATACTTTATTTGAAGATGGTGATGGTGCAAATACATTCCGTGCTTTTAACCCAACTCAAGCCGAAGAAACTTATTCAATGGTCACCGCTAACCGCTTTTGGTCACAAATCTTTGGGGTTGCTTTTTCCAATAAACGTTGGTTACATTTCTTTATGTTATTTGTACCAGTAACTGGTTTATGGATGAGTGCTCTTGGAGTAGTCGGTCTAGCTTTGAACCTACGTGCCTATGACTTCGTTTCCCAGGAAATCCGTGCAGCGGAAGATCCGGAATTTGAGACTTTCTATACTAAAAATATTCTTTTAAACGAAGGTATTCGCGCTTGGATGGCGGCTCAAGATCAGCCTCATGAAAACCTTATATTCCCTGAGGAGGTTCTACCACGTGGAAACGCTCTTTAATGGAACTTTAGCTTTAGCTGGTCGTGACCAAGAAACCACTGGTTTCGCTTGGTGGGCCGGGAATGCCCGACTTATCAATTTATCTGGTAAACTATTGGGAGCTCATGTAGCCCATGCCGGATTAATCGTATTCTGGGCCGGAGCAATGAACTTATTTGAAGTGGCTCATTTTGTACCTGAAAAGCCCATGTATGAACAAGGATTGATTTTACTTCCCCACCTAGCCACTTTAGGCTGGGGGGTAGGTCCTGGGGGAGAAGTTATAGACACCTTTCCATACTTTGTATCTGGAGTACTTCACTTAATTTCTTCTGCAGTTTTGGGCTTTGGCGGTATTTATCATGCACTTCTGGGACCCGAAACTCTTGAAGAATCTTTTCCATTTTTCGGTTATGTATGGAAAGATAGAAATAAAATGACCACCATTTTGGGTATTCACTTAATTTTGTTAGGTGTAGGTGCTTTTCTTCTAGTATTCAAGGCTCTCTATTTTGGGGGCGTATATGATACCTGGGCTCCAGGAGGGGGGGATGTAAGAAAAATTACAAACTTGACTCTTAGCCCAAGTGTTATATTTGGTTATTTACTAAAATCTCCCTTTGGGGGAGAAGGATGGATTGTTAGTGTGGACGATTTGGAAGATATAATTGGAGGGCATGTATGGTTAGGTTCCATTTGTATATTTGGTGGAATCTGGCATATCTTAACCAAACCTTTTGCATGGGCTCGCCGCGCACTTGTATGGTCTGGGGAGGCTTACTTGTCTTATAGTTTAGCTGCTTTATCTGTTTGTGGTTTCATTGCTTGTTGTTTTGTCTGGTTTAATAATACTGCTTACCCTAGTGAGTTTTACGGACCTACAGGGCCAGAAGCTTCTCAAGCTCAAGCATTTACTTTTCTAGTTAGAGACCAACGTCTTGGAGCTAACGTGGGGTCTGCTCAAGGACCTACAGGTTTAGGTAAATACTTAATGCGTTCCCCGACTGGAGAAGTTATTTTTGGAGGAGAAACAATGCGTTTTTGGGATCTGCGTGCTCCCTGGTTAGAACCTTTAAGGGGTCCTAATGGTTTGGACTTAAGTAGGTTGAAAAAAGACATACAACCTTGGCAAGAACGACGTTCTGCAGAATATATGACTCATGCTCCTTTAGGTTCCTTAAATTCTGTAGGGGGCGTAGCTACTGAGATCAATGCAGTCAATTACGTCTCTCCGAGAAGTTGGTTATCTACCTCTCATTTTGTTCTAGGATTCTTCCTATTCGTGGGTCATTTATGGCACGCGGGAAGAGCTCGGGCAGCGGCAGCAGGATTTGAAAAAGGAATTGATCGTGATTTTGAACCTGTTCTTTCTATGACTCCTCTTAACTAAAGTAGTAGTTAAAATAGGAAAGTAAAAATCGGGTCATATTAAAAAGTCTTCTTTCTTTCTTTCAATTCAATCTCGTTTTTTCTGGCTCGGCTGGATAGTATAGCCGAGCCATTCTCCTTTTTTATGATGCTAAGAAGTAAAAAAAGCCAATAAAGAAAAAAATCTATTCATCCAACAAAAGGAGAGAGAGGGATTCGAACCCTCGATAGTTATTTTTTATGAACTATACCGGTTTTCAAGACCGGAGCCATCAACCACTCGGCCATCTCTCCAAAAGATAATTTCTATTTTATCTTTTTTTTTCGCCAAATAGAACATAGCTCGATGAGTTAATACGATCACTATGTAGAAAAAGATATAGGGTGTGACTTTCTTTATAAGTCTATCAATTGTATCTATATAAATGAGATACATGATCCAGTCTACCCATTTGTGAAGTAAAAAAGAACCTTTAACTTCATGTCCGAATAGAATAAAAGTGGTAAAAAGAAGTTGGAAATAAGGCATCTCGAATAAACGGATTCATGATAAAATCCCTTTATTTATTAAAATTTTTTAGTGGGTAAGAGGATTAAATGGTGTATATTGTTAATAGCTTGGAGGATTAAAAACATGACTATTGCTTTTCAATTGGCTGTTTTTGCATTAATTATTACTTCATCAATCTTACTGATTAGTGTACCCGTTGTATTTGCGTCTCCTGATGGGTGGTCGAGTAACAAAAATGTTGTATTTTCTGGTACATCTTTATGGATTGGATTAGTCTTCTTGGTGGGTATCCTTAATTCTCTTATCTCTTGAATTCATTCGTTGCAGATCAAAAAATGAGATGACCCCTCCCATTCCACGAATTACACATTCAAATTCAATATAAGTCCATAAAATGCAAATAAAGAAAACAAAAAAATTAGAGGGGGGGTCGAACTTCTGTAACTTGAGTGAAATATGAATCAAATATTAATAAATAGCAATTTACTAAATATAACTATGAAATAGTAATAACTAATTAAATAAAAAAAAAACGAATCAAAAATTGATATCTGATATCAATATAGAATATAATATTTTATGGAAATAGAGAATAATATATTATTGAATATGGAATTCTATATATAGATATAGAATAAATATATTATTAATATATAATAAATATATATATATTTATATATATTAATAGAATTGTTAATTGAACTTTTTTGGTAGTAGAGTTTTATCAAATGACCCCAAACCAAAGAGTGTATCTCGTATAGCTTTGAACAAATATTATCCATAAATTTCTTATCAAGAAGGCAAAAAAATGCGGATATAGTCGAATGGTAAAATTTCTCTTTGCCAAGGAGAAGACGCGGGTTCGATTCCCGCTATCCGCCCAAATAGAAATGGATTCAAAAAGATCAAAGATTCGGTATAGTTGACCGGGAAATATAGTAATTTTTGCCTCGCGTCCCAAAAGATAAGTATTAATTATAGTTAATAGAATCAAACTTACATTTGTTGAAAAAAAAATGTTGCGGAGACAGGATTTGAACCCGTGACCTCAAGGTTATGAGCCTTGCGAGCTACCAAACTGCTCTACCCCGCGATGAAACAAAAAAACTTGGACTAAACTCTAATAAACAAAGACGAATTGAATGCGCCCCTATTCCATATCTGTACAAATAGAATAGCCTATTTAGACAGAATGGTAAAGGGGCCTCGTCGAGCATAGAAAAAATAGAAAAATTAAAGGATACTTAAATCTTTACCAGCTTGATCTTGTTGCCCCTGGCAATAAACATGCCTGAACCATTTCCCGAAGGATGTGTCCAGATAGTCCAAAGTCTCGATAGTTAGCTCTCGGTCTTCCGGTCGAGAAGCAACGTCGATGAAGACGTGTAGGTGCACTATTACGCGGTGGGGATTGTAATTTTCCATGAATTTTCCACTTCTCACTTAGCGACGGAATCTCACTTATTTCCTTTTTTAAGGATCGACGAATCAAATGATATTTTTGTTCTAATTTTTGCCTCTTCTTCTCCCTATAAATCAAACTTTTCTTTGCCATAATGCTTAAGTTCCTCTTATTATCAATGATAATGATACAAATCGGATCCTAGATGTAGAAATAAATATAAGAGTGCATACCTATATTTTTATTATTTTAATAAAATTAATAAAAAAAAATATATTATTGCGGATAGAATAATTAAATAATTAACCGAATTTGCCCGACGTGGAGGCAATCAAGAAAGCCGCATAAGTGAATATATAACCTACAGAAAAGTGAGCTAATCCAACCAATCTTGCTTGCACAATTGAAAGAGCTACTGGTTTATCTTTCCATCGAATCAAATTTGCCAAAGGTGTACGTTCATGAGCCCATGCTAAAGTTTCAATCAATTCCTGCCAATAACCACGCCAGGAAATTAAGAACATAAATCCAGTAGCCCAAACAAGATGCCCAAATAAGAACATCCATGCCCAGACTGATAAACTATTCATACCAAACGGGTTATATCCATTGATAAGTTGTGAAGAGTTTAACCATAGATAATCTCTTAACCATCCCATCAAATAAGTGGAAGATTCATTAAACTGTGAAACGTTACCTTGCCATAATGTGATGTGTTTCCAATGCCAATAAAAAGTAACCCATCCAATAGTATTTAACATCCAAAAAACTGCCAAATAAAATGCGTCCCAAGCCGAAATATCACAAGTACCACCTCGTCCCGGACCATCGCAAGGAAAACTATACCCGAAATCCTTTTTATCTGGCATTAACTTGGAACCACGTGCATCTAAAGCACCTTTTACTAAGATCAATGTAGTTGTATGTAAACCTAAAGCAATAGCATGATGAACCAAGAAATCTCCAGGACCTATTGTTAAGAATAATGAATTACTATTCTCATTAATAGCATTTAACCAGCCGGGCAACCATATGCTTCGACCCGCATTAAATGCTGGGCCATTTGTCGAAGATAAAAGTACATCAAATCCATATGAAGTTTTCCCATGAGCGGATTGTATCCATTGGGCAAATATGGGTTCGATCAAGATTTGTTTTTCGGGAGTACCAAAAGCAAGCATGACGTCATTATGAACATAAAGTCCCAAAGTATGGAACCCTAGAAAGAGGCTGGCCCAACTTAAATGGGATATGATAGCTTCTTTATGGTCTAACATTCTTGCCAATACGTTATCCTCATTCTGTTCTGGATTGTAATCTCTAATAAAAAATATAGCTCCATGAGCAAAAGCTCCTGTCATGATGAACCCTGCAATGTATTGGTGATGGGTATATAACGCAGCTTGAGTCGTAAAATCTTGCGCTATGAACGCATAAGCAGGTAAAGAGTACATGTGTTGAGCTACCAAGGAAGTAATAACTCCTAAGGAGGCTAGAGCAAGGCCTAATTGAAAATGAATCGAATTATTGATTGTGTCATAAAGACCCTTATGCCCACGCCCCAACCGTCCTCCCGGAGGAATATGTGCTTCTAAAAGATCTTTTATACTGTGTCCGATTCCAAAGTTAGTTCTATACATATGACCCGCAATGAGGAAAAGAATTGCGATAGCTAGATGATGATGTGCCATATCGGTTAGCCATAAACTTTGCGTTTGTGGATGGAATCCCCCAAGAAGGGTTAGAATGGCAGTTCCTGATCCTTGGGAGGTACCAAATAAATGACTACTTGAATCGGGGTTTTGAGCATACAGATTCCACTGACCCGTAAAAAGTGGGCCTAACCCTTGGGGATGCGGTAATACACTTAAGAAATTATTCCATCGAACATATTCCCCCCTGGATGCAGGAATAGCGACATGTACTAAATGACCTGTCCAAGCCAAGGAGCTTACCCCGAATAGTCCTGACAAATGATGATTCAGACGAGATTCAGCATTTTTGAACCATGAAACTCTTGGTTTCCATTTTGGTTGTAGGTGTAACCAACCCCCTATTAAGGATAGGGCAGAAAGAAATAATAGAAAAAGAGCTCCAGTATAAAGATCTTCATTAGTACGTAAACCGATTGTATACCACCACTGATAAACACCAGAATAAGCTATATTCACCGGGCCAAGAGCACCTCCTCGAGTAAATGCTTCCACAGCCGGTTGACCAAAATGAGGATCCCAAATAGCATGAGCAATCGGTCTTACATGTAAAGGGTCTTGTATCCATGTCTCAAAATTTCCTTGCCAAGCTACATGAAACAAATTTCCGGAAGTCCACAGAAAAATTATTGCTAATTGCCCAAAATGAGAAGCAAAAATATTCTGATAAAGACGTTCTTCAGTAATATCATCATGACTCTCGAAGTCATGTGCGGTAGCAATACCAAACCAAATACGACGAGTAGTGGGGTCCTGAGCTAAGCCTTGGCTAAACCTTGGAAATCTTAATGCCATAATGCCTTTCAAATCCTCCTAGCCATTATCCTACTGCAATAATTCTTGCTAAGAAGAACGCCCATGTTGTGGCAATTCCACCCAGAAGGTAATGGGTTACTCCTACAGCACGTCCTTGTACAATGCTCAAGGCTCTAGGCTGAGTAGCAGGAGCAACTTTTAATTTATTATGAGCCCAAACAATGGATTCAATAAGTTCTTGCCAATAACCACGCCCGCTGAATAGAAACATTAAACTGAAAGCCCATACAAAATGAGCACCTAGGAAAAAAAGACCATATGCAGATAACGAAGAACCATAAGATTGAATTACCTGAGATGCTTGTGCCCATAAGAAATCGCGGAGCCACCCATTAATAGTAATGGAACTCTGTGCAAAGTTTCCTCCGGTAATATGAGTTACCACCCCTTGATCGCTTATACTACCCCAAACATCTGACTGCATTTTCCAACTGAAATGGAATATTACTACCGAAATAGAATTGTACATCCAGAATAGTCCTAAGAAGACATGATCCCAAGCAGATACTTGACACGTTCCTCCTCTTCCAGGCCCATCACAAGGGAAACGAAAACCAAGATTTGCTTTATCTGGTATTAACCGCGAGCTACGAGCAAATAAAACACCTTTCAACAGTATCAATACCGTCACATGAATTGTAAATGCATGAATATGATGTACCAAAAAGTCGGCCGTTCCTAATGGAATAGGTAGCAAAGCTACTTTGCCACCCACTGCTACTAACTCACCGCCCCCCCAAGTCAAACTGGTGCTCGCTGTTTCACCAGGGGCTGTTACACCAGGTGCTAAAGCATGGGTATTTTGTATCCATTGAGCAAAGACTGGTTGTAATTGTATAGCAGTATCTGAAAACATATCTTGTGGACGCCCTAAAGCACTCATGGTATCATTATGAATATACAAACCAAAACTGTGGAAGCCTAGAAATATACATACCCAGTTGAGGTGTGATATGATTGCATCGCGATGCCTCAGGACACGATCTAATAAATCGTTGTATCGATTAGTTGGATCATAGTCTCTTACCATAAAAATGGCTGCATGCGCAGCAGCACCAACTATGAGAAATCCACCAATCCACATGTGATGTGTGAACAATGATAGTTGTGTAGCATAGTCAGTAGCTAGATATGGATAAGGGGGCATGGAATACATATGGTGAGCTACAACAATAGTTAAAGAGCCTAACATAGCCAGGTTAAGAGATAATTGAGCATGCCATGATGTTGTTAGAATTTCATATAAACCTTTATGGCCTTGGCCTGTAAATGGACCTTTATGAGCCTCTAAAATATCTTTTAGACCATGACCAATACCCCAGTTGGTCCTATACATATGACCTGCTATTAGGAAAAGAATTGCGATAGCTAAATGATGATGTGCTGTATCGGTTAACCATAGACCCCCGGTCACTGGATCTAATCCACCACGAAAAGTAAGAAAGTCTGAGTATTTTGACCAATTCAAGGTAAAAAAGGGGGTTGCTCCTTCAGCAAAACTTGGATAAAGTTGAGCCAAAAGATCCCGATTCAAGATAAATTCATGAGGAAGTGGTATTTCTTTAGGATCTACTCCAGCATTTAGAAATTGGTTAATCGGTAAAGATACATGTACTTGATGTCCTGCCCACGAAAGGGACCCAAGTCCTAGTAGCCCTGCTAAATGGTGATTCAACATAGATTCTACATCTTGGAACCAAGCCAATTTTGGAGCTGCTTTGTGATAATGGAACCAACCAGCAAAAAGCATTAAGGCTGCGAAGACCAATGCGCCAATTGCGGTACAATAAAGTTGTAATTCACTAGTTATTCCAGATGCTCGCCAAAGCTGAAAAAAGCCAGAGGTTATTTGTATTCCTCGGAAGCCTCCGCCCACATCTCCATTCAGGATTTCTTGGCCCACTATTGGCCAAACCACCTGAGCACTAGGTCCAATGTGAGTAGGATCACTCAGCCATGCTTCATAATTGGAAAAACGAGCACCGTGGAAATACATGCCACTCAGCCAAAGAAAGATGATAGAGAGTTGGCCAAAATGGGCACTAAATACTTTTCTAGAGATTTCCTCCAAATCACTGGTATGACTATCAAAATCGTGAGCATCAGCATGTAGGTTCCAGATCCAAGTGGTAGTATCAGGTCCCTTAGCTATTGTTCTTGAGAAATGACCGGGTTTAGCCCATTCCTCGAAAGAAGTTTTTATGGGATCCCTATCTACCAAAATTTTGACTTCTGGTTCCGGCGAACGAATAATCATTGAGTCCTCCTCTTTCCGGACAACACATACAAAGAAACCCGCCAACAGTCACTCAAATAATTAGTGAACCGATGATAGATGCTTAGAATTTTGTTCTTTCTCTTCTATCTCCCATCTATTCATCCATTTTCTTTAGTTATTCACTAGAGCAATTATGATCTGGAAGTCGATCTGGGGCAAGTGTTCGGATCTATTATGACATATCCATAGGGTGCTCAACGGACCCCCCCCCCCCTTTTTTTTTTATTAAAAAGCGTTTTCGCACCTTTACATTAGTATTGGTACACAAATAATTTTTTTTTATAACCTAATCTAGTGTATTCATATTTCAATTATAAGTTCCGAAATATAGCCTATTTTTTATGTTTTAAATAGAGGATATTATCCTATTTCAATAACCGCTTATTAGTCATTACTAAGAAACATTCTAGTATTGATATTTAGTCATTTTCAAATCCCTTTTATTCGTTTTAATAGTCGAAAAGAAAAAAATAGAAAAAACTAGATATAGATATTATAGATATTCTCATATTCATGTACTACTTATCCCTAGAGAATACCAGATTAAATAGAACGATTTGAGAAAAGGATATAATGAAATTTTTTTCTGGGATTGGTTCTTCTGATAGAAAAAAGAATCTGGTTTATTTGACCGAGAGGGCCAAGAAACTAAAAAACAATTAATTGTAAAAACAAATAAAGATATATTATAGAATAAAAAAAAAAGAAACAAAGAAAAAGTTCTTATTCGAAGCGCCTCGTGATCGTCAACCAATTCTGTGCTTCAATATAATTACCAGGAGTAAGCGTTATAGCCTGTTTCCAATACTCGGCGGCTTGAGCAAACCAAGCCTCCGCCATTTCAGAATCTCCTTGTTGAATGGCCTGTTCTCCACGGTCGGAATAGGCGGGTCAATTCCCTCCCTGAGAACCGTACTTGAGAGTTTCCTACCTCATACGGCTCGACAACCAACTCTTTTGTTTTGGTGTACCAGTTTTTTTCACTTTAACCTACTTTCACTTTATATCTAATTGAATGAGATTTCTTATAGATATTCATTCGGTTTTTCTTGGATTAAACAAAAGAGAGTAATTACATGAGTTTCAAACTTTCGTTTTGATTTAATTAATATATTAATTAATCTAATAATAAGTTTTATCTTTTCTCCTACCTTCAGAAAAAAAAAGGCATGTCCACTGTTATTAGATATTAGAATTTTCTGAAAGGTAACTATCCCGCTTTCATATATAAATTTATATAGAATCGTTGAAAAAGACTTTTTTTCATACTTCATAAAAAAGAAAAAGACTTACTGTCTTTAGGATCTGATGCTACACCGCTGCTCAATACCTTAGGGGATCCACTCTATTACATAAGTAGATTCCTAAGATTTATCTCATATTATGATATAAATAAACAGCTCTTGTTGTATCGGTCCAAAACCTTTCCAGTTGATCTTTACGGTGCTTCCTCTATCAATTAAATCTTTTTTTATCCATAGAAATAAAGTATTTAGGCATATCTAGTCTTCACTTCATATTTCGATCCATGAAGTTTATTTATTTGCTACAGCTGATAAAAAATCGTTTTGGACGATGCTTATGTAGAAAGCCTTTTTTTTTTCTAGTATTTCATTGACTAGCTGTTCGTTCTTTTTTTCTATAGTGGAGATAGTCGCACGTAATGACAGATCACAGCCATATTATTAAAAGCTTGTGGTAAAAAGGGGTTTCGTTCTAATGCCCGAAAATAATATTCTAAAGCTTTGGTATGTTCCCCATTACTTGTGTGGATAAGGCCTATATTATAGAGTATATAACTTCGATCATAGGGGTCAATTTCTAGTCGCATAGCTTCATAATAATTCTGTAATGCTTCCGCATAATTTCCTTCAGATTGAGCCGACATCCGTTACGGTCGTCATTCGCTTTAACGAATTCTCCGTTTCAGAACCGTATGTGAGATTTTCATCTCATACGGCTCCTCCTTTAGGTGCATAATGAAAATAATAAATATATGGATATGGAAAAATTTGATGTCATTATGAACTAAGCGGGGCTAATGTTTTTACAAGAAATCCCTAGCCAACCTTCTTGTAAAAGATCTTTTCTTACTACCAAGTGGATTCATATTCATACTAGATAAAAATAAAAAAGGAAACTCTAACAATTTCTTTGTTCTCAACGCCCCTAAATTTCCAGGAATTAGTCACTTCAACAGTCTTCAATGGTTATACGGGTATCCAAAGTACGGACGAGATGGATGTTTATTGTTCCAACCATTTTAATTAGTCCCAATCCCAAAGAAGAAGAAAGAAAAGGAATCTTTTTGAAGAAAGTTTTCGTGTTGTTGATTTCTCGGCGTAGTGCTTCTTCCCCTGTGCCTCCTATTCGTATATTGTATTAGTCTAGTAGGATTGATCTGTAATACGGGAACCATAGGTAAAAACCTTTTGCTCAATACTAGAATTCATAATTGAAGCATCTAAGGCTGCACTAATCGTGGATACATGACAGAAGGGATTGCTTTTTTATATTATAAACTTCACCTTCAAAAGCGTAGATTTTTTTCAATACTCATTTTTTTCTATTCCAAATCGGTGAGAAATAGAAAAAAATGATAATGATAATCAAATCGCACCATCTCTGTAATAAGTAAATGCCTCTTTTTCTCCGGAAGTTGTCGGAATGACTCGTAATAAGATATCGGCTACAATTGTAAAGGTTTTATCAATAAAATTTCCATTTATACGCGATCTTGGCATAGGTAGTAATCCATTCTATAACTCTTTTTATTTCCTTTAACTTTTCTTTTGTGAGAAAATTTTCTCACAAACAAAGGAATTTTATAGTACGAACTAACATAAAAGCGGACTCGTTTTTTATAAAAAAATATTCTATCTACTTCCAATTTTTCCGATCAAAAAAGGTATCTATTAACCATAATCTAAAAAACGATGAATAACTCGCTATTCACCCAGGTACTCAGTCATAATCCTGATGTCGGAGAGATGGCCGAGTGGTTGAAGGCGTAACATTGGAACTGTTATGTAGACTTTTGTTTACCGAGGGTTCGAATCCCTCTCTTTCCGTACTTTCAACTAAATAACCAATCTTACGTGATTGACCACAACGTATCAAATCAAATAAAAAAAATAGATAAAAAATCTACATTTCTTTGCTATGGAAAATGCTGGGAAGAGCAAACAAGGGATCCAAACCTCCCTACCAATCTATGATACATGAATAGGAAAAAGATTCCCCGACAAAATCCCTTACCTTGTCCCTTTTTAGTTTTAATCAAAAAAGCGGGCAAAGGGGAGTTGTCCGAACTCTTGTTTTTAGTGATTTTTTTTTACTTCACTTAGGTTTTTTAAGTCTGGCGAGAGTAATATTCTACGACAAGCAATTCATTTATTTTCAAACCGACGCATTTCCTATCTATTATTTGATTGACTAACCCTTCATATTGGAATGTGTGAAGAGTCAGATGGTTTGGCAATTCCTCGGGGGCAGATGACTCAAGAAGATTTTGAACCAAAGTTCTAGAGTTTTGTTCATCCTTCACTGTAATAATATCTCGGGGTTTGCATCGATAACTTGGTATATCAACTATACGACCATTAACTAAAATATGCCCATGGTTAACTAATTGGCGCGCTTGAGGAATAGTCAAAGCCATACCCAACCGAAAAAGGATGTTATCCAAACGCATTTCAAGTAATTGTAATAAAACTTGACCCGTTGACCCCTTGGCTTTTCCGGCGATACGAACATATTTAAGTAATTGGCGTTCTGTAAGACCATAATGAAAACGCAATTTTTGTTTTTCTTCTAAACGAATACGATATTGAGATTTTTTTCCGGAGCGTGATTGGTTTCTAAGATCGCTTCCTGCCCTAGGCCTTTTACTAGTTAGTCCCGGTAAAGCCCCCAGACGGCGTATTTTTTTAAAACGAGGCCCTCGGTAACGTGACATAAAGACTCCTTTTTTTATTGAAATTGTACAAAAACTAAACAAAATTAAAACTGAACTAAATGATAATGATAAATAACGTAAAATCCACTCCAATAGTTTATTGGAATACAAAGAGTCAGAAGATATATTCTCTCAATATACAGATTTTTTTTATTGTATATACAATATATATAAATCAATAAATCACAAAAATTTTCCTTTATTTTCTTCATTTATTTTGCCAAGATCTAACCCTTTTACCCCAATATATATTCCTATATGGAAGTTTATATGACATAATATAAATGGCGTGGTAACTCTTGGAAAAAGGTGAAAGAAGTCTTTTCAATCTTATTTTTTTTGAAAGTACATTAAAAATCATGTAAAAAAAATGAAAAACTATGTAAAAGCCGGCTATCGGAATCGAACCGATGACCATCGCATTACAAATGCGATGCTCTAACCTCTGAGCTAAGCGGGCTCAACTAAAATAGTGTATACAAATTCACTAAACTACTAGATCGTATTAATTAACTATTCTATTCATATTTTTCCTTATCTATTTAGAATTCATCATATTTTCGATATTCTAGAACAGAATATAGCTCAAATAAATAGTGACTATCATTAAATAAATAAAACAAAACCTTAATGAATTAATATAATATAGCAATATATCGACTTTCTAATTTTGATTTCATGAGTTTCTAAATAAGAAAATTTTAATTAGACCGGAAAGCTTTTTTTTTAAGTTAAATGATATCTGATTTGAAATTCTTGGTTTTTTTGTTCTAACCTCATGCAATTATTATTATTTGATACTTTTTCTCTTTTTATATTCTTTATTATTTTATAGAATTATTAGAATGAATATTCGAATATTCATTTCGAATATAATTTTTTAGAATTATTCGAATTTCAAATCTACGAAGTAGACTTATAATCTTTTTCCATTGCACATTCTAGAATTCTAAGTTTCAATAATGATCATAAATTTCTTTTCATGGAAGTAAAAAAAACGAATCGACCGTTCGACTATTTCTTAAAATTGAAGACAACGATGAGAAAAGGAAGAACATATATATGTTCTCTAATATATAACCATATTGAATTGCAAATACAAAAATGATAGAATCTTTGTTGATTAAACTAAATCAATATGGATGGGGCTAAAAAAAATGCAAGAAGATACCAAAGAAATAAAATAAGTATCTGTATGTAATGAATTCCAAGGTTTCGTCATAAGAAAAAGTGGAAAGACATCATAATGAGATCCTAATCTCAAAGCAAAAAGGGGGATATGGCGGAATTGGTAGACGCTACGGACTTAATTGGATTGAGCCTTGGTATGGAAACCTACTAAGTGATAACTTTCAAATTCAGAGAAACCCTGGAATTAACAATGGGCAATCCTGAGCCAAATCCTGGGTTACGCGAACAAACCAGAGTTTAGAAAGCGGGATAGGTGCAGAGACTCAATGGAAGCTGTTCTAACAAATGGAGTTCAATCCCTTGTGTTGAATCAAACGATTCACTTCATAGTCTGATAGATCCTTGGTGGAACTTATTAATCGGACGAGAATAAAGATAGAGTCCCATTCTACATGTCAATACTGACAACAATGAAATTTATAGTAAGATGAAAATCCGTTGACTTTTAAAATCGTGAGGGTTCAAGTCCCTCTATCCCCAACCCTACTCCCTAAAAAAGTCTGTTTGACACCTTACCCTTTTTTTAGTTATTCAAGAATTCATTGATCTTTTTTCATTCATCCGACACTTTTACAAACTCGAATTTCTTTTCTTATTATATACAAGTCTTGTGGGATATATCATACATATACAAATGAGAAAGAACTATCGATTTGAATTATTTCGAATCTAAATAATTTTTCATTCTAAAACTTAGAAAGTCTTCTTTTCGAAGATCCAATAAATTCCCGGTCCAAAACTTTTTTCATTTACTACTTTTGCGTTTCTTTTAATTGACATAGACCTAAGTCATCTCATAAAATGAGAATGATACTTCGGTAATGGCCGGGATAGCTCAGTTGGTAGAGCAGAGGACTGAAAATCCTCGTGTCACCAGTTCAAATCTGGTTCTTGGCATAGGGCAGAGGACTGAAAATCCTCGTGCCACCAGTTCAAATCTGGTTCTTGGCATAGGATTGATTAATTTTGATAAGTTTATAGTCTTCAAATTAAACGTATCTTTAGTAAAAAAAGTGCAATAATCCTTTATCCCCCTCTCTTTTTTGTTCATGTTGTGGATCCATCCGTTCAAAAAAAATGTATAAGACTTTATACCTAATACATATTCGAAAGGAAAGTTCTGGTTGAAAGAATAAAAAAAAGTAAAAAAAAAGATCTATATCTATCTATCTATATCTATCTATAGTATCTATCGTTGAAGGGCAGAAATACCCCCAAGATTCATTAGATTAGATACAATAGAAATAGAATTTTAACCCCCCCCCATTTATTGTATTGCTTTCCAATCTTATTTATTCATTCCCAGTTATGTGACTAAAGTTGACTAAGTTATGTGCGCGATACAAAGTTCATAATGCAGAACTCTTTTTTTTTTTTTTAGTTCATCCTATTGGCTCGGCTTTTAGGAAAAAAGTATCTTTCAAATTGGAGATTAAGCTATCTATAATAATATGAATAAGACCTTAATTCTTCTGTTTGTTTGATCTAAAAACGACTCGAATTCGAAATATTCCGCGAAGGTCCGTAGTTGTAGAAACTAAGACTCATTTTTATCATTCAAATTTTTTATCATTCAATAAGCATCTTGTATTTCATAAAAATTGGGGGCAATATAATCCTTACGTAAAGGCCACCCTATCCAACTTTCGGGCATTAAGATCCGTTTCAGCCGCGGATGGCTATCATAAGTGATTCCTAACATATCATAAGATTCCCGTTCTTGAAAATCCGTACTTTTCCAAACCCAGAAAACAGATGGAATTCTGGGATTACTCCTGTGAGTAAATACTTTTATGCAAACTTCTTCCGCTTGATTGACACCATATTCTATTCTCGTAAGATGATACACACTGGCTAAGAGGCCACCTGGTGCCACATCATAGGCACATTGGGAACGTAAATAATTGTAACCATATACATATAAAATTACAGCAATAGAATGCCAATCTTCGGGCTTTATTTGTAAAGTCTCTATTCCTTGGTAATCGAAGCCCAACGATCTATGAACCAGCCCGCGTTTGGCTAGCCAAACGGACAAAGTGCCCTGCATCTTTTTTATTTCCCCCACACCTTTTTTATATAAATTTAAGTATTTCACATTTACCATGAGTTCTAATTTATGAAGATTTTTTTCTTATTCTCTCAAATCCTCCCTAATTCACTAATTCGTGGGAAGATACTGGGCTTTTGTATTTAAAAAATGTTTCAGTAGAGATCTCTGAAGTAGATGATGGTGGATAGAGTAATTCTTGATCATAATTTCCAGTCTGTGTACTGCGTACAACAAAAAACTTGTGATTGGTAGTAAAACACCGATTACCCCGTTGAGGTCTAATTCGATCCTTATAGATTTCTCTAGCTATTTTCTTACGAAGCTTTGTTATAGCGTCTATAACAGCCTCTGGTTTAGGTGGACAACCCGGCAAATAGACATCTACAGGAATTAGCTTATCAACCCCTCGAACAGTACTATAAGAATCGGTACTGAACATCCCCCCTGTAATTGTACACGCTCCCATAGCAATAACATACTTTGGTTCAGGCATTTGTTCATATAATCTCACTAAAGAAGGAGCCATTTTCATTGTTACTGTACCTGCTGTTAAAATAAGGTCCGCCTGTCTAGGACTTGATCTTGGTACTAGCCCATAACGATCAAAGTCAAATCGGGAGCCTATTAATGAGGCAAATTCAATAAAACAACAACTGGTACCATAAAGAAGCGGCCATAGGCTGGAAAGTCTTGACCAATTTGAAAGATCATTTAACGTAGTTGAAATAACTGAGTTTTTTGTTGTTCGATCAAGTACGGGAAACTTAATGGAATTCATAATTGTTTCAATGGTTTTTTTTTACTTTTTTTTGATTGTTATTGTACAAGTATTCAGGAAACGAACTAAGACCATTCCAACGCTCCTTTTCGCCATGCATAAACTAAACCAAGAATTAGGATAAGCACGAAAATGAAAGCTTCTATAAAAGCGGATACCCCTAGTACATCGAAACTCATTGCCCACGGATACAGAAAAACGGTTTCAACATCAAAAACAACAAAAACTAGAGCAAACATATAATAACGGATTCTAAATTGTAACCAAGCATCCCCGATCGGTTCTATACCTGATTCATAACTAGAAAGTTTCTCCGGCCCCTTCGTAATTGGAGATAAAACCCCGGAAATTAGAAATGCCAAAACAGGAATAGCACTTGATATTATTAAAAATGCCCAGAAAATATCATATTCGTAAAGCAGAAACATAGACGAACTCCTATGAATGTGGAAAAAATACCCGCTTAGTCAATTCCAATCGGAGTGGATTGGGCAAGGTATATATAACTCTTGCGTCAAAACAAAAATTCAGGTTAATCGAATCATTTATTTTCGTTTGGTTGCTGTGGTAGACGTCTCCTTTTAAGATTTATTGATTGTAATCTTATTTTCAGTACACTTATTACTTAATATTTCCATGTTTCTATTACTAATAGTTTCTCATATTAATAATATAATATTAATATGATTAATAACTAGTAATTTTTTTTATTTCTGTTTCTTAAATTTGCTTTATGTTTTATTTAAAAATAAAACAAATTGATAAAAATATCTTCGTTTTTAAAATTATGACGTATCAAAAAATCCACTTACGACTATGAAAATGAATGAATAAAAAACGTTTATTCTAAATTATAAGTATCTATCTAGATATATCGATAGATAGTGATTGGATCCACTGAAATCAAATTTGGTTTTCCGTTTTATTCTGAACGACCCCCAGGACTTATGGTTTAGGGTCTGGGAGTTTTTTTTATGAACCAACAAATTGAAAGTAACCAGTTAGAAATAAAGAATACAATAATAAGTCAAAAATTATCCAATTATTTGGATTTGAATGTCATTTATTAGAATAAATTTATTAGTTAGGGCTATACGGATTCGAACCGTAGACCTGCTCGGTAAAAGAGCTCGAACTTATTATTATCAAAATGATTCGAACTCTTTCAAAGACCCAACATGCATTTTTTTTTGCATTGGGCTCTTTCATTAACTGATAGAAAGATCAGTTAGTCTACCATATTTTTTCTTAAAAAAAAAAGATAAGAAATGGTTCCAAGTACTCTGATTGATTATTTTTTAATTCTATAATTCTAATACAATACAGAATAACTACCAAAGTGTTTCAAAGAAGGGTTCTCTTGACGTAGGTTTGCTTTTGGTCTAGATCAACTTAAGTTAAATATAGTCTCTAACATCCTGATTAAAAAATCAAATATGAAACTTGCTACACCTTAAGGTTCATAGGACGAAAAGATCATTTTTGAGTTCCTTATACTCATTCTGCCTAGCATTAAGTAGACTGGGTATTCACCCTATCAATATCTCAAATCAATGATGGGTTCTATTAATTCCCTACCGAAATGGGGTACTTTAATAGGACCTAATGTCAGGCTATTGTTCTCCTCTTTTTCCTAAAAAAAAGTCATGGAGTAAGACATCGATTTATTAATAAGATCAATCAATTGGTTTGATTGCGTGATGGACTCCTCTGAAAAACTTTGGCGCACGTGTAAACGAGGTGCTCTACCTAACTGAGCTATAGCCCTTGTGTTTATGATCCACATTTTATCTTATCATGTAGATAATTTCTTGTCAAGATTAATATTATATGATCGAACATTATATCTCTTTCATCTCGTTGTTTATTGGTATTGCTTAGAAATAATATTGGATTTATAATCCTATCGATGTGATAAGTATCCCCGTGCCTTCTCTTTACGATGATAAATAACCTACTTAACTCAGTGGTTAGAGTATTGCTTTCATACGGCAGGAGTCATTGGTTCAAATCCAATAGTAGGTATAACTTATTAGACACCATGATCAATGGTGTCTAATAAGTTTTTGTAGCCAGCTTTTTTTTTTTTTTTTTCTCGCTTTTGGATCCTATTTTTTTATACGTCAGCTAGTTACAAAATCAAATCGTATTGAGAGCCTCGACGCGTGTCCGAGCTCGTCTGAGAGCTAGATTTGCCTCAATTGTTTGTCTCTTGCCTTCAGCTTTTCTCAAGTTCGCCTCTGCTATTTCAAGAGTTTGCTGAGCTTCTTGTGGATCAATGTCACTATTCTTCTCTGCATCATTTACTAAAATAGTAATTTCATTATTGCCTATTCTAGCAAAACCGCCCATCAGAGCCATTGTTAACCATTGGTTATTAAGGCGTATTTTCAAAATACCTATATCAACAGCTGTGGCAATCGGCGCGTGATTTGGTAATACGCCAATTTGTCCACTATTAGTAGATAAAATGATTTCTTTTACTTCTGAATCCCAAACAATTCGATTCGGAGTCAGTACACAAAGATTTAAGGTCATTTCTTCAATTTACTCTCCATTTCTAAGTTCGTAGCCTTCGCAGTAGCTTCATCGATGTTACCCACTAAGTAAAAGGCCTGTTCAGGAAGAGAATCAAATTCTCCGGAAAGGATCAAATTAAACCCTCTAATTGTTTCCGCTAGCCCAACATATTTTCCCGGAGAACCTGTAAATACTTCTGCTACGAAAAAAGGTTGTGATAAGAAACGCTCAATCTTTCGTGCTCTTGCGACGGTTAAGCGATCCTCTTCGGATAATTCGTCCAACCCCAGGATAGCTATAATGTCCTGAAGCTCCTTGTAACGTTGTAAAGTTTGCTTTACTTGTTGCGCAGTTTCATAATGTTCCTCGCCAACGATTCGAGGTTGTAGCATAGTTGACGTTGAATCTAAAGGATCTACCGCTGGATAGATACCTTTAGCAGCTAATCCTCTTGATAGTACGGTAGTCGCATCTAAATGTGCAAATGTGGTGGCAGGAGCAGGGTCAGTCAAATCGTCTGCAGGTACATAAACTGCTTGAATAGAGGTTATGGACCCTTTTTTCGTAGAAGTAATTCTTTCTTGTAAAGAACCCATTTCGGTACTAAGGGTGGGTTGGTAACCCACAGCAGAAGGCATTCTACCCAATAAAGCGGATACCTCGGATCCTGCTTGTACAAAACGGAAGATATTGTCGATAAATAGAAGTACGTCTTGCTCATTAACATCTCGGAAATATTCTGCCATAGTTAAGGCAGTCAGACCAACTCTCATACGAGCTCCCGGCGGTTCATTCATCTGACCGTAGACTAGGGCTACTTTGGAGTCCGCAAGGTTTAGTTCATTAATGACTCCAGATTCTTTCATTTCCATGTAAAGATCATTTCCTTCACGAGTTCGTTCGCCTACTCCACCAAATACGGATACACCACCATGAGCTTTGGCAATGTTGTTGATCAATTCCATAATTAGTACTGTTTTACCCACGCCAGCCCCACCGAATAGTCCGATTTTTCCCCCACGACGATAAGGGGCCAAAAGATCTACTACTTTAATTCCTGTTTCAAAAATAGATAAGGTTGTATCTAAGTCTATAAAAGCAGGCGCGGATTTATGGATAGGAGATGTTGTGAGAGTATCGACAGGACCTAAATTATCAACAGGTTCCCCAAGTACATTGAAAATTCGTCCTAGAGTCGCTCCGCCGACTGGAACACTTAGAGGATTTCCCATATCAACCACGTCCATCCCTCTCTTTAAACCCTCGGTCGCGCTCATAGCTACAGCTCTAACTCGGTTGTTTCCTAATAATTGCTGTACTTCACAAGTCACATTAATTTCTTGACCAAGCGTATCTCGACCCTTAACCACCAGAGCATTGTAAATATTAGGCATCTTGCCCGGGGGAAAGGCTACATCCAGTACCGGACCAATGATTTGGGCAATACGTCCCAGGTTGTTTTTTTCACGTATTGAAACCGCTGGATCCGAAGTAGTAGGATTTATTCTCATAATAAAAAATATGTTCAATTTTGTTGCGAAATTTTTCGAATACAGAAAAAATCTTCGTATAGTAAATTCATTGGTTAATTCAATAATAAATGGGAGTAAGCACTCGATTTCATTGGTACCACCCAAGCGAATATGCAATTCAATTTTTTACTTAATTAAATTTCAATGAAGGAATAGTCGTTTTCAAGCTCAACTAACCAAAACCTAGTTTTAAAATAAAAAATATATGAATAAAAAAAATTTTTGTGGAAAGTCTTTGATTTATTTGTCATAATAGGCAAGACTTTGTTTTATCTAGCCAATTCCGAAATGGAACTCTATTTATGATTCATTATTTCGATCTCATTAGCTTTTTTTTTTTCATATTTTCATTTTAGCATATCCGGTTATGCGTCCCATCGATATCAACCCCCCCCTTGTTTTTCATTTTCATGGATGAATTCCGCATATTGTCATATCTAGGATTTACATATACAACATATATTACTGTCAAGAGTGATTTTATTATTATTTTAATATTAAATATTTCGATTTATAAAAAGTCAAAGATTCAAAACTGGAAAAACAAGTATTAGGTTGCGCTATACATATGAAAGAATATACAATAATGATGTATTTGGCGAATCAAATATCATGGTCTAATAAAGAATCATTCTGATTAGTTGATAATTTTGTGAAAGATTCCTGTGAAAAAGGTTAATTAAATCTATTCCTAATTTATGTCGAGTAGACCTTGTTGTTTTGTTTTATTGCAAGAATTCTAAATTCATGACTTGTAGGGAGGGACTTATGTCACCACAAACAGAGACTAAAGCAAGTGTTGGATTCAAAGCTGGTGTTAAAGAGTATAAATTAAATTATTATACTCCTGAATATGAAACCAAGGATACTGATATCTTGGCAGCATTCCGAGTAACTCCTCAACCCGGAGTTCCACCTGAAGAAGCAGGGGCTGCGGTAGCTGCTGAATCTTCTACTGGTACATGGACAACTGTGTGGACCGATGGGCTTACCAGCCTTGACCGTTACAAAGGACGATGCTACCACATCGAGCCCGTTCCAGGAGAAGAAACTCAATTTATTGCGTATGTAGCTTACCCATTAGACCTTTTTGAAGAAGGGTCTGTTACTAACATGTTTACCTCAATTGTGGGTAACGTATTTGGGTTCAAAGCCCTGGCTGCTCTACGTCTAGAGGATCTGCGAATCCCTCCGGCTTATACTAAAACTTTCCAGGGACCACCTCATGGTATCCAAGTTGAAAGAGATAAATTGAACAAGTATGGACGTCCCCTATTAGGATGTACTATTAAACCTAAGTTGGGGTTATCCGCGAAGAACTATGGTAGAGCAGTTTATGAATGTCTACGTGGTGGACTTGATTTTACCAAAGATGATGAGAATGTGAACTCTCAACCATTTATGCGTTGGAGAGACCGTTTCTTATTTTGTGCCGAAGCTATTTATAAATCACAGGCTGAAACAGGTGAAATCAAAGGACATTATTTGAATGCTACTGCGGGTACATGCGAAGAAATGATGAAAAGAGCTATATTTGCCAGAGAATTGGGAGTTCCTATCGTAATGCATGACTACTTAACAGGGGGATTCACCGCAAATACTAGTTTGGCTCATTATTGCCGAGATAATGGCCTACTTCTTCACATCCACCGTGCAATGCACGCTGTTATTGATAGACAGAAGAATCATGGTATGCACTTCCGTGTACTAGCTAAAGCTTTACGTCTATCGGGTGGAGATCATGTTCACGCGGGTACAGTAGTAGGTAAACTTGAAGGAGACAGGGAGTCAACTTTGGGCTTTGTTGATTTACTGCGCGATGATTATGTTGAAAAAGACCGAAGTCGTGGTATCTTTTTCACTCAAGATTGGGTCTCACTACCAGGTGTTCTACCTGTGGCTTCAGGGGGTATTCACGTTTGGCATATGCCTGCTTTGACCGAGATCTTTGGAGATGATTCCGTACTACAATTTGGTGGCGGAACTTTAGGCCACCCTTGGGGAAATGCACCGGGTGCCGTAGCTAACCGAGTAGCTCTAGAAGCATGTGTACAAGCTCGTAATGAGGGACGTGATCTTGCAGTCGAGGGTAATGAAATTATCCGTGAGGCTTGCAAATGGAGTCCTGAACTAGCTGCTGCTTGTGAAGTATGGAAGGAGATCACATTTAACTTCCCAACCATCGATAAATTAGATGGCCAAGACTAGAAATTAGATTAGTAATTCACGTCCGTTTTATTAGTTTAATTGCAATTAAACTCGGCTCAATCTTTTTTTTACTAAAAGGATTGAGCCGAGTTTATCTAGTGTATATACTGTTTTTGATAGATACATACTTAATCTAGATATACAAAATCTGAAAAAAAAAGAAGATTAAACACAACTACACTTTTGTATTGTAGTGTCCACAAGAAATTCTATACGAAATATGGATTCTTAGGATTTTTTTATTCTTTTTTTAAGTTTCGTGTCAGGGCTTGAACCAAGTATCCCCACTTCTTCTACCCATTCTGCATGTTGTCCTTTTCTTTTCATTCCGTATTGGAATAAAAACCTTTTTTTTATATTAGTATACGAGATTTTACTAAAAAAGTTCTTCATATCGTTATATTCATAAGCGAAGAACAAATATTTCTTTTTTTTAATGAGAATTTTACACAATATAAGAAAATCCTTATTTTCATTTAGAATTGAAATTTATTAATTTCAATTGCTTTTACTTAATAATCTTAGCAATTAGCAATTGCATTGACATGCTTTGCTTACTCTGAATAGAAAATGAACTATTCAAATTTTTTTTTTTGCATTTTTCAATTTTTTCATTGAATGACTATTCATCTATTGTTATTTTATTTTCATGTAAATAGAGGCCAGAAGCTCTATGGAAAAATCGTGGTTCAATTTGATGTTTTCTAAGGGAGAATTGGAATACAGAGGCGAGCTAAGTAAAGCAATGGATAGTTTTGCTCCTATTGAAAAGACTACTATAAGTAAAGACCGGTTTATATATGATATGGATAAAAACTTTTATGGTTGGGGTGAGCGTTCTAGTTATTACAATAATGTTGATCTTTTAGTTAACTCCAAGGACATTCGGAATTTCATATCGGATGACACCTTTTTTGTTAGGGATAGTAATAAAAATAGTTATTCTATATATTTTGATATAAAAAAGAAAAAATTTGAGATTAACAATGATTTGAGTGACCTAGAAATTTTTTTTTATAGTTATTGTAGTTCTAGTTATCTGAATAATAGATCTAAAGGTGACAACGATCTGCACTATGATCCTTACATTAAGGATACTAAATATAATTGTAATAATCACATTAATAGTTGCATTGACTCTTATTTTCGTTCTCACATCTGTATTAATAGTCACTTTTTAAGCGATAGTAATAATTCCAATGAAAGTTACATTTATAATTTCATTTGTAGTGGAAGTGGAAAGATTCGTGAAAGCAAAAATGACAAGATAAGAACTAATAGTAATCGTAATAATTTAATGAGTTCTAAGGCTTTCGATATAACTAAAAACTACAATCAATTGTGGATTCAATGCGACAATTGTTATGGATTAATGTATAAGAAAGTCGAAATGAATGTTTGTGAAGAATGTGGACATTATTTGAAAATGACCAGTTCAGAGAGAATTGAGCTTTCGATTGATCCGGGTACTTGGAATCCTATGGATGAAGACATGGTCTCTGCGGATCCCATTAAATTTCATTCGAGGGAGGAACCTTATAAAAAGCGTATTGCCTCTGCTCAAAAAAAGACAGGGTTGACTGACGCTATTCAAACAGGTACAGGTCAATTAAACGGTATTCCGGTAGCTCTTGGGGTTATGGATTTTCAGTTTATGGGGGGTAGTATGGGATCCGTAGTAGGCGAAAAAATAACTCGTTTGATCGAGTATGCTACCAATCAATGTTTACCTCTTATTTTAGTGTGTTCTTCCGGAGGAGCACGAATGCAAGAAGGAAGTTTAAGTTTGATGCAAATGGCTAAAATTTCTTCGGTTTTATGTGATTATCAATCAAGTAAAAAGTTATTCTATATATCAATTCTTACATCTCCTACTACCGGTGGGGTGACAGCAAGTTTTGGTATGTTGGGGGATATCATTATTGCCGAACCCTATGCCTATATTGCATTTGCGGGTAAAAGAGTAATTGAACAAACATTGAAAAAAGCCGTGCCTGAAGGTTCACAAGCAGCTGAATCTTTATTACGTAAGGGCTTATTGGATGCAATTGTACCACGTAATCCTTTAAAAGGTGTTGTGAGTGAGTTATTTCAGCTCCATGCTTTTTTTCCTTTGAACAAAAATGAAATCAAATAAAACAGTTAGTTTATCATAATTAAACGAAAACCCTGAAAAATTCATTTTTCTTTAGAATCATTTTTTTATCGATATTCTTGTTTACTACTCAGTAAACCTTTATCAACAAGATAAAAAGTGAATTTTTGCTTTCGGGAAGTTCAAATTCGACTAGAAAAATAAAACAAAGTTTTTTTCCTCTCTTGCTTGCATATGGATAGATAATTCAAATAGAGATATAGATCTATAGAGAGTCTTGCATCGTTTTGCATTTCCCGAAAATTCCCTGTTGGTGGATCAGATTCCAATCAATTTTGTATAAAATTTTAATGGAATAAAATTTTTTCTTTATTAATGACTATTAGAAGACAAAAAGAACAAAAAGAATAATAAATCTAACAGGTAGATTATGATAATACATCTATTTTATTTTGAAAGATTAATAAGTCCATTTATTTAGTTTGGCGTTTCTTGTACCTATTTTTTTATTCTATTTCTAGTAGGTTCTATTCTATATATTTCTATTAGGTTGTATATTAGTATTCGATATATATTTACTTAAAGATACTTAGTATAATTATATAATATATATAATAGAAATAATAAAACTACAAGATATTCTAAGATATCTTTAGAATTCAGAATATAACAATAACAGGTACAAATATTAAATTGAGGTACCCCATTTTATGACAACTTTCAACAACTTACCCTCTATTTTTGTGCCTTTAGTAGGCCTAGTCTTTCCGGCACTTGCAATGGCTTCTTTATTTCTTCATATTCAAAAAAATAAGATTTTTTAGATCGGATGAGACCGAATCGTATAACTCCCCTTTTTATTTTAAAAACTTCGATTTGATAAGACCCATTTGGTAGAATATTGTATAACACATAGATTCCTACAAACATAACTAAAAAAAGTTTTTATGCATGTGTAAACGTATTATATGGGGTAACTCAATTTGCGCTCTTTTGAAAAAATGGATCATCATCGGACCGCTGGATGAAATTCAAGTCAATGTATTTATTTGTATGTATATAGTTATAGGGGATCATATAAAGGAAGGAGATTTTATTATTTTAGATATAAACAATTATATAAATTATTCCTAAAGTAAAGGTTCACAACAAAATAGTTATAGTTGATGAGAGTTACTTTGAAAACAAAAAAAGGAAAGTCATATTTTCTCAATTCCAAAAAATTGTATAACTGGATCTAATATATATGAGTTGGCGATCAGAATCTCTATGGATAGAATTTATAACGGGGTCTCGAAAAACAAGTAATTTCTGCTGGGCCTTTATCCTATTTTTAGGTTCATTGGGATTCTTATTGGTTGGAACTTCCAGTTATCTTGGTAAAAATTTTATATCGTTAGTTGCATCTCAGGAAATCCTTTTTTTTCCACAAGGGATTGTGATGTCTTTCTATGGGATCGCGGGTCTCTTTATTAGTTGCTATTTGTGGTGCACTATTTTGTGGAATGTGGGTAGTGGTTATGATCTTTTCGACCGAAAAGAAGGGATAGTACGTATTTTTCGTTGGGGATTTCCTGGAAAAAGCCGTCGCATCTTTTTACGATTCCTTATGAAAGATATTCAGTCGATCAGAATCGAAGTTAAAGAGGGTGTTTCTGCCCGGCGTGTCCTTTATATGGAAATTAGAGGTCAAGGGGCTATTCCTTTAATTCGTACTGATGAGAATTTTACTACACGAGAAATTGAGCAAAAAGCTGCTGAATTGGCTTACTTCTTGCGTGTACCAATTGAAGTATTTTGAAATGAATTCATTTTTAAAGTTTAAAGACTAAATCCTTTGGCAGTAGGAAGAAAAAACGAAAGAATTGCTTTCTTTTTTTTCAATTGAACATTAATCTATTCTTTTATGCTCGTTTTTTTATATATTTGATAGAAAAGAAAGGGAGTTTATTCGTCTCGAAAATAGAATCATATTTTTTATTTTAAAAATTCAAAAAAGTTCTTTTAGTATTGATCGAAAAAAGGGGGAATAACATCCTGGAAATACAATTTTTTCTTTATTCAAATTGTAAGTGTATTCTGAGTCTATTTCTGTATTCTTTCTAGATTCAAAACAAAGACTAAGTATTGAATCAAAAGAAAAAGATAAAAGGGATTATAGGCTCAATACATTCTATTTGAATTAGAATAGAAACTCATGCTCGATAGAAATAGTAGATCTAATAGAATCCACAAATGCGGTAGGTTCATTAACAATTCACAGATTCAAAATGGCAAAAAAGAAAGCATTCATTCCTTTTTTTTATTTTACATCTATAGTCTTTTTGCCCTGGTTGATCTCTCTCTGCTGTAATAAAAGTTTGAAAACTTGGATTACTAATTGGTGGAATACTAGACAATGCGAAACTTTCTTGAATGATATTCAAGAAAAAAGTGTTCTAGAAAAATTCATACAATTAGAGGAACTATTCCAGCTGGATGAAATGATAAAGGAATACCCAGAAACCGATTTACAACAATTTCGTCTAGGAATCCACAAAGAAACGATCCAATTCATCAAAATACACAATGAGTATCGTATCCATACAATCTTGCACTTCTCGACAAATCTAATATCTTTCGTTATTCTAAGTGGTTATTCCTTTTGGGGTAAGGAAAAGCTTTTTATTCTCAATTCTTGGGTTCAAGAATTCCTATATAATTTAAGTGATACAATTAAAGCTTTTTCGATTCTTTTATTAACTGATTTATGTATCGGATTCCATTCGCCTCACGGTTGGGAACTAATGATTGGTTATATTTACAAAGATTTTGGGTTTGCTCATTATGAGCAAATTTTATCTGGTCTAGTTTCTACCTTTCCAGTCATTCTTGATACAATTTTTAAATATTGGATCTTTCGTTATTTAAATCGTGTATCTCCGTCACTTGTAGTGATTTATCATGCAATAAATGACTAAAAAACGATTCACTGATCCAATTCTACTCTTTCTTACTTTATACATCATAACCAAATCAAAGTCGTATTTACTTTACTCTTTTTTACCCACGAGGGATTCCTTGTATATTAAAAAAAAAAAAAATTTCTTTTTTCAGTAAATGTAAATAACAGAATTGTGGCTAGGGAAGTATATTATCGACCTACCTAACTTTATTGTAGAAATTTTCGGGATAAACGATTGGACCATGCAAACTAGAAATACCTTTTCTTGGATAAGGGAAGAGATTACTCGCTCCATATCTGTCTCACTCATGATATATATAATAACTTGGGCATCCATTTCAAGTGCATATCCAATTTTTGCCCAGCAGAATTATGAAAATCCACGAGAGGCAACTGGGCGTATTGTATGTGCCAATTGCCATTTAGCTAGTAAGCCCGTGGATATTGAGGTTCCACAAGCGGTACTTCCTGATACTGTATTTGAAGCAGTTGTTAAAATTCCTTATGATATGCAGCTAAAACAAGTTCTAGCTAATGGTAAAAAAGGAGCTTTGAATGTGGGAGCTGTTCTTATTTTACCGGAGGGGTTTGAATTAGCCCCCCCCGATCGTATTTCACCCGAGATGAAAGAAAAGATAGGAAATCTGTCTTTTCAGAATTATCGCCCCAATAAAAAAAATATTCTTGTGATAGGTCCTGTTCCTGGTCAAAAATATAGTGAAATAACCTTTCCTATTCTTGCCCCAGACCCTGCTACTAATAAAGATGTTCACTTCTTAAAATATCCTATATACGTAGGTGGAAATAGGGGAAGGGGTCAGATTTATCCTGATGGTAGCAAAAGTAACAATACAGTTTATAATGCTACGGCAGGAGGGATAATAAGTAAAATTTTACGAAAAGAAAAAGGGGGATACGAAATAACCATAGTGGATGCATCGAATGAACGCCAAGTAATTGATATTATCCCTCGAGGCCTAGAACTTCTTGTTTCAGAGGGCGAATCCATTAAACTCGATCAACCATTAACAAGCAATCCTAATGTGGGTGGATTTGGTCAGGGGGATGCGGAAATAGTACTTCAAGATCCATTACGTGTCCAAGGCCTTTTGTTCTTCTTAGGATCTGTTGTTTTGGCACAAATCTTTTTGGTTCTTAAAAAGAAACAGTTTGAGAAGGTTCAATTATCCGAAATGAATTTTTAGATCTGTCTATTTCGCCTTATCAAATTCGTAAAAAAGAAAGAACCAAAAAAATTATCAAAAGCCTTTTTGCCTCTCTTTAGACTTTCGATTTCGACCAGGTGTCAGGAATTACTTGTCTGATAGTCCTAATCCTAGTATGTATATTAAGAAGAATTCACTTTACCCCCCCTTTTCTTTATTTTTCAATACAAATTTGGATTGAAAAATGGGAGGGGGTGTGATGTAACTCTGTCGTTAGTGACCAATTGAAATTGATAGAATGTATCAATAATCAAGAGTTTTTTTCTAATGTAATTTAGAATGGAAAAATTTGACTAGATACTAAAATAAGGAAAGCAAGCGCAGAAAAAAGGGAACTAGAAATTGGCGAAATAACAAATTTTAGGGACTATAGGGAGTATTACTTGTCTTGCGAGTCTTCGACACAAGAAAAGGAATTTTAGACATCCTTTTCTTGTGTCGATCTTGTCATTCTTAATTGCATTCGTTAAAAAATCCTATTCTTAGTTTACATATATATTACTGTTTCTATATATATAAGGTTTTAATATATCTATATTAATATATATCGTATATATAATTATTAATTAATAGTATAATAATAGTTACTTTTTGTAGTTTTATTTATTTTAATTTCAAGTTTGATGAAATACTAAAAAAATAAGAAAAAACTGCTATTAGTATAGAGTATAGACAAAATTTTAATGATAGATCTAATGATAAAAAATATTGTGTCAGTCGGGAAAGCAGAAAAATAAAATTAAGTGATCCCCCCCCCTTTTTTTCGATCTTTGAAAGGTTAATAATAAATACTATATGCTCGTAACCTACTAATCTAATTAAGTTCATTTTTCAAAAACACGATAAAAATTGTTCTTATTATTAGCAGTTCAACGGGACCCCCTCGAATCAGACAAAGAAGGAAGAGTTGGGCCCCGTTGAGTTCTTATGTTTTCACGTCTATAACTCAGTTCATCCAATTTCTACAGGGATGAACCTAATCCTGAATATGAACCATAAAAGAAAATACCTATTAAACCGATCACAAGAATACCAGCTACAGTACCTATTACCCAAAGAGGAATCCTTCCAGTAGTATCAGCCATTTATCCCGCTTCCCTCCACATTTCATCGAGTGGTCATGCTAGAAACATAAACAGTCAGAGATAATTATGATATATAATCCATCCGAATGGGATAAGAAAATTACTACTCTTTGTTTTTTATTTTATTATTCTACGCTCTTTTCTTAATTTTACTTAATTTTAATTGAAGAAATAATTTGAAAATAAAACAGCAAGTACAAAAATGAGTAATAACCCCCAATAGAGACTGGTACGATTTAATTCAACATTTTGTTCGTTCGGATTTGATTGTGTCATAGCTCTATAAATAATTGAATTCGGTTTATCGTTGGATGAACTGCATTGCTGATATTGACCCCAAAAAAGAAACGGTAGGTACAGCTAGTCCATGAACAGCCAACCAGCGCACTGTAAAAATTGGATAGGTCCTATCTATAGTCATTGGGTCCTCCTAAAAAGATCTACTAAATTCGTCGAGTTGTTCCAAAGAATCAAAACGGCCTGTTATTAATGGAATGCCCTGTCGGCTCTCTGTAAAATACTCGTTTGGACGAGGGCTCCCAAACACATCGTAAGCTAAACCGGTGCTGACGAATAACCAGCCCGCAATGAATAGGGAAGGTATAGTAATGCTATGAATGACCCAGTATCGAATACTGGTAATAATATCAGCAAAAGAACGTTCTCCTGTGCTTCCAGACATACTGAACTCCAGATATTCTTGTAGGGAATCGATTCTGTAAAAGATGAATCAGTAAATTCAAATTCACTGAGATTACATCTTTGTGAGATCGTCAATAAAGTACCAAGGGTATTTTTAGAGTCTACCGAATCAGTATAGCTATCCTTCTTCTGACACAGCAACGCAATTTGAATTAGTATAGAACTGAAGTGTTAGATAATTTATTTCGTTTTTTTTTTTTGCTTGTCGATGTATAACCATGTTCCACTTCTTCAAATTCCTGTATCTGTAATCTATAGGGGCTTTGATCCTTTATTTGTTTTGGACTAGAAAATAAACTAAAGATTAGATAAAATGAAAATTCAAGAGGGTGGTTTCTAATTCTAATAATTCATTAAGGAAATTCTCATATTGTCCCAAGTCAATTGAATCCAAAAACCATCAATTTCATTTTTGTTGCATATGCATAATTGTAGAAGAGATTTTTTTGTAATGATAATGTGACCCCCCCCGTTTTTGCTTTTTATTTCATTTTATTTTAAGGAATTAATTTACAGTAACAAGAAAAGGAAGAATAGTTTTGGATCAATTCAAAAGAACAACAAATAATAAAAAAATAATAATACTAAATCTTTGGAATGCGTGCATTGTTTTGTTGTATTCAATTTAAAGGTTTTTTCTTTCTTTAACTAACTACAAAGATGATGGGTTTTTCACTCTATTTTCTATATAATATCGAAAGAAAAAAACCTAAAACCTAGAAAGGAAACGATAATAGAAATTGCTAATTACAAGTTTTAAAAATCTAGTTAAAATAAATAACTCTTTTTTTGACTGATCTCGTTCTCCGTGTAGGATACTGCTTTTTGGTTTAATTTCATAGATTAAATGAAGAAAACTGCTCTACTATATTAATCTACTTTATTCAAAATTGAATTTATTTCAATTTGAATAAATGTAGAAGGGGGCGTATTCTAGGTTCTAAGGTCACTTAAAAAAAAAAGAATCAAACAACTTTTTTTTTAATTTTTACATGTTTATTCAAAAAAAGTTCTATGTTTTAACTGAAGTTATATAATAGAGTTATTTTTTTTATGTATTATTTTTTCTTATTAATTTCTTAAAGAGTTTTTCAATGAATCAGTTACGTGAATTCTGAATCCTGAGATGGTGCAGATGCCAAAGACGATGAATTTCGTTTTTTCTTTTTCTCTATTTTTGTTCATACCACCGATAATGCTTGATAACTCACAAATTTTCAATTTAATTTTTTGATTCTTGGAACTAGTATTTGTATCTATCTTTACTTTTAAAAATTTTTTTATTGAAACTTCGGGAAGTACTTTAGAAACATATGTATAAAAAAACATATTTTATTGAGTCCCTTCATGCCTACTATAACTAGTTATTTCGGTTTTCTACTAGCAGCTTTAACTATAACCTCAGTTCTATTTATTGGTCTAAGCAAAATACGACTTATTTGAAATTAATTGAATGAATCTTTTTTGATCAAAAAAGATTTATATGGTATTTCATATGTTCGATAGTTCCTTACCGTGTTAATTACCCAATTTTGGTCATTGAGATTCGTCGGCAATACAGATTAAGAGCTAGGAATAGATAGTACCTCTCTTTTCTCCCTTTCAAAAATGAAAACAAAAGAAAATTGAAATGATTGAAGTTTCTTTATTTGGAATCGTCTTAGGTCTAATTCCTATTACTTTGGCTGGATTATTCGTAACTGCTTATTTACAATACAGACGTGGTGATCAGTTGGACTTTTGATTAATTAACATCTCTTTTTTTTTACTGACCTCCTTCTTGCTTTCATATGCGGGAGGTCGAATTCAGATTGCTGCTCAATTATTTGCGAACAGTGGAATTTTGACACAATCTAATAAACAAGAGTGACATCACGCTCTGTAGGATTTGAACCTACGACATTGGGTTTTGGAGACCCACGTTCTACCGAACTGAACTAAGAGCGCTTTTCTTGTTTTTTCTAAAAAAACGAAAAGGCTAGAAAGAGGACATTCTTTAACTCGAATCGATTTTGTACGTATATACTATATCATAGTATATCATAAAATTCAGAATTATATGTATGTCCAATTTTATTAAAAAAAGATAAATCTAAAATGGATTCCTCGTTACTGCTCTTCTGAGCAGTAATTAGGTAGGGATGACAGGATTTGAACCCGTGACATTTTGTACCCAAAACAAACGCGCTACCAAGCTGCGCTACATCCCTTTCGATTGGTTTACAGTGTCATTGTAAACAATTCCTATCTTGTTTTCCACATCCTTCTTTTTTTTTGTTTCATATCAGATAACAAACATATATATAAGTATAATTAAAAAAATTACTTTTTTTAGGCAAATCCTATCAATTTCAAATTTACATAAAAAGGCGTTTCCATTTTCAAATGGAATCTATAAGATCGTTCTAGTAGACAATATTTCAATTCTAATTTTGAAAATGGGGGGTTACATATACAAATACAAGAACTTCTTAACTACATGTACATCTATAGTTATATATATTACTATATATATTGTAATACAATAAAGAAGAAAGAAGGAGGATTTCAAATGCGAGATCTAAAAACATATCTTTCCGTAGCACCGGTACTAAGTACTCTATGGTTCGTTTCGTTAGCAGGTTTATTAATAGAGATTAATCGTTTATTTCCAGATGCATTAACATTTCCCTTTTTTTAATTCTAGTTATTAACATCAGAAAGGATAAAAAAATTTAGAGATACGATCAACGATCGGGGAATAACCCCCCCTTTTTTTTCTAATTCTTTTTTAAGAATAAAAAAGAATTAGAAAAAAAAAAAGGGGGCCGAAAGGGTCATAAAAACGAGGGTTCAGAATCCAATAAAAAAAAAAGTGTTGCTAGGGAAAGAGTATCCTACGAGATACTTAAAAAAAATACTGTACAAAGATTTGAAATATAGTTTTCAAAAAATCATTATATTACTTATTATTTTCTTTTTATTTAATTACTAATTAATATTCATTGCAACGAAATATTTAAGACATTTTTTTGAGTTAATTAACAGCTTCTATTTTTTTTTGTTCTTGTTCTTTATGGACCCTAAAATTAAAATAGAAGATTGGGGGTGAATCATAAATCCAAAGGAGGTTTCATGGCCAAAGGTAAAGATGTTCGAGTAACAATTATTTTGGAATGTACCAGTTGTGTTCGAAATGATATTAAGAAAGAATCGGCTGGAATTTCCAGATATATTACTCAAAAGAATCGGCATAACACTCCTAGTCGATTGGAATTGAGAAAATTCTGTCCCTATTGTTATAAACATACAATTCATGGGGAAATTAAGAAATAGATAAAATTGAGTGCTTGTATGTCAAATTTTATTTTAAGAACAGGAATAATGAGAGTATCTACGTATTATTACATATATATAAATATAAACAAATAAAATAATAGAAATAAATCAAATCCTATATTCTTAATTCTATATAGAAACTCTATCCTATATAGAAATAGAAATCGTTTTTATTTTGATCCAATCAAAATAGGATTTTATAGGTAAGGAATAAAAAATTATGAATAAATCTAAGCGACCTTTTACTAAATCCAAGCGATCTTTTCGTCGGCGTTTGCCCCCGATCCAATCGGGGGATCGAATTGATTATAGAAACATGAGTTTAATTAGTCGATTTATTAGTGAACAAGGAAAAATATTATCTAGACGGGTGAATAGAGTAACTTTAAAACAACAACGATTAATTACTATTGCTATAAAACAAGCTCGTATTTTATCTTTGTTACCTTTTCTTAATAATCAGAAACAATTTGAAAGAAGTGAGTCGACCCCTAGAACTACTAGCCTTAGAACCAGAAAAAAATAGACTTATTCTTCAATTGAATAACTAATCTGAAGGAATTAAAAAAGAGGTTAATATTTTGTTCGACAAATCCAATTAAGAATCAAAATTTGATTGTTACGTCTGTTTCTGTCATAAAAAAAAAAAAAAGAAAAGAATCGTCGAAAAGAAAAAGAATAAGTCTTTTTTTAGCGACTATATACCCTCGTTTTGTTTTGACGACTTTTTTTATAATACTAATTTCTACTCTACCCTCCCCGAGCTTATTCTACTTAAGAACTCTATTTCAAATATTTTAGTGGATTTCTTCCAATCCCCTCATTTTTTGATCTCATTTGAAATCGTATAAAGACAACTCCTATTTAATAGAGCTATTTGTGCAAGTATTTTTCGATTAAGAAGTAATTGCTTCTTGTACAGATTGTGTATGAATCGGTTATAACTATAGAATACCTCCGTTTCGTGAATTACGGCATTTATTCGAGTGATCCATAAACGACGAAAATCCCTTTTTCTTTTACCCCTATCCCGACGAGCCGAAACTAAAGCTCTTATTCTCTGTTGAGTCATAGTTCGTGTAAGTCGTGAATGAGCCCCTTGAAAGCTTGATGCAAATAAACGAAGTTTTGTTCTACGCCTCCGAGCTATATATCCGCGTTTAATTCTAGTCATTGAATAAATCAAACTTTGATGAATAACTAATTCTTTTTTTAGTTTTAGTTATTCTTTTCCCCTTTACTAGTCATTAATAACCAAACGAATTATTCCAATGTATAAAAAAAAATTCCAATGGCTTTTGCTACTCTAACCTTCCCCACCACTATTTTTGGCTAGGTATTTTCCTTTGCTTTGAAAGGATAAATTGCCTTGGTACTTGATATAAAAAAAATAGAATAACTACAAAAAGTAGTAAATAGAAATGGATAAATAGTGGGTTCCTTCGTTTCTATGGTTACTTCTAAAACGGTGAGGTCCTCTCTATACACCGGAGCTCCTTCTTTTAATTAATCAATACTATTGGTAACTTGTACAATTCACATTCTTTGGCTCTACCCCATTAATATTCCAGTAATAGATCTTTCACAATGAGATCCACTTTATACAGTAACGGTATTTTATTTTAAAATTGATTTGGTCATTTACCCTGTTAGTCCGTTTTTTCTTTCAAGAGTGGAATCTTTTTAATAAAAAATGGGATTTCCCCCGCTTAATTGATAACCATTTGTTATCATTGGGGGTTTTCTAAAAAATGGAGTTGATTGGATTTGCACCAATGTAAACCATAAGTTTCAGACACAATAGAAGATATGAATGATCTATCTTTTTGAAATAATGAATCGAGTTCCTCCATTCTATTTTATTTTATTAACAGGTACTGATCCTTGATATTTCAAAAAGAATTTCCTTTTTGTGTTTCAGTCTATGATCTAAACGAGTCGCACATACACCCGAGTACATGTTCCTCGTCGCTGAGGGCATCCCCGAAGCGCTGGGGATTTTGTGACATTTCGGATTGGCTGTCTTGTATTTCTAATAAGTTGTTTAATGGTTGGCATACGGAATCATATAAATAATGGGCTGGTTTAGATGGGTTCTAACCGGCTAATTCTGAATTACTTCTCTTCAAGATTCTCTTCAATATATTTTTTTTATATTGAAGAGAATATGAAACTAAACCTTTAATCTAAAAAGATATAAAATTAGCAGTGGTAGATTTGCATGAAATCGCTCCTATTTTTATTGAACCGCTACAAGATCAACAATGCCATGAGCTTGGGCTTCTGTTGCTGACATAAAAACATCCCTTTCCATGTCTTCGGATACAACCCATATAGGTTTGCCCGTTCTTTGTACATAAACCCTTGTGATGGTTTCGCGAAGTTTTAGTAGTTCTTCCGCTTCCAAGATAACTTCTCCCGTTTGTGCCTCATAAAACGAACTAGCGGGTTGATGGATCATTACCCTGATGATATAATAGAAAAGCTTTTTCTATTTCGCAGAATGAGGCGAGATAACCAAAAAAACAGAGAAATTTGAATAACCGTACAGGCTTTTTTTGTGCGTTGCATACGGCTCTAGAATGGAATTTACGTTTTTGACCTTTCCTTTCGGCGAAAGAAAACAAAATATAGGTTCTATTATACGCGGATCCATAAATGATCCAATTACCATCCTTCTTTTTTGTTTTGTAGGAGTTAAAAAAATACTATGATGGTTCCGTTGCTTTATATATCATTTTTTTTGATCCGTCTATGATTCAGCAATCCCAAAGTGTCTTTTTTTTTGTTTTTGTAAATAAGCTTCCGGTGTGAAAACAAAGTTTGTGACGCTGGGATGTGCCCGAATAGGGAAGATATCATTTTAAATACCCCTTTCTTATCCCATACTACTCTTTCAATATATAATCTAATTTTTTTAATCTAAAAAATTTCATATCGAATTCGAAGTGCCATGCTATTATTACTTAACTAATTCATATTTCCGAGGGCGAAGGCATAGTATTTTTTCTCTAAAATAAAAAAACTCATTGGCGCCAAGCGTGAGGGAATGCTATACGTTTGGTAATTTCTCCTCCGACTAGGATAAAGGATGCTATTGAAGCGGCCAATCCCATGCATATTGTCTGTACATCGGGTCGCACAAATTGCATAGTATCATAAATAGCCATTCCAGATATTACCCATCCACCAGGAGAGTTTATAAACAAATAAAGATCTTTGGTATCCTTTTCTATACTGAGATATATCATAAGACTAATAAGTTGATTCGAAATTTCGGTATCAACCTCTTGGCCTAAAAAAAACAATCTTTCTCGATAAAGTCGGTTGATTAGGATAAAATTTTATTCCTTAGGAGCCGTACAGGCACCTTTTGATGCATACGGTTCAACAAAAATTGTTAAAAAATCAATGTGTCGATTCCAACCCCCTTTTTTTCAGAGAAGGCTTTTCTTTCTAACTTAATAAGGGAAGGGCTTGCTTCCCTTTTAAAAGTAAAAGAAAAAATAAATAAGTTTTGGCCCCTTTTATTTATTAGATATTATAATCCTAATAATAAAATAATAAAACGATTGATTAGGCCTGTCAGACTAACTTGATTCATTGATATTTTTTTTTCATCGAGATTCAGTTGAAATGGGGATGGTTTTTTCTTGTTCCTGAATGGGCTTCTTCCTTTTTTTATTCTGTTTTTTTAGGTTTATGCTCTACTCCGAGTAAAAGGAAAAATTTGCCCGATTTTGATTTGCACATATAGGACAAATGAACCAAATACCGCGTCTTTTTTTTTTTTACTACTCCTTCTTTTTTTTTCAATTCATTTCTTTCACATGTCTTCTGTCAAATAGTCAATAAATTTTTAATTATATTATTTTATTTGATCAACAGTTTTAGATCACCCTGTTTCAATTTTTTGTATTTTTTTTATTTTTTAATAGAATTTTTATCATAATTTTGATATCATATTCATATCATATTAAGTAGTAATTATAAAAATATTATATATTAATTATCAATTGGATTTTTGCTAAACGGAGCCTGGATACTTAATTTTATTAGTCCGATCACGTAAACCATAAAAAATTTTTGATAATCTAATATCAATCTAAATACTCCCTGCATTTAATTCTAATTTATTTTTTGCGCTTCGCGTTACAAATTTTTGATAATTCAATCAATCTTTTTGAGCGAAACAGAGGATATCTCGATCGAGGGAGAAAATGGGGAAATCCCATATAGCCCAATATATCTGACAAGTCGCACTATATGTCAACCCAAGATGTATCTCCTTCTCCAGGACTTCGAAAAGGTACTTTTGGAACGCCAATAGGCATGAAATGAAAAAAAAAGAGAATGAAGTTCTCTATTTCACTTTGATGTGGAAACGTAAGACTGGGGTTTCATTTTTTTTATCATATTATCCTTTTTTCCTACTTTATTAATATTAATCATATTTAAATTAATAATATTTAATACAGAAGTTGAATAAGCTAAAATAAAATATAAAATAAAAGTAAAGTAAGAGAGAATGAATAAAAATGAAAGGAAACTTTTTACGAACGGGCTTCTGAACGATGAACAACAATAGCTATCTTGGTTCATATAACATAGGATTCACCCCCATTGCGTATTGGTACTTATCGGATATAGAATAGATCCGCTTCCCTTTTTTCCTATGAATCGAATTGTTCCATTATTACTAACAGAATAGAACAAATATTAATCCTTTCTCCGAAATAATTACCTAAAAAGGGGGGGTCCGTAACATAGTTTTTTCCAATGCAATAAAGTTACATAGTGTCTATTTTTCATTGATAAAGGGGTATTTCCATGGGTTTGCCTTGGTATCGTGTTCATACTGTTGTATTGAATGATCCCGGTCGTTTGCTTTCGGTTCATATAATGCATACTGCTCTGGTTGCTGGTTGGGCCGGTTCCATGGCTCTATATGAATTAGCTGTTTTTGATCCCTCCGACCCTGTTCTTGATCCAATGTGGAGACAAGGTATGTTCGTTATACCTTTCATGACTCGTTTAGGAATAACCAATTCATGGGGCGGTTGGAATATTACAGGAGGGACTATAACGAATCCGGGTCTTTGGAGTTACGAAGGGGTAGCCGCAGCACATATCGTGTTTTCTGGCTTGTGCTTCTTGGCAGCTATTTGGCATTGGGTATATTGGGATCTAGAAATTTTTTGTGATGAACGTACAGGAAAACCTTCTTTGGATTTGCCCAAGATTTTTGGAATTCATTTATTTCTTTCAGGAGTGGCTTGCTTTGGTTTTGGCGCATTTCATGTAACAGGATTATATGGTCCTGGAATATGGGTATCCGACCCTTATGGACTAACCGGAAAGGTCCAACCCGTAAATCCGGCGTGGGGCGTGGAGGGTTTTGACCCTTTTGTTCCGGGAGGAATAGCCTCTCATCATATTGCAGCAGGGACGTTGGGTATATTAGCGGGCTTATTCCATCTTAGTGTTCGTCCGCCTCAACGTCTATACAAAGGATTACGTATGGGAAATATTGAAACCGTCCTTTCCAGTAGTATTGCTGCTGTCTTTTTTGCAGCTTTTATTGTTGCTGGAACTATGTGGTATGGTTCTGCAACTACTCCCATCGAATTATTTGGTCCTACTCGTTATCAATGGGATCAGGGATACTTTCAACAAGAAATATATCGAAGAGTTAGTGCCGGACTAGCTGAAAATCAAAGTGTATCAGAAGCTTGGTCTAAAATTCCTGAAAAATTAGCTTTTTATGATTATATTGGTAATAATCCAGCAAAAGGGGGATTATTCCGAGCGGGTTCAATGGACAATGGGGATGGAATAGCTGTTGGATGGTTAGGACACCCCGTCTTTAGAAATAAAGAAGGGCGTGAACTTTTTGTACGCCGTATGCCTACTTTTTTTGAAACATTTCCGGTTGTTTTGGTAGACGGAGACGGAATTGTTAGAGCCGACGTCCCGTTTAGAAGGGCAGAATCTAAATATAGTGTCGAACAAGTAGGTGTAACTGTTGAGTTTTATGGTGGTGAACTCAATGGAGTAAGTTATAGTGATCCCGCAACTGTGAAAAAATATGCTAGACGGGCTCAATTGGGTGAGATTTTTGAATTAGATCGTGCTACTTTGAAATCCGATGGTGTTTTTCGTAGCAGTCCAAGAGGTTGGTTTACTTTTGGGCATGCTTCGTTTGCTCTACTTTTCTTCTTTGGACACATTTGGCATGGTTCTAGAACCCTCTTCAGAGATGTTTTTGCTGGTATTGATCCAGATTTGGATGCTCAGGTGGAATTTGGGGCATTCCAAAAACTTGGAGATCCAACTACAAAAAGACAAGCAGTCTGATGCAACATTGCTTTTTTCTTTTAGTTTCTGTTTGCGATTTTTTTGATTTCATTTTATTTAATAGGTAGGGTACTGTAGGAATCTTGATTTAAATCGCTGCCGTTTCTTTGACTCTTTTTTGTTCTTTATCCGGAGGTATACTCCTTCAGTAAACATAAACAAAACAGGTATGAAAGCTATAATTGTAAACCACGATCAAATTTATGGAAGCATTGGTTTATACATTTCTCTTAGTATCGACTTTAGGGATCATTTTTTTCGCTATTTTTTTTCGGGAACCGCCTACAATTTCAACTAAAAAATGAAATAATTTTTCATTCTCTTCATTGACGTAATCAGCCTCCAACTATTTGGAGGCTGATTACGTCAACTAGTCCCCGTGTTCCTCGAATGGATCTCTTAGTTGTTGAGAGGGTTGCCCAAAGGCAGTATATAGAGCATACCCAGTAAAACTTACAAGTAACCCAGATATAAAGATGGCGACTAGAGTTGCTGTTTCCATTATTATATAATTGAAAGACCACAATGGATCTATGCTAAGATCGTTTATTTACAACGGAATGGTATACAAAGTCAACAGATCGTAATGAATACAAAATAAGATTTATGGCTACACAAACTGTTGAAGATAGTTCTAGATCTGGTCCAAGAAGCACTACTGTAGGGAAGTTATTGAAACCGTTGAATTCTGAATATGGTAAAGTAGCTCCTGGATGGGGAACGACCCCTTTGATGGGTGTTGCAATGGCACTATTTGCGGTATTCCTATCTATTATTTTGGAGATTTATAATTCCTCTGTTCTACTGGATGGAATTTCAATGAATTAGACTGAGAAGAATCTTGAAGTCCTAGCTTTTTGTTCGATACAAAAAAGTAAAGTATGTAGGTCTAAAATTTTGCACCTATTCTCCTTTGGTAGTTCGACCGCGAAATTTTTTTCTGCATTGTATATTTCCGGAATATGAGTGTGTGACTTGTTAGAATTGACCCTATGGATAGTACAGAGAAGGGGGTCTGTCATCTTTATCAAGATGGTTTTATTTCGTCGGATATTCATTCGAGTATCTGGAGCACGAAATAGATCAAATAGATCACAAAGTTTTCGAACTATGATTCATACTTAATACTTAGACCTCGTAGCCGGACTTCTTTCCGTTCTATCTTATAAATTTTCATAAATCAATTTTTTTCTGCTTTTAAACTCTTATTTAGATCAAAGGACAAACGCTTCTTTGTATTTTATGTTTTTAATCATTATAGCTCTTTTTTTTTTTTATTGAATAAGTGATGATCCAATGGTTCTCACTCAGTGAACTTTGGACTTTGAAGGTTTCATTGAATTATCGTGGTTTTCGTATGAATCTGAGGTTTCAATTAATAAGTAGGGTCTTAACAAGAAAATTCCTATCAATAATAAAGAAAACAAGAAGAAATCCGTATTCCCATTCCATACAAATACCAACTAAAAAAGACAATAACGATAGGTAATCTAGAAGATTCAAGAGGCCTGTAACGATCAACACAACATAAAGACGTATGAGCTGACTTGAGTTTTTGGCATTTAACCACAAAGAAGAGCTTTCGCATTTTGACTCTTAAATAATATTGAATGAGAGAGAAGTTTAAAACTTTATATTCCATATCCGTTTCAATCAGTATTTGGGTCTTTTTTTTGTTTGAGCTGTACGAGATGAAATTCTCATATACAGTTCTTGGAGGGGGAGGAACCTTGGTTTACCTATCTCAATAAAGTTTATGATTGGTTCGAAGAACGTCTTGAGATTCAAGCGATTGCAGACGATATAACTAGTAAATATGTTCCTCCGCATGTCAACATATTTTATTGTCTAGGAGGAATTACCCTTACTTGTTTTTTAGTACAAGTAGCTACGGGATTTGCTATGACTTTTTATTACCGTCCAACTGTTACTGAGGCTTTTGCTTCTGTTCAATATATAATGACTGAAGCTAACTTTGGTTGGTTAATCCGATCAGTTCATCGATGGTCGGCAAGTATGATGGTCCTAATGATGATCCTGCACGTATTTCGTGTATACCTCACCGGTGGTTTTAAAAAACCTCGCGAATTAACTTGGGTTACTGGTGTGGTTCTGGGTGTATTGACCGCATCTTTTGGTGTAACAGGTTATTCTTTACCTTGGGATCAAATTGGCTATTGGGCAGTCAAAATTGTAACAGGTGTACCTGACGCTATTCCGGTAATAGGATCGCCTCTTGTAGAATTATTACGCGGAAGTGCTAGTGTTGGACAATCCACTTTGACTCGTTTTTATAGTTTACACACTTTTGTATTACCTCTTCTTACGGCTGTATTTATGTTAATGCATTTCTTAATGATACGTAAGCAAGGTATTTCTGGTCCCTTATAAATAATATAGATTCTAGATATTTTTAATTACTAATTTATCTTATTACTTGGTGAAGGAACAATCGTATTTTATTGCTATAAATATGGATTATTAAAAAAATAAGACATGTATTTGGATATTTCCCTTCAACTCCACAATATTGTATTATTTTTTTGACATAAAAAGTTGAAGGGAATTCTATGAAGAGAAAATGGATTATGGGAGTGTGTGACTTGAACTATTGATCGGGCCGTGCAGAAATATGACTTTATCTGCTACATTGGAATTCACAACCAAATGTGTCTTTGTTCCAACCACTGTGTAAGCCCCATACAGGGGATAGGCTGGTTCACTTGAAGAGAATCTTTTCTATGATCATAATACCCGACGATGTCGTGGATGAGTGGGCTCCGTAAAATCCAAAAATCCAGGAGATTAAGGGATGGAACATAATCAGGATTATGTTTTTAGCTATTTTTTACTAAAAAATAGCTAAAAACATAAAAAATTAATAGTATGTAAATGCATTCATTTCCTCTGCATCGACTCGATTTCTGATACTATCGGAGTGAATACAGGATCTAATGAAGAGTAGAGGGTAGACTTCATTAGTAACAAGTAAATCCTTTGTATTTGAAAAATCTCGATATAATTTTTGAGATTAAGGATTAATTGATAAGGTATGAGACGATCCAGAAAGCACTTAATCATGATCAACTTTTAAGCTTACGTGGGTGTTGAGCATTTACCTGTAAGAATGGAATTTATGGTAATCTTTAGTTGCAATAACTTTGGAATCGGATAATTCTTTTTTTACATATTAAATACTTGTTGATAACATATATATTTTTTGTATGTATTAATTTAGTTTGGTTAATTCTTGCTCGAGCCGGATGATGAAAAATTATCATGTCCGGTTCCCTCGGGGGATGGATCCATAAGAATTCACCTATCCCAATAACAAAAAAACCAGATTTGAATGATCCTGTATTACGAGCTAAATTAGCTAAAGGTATGGGTCATAATTATTACGGAGAGCCCGCATGGCCCAATGATCTTTTATATATTTTTCCAGTAGTCATTCTTGGTACCATTGCCTGTAACGTAGGCTTAGCGGTTTTAGAACCATCAATGATTGGTGAACCTGCGGATCCTTTTGCAACTCCTTTGGAAATATTACCTGAATGGTATTTCTTTCCTGTATTTCAAATACTTCGTACAGTGCCTAACAAATTATTGGGTGTTCTTTTAATGGCTTCAGTACCGGCGGGATTATTAACCGTACCCTTTTTGGAAAATGTTAATAAGTTCCAAAATCCATTTCGTCGTCCAGTCGCGACAACCGTCTTTTTGATTGGCACCGTGGTGGCCCTGTGGTTAGGTATTGGAGCAACATTACCAATTGATAAATCTCTAACTTTAGGTCTTTTTTAATTAAATTTATTCAATTGTAAAATAAAAGGCGTGGGTATCTAGGGAGTAGTCATTTCAAAATGAATTCTCCCTAGATACATATCTAAATTAATTTTATTAAGTAAAATAGGTTTGACTGGAAAATCGAAATTACGTTGAAGGTTTAAAATCCATTTCAATTTTAAATTGACTTTTTAGTCAAATTTTTTTTAATGCTTTTTTTATTTTTTTTCTAAAATGTCTAATATCTTTTTTACATCTTCTATGTGAAAATGTTCCATTTTGATAAGGTCTTCTTGACTGTTATTCAAAAGATCCAATAATGTATGTATATTGGACTTTTTGAGACAATTATAGATTCTGGGAGGCAATTCTAATTGGTCAATAAAAATATATTGAAACGCTAGTTCTTTTTTTTTTTTTCTTAGGTTAACTAATCTATTATGAAAAGGAAAAAGGGGTAAAGTAACTTGATGTTGATTGTTCTCTAAATAGAACGTTTCTTCTTCTACATGTAGAAAAGGAATAAATAAATTAATCAAATTCCGGGAGGCTTCATGAAGTGCTTCTTTAGGAGTTAAACTTCCATTTGTCCATATTTCTAGAAAAAGAATCTCTTGTTTTTCATTCCCATTCCCATAAGAATGAATACTATGATTCGCATTTTGAACAGGCATGAATACAGCATCTATAGGATAACTTCGGTCTTCAAAGTTATTTGACATTTTTAAACTATATCCGCGATTCCTCTCGATTTTTAATCCAATACACAAATTTATTGGTTCCGTTAAGGTAGCTATATGCTGTGTATTATCAACGATTTCCACAGAGGGCGGTAAAACTATGTCTCGAGCAGTTATATATCCGGGACCTTGGACACAAATAAGCGCGTTGCGCGTTCCATATAGATTACTTTTTAATACAATCTCGTTCAAATTCATTAAAATTTCATGTACTGATTCTTGAATACCGACTATGTTAGAATAGTCATGTGGTATGTTCTCAGATTTTGCACGTGTAATACATGTTCCTTCTATTTCGCCAAGTAAAGCTCTTCGCATCGCAATGCCTATTGTGTCGGCTTGACCTTTCATAAGTGGAGACAGAATAAAGCGTCCATAATAAAGACGCTTACTGTCTCTTCTTGATTCAACACACTTCCACTGTAGTGTCCGAGTAGATACTTTGACTTTCTCTCGAACCATAGTAATTTTATTTGATCAGATCATTGAATCATTTATTTCTCTTGAAACCCTTTCAGCCTTTATTTAGTTCTATACACGTCGTTTTTTAGGGGGTCTACAACCATTATGTGGCATAGGGGTTACATCTCGTACGAAACTTAAAAGTATACCGCTTCTACGAATAGCTCGTAATGCTGCATCTCTTCCTAGTCCAGGGCCTTTTATCCTTACTTCAGCTCGTTGCATACCTTGATCCACTACTGCTCGAATAGCATTTCCTGCTGCGGTTTGAGCAGCAAAAGGTGTTCCTCTTCTTGTACCCCTGAATCCACAAGTACCCGCGGAGGACCAAGAAATCACCCGACCCCGTACATCTGTAACGGTCACAATGGTATTGTTGAAACTTGCTTGAACATGAATAACTCCCTTTGGTATTCTACGTACATTTTTACGTGAACCACTACGGGTATTTTTACGTGAACCAATTCTTAATATAGGTTTTGCCATATTTTTTCATTTCACAAGAAATATATGGATATATCCATTTCATGTCAAAACGGACCTTTTTTTTACTAGCTCCTTGGAAGTGCCTTTTCCTTTAGTAAGATTATCCTTGTCTTTGTTTATGCCTCGGGTTGGAACAAATTACTATAATTCGTCCCCTCCTACGGATTAGCCGACACTTTTCACAAATTTTACGAACGGAAGCCCTTATTTTCATAGTTGTTATTCCTTAATTCTCTTAATATACTTATTGTTGGACGAAAAAAAGGTTTCTTGATATTTTTGAATCTTGAATTGTATCTTCGTGAAAGGAATGTTGAATTTCAAAAAACCACTGACTTATTTGAATCCTTGTTATGGAGTCTAGAAAGTGGCTGTTCCCCGATTAACTTAATACCTAAGAACTTACTAAAATTTTTACCCCTTTTTCTCCTATAGGTATACCTATACAAAAATATGTCGAATCCTTTCAGAAGCATGACCTAAAATAAAAAAAATCTTTAGTATCTAAACAAAATCGAACCATACCGTTCGGAAGTGATTCATAAAGAAAACTTTCATTAATTCATTTTTTTCTTTAATTTCATTCGGGGTAAAAAATTCTAAACTTTTTTAGCAGGGGTGGTATTACACAACCCCCCCCCTTTTTTTTCACAAATGCTAAGTTCCGGATATCCAATTTTGATATTAGAAGGATTACCATATATAACACAAAATTTCTCCGCCGATTCTTTTTAGTCGAGCTTCTCGATCTGTCATTATACCTTGAGAAGTTGAAAGGATTACAATTCCTATTCCGCCTAAAATTCGTGGAATTCGTTGAGAGTTAGAATAGATTCGTAGACCCGGTCGGCTTATTCTCTTTAAATTTAAAATCGTTTTATAGGATTCTTTCTTATTTCGTCTATGTCTTAGGGTTAAAATCAAAAAATATTGATTGTTTTCGCGATGTTTCCTTACGTTTTCGATAAAACCCTCTTGTAAAAGTATTTTAACAATGCTTTCGGTGATGTTAGTCGATCCTATCCGAACTGTTCCTTTTCTATTCATGTCAGCATTTCGTATAGAGGTTATTATATCAGCAATAGTGTCTTTCCCCATGATAAGTTAAAATTCCTTAATTGTTCTATAATTTTGATATAATCAACATGTTATTTTTCTTTTATTTATATAAAAATAGAGACGAATTATATATTAATATATGAATTCAATTATTAATATATAAAATTATTAAGGGTATATGCGTGATACACAATCTATTAATTATAATTAATTTGATTTCAATACCATTTTTTTAATCCTATCCTATATTAACTATCGATATTTAGGTCTTATAATACTTCAGGAGCTAATGAAACTATTTTAGTAAAGTTTAATTGTCTCAATTCCCGTGGGATCGCCCCAAAAACGCGAGTTCCTTTTGGATTTCCTTCTTGATCAATGACAACTGCGGCATTGTCGTCATATCGTATTATCGTCCCATTCTTACGTTTGAGTTCTTTACAAGTACGTACAATTACAGCTCTGACCACTTCTGATCTTTCTAGAGTAGTATTTGGGATTGCTTCCTTGATTACAGCAACAATAACGTCACCAATATGAGCATAGCGGCGATTACTAGCTCCTATTATTCGAATACACATCAATTTTCTAGCCCCGCTGTTGTCTGCTACATTCAAATAGGTTTGTGGTTGAATCATATTTTTGTATCTCTTCTTTTAGTGCAAAGGACGAAGTAAAAAAAATATTGTTTGTCAAAAAAAAACTTATAATCTTTTTATCCTTAAATGTTATTTAGCTTTTTCATTCTATATTCCTATTCAGAAATAATGAATTGGGTTTTTATAGGCATTTTTGATGCCGCGATTGAAATAGCTTTTCTGGCTATATTTTCTGGTACACCACCCATTTCATAAAGGATTTTACCTGGTTTAACCACAGCTACCCAGTACTCTGGGGATCCTTTCCCAGAACCCATACGCGTTTCCGCGGGTCTTACTGTAACTGGCTTGTCTGGAAATATACGTACCCAAATTTTTCCACCACGTCGTATATTTCGTGTCATTGCTCGTCGCCCTGCTTCTATTTGTCTAGATGTAATCCAAGCGGGTTCAAGTGTTTGAAGAGCATATCTGCCAAAACAAATACGATTCCCACGAGAGGATATTCCTTTTAGTCTTCCTCGATGTTGTTTACGAAATTTGGTTCTTTTTGGGTTATAGTTGATGGGTTTTTTCTAAATGAGAAATTCCATCTCTACTGCAGAACTGGACGTGAGAGTTTCTTCTCATCCAGCTCCTCGCGAATAAAAGGATTAATTAAGATATAGATGTAGTTAATGATTAATCCTATTAATCATGGTATTTTTTTTTTATTTCATCTTATCTCTTCTAAATTTGTGTATGTCTTTTTTGAAATAGAATCAAAGATCAATTTTATTTCGATTTATTTAAAAATAACGTAATATCATCATTACAAATGTAATTTTTATTAGAGTTAGAATATTATAACAAATCCTTATTTTATTTTTTTCATTGTTTTTTTCATCTTTTATTACTGTTTTTATTTGAAAAAAAAACCAATTTTTCGCCGGCGAATATTTACTCTTTCAATATCTATTTAAGTTTGCTGTTTATCCCCCGAGGTCTCAGAATCAAAATCAGAAATCAGAATAGATAATAAAGTTTCTGGTTTATTCCGCCATCCTGTCCAATGAATTACTAAGATTTCTTGTTCACTAGAATCCTATATATTCATGGGTTCCGTCGTTCCCATCGCTTCTTGATTAATCATTAGGCCTGAATTCTACAATGGAGCTTTTACATGAAATTTTGAATTTCTTTTTTTTTTTTTGAGGCAATTTTCTCAGTTTTTATTGGCTCAAGGCTCTTAATTTTTTGTTTTCGGAACAGATTTATCTAATTATTATGAATGAATCTGTATTGATGCTTTATTACATTGCTTTTCTTACAGTGACCTCATAGATTTTCCAAATTGGAATCATATATCATTAATATTCAATTTTTTCGCTCTTTCTTTCATCCTTCCATTTATCCGCATACTTTTTGATTACCTTTCATAACTTAATAATCATCTTTCTTTATTCTTTTTTTTTAGTCAGTTGCTCCAATGATATGATCAGCCTATCATATCTTGACTAATTTTTTGGATCCAGATAATGCGAAGCAATGAGTTGCTTAGGTTATTTATTAATGCTGTAGTTATTAGTTGGTAAGTTCTTTTTTTTTTTATCGTAATCTAACCCTAAACCAACGAGTCACACACTAAGCATAGCAATTATATCAAAGGAGTTTTGATGGAAATGTTTATTCAACCTTATAGAATTGCTCATTTTTTTTTCTTAAACATAAAAAAAAAGACTACAAGTTTTTATTTTTATTTCTTTATAGTGTTATACTACATAGTTTTCGTTTTTTATCATTGGATAAAATGTAAAGACAAATAAAGTTTTTTTATTCTTCGTCTACGAATATCCAAATTTTTATTCCTAAAACCCCATAAATAGTTCGAACTGTATAGGAACAATAATCAATTTTAGCTTCAATTGTTTGTAAAGGAACTCTGCCTTCTCTGATCCATTCAACACGTGCAATTTCTTTTCCGTCGATACGTCCTGCAATTTGTACTTGAATTCCTTTTGTATTCGCCTGTTCAGTTAATTCAATAGCTTTTTTCATTGCTTTTCGAAAAGAAACGCGATTTTTTAATTGGCCAGCTATAAATTCTGCAAGAATATTAGGATGCCCATACGGATTGGAAATTCGGGTAATAGCAATGTTGAGTTTTCTATTGACACAATTAAGTTCTTTTTGAACATTCATCTGTAATTCTTCGATTCTTCGGGGTTTATCTTCAATTAATAATTTAGGAAATCCCATATAGATTATGATCTGAATGAGATCGATTCTTTTTTGAATTTCGATACGTGCAATTCCCTCCATACCAGAGGATATTCTTATATTTTTTTGGACATAATTTTTAATACAGTCTCGTATTTTTTTATCTTCTTCTAAACCTTCAGAATACTTTTTTGGTTGTGCAAACCAAATAGAATGATGACTTTGGGTTGTACCAAGTCTGAAACCAAGTGGATTTATTTTTTGTCCCATGGGCCTCCACTACTATATGTATCGTAACATGTTAGATTTATGTTTTCATTGCTGCATCCAGGTTTTTTTAAATACATTAAATATTCTTCATATTGTTGATATAAGGATATATCTTCCAATACGATAGTTATATGACAAGTGGATCTTTTTATTGGGTAACTCCGTCCTCGTGCCCGAGGTTTTAATTTTTTCACCGTATTTCCTTGATTCACTTCAGCTTTACTAATGACTAAATTGGTTTCTTTGAAACCCTTATTGTGACTAGCATTTGCTGCTGCAGAATAAACTAATTTAAAAATGGGATAACATCCTCGATACGGCATAAGTTCTAATATCATAAGTGCTTCTTCGTAGGAACGTCCACGGATCTGATCAATAACTCTCCGTGCTTTGTGGGCAGACATAGATATATATTGCCCTAAAGCATATACGGAAGTATATGATTTCTTCTTTTTCTTCTTTATCATAAGGTTTACCTCTCACTAAAAAAAAAAATTATATTCATTATTCATTTTTTTGAATTCATTTAATTAACGACGAGATCTATTATCATTTTTCGCGTGTCCTCTAAAATTTATAGTAGGTGAAAATTCTCCCAATTTATGTCCTACCATAAGGTCGATTATATAAACGGGTAAGTGTTCCCTTCCATTATGTATAGCGATAGTATGGCCAATCATTGTGGGTATAATAGTAGATGCCCGGGACCAAGTTATTATGATTTCTTTTTCCGCCTTTGTATTAAGCTTCTCTATTTTTCTTAATAAATGCTTTGCTACAAAAGGATTTTTTTTTAGTGAACGTGTCACAGTTAATTAACTCCTATTTTTTTTAAGACGAAGAAAGAAATTCGATTTTCTCTCCTATTTACTACGGCGACGAAGAATCAAAGTCTCACTATATTTTTTCCTTTTTCTAGTTCTTCTTCCAAGCGCAGGATAACCCCAGGGGGTTACGGGTTTTTTTCTACCAATTGGAGCCCTCCCTTCACCACCTCCATGGGGGTGGTCGACAGGGTTCATAACTACTCCTCTTACTACAGGACGTTTACCTAGCCAACATTTCGATCCGGCTCTACCCAAACTTTTCTGGTTTACCCCAACATTTCCCACTTGTCCGACTGTTGCTGAGCAGTTTTTGGATATCAAACGGACCTCTCCAGAAGGTAATTTTAATGTGGCCGATTTCCCCTCTTTTGCAATCAGTTTCGCTACAGCACCCGCTGCTCTAGCTAATTGTCCACCCTTTCCAAGTGTGATTTCTATATTATGTATGGCCGTGCCTAAGGGCATATCGGTTGAAGTAGATTCTTCTTTTTGATCAATCAAAACCCCTTCCCAAACTGTACAAGCTTCTTCCAAAGCATACGGCTTTCTGAATGTAGATGATGATATCTATACGGATGGATCTTATCTTATATATATCGTAGAATTCTTCTATATATGGTAGAAGTACCACACGAGTGGATATATAGGAATCAAAATCTGCCGAATAACTTATGTTATGATCTTCTACATCCTAGGTCTTCCCGTTCCGTCATCTGGCTTATGTTCTTCATGTAGCATTCAGACCGAATGACTCTATGAAATTACGTCGATACTTCCACATATTATGGGTAACGTAGGAGACATCTCTATTTTTCCCCGGGGGAATCTTTAGAATTACCACTGCTTAGCTTTCAATTCGCCTCTGACCATCAAATGAAATGTGAATAACCCGTCCTCCTCTCTTTGAAACAAGGGGCGCTTATGGTTCTGTCGGTGCTTGAAACAATTTTGTCTTCTCCATATTACTATATCTCTAGAGTCAATAATTTTATATGAGGAACTACTGAACTCAATCACTTGCTGCCGTTACTCTTCAGTTTTCTGTTGAGGTCTATCCTGCAGAGGTACTCAAATTGGATCAGTGATCGATTTCTAGGTTTTGTCGTAAACCTAATTGGTTACTTCCAATTACGTAAATCAAATAGTTCAAACCGCACTCAAAGGTAGGGCATTTCCCATTTTTATAGGAACTTCTGTACCAGAAACAATGGTATCTCCAATTATAGCCCCTCTGGGATGTAAAATATATCTCTTCTCACCATCCCCATAGTGTATGAGACAAATGTATGCATTTCGATTAGGGTCGTATTCTATGGTTACGATTCTACCATATATGTCTTTTGTATTTCGTCGAAAATCTATTTTACGGTATAGACGCTTATGACCTCCCCCTCTATGCCTTACGGTAATGATTCCTCTGGCATTACGACCTTTACCACAATGATGCTGCCCATAGATCAAATTATTTCGTGGATTGGATTTCACTTGACTGTCTACGGCTCCATTGCGTGTGCTCGGGGTAGAAGTTTTGTATAAATGTATCGCCATGCTATTAAGTATTTTGATTTAAGTTCTTTTCTTTCTAAGAGGTGGAATAGAATAACCCGGTTGAAGCGTAATGATCATACGTCTGTAATGCATTGTATGTCCCAGAATAGGTCCCATTCTTTTAACCTTTCCGGGGAGTCGATGACTATTCATAGCTATTACCTTGACACCAAAGAAGAGTTCGACCCAATGCTTTATTTCTGTCCTAGTTGATCCTGATTCGACATTAAAAGTATATTGATTTTTCCCCAATAACCGAATACTTTTGTCTGTAAATACTGCATATTTGATTCCATCCATAAATCGATTTTCTTCCCTATGAGTTCTAGTCTCAATAAGAATGCTAGTTCTTACTGTTCATATGTTATGTTATGATATGAATATACCACACCAATTCGTTATGTATAGATGATGAGAAGATTCCATTGATACAGAGCCAATTCCAATAGACTTATTGGAGGGTCCCATTGGCGTGCATCCAGTAGGAATTGAACCTACGAATTCGCCAATTATGAGTTGGGCGCTTTAACCATTCAGCCATGGATGCTTAGTGGGGATCCTCGTACATGGTGAATAACCAAATTCCAATTGAAATGAAATCTTTAGGATAAATCAATGCAATTTAGGAGGAATCAATGAAAGGACATCAATTCAAATCCTGGATTTTCGAATTGAGAGAAATAGTGAGAGAGATCAAGAATTCTCACTATTTCTTAGATTCATGGACCCAAATCAATTCAGTGGGATCTTTCATTCATATTTTTTTCCACCAAGAACGTTTTAGAAAACTCTTGGACCCTCGAATTTTTAGTATCCTACTTTTGCGCAATTCACAGGGTTCAACAAGCAATCGATATTTCACGATCAAGGGTGTAGTACTATTTGTAGTAGCGGCCCTTCTATATCGTATTAACAATCGAAATATGGTCGAAAGCAAAAATCTCTATTTGAAAGGGCTTCTTCCTATACCTATGAATTCCATTGGACCCAGAAATGATACATCGGAAGAATCTTTTGGGTCTTCCAATATCAATAGGTTGATTGTTTCGCTCCTGTATTTTACAAAAGGAAAAAAGATCTCTGAGAGCTGTTTCCGGGATCCGAAAGAGAGTACTCGGGTTCTCCCAATAACTAAAAAGTGTATCATGCCTGAATCTAACTGGAGTTCGCGGTGGTGGAGGAACTGGATCGGAAAAAAGAGGGATTTTTGTTGTAAGATATCTAATGAAACCGTCGCTGGAATTGATATCTCATTTAAAGAGAAAGATATCAAATATCTGGAGTTTCTTTTTGTATATTATATGGATGATCCGATCCGCAAGGGCCATGATTGGGAATTGTTTGATCGTCTTTCTCCGAATAAGAGGCGAAACATAATCAACTTGAATTCGGGACAGCTATTCGAAATCTTAGTGAAAGACTGGATTTGTTATCTCATGTTTGCTTTTCGTGAAAAAATACCAATTGAAGTGGAGGGTTTCTTCAAACAACAAGGAGCTGGGTCAACTATTCAATCAAATGATATTGAGCATGTTTCCCATCTCTTCTCGAGAAACAAGCGGGCTATTTCTTTGCAAAATTGTGCTCAATTTCATATGTGGCAATTCCACCAAGATCTCTTCGTTAGTTGGGGGAAGAATCCGCACGAATCGGATTTTTTGAGGAAAATATCGAGAGAGAATTGGATTTGGTTAGACAATGTGTGGTTGGTAAACAAGGATAGATTTTTTAGCAAGGTACGAAATGTATCGTCAAATATTCAATATGATTCTACAAGATCTAGTTTCGTTCAAGTAACGGATTCTAGCCAATTGAACGGATCTTCTGATCAATTCATAGATCCTTTCGATTCCATTAGTAATGAGGATTCGGAATATCACTATCACACATTGATCAATCAAAGAGAGATTCAACAACTAAAAGAAAGATCGATTCTTTGGGATCCTTCCTTTATTCAAACGGAAGGAAGAGAGATAGAATCAGACCGATTCCCTAAATACCTTTCTGGATATTCCTCAATGCCCCGGCTATTCACGGAACGTGAAAAGCGAATGAATAATCATCTGCTTCCGGAAGAAAGCGAAGAATTTTTTTGGAATTCTACAAGAGCCATTCGTTCTTTTTTCTCTGACAGATGGTCAGAACTTCATCTGGGTTCGAATCCTACTGAGAGGTCCACTAGGGATCAGAAATTGTTGAAGAAAGAACAAGATGTTTCTTTTGTCCCTTCCAGGCGATCGGAAAATAAAGAAATAGTTAATATATTCAAGATAATTACGTATTTACAAAATACCGTCTCAATTCATCCTATTTCATCAGATCTGGGATGTGATACGGTTCCGAAGGATGAACTGGATATGGACAGTTCCAATAAGATTTCATTCTTGAACAAAAATCCATTTTTTTATTTATTTCATCTATTCCATGAACGGAAGAGGGGGGGATACACGTTACGCCACGATTTTGAGTCAGAAGAGAGATTTCAAGAAATGGCAGATCTATTCACTCTATCAATAACCGAGCCGGATCTGGTGTATCATACGGGATTTGCCTTTTCTATTGATTCCTACGGATTGGATCAAAGACAATTCTTGAAGGAGGTTTTCAACTCCAGGGATGAATTGAAAAAGAAATCTTTATTGGTTCTACCTCCTATTTTTTATGAAGAAAATGAATCTTTTTATCGAAGGATCAGAAAAAATTGGGTCCGGATCTCCTGCGGGAATTTTTTTGAAGATCCAAAACCAAAAAGAGTGGTATTTGCTAGCAACAACATAATGGAGGCAGTCAATCAATATAGATTGATCCGAAATCTGATTCAAATCCAATTCCAATATAGTCCCTATGGGTACATAAGAAATGTATTGAATCGATTCTTTTTAATGAAGAGACCTGATCGCAACTTCGAATATGGAATTCAAAGGGATCTAATAGGAAATGATACTCTGAATCATAGAACTATAATGAAAGATACGATCAACCAACATTTATCGAATTTGAAAAAGAGTCAGAAGAAATGGTTCGATCCTCTTATTTTTCTTTCTCGAACCGAGAGATCCATAAATCGGGATCCTAATGCATATAGATACAAATGGTCCAATGGGAGCAAGAATTTCCAGGAGCATTTGAAACATTTCGTTTCTGAGCGGAAGAGCCGTTTTCAAGTAGTGTTCGATCGATTATGTATTAATCAATATTCGATTGATTGGTCTGAGGTTATTGATAAAAAAGATTTGTCTAAGTCACTTCGTTTCTTTTTGTCCAAGTTACTTCGTTTTTTGTCCAAGTTACTTCTCTTTTTGTCTAATTCACTTCCTTTTTTCTTTGTGAGTTTCGAGAATATCCCCATTCATAGGTCTGAGATCCACATCTATGAATTGAAAGGTCCGAACGATCAACCCTGCAATCAGTTGTTAGAATCAATAGGTCTTCAAATCGTTCATTTTAAAAAATTGAAACCCTTTTTATTGGATGATCATAATACTTCTCAAAAATCGAAATTCTTGATCAATGGAGGAACAATATCACCATTTTTGTTCAATAAGATACCAAAGTGGATGATTGACTCATTCCATACTAGAAAGAATCGCAGGAAATCTTTTGATAACACGGATTCCTATTTCTCAATCGTATCCCACGATCAAGACAATTGGCTGAATCCCGTGAAACCATTTCAGAGAAGTTCATTGATATCTTCTTTTTCTAAAGCAAATCGACTTCGATTCTTGAATAATCCACATCACTTCTGCTTCTATTGTAACAAAAGATTCCCTTTTTATGTGGAAAAGGCCCGTCTCAATAATTCTGATTTTACGTATGGACAATTCCTCACTATCTTGTTCATTCACAACAAAATATTTTCTTCGTGTGGTGGTAAAAAAAAACATGCTTTTTTGGAGAGAGATACTATTTCACCTTCGTCAATCGAGTCACAGGTATCTAACATATTCATATCTAACGATTTTCCACAAAGTGGTGACGAAAGGTATAACTTGTACAAATCTTTCCATTTTCCAATTCGATCCGATCCATTAGTTCGTAGAGCTATTTACTCGATTGCAGACATTTCTGGAACACCTCTAATAGAGGGACAAAGAGTAAATTTGGAAAGAACGTATTGTCAAACTCTTTCAGATATGAATCTATCCGATTCAGAAGAGAAGAGCTTGCATCAGTATCTCAATTTCAATTCAAACGTGGGTTTGATTCACACTCCATGTTCTGAGAAATATTTACAGAGGAAAAAACGGAGTCTTTGCCTAAAAAAATGCGTTGACAAAGGGCAGATGGATAGAACCTTTCAACGAGATAGTGCTTTTTCAACTCTCTCAAAATGGAATCTATTCCAAACATATATGCCATGGTTCTTTACTTCGACAGGGTACAAATATCTAAATTTGATATTTTTAGATATTTTTTCAGACCTATTGCGGATACTAAGTAGCAGTCAAAAATTTGTATCCATTTTTCATGATATTATGTATGGATTAGATATATCATGGCGAATTCTTCAGAAAAAATTGTGTCTTCCACAAAGGAATCTGATAAGTGAGATTTCGAGTAAGTCTTTACATAATCTTCTTCTGTCCGAAGAAATGATTCATCGAAATAATGAGTCATCGTTGATATCGACACATCTGAGATCGCCAAATGTTCGTGAGGTCCTCTATTCAATCCTTTTCCTTCTTCTTGTTGCTGGATATATCGTTCGTACACATCTTCTCTTTGTTTCCCGAGCCTATAGTGAGTTACAGACAGAGTTCGAAAAGATCAAATCTTTGATGATTCCATCATACATGATTGAGTTGCGAAAACTTCTGGATAGGTATCCTACATCTGAACAGAATTCTTTCTGGTTAAAGAATCTTTTTCTAGTTGCTCTGGAACAATTAGGAGATTGTCTAGAAGAAATACGGGGTTCTGGCGGCAACATGCTATGGGGTGGTGATCCCGCTTATGGGGTCAAATCAATACGTTCTAAGAAGACAGATTTGAAAATAAACTTCATCGATATCATCGATCTCATAAGTATCATACCAAATCCCATCAATCGAATCACTTTTTCGAGAAATACGAGACATCTAAGTCATACAAGTAAAGACATCTATTCATTGATAAGAAAAAGAAAAAACGTGAGCGGTGATTGGATTGATGATAAAATAGAATCCTGGGTCGCGAACAGTGATTCGATTGATGATAAAGAAAGAGAATTCTTGGTTCAGTTCTCCACCTTAAGGGCAGAAAAAAGGATTGATCAAATTCTATTGAGTCTGACTCATAGTGATCATTTATCAAAGAATGACTCTGGTTATCAAATGATTGAACAACCGGGAACAATTTACTTACGATACTTAGTTGACATTCATAAAAAGTATCTAATGAATTATGAGTTCAATACATCCTGTTTAGCAGAAAGACGGATATTCCTTGCTCATTATCAGACAATCACTTATTCACAAACTTCGTGTGGGGCTAATAGTTTTCATTTCCCGTCTCATGGAAAACCCTTTTCGCTCCGCTTAGCCCTATCCCCCTCTAGGAGTATTTTAGTGATAGGTTCTATAGGAACCGGACGATCCTATTTGGTCAAATACCTAGCGACAAACTCCTATGTTCCTTTCATTACAGTATGTCTGAACAAGTTCCTGGATAACAAGCCGAAAGGTTTTTTTCTTGATGATATCGATATTGATGATAGTGACGATATTGATGCTAGTAACGATATTGATCGTGAACTTGATACGGAGCTGGAGCTTCTAACTATGATGAATGCGCTAACTATGGATATGATGTCGGAAATAGACCGATTTTATATCACCCTTCAATTCGAATTAGCAAAAGCAATGTCTCCTTGCATAATATGGATTCCAAACATTCATGATCTTGATGTGAATGAGTCGAATTACTTAGCCCTCGGTCTCTTGGTGAACTCTCTCTCCAGGGATTGTGAAAGATGTTCGACTAGAAATAGTCTTGTTATTGCTTCGACTCATATTCCCCAAAAAGTGGATCCCGCTCTAATAGCCCCGAATAAATTAAATACATGCATTAAAATAAGAAGGCTTCTTATTCCACAACAACGAAAGCACTTTTTCACTCTTTCCTATACTAGGGGATTTCACTTGGAAAAGAAAATGTTCCATACTAATGGATTCGAGTCCATAACCATGGGTTCCAGTGCACGAGATCTTGTAGCACTTACCAATGAGGCCTTATCAATTAGTATTACACAGAAGAAATCAATTATAGACACTAATACAATTAGATCTGCTCTTCATAGACAAACTTGGGATTTGCGATCCCAGGTAAGATCGGTTCAGGATCATGGGATCCTTTTCTATCAGATAGGAAGGGTTGTTGCACAAAATGTACTTATAAGTAATTGCCCCATAGATCCTATATCTATCTATATGAAGAAGAAATCATGTAACGAAGGGGATTCTTATTTGTACAAATGGTACTTCGAACTTGGAACGAGCATGAAGAAATTCACGATACTTCTTTATCTTTTGAGTTGTTCTGCCGGATCGGTCGCTCAAGACCTTTGGTCTCTACCCGGACCCGATGAAAAAAATAGGATCACTTCTTATGGATTCATTGAGAATGATTCGGATCTATTTCATGGCCTATTAGAAGTGCAAGGCGCTTTGGTGGGATCCTCACGGACAGAAAAAGATTGCAGTCAGTTTGATAATGATCGAGTGACATTGCTTTTTCGCTCCGAACCAAGGGATCCCTTATATATGATGCAAGATGGATCTTGTTCTATCGTTGATCAGAGATTTCTCTATGAAAAATACGAATCGGAGTTTGAAGAAGGGGAAGGAGAAGCAGTCCTCGACCCGGAACAGATAGAGGAGGATTTATTCAATCACATAGTTTGGGCTCCTAGAATATGGCGCCCTCGGGGCTTTCTATTTGATTGTATCGAAAGGCCTAATGAATTGGGATTTCCCTATTTGGCCGGGTCATTTCGGGGCAAGCGGATCATTTATGATGAAAAGTATGAGCTTCAAGAGAATGATTCGGAGTTCTTGCAGAGCGGAACCATGCAGTACCAGAGACGAGATAGGTCTTCCAAAGAACAAGGCTTTTTTAGAATAAGCCAATTCATTTGGGACCCCGCAGATCCACTCTTTTTCCTATTCAAAGATCAGCCCTTTGTCTCTGTGTTTTCACATCGAGAATTCTTTGCAGATGAAGAGATGTCAAAGGGGCTTCTTACTTCCCAAACAGATCCTCCTACATCTATATATAAACGCTGGTTTATCAAGAATACGCAAGAAAAGCACTTCGAATTGTTGATTCAGCGCCAGAGATGGCTTAGAACCAATAGTTCATTATCTAATGGATTTTTCCGTTCTAATACTCTATCCGAGAGTTATCAGTATTTATCAAATCTGTTCCTATCTAACGGAACGCTAGTGGATCGAATGACAAAGACATTGTTGAAAAAAAGATGGCTTTTTCCGGATGAAATGAAAATAGGATTCATGTAATGTAACAGGAGAAAGGTTTCCCATTACTTAGCCGGAAAGATATGTGTCCATGAAATAGGGATTAAGTGGAACGGAATTGACTGGGTGGTAGAGTTGTAGAAACACCTGTTTCTTCCACTTAGCTCCATGGAACAATATGCTACGACGGAAACATGGAAGAATTGAAATCTTAGATCAAAACACTATGTATGGATGGTACGAACTGCCTAAACAAGAATTCTTGAACAGCGAACAACCAGAGCTATTACTCACTACATCAAAAAAATTTCCATTAATGAAGGATGGAAATCCATTGGAAAATCAAAAATACGCATGTCGGATGAAATTGTTGTTGCTATCTGTTCCAATAACGAATCAACTGAATAACTAAATAAAATAGATAGACCTTTCTCTTCGTCTCAGGTCGATAGATCTTCTCAATTGGAAGATCCCCTATATGGATAATACACATTCCAGTTGACCGAGCCTAATTCTAATTGTTTTGTTCCGAAGTAAAGATATCCACGGAGTGGTTCGCCCTATTCAGATATTCACGACCAAGAAGTACTGGATTCTGTTTAGGATAGGTCCTGAAAGGAGAAGGAAGGCTGGAATGCCGCCAGGCGTCTATTATTGAATTCACCCGACCCGATAGTACCAATTTTGGTAACGTCCATCCAGTGCCAAAGTCACTGAATGGGTAAGTCACCAATCCCTAAAACGGACTATGTACTTTATCTGCTGGGTTACGGGGGCATTTTACCAGAGGTTTAGATTGTATCAATCTACCCTTGTGTGATTCCTGTTGAATCATATACTGCGGGGCGCAGGGCGGACGATTTCAAAGCGGACTCCCCCTCCCCATTCATTAGATAGAGAAGATCGCCAAGATTTCGCGATCCGCTGCCGAACTTATTCCATTTCAATATTATGCCTTGAAGAGGACTCGAACCTCCACGCTTTTTAGCACGAGATTTTGAGTCTCGCGTGTCTACCATTTCACCACCAAGGCATCTTGAAAGTGAATCGTATTCCATAAATATGATATCTATCTAGTACGGTGTATTGAATATATGACAAAGGTGGAGTGTTGAAGTATTTCTATTGATCGGTCATGTCATATAGGCCCGAGTCGGACATCTAATTGCTTAGATTTTAATTATCCTTATCCGGAGGATGCCTTATATATATATTAATATTATATCAAAAAGATGGACAATCAAACCTATTTCTCGATTCAATAGAAGTCCAACCAAAGAGGTGAATAGGGTCCCAAATAACGAGAGATATGTAAAAAGTAGGTCAGATTTCGCCTATTCCTAATCCTAAATGGAATGTAACGACGTAGGGATCCCTATGTAAACATAGTATCTATTTAGATACGCTCGAATGACCCCTTCTCATAATGAGAATGTATATAACCTTATTCCGGTCTGGTCCGGTATGGAATGAACTTATAATCATGGAATCGACTCGATCATCAGATTATAGATTATAAGTTCATAACCTTAGTCCATTCCCATTTTGGGCGGAACCGATCTACTAATTCTTTGATTCCAGTTAGTAAGAGGGATCTTGAACTAAGAAATAGATTCTAGAAGCTAAAAAGGGTATCCTGAGCAATCGCAATAATCGGGTTCATTGATATTCCTGGTATAGTAGATGCTATCACACATACAATCATACTCAATTCGATGGAATTGGTTGATCTTAAAGGGGATATTCTATAATTTCGCACGTGAGGGGTTATTTCTTGGTTTCGTCCAGTCATTAATAACTTGATTATTTTTAGATAATAGTAGATAGAAAGAACGCTCGTAAGGAGTCCTATTGAAACCAAGAAATATAGGCCTGCCCGCCATCCACACCAGAATAAATGGAGTTTTCCAAAAAAACCTGCTAGTGGAGGAAGACCTCCTAGGGATAAGAGACATAGAGCTAAAGAGAGAGCCAAAAAAGGATCTTTTGTGTATAATCCTGCATAATCTCGAATGTTATCAGTTCCGGTACGTAGACCAAATAATATAATGCAAGCAAAAGTTCCTAGATTCATGGAGATATAGAACAGCATATAAGTTATCATGCTCGCATATCCACCATTTGAGTCTCCAACAATTATTCCAATAATTACATATCCGATTTGACCTATGGACGAATATGCAAGCATACGTTTCATGCTTGTTTGAGTAATAGCAATGAGATTCCCCAATATCATGCTAAGAATAGCTAGGATTTCCAGAAGAAGATGCCATTCATTTGATGAGAAATAAAAAGGAATATCGAAAATTCGAGTGGCTAAAGCTGAAGCAGCTACTTTCGAAGTAACAGAAAGAAAAGCAACGACTGGAGTGGGAGAGTCAGAGTCGAAAAGAGGATTCCTCACTTCTTTCTCTCATTCAAAACCGTGCATGAGACTTTCATCTCGCACGGCTCCTAAGTGATAAAAGTAAAGAAGAACTCATCTTCTTTCTTTTTTGATTACTTTCCTCGCGTATGTATAAGATCGAATCCTTTCTAAAACGGATGACTAATCCTTAACTTTTCGAGGAATCCTTCATCAGTGGTTGTGAATGACTGATTTTTCTCAATCGTTTCGACCTTGGTTCCGTAGGAGCACGTCCGAAAGATTGAGAAATGGAACCATCTGATTTGATTCGTTCTCAATAGCCATGAGATGATCATCTTAGGGTGATCCTTTTGTCGACGGATGCTCCTATTACACTCGTAGTCTCTGAAGGATGAGAACCAACTATGTAGCATCTACATCGAGAATTCAAGTCTTTCTTGTATACGTCATTAGTCCGATCCTTTGTAGGAACTACCCGTAATAACAAACTTGCAAAATGGATCCGTTTATCATAAAGAGATTCGTTGTTCCTGACCCTGCTTCACATTAATTGTTATTTGAACAAGTCAAAGTTCTGTCTTGGTCTGCGTGGGGATAGCATTTCTCTTCTGCATGTCCATGGAGTTTTGAAAAATCCAAACATCTCAGAGATAGATAGAGAGGTAGGAATTTCTCAAACGAACCGCACTCCTTCGTATACGTCAGGAGTCCATTGATGAGAAGGGGCTAGGGAAAGCTTGAACCCAATTCCTACAGTGATGAATATAAGCGCAATTGAAATTCCTGGGGAGTTATACATTTGTGTATTGATAAGACCATTCACTATTTCTTGAAGCTCAATCTCTCCCCCGGATGAACCATATAGCCAAGAGAAACCATGAACCAGAATAGAAGAGCTTGCCCCACCCATGAGTAAATATTTCATAGTAGCTTCATTAGATCGTACATCTTTCTTGGTATATCCAGATAATAGGTAGGAGCATAAACTGAAACATTCTGGAGCTACAAAGATAGTTATTAAATCGTTAGCACCACATAAAAACATTCCTCCTAGAGTAGCTGTTAATACGAATAACAGAAACTCTGTTATAGCCATTTCTGTACATTCAATGTACTCTACGGATAGAGGAATACAGAGAGTTGAACATAGTAAAATAAGAAATTGAAAGATTTCGTTGAAATTGTTCGTTTGGAAATTTCCTGAAAAGCTAATCATAGGTTCTTCTCTCCATCGGAACAATAGGGCCGTTATGCTCATTACGAAACTTGTTGACGAGATGAAATATAACCAAGGTATATCTTTTTGATCAGAGGTTGAATCGATCATCAGAAGAAGGATTAGGCCAAAAATTAGGATACATTCTGGGAAAATAAAACTTCCATCGAAGAGAAGCAAATGAAAGGCTTTCATAAAAATTCTCGTAGAATCGAGAATGAAATTTTCATTCTGTACATGCCAGATCATGAATTAGTAACTGCATCCAATCTCCAAAAAAAAACCAATTTTTTTTTTTTTTGAATGGAATATTTACGGAATCCCCATGAATAGGTTAAAACCTTATTCCATGGTATTTACATGAGATTGCTCTTTCTTATTCTTAAGCAAGTCCCCGAGAGGGCTTAGTTGATCCATGATTTATGTTTCGTCTTTTCTTTCCTTTTCGTTTGTTTCGAGAAAGAGATCGATCAATTCCGATTTTTTCTTTTTCTATTGATTCTTTTCGGATCGAGATGTATGGATCCACGGATCTATGTGTCTATATAGATCCTGTTCATGGATTAACGAAAATGTGCAAACGCTCTATTTGCCTCTGCCATTCTATGAGTCTCTTCCTTTTTGCGTATGGCATCGCCACTCCCTTTGGCAGCATCCACTAATTCGGAACTTAATTTGAAAGCCATATTTCGACCCGGACGTTTTCGGGATGCCCCTAATAACCAACGAATGGCAAGTGCTTTTCCTTGCGTGGATCCTATTTCAATGGGAACTTGATGAGTTGATCCGCCTACACGTCTTGCTTTTACTGCTATATCGGGAGTTACTCCACGTATTGCTTGACGTAAAACAGATAGTGGATTTGTTTCTGTCTTTTGTTGAATCTTTTTCAAGGCTCGATAGATAATTTGATAAGCCAATGATTTTTTTCCGTGTTTCAGAATACGGTTAACCAACATGTTAACTAATCGATTACGATAAATTGGATCGGATTTTGCAGTTTTTTCTTCTGCAGTACCTCGACGTGACATGAGCGTGAAAGGGGTTCAAGAATCTGTTTTCTTTTTATAAGGGCTCAAATCTTTTATTTTGGCTTTTTGACCCCATATTGTAGGGTGGATCTCGAAAGATATGAAAGATCTCCCTCCAAACCGTACATACGACTTTCATCGAATACGGCTTTCCACAGAATTCTATATGTATCTATGAAATCGAGTATGGAATTCTGTTTACTCACTTTTAAATTGAGTATCCGTTTCCCTCCTTTTCCTGCTAGGATTGGAAATCCTGTATTTTACATATCCATACGATTGAGTCCTTGGGTTTCCGAAATAGTGTAAAAATAAGTGCTTCGAATCATTGCTATTTGACCCGGACCTGTTCTAAAAAAGTCGAGGCATTTCGAATTGTTTGTTGACACGGACAAAGTCAGGGAAAACCTCTGAAATTATTTCAATATTGAACCTTGGACATATAAGAGTTCCGAATTGAATCTCTTTTGAAAGAAGATCTTTTGTCTCATGGTAGCCTGCTTCAGTCCCCTTACGAAACTTTCGTTATTGGGTTAGCCATACACTTCACATGTTTCTAGCGATTCACATGGCATCATCAAATGATACAAGTCTTGGATAAGAATCTACAACGCACTAGAACGCCCTTGTTGACGATCCTTTACTCCGACAGCATCTAGGGTTCCTCGAACAATGTGATATCTCACACCGGGTAAATCCTTAACCCTTCCCCCTCTTACTAAGACTACAGAATGTTCTTGTAAATTATGGCCAATACCAGGTATATAAGCAGTGATTTCAAATCCCGAGGTTAATCGTACTCTGGCAACTTTACGTAAGGCAGAGTTTGGTTTTTTGGGGGTGATAGTGGAAAAGTTGACAGATAAGTCACCCTTACTGCCACTCTACAGAACCGGACATGAGATTTTCACCTCATACGGCTCCTCGTTCAATTCTTTCGAAGTCATTGGGTCCCTTTCCTCGTTCGCGAATCTCCTCCGTCCCGAAGAGTAACTAGGATAAACTCGGTCACGTTTTCATGTTCCAATTGAACACTTTCTATTTTTGATTATTCTCAAAGGATAAGATTATTCTTTTTACCAAACATCTGCGGGTCCAATCACACGATCTTATAATAAGAACAAGAGATCTTTCTCGATCAATCTCTTTGCCCCTCATTCTTCGAGAATCAGAAAGAGACTTTTTCAAGTTTGAATTTGTTCATTTGTAATCTGGGTTCTTCTACTTCATTTTTATTTACTTATTATTTCTTTATTTTCCCTCTCTTTTCTTTATTTGATTTCTTTTTTGATTTTATTCCCTTCCATCATTCTTAAGTCCCATAGGTTTGATCCTATAGAATCTGACCCATGTTCTCATTGAGCGAAGGGTACGAAATAAATTCAATCATATTTTTTTTTGATCAAAAAAAAATCACTATGTGAAATCTTCGTTTTTTTTTTTCTCTTTCTCTATCGCTTTCCCATAAGTACAGCACTTGTTGAATCGATAGAGAACCTTTTCTTCTGTATCGATATGAATCCATTATGAATCGATATTATTACATTCCAATTCCTTACCAATATCCCTCAAGGAAAATCCCGAATTGGATCCCAAATTGACGGGTTAGTGTGAGCTTATCCATGCGGTTATGCACTCTTCGAATAGGAATTTATTTTCTGAAAGATCCTGGCTTTCGTGCTTTGGCGGGTCTCCGAGATCCTTTCGACGACCTATGTTGTGTTGAAGGGATATCTAGATGATCCGATCGATTGCGTAAAGCCCGCAGTAGCAACGGAACCGGGGAAAGTATACATAAGTATACAGAAAAGACAGTTCTTTTCTATTATATTAGGATTTTCTATTCTATTAGATTAGTGTTAGTTAGTGATCTTGGCGCAGTGAGTCCTTTCTTCTCGGTCCACAGAGACAAAATGTAGGACTGGTGCCAACAGTTAATCACGGAAGAAAGGAGGCTCAGCGGGAAGAGGATTGTACCATAGAAGCAAGGAGGTCAACCTCTTTCCAATAGATAACATGAATTCTGGCAATGCAATGTAGTTGGGCTTTCATGTTGATCCGAATGAATCATCTTTTTCGCGGAGTGAAATCTTTGCCTGCTAGGCAAGATTATAGGATAGCAAGTTACAAATTCTGTTTCGGTAGGACATGTATTTCTATTACTATGAAATTCATAAATGAAATAGTTAATCGTGGGGTTACCATTCTCTCTTTTTTTTTTTATCTCGCACGTGTTCCTAAGAAAAGGGAATTTGTTAATTTTTCGGGGTCTTAAAGGGGCGTGGAAACACATAAGAACTCTTGAATGGAAATGGAAAAGAGATGTAACTCCAGTTCCTTTGGAAATAGGAAGATCTTTGGCGCAAGAATAAAGGATTAATCCGTATCATCTTGACTTGGTTCTGATTTCTCTATTTTTTGAAGTTTAAGAAAAGAATACCGTTTCTCCTACCCGTATCGAATAGAACATGCTGAGTAAAATCTTCTTCATGTAAAACCGGCTTGATTTAGATCGGGAGAATCGTACGGTTTTATGAAACCATGTGCTATGGCTCGAATCCGTAGTCAATCCTATTTCCGATAGGAGTAGTTGACAATTGAATCCAACTTTTTCCATTATTTTCATTTCATACCCGTAATAGTGCGAAAGGAAAGCCCGGCTCCAATCCAAGTTGTTCAAGAATAGTGGCCTTGAGTTTCTCGACCCTTTGACTTAGGATTAGTCAGTTCTATTTCTTGATGGGGGAAGGGATATAACTCAGCGGTAGAGTGTCACCTTGACGTGGTGGAAGTCATCAGTTCGAGCCTGATTATCCCTAAACCCAATGAATGTGAGTTTTTCTATTTTGACTTGCTCCCTCGCTGTGATCGAATAAGAATGGATAAGAGGCTCGTGGGATTGACGTGAGGGGGTAGGGGTAGCTATATTTCTGGGAGCGAACTCCATGCGAATATGAAGCGCATGGATACAAGTTATGACTTGGAATGAAAGACAATTCCGAATCAGCTTTGTCTACGAAGAAGGAAGCTATAAGTAATGCAACTATGAATCTCATGGAGAGTTCGATCCTGGCTCAGGATGAACGCTGGCGGCATGCTTAACACATGCAAGTCGGACGGGAAGTGGTGTTTCCAGTGGCGGACGGGTGAGTAACGCGTAAGAACCTGCCCTTGGGAGGGGAACAACAGCTGGAAACGGCTGCTAATACCCCGTAGGCTGAGGAGCAAAAGGAGGAATCCGCCCGAGGAGGGGCTCGCGTCTGATTAGCTAGTTGGTGAGGCAATAGCTTACCAAGGCGATGATCAGTAGCTGGTCCGAGAGGATGATCAGCCACACTGGGACTGAGACACGGCCCAGACTCCTACGGGAGGCAGCAGTGGGGAATTTTCCGCAATGGGCGAAAGCCTGACGGAGCAATGCCGCGTGGAGGTAGAAGGCCTACGGGTCCTGAACTTCTTTTCCCAGAGAAGAAGCAATGACGGTATCTGGGGAATAAGCATCGGCTAACTCTGTGCCAGCAGCCGCGGTAATACAGAGGATGCAAGCGTTATCCGGAATGATTGGGCGTAAAGCGTCTGTAGGTGGCTTTTTAAGTCCGCCGTCAAATCCCAGGGCTCAACCCTGGACAGGCGGTGGAAACTACCAAGCTTGAGTACGGTAGGGGCAGAGGGAATTTCCGGTGGAGCGGTGAAATGCGTAGAGATCGGAAAGAACACCAACGGCGAAAGCACTCTGCTGGGCCGACACTGACACTGAGAGACGAAAGCTAGGGGAGCGAATGGGATTAGATACCCCAGTAGTCCTAGCCGTAAACGATGGATACTAGGCGCTGTGCGTATCGACCCGTGCAGTGCTGTAGCTAACGCGTTAAGTATCCCGCCTGGGGAGTACGTTCGCAAGAATGAAACTCAAAGGAATTGACGGGGGCCCGCACAAGCGGTGGAGCATGTGGTTTAATTCGATGCAAAGCGAAGAACCTTACCAGGGCTTGACATGCCGCGAATCCTCTTGAAAGAGAGGGGTGCCTTCGGGAACGCGGACACAGGTGGTGCATGGCTGTCGTCAGCTCGTGCCGTAAGGTGTTGGGTTAAGTCCCGCAACGAGCGCAACCCTCGTGTTTAGTTGCCACCGTTGAGTTTGGAACCCTGAACAGACTGCCGGTGATAAGCCGGAGGAAGGTGAGGATGACGTCAAGTCATCATGCCCCTTATGCCCTGGGCGACACACGTGCTACAATGGCCGGGACAAAGGGTCGCGATCCCGCGAGGGTGAGCTAACTCCAAAAACCCGTCCTCAGTTCGGATTGCAGGCTGCAACTCGCCTGCATGAAGCCGGAATCGCTAGTAATCGCCGGTCAGCCATACGGCGGTGAATTCGTTCCCGGGCCTTGTACACACCGCCCGTCACACTATGGGAGCTGGCCATGCCCGAAGTCGTTACCTTAACCGCAAGGAGGGGGGTGCCGAAGGCAGGGCTAGTGACTGGAGTGAAGTCGTAACAAGGTAGCCGTACTGGAAGGTGCGGCTGGATCACCTCCTTTTCAGGGAGAGCTAATGCTTCTTGGGTATTTAGGTTTGACACAGCTTCAAACCCAAAGCCCATGAGCTTATTATCCTAGGTCGGAACAAGTTGATAGGATCCCCTTTTACGCCCCCATGTCCCTCTCGTGTGGCGGCAGGGGGGCGTAAAAAGGAAAGAGAGGGATGGGGTTTCTCTCGCTTTTGGCTTGGCATAGCGGGCCCCCAGCAGGAGGCCCGCACGACGGGCTATTAGCTCAGTGGTAGAGCGCGCCCCTGATAATTGCGTCGTTGTGCCTGGGCTGTGAGGGCTCTCAGCCACATGGATAGTTCAATGTGCTCATCAGCGCCTGACCCTGAGATGTGGATCATCCAAGGCACATTAGCATGGCGTACTCCTCCTGTTCGAACCGGGGTTTGAAACCAAACTTCTCCTCAGGAGGATAGATGGGGCGATTCAGGTGAGATCCAATGTAGATCCAACTTTCTATTCACTCGTGGGATCCGGGCGGTCCGGAGGGGACCACCACGGCTCCTCTCTTCTCGAGAATCCATACATCCCTTATCAGTGTATGGACAGCTATCTCTCGAGCGCAGGTTTAGGTTCGGCCTCAATGGGAAAATAAAATGGAGCACCTAACAACGTATCTTCACAGACCAAGAACTACGAGATCACCCCTTTCATTCTGGGGTGACGGAGGGATCGTACCGTTCGAGCCTTTTTTTCATGCTTTTCCCAGGGGTCTGGAGAAAGCTGCAATCAATAGGATTTTCCTAATCCTCCCTTCCCGAAAGGAAGAACGTGAAATTCTTTTTCCTTTCCGCCTCGAAATGGGAGCAGGTTTGAAAAAGGATCTTAGAGTGTCTAGGGTTAGGCCAGTAGGGTCTCTTAACGCCCTCTTTTTTCTTCTCATCGAAGTTATTTCACAAATACTTCCTATGGTAAGGAAGAGGGGGGGAACAAGCACACTTGGAGAGCGCAGTACAACGGAGAGTTGTATGCTGCGTTCGGGAAGGATGAATCGCTCCCGAAAAGGAATCTATTGATTCTCTCCCAATTGGTTGGACCATAGGTGCGATGATTTACTTCACGGGCGAGGTCTCTGGTTCAAATCCAGGATGGCCCAGCTGCGCCAAGGAAAAGAATATAAGAAGGATCTGACTCCTTCATGCATGCTCCACTTGGCTCGGGGGGATATAGCTCAGTTGGTAGAGCTCCGCTCTTGCAATTGGGTCGTTGCGATTACGGGTTGGGTGTCTAATTGTCCAGGCGGTAATGATAGTATCTTGTACCTGAACCGGTGGCTCACTTTTTCTAAGTAATGGGGAAAAGGACCGAAACATGCCACTGAAAGACTCTACTGAGACAAAGATGGGCTGTCAAGAACGTAGAGGAGGTAGGATGGTCAGTTGGTCAGATCTAGTATGGATCGTACATGGACGGTAGTTGGAGTCGGCGGCTCTCCTAGGGTTCCCTCGTCTGGGATTGATCCCTGGGGAAGAGGATCAAGTTGGCCCTTGCGAACAGCTTGATGCACTATCTCCCTTCAACCCTTTGAGCGAAATGCGGCAAAAGGAAGGAAAATCCATGGACCGACCCCATCGTCTCCACCCCGTAGGAACTACGAGATCACCCCAAGGACGCCTTCGGTATCCAGGGGTCGCGGACCGACCATAGAACCCTGTTCAATAAGTGGAATGCATTAGCTGTCCGCTCGCAGGTTGGGCAGTAAGGGTCGGAGAAGGGCAATCACTCATTCTTAAAACCAGCATTCGAAAGAGTTGGGGCGGAAAAGGGGGGAAAGCTCTCCGTTCCTGGTTCTCCTGTAGCTGGATCCTCTCGAACCACAAGAATCCTGAGTTGGAATGGGATTCCAACTCATCACCTTTTGAGATTTTGAGAAGAGTTGCTCTTTGGAGAGCACAGTACGATGAAAGTTGTAAGCTGTGTTCGGGGGGGAGTTCTTGTCTATCGTTGGCCTCTATGGTAGAATCAGTCAGGGGCCTGATAGGCGGTGGTTTACCCTGTGGCGGATGTCAGCGGTTCGAGTCCGCTTATCTCCAACTCGTGAACTTAGCCGATACAAAGCTATATGATAGCACCCAATTTTTCCGATTCGGCAGTTCGATCTATTATTTTTCATTCATGGACGTTGATAAGATCTTTCCATTTAGCAGCACCTTAGGATGGCATAGCCTTAAAGTTAAGAGCGAGGTTCAAACGAGGAAAGGCTTACGGTGGATACCTAGGCACCCAGAGACGAGGAAGGGCGTAGTAAGCGACGAAATGCTTCGGGGAGTTGAAAATAAGCGTAGATCCGGAGATTCCCGAATAGGTTAACCTTTTGAACTGCTGCTGAATCCATGGGCAGGCAAGAGACAACCTGGCGAACTGAAACATCTTAGTAGCCAGAGGAAAAGAAAGCAAAAGCGATTCCCGTAGTAGCGGCGAGCGAAATGGGAGCAGCCTAAACCGTGAAAACGGGGTTGTGGGAGAGCAATAAAAGCGTCGTGCTGCTAGGCGAAGCGGTGGAGTGCCGCACCCTAGATGGCGAGAGTCCAGTAGCCGAAAGCATCACTAGCTTATGCTCTGACCCGAGTAGCATGGGGCACGTGGAATCCCGTGTGAATCAGCAAGGACCACCTTGCAAGGCTAAATACTCCTGGGTGACCGATAGCGAAGTAGTACCGTGAGGGAAGGGTGAAAAGAACCCCCATCGGGGAGTGAAATAGAACATGAAACCGTAAGCTCCCAAGCAGTGGGAGGAGCCCTGGGCTCTGACCGCGTGCCTGTTGAAGAATGAGCCGGCGACTCATAGGCAGTGGCTTGGTTAAGGGAACCCACCGGAGCCGTAGCGAAAGCGAGTCTTCATAGGGCAATTGTCACTGCTTATGGACCCGAACCTGGGTGATCTATCTATGACCAGGATGAAGCTTGGGTGAAACTAAGTGGAGGTCCGAACCGACTGATGTTGAAAAATCAGCGGATGAGTTGTGGTTAGGGGTGAAATGCCACTCGAACCCAGAGCTAGCTGGTTCTCCCCGAAATGCGTTGAGGCGCAGCAGTTGACTGGACATCTAGGGGTAAAGCACTGTTTCGGTGCGGGCCGCGAGAGCGGTACCAAATCGAGGCAAACTCTGAATACTAGATATGACCTCAAAATAACTGGGGTCAAGGTCGGCCAGTGAGACGGTGGGGGATAAGCTTCATCGTCGAGAGGGAAACAGCCCGGATCACCAGCTAAGGCCCCTAAATGACCGCTCAGTGATAAAGGAGGTAGGGGTGCAGAGACAGCCAGGAGGTTTGCCTAGAAGCAGCCACCCTTGAAAGAGTGCGTAATAGCTCACTGATCGAGCGCTCTTGCGCCGAAGATGAACGGGGCTAAGCGATCTGCCGAAGCTGTGGGATGTCAAAATGCATCGGTAGGGGAGCGTTCCGCCTTAGGGGGAAGCAACCGCGCGAGCGGCGGTGGACGAAGCGGAAGCGAGAATGTCGGCTTGAGTAACGCAAACATTGGTGAGAATCCAATGCCCCGAAAACCCAAGGGTTCCTCCGCAAGGTTCGTCCACGGAGGGTGAGTCAGGGCCTAAGATCAGGCCGAAAGGCGTAGTCGATGGACAACAGGTGAATATTCCTGTACTACCCCTTGTTGGTCCCGAGGGACGGAGGAGGCTAGGTTAGCCGAAAGATGGTTATCGGTTCAAGAACGCAAGGTGTCCCTGTTTTTTCAGGGTAAGAAGGGGTAGAGAAAATGCCCCGAGCCAATGTTCGAGTACCAGGCGCTACGGCGCTGAAGTAACCCATGCTATACTCCCAGGAAAAGCTCGAACGACCTTCAACAAAAGGGTACCTGTACCCGAAACCGACACAGGTGGGTAGGTAGAGAATACCTAGGGGCGCGAGACAACTCTCTCTAAGGAACTCGGCAAAATAGCCCCGTAACTTCGGGAGAAGGGGTGCCTCCTCACAAAGGGGGTCGCAGTGACCAGGCCCGGGCGACTGTTTACCAAAAACACAGGTCTCCGCAAAGTCGTAAGACCATGTATGGGGGCTGACGCCTGCCCAGTGCCGGAAGGTCAAGGAAGTTGGTGACCTGATGACAGGGGAGCCGGCGACCGAAGCCCCGGTGAACGGCGGCCGTAACTATAACGGTCCTAAGGTAGCGAAATTCCTTGTCGGGTAAGTTCCGACCCGCACGAAAGGCGTAACGATCTGGGCACTGTCTCGGAGAGAGGCTCGGTGAAATAGACATGTCTGTGAAGATGCGGACTACCTGCACCTGGACAGAAAGACCCTATGAAGCTTCACTGTTCCCTGGGATTGGCTTTGGGCTTTTCCTGCGCAGCTTAGGTGGAAGGCGAAGAAGGCCTCCTTCCGGGGGGGCCCGAGCCATCAGTGAGATACCACTCTGGAAGAGCTAGAATTCTAACCTTGTGTCAGGACCTACGGGCCAAGGGACAGTCTCAGGTAGACAGTTTCTATGGGGCGTAGGCCTCCCAAAAGGTAACGGAGGCGTGCAAAGGTTTCCTCGGGCCGGACGGAGATTGGCCCTCGAGTGCAAAGGCAGAAGGGAGCTTGACTGCAAGACCCACCCGTCGAGCAGGGACGAAAGTCGGCCTTAGTGATCCGACGGTGCCGAGTGGAAGGGCCGTCGCTCAACGGATAAAAGTTACTCTAGGGATAACAGGCTGATCTTCCCCAAGAGCTCACATCGACGGGAAGGTTTGGCACCTCGATGTCGGCTCTTCGCCACCTGGGGCTGTAGTATGTTCCAAGGGTTGGGCTGTTCGCCCATTAAAGCGGTACGTGAGCTGGGTTCAGAACGTCGTGAGACAGTTCGGTCCATATCCGGTGTGGGCGTTAGAGCATTGAGAGGACCTTTCCCTAGTACGAGAGGACCGGGAAGGACGCACCTCTGGTGTACCAGTTATCGTGCCCACGGTAAACGCTGGGTAGCCAAGTGCGGAGCGGATAACTGCTGAAAGCATCTAAGTAGTAAGCCCACCCCAAGATGAGTGCTCTCCTATTCCGACTTCCCCAGAGCCTCCGGTAGCACAGCCGAGACAGCAACGGGTTCTCCGCCCCTGCGGGGATGGAGTGACAGAAGTTTTGAGAATTCAAGAGAAGGTCACGGCGAGACGAGCCGTTTATCATTACGATAGGTGTCAAGTGGAAGTGCAGTGATGTATGCAGCTGAGGCATCCTAACAGACCGGTAGACTTGAACCTTGTTCCTACATGACCTGATCAATTCGATCAGGCACTCGCCATCTATTTTCATAGTTCAACTCTTTGACAACACGAAAAAACCATTGTTCAACTCTTTGACAACATGAAAAAACCAAAAATTCTGCCCTTCTATCCAAAGGATGGAGGGGCGGAGGCCTTTGGTGTCCACTCCAGTCAAGAATTGGAGCCTCACAATCACTAGCCAATATGCTTTTCTCGCATGCCTTTCTTCGTTCATGGTTCGATATTCTGGTGTCCTAGGCGTAGAGGAACAACACCAATCCATCCCGAACTTGGTGGTTAAACTCTACTGCGGTGACGATACTGTAGGGGAGGTCCTGCGGAAAAATAGCTCGACGCCAGGATGATGAAAAGCTTAACACCTCTCATTCTTATTACTTTTTCATATTGAAAAAAAATGCAAAATGAAAAGGTTGTCTTATTCAAAACCCCAATTATGAAATCCCTTCTATCCCACTTCACACCCCGGAACGCACCGTTCTTATAGAGAGAAAGGCACTTTCACATCTTCTTAACCCGAAATGGCTGGGGAGAGGAAAGGTTCCTTTTTTTGTAGGGTACTCCTGGGAACAGATCCAGTGGAGACGGGGTGGGGCTTGTAGCTCAGAGGATTAGAGCACGTGGCTACGAACCACGGTGTCGGGGGTTCGAATCCCTCCTCGCCCACAACCGGCCCAAAAGGGAAGGACCTTTCCCTCCGGGGGGTAGGAAAATCATGCTCGGGATAGCGGACTCAAAGCTATGGAACTTGGTTGGGGATGGGTCTTTTGTCGAAATAGAGTGGAGTGGCCTTCTTTTTTATTTGAATTTAGATATATATATCTATTATATCTATCGCTTTTTTTTTTACATATAGTATGATTATCGGCCGAATCAGCATATTTTTCGAAGCCCCGTAACTCTTCCTCAGCCAGGCTTGGGCAGAATAGCAGAGCAAGTACAAGTATTAGTAGCATAGAAAAAATGCGTTCCTCATCATTAAGTCATTAATATGTTTGCGCGCGGTAATTGTGAACTCTCGGGAGAATCGATGACTGCATCAAAGATGCACTTGTTAGTACACCTGCAAATTCTGAATTGGCTAGTTGTAAATAGCCCCAGGACTATGGAATAAAGGATTATCCCGGACCTACACCGAGGTATTGACGGTGATTCTCAAATATCACAGAACAGAATGTGATACGATGAGATAGAATGCAATAGAAACAAAGACACAGGGAACGGGTTACCTACTCTTACATTCTGAATTCTTGAATTCGGAATGAATCAAATCTCCCCAAGTAGGATTCGAACCTACGACCAATCAGTTAACAGCCGACCGCTCTACCACTGAGCTACTGAGGAACAACGGGAGATTAGATCTCCTAGAGTTCAATTCCCGTTCTCAACCCATGACCAATATGAACTCGAAGTTTCCTTCGTAACCCCCGGAACTTCTTCGTAGTGGCTCCGTTCCATGCCTCATTTCATAGGGAACCTCAAAGCGGCTCTATTTCATTATATTCCATCCATATCCCAATTCCATTCATTTAATATCCCTTTGGTGTCATTGACATAAGAGATGTCGTTTCTAGTCTATCTCTTTCTATTTCTATATATGGAAAGTTGCAAAATCATCATATAATAATCCAGAAATTGAAATAGAAAAGAAAAAAGGGAGGTTTGTGATGGTTTTTCAATCTTTTATACTAGGTAATCTAGTATCCTTATGCATGAAGATAATCAATTCGGTCGTTGTGGTCGGACTCTATTATGGATTTCTGACCACATTCTCCATAGGGCCCTCTTATCTCTTCCTTCTCCGAGCTCGGGTTATGGACGAAGGAGAAGAAGGAACCGAGAAGAAAGTATCAGCAACAACTGGTTTTATTGCGGGACAGCTCATGATGTTCATATCGATCTATTATGCGCCTCTGCATTTAGCATTGGGTAGACCTCATACAATAACTGTCCTAGCTCTACCGTATCTTTTGTTTCATTTCTTCTGGAACAATCACAAACACTTTTTTGATTATGGATCTACTACCAGAAATGAAATGCGTAATCTTCGCATTCAATGTGTATTCCTGAATAATTTCATTTTTCAATTATTCAACCATTTCATTTTACCAAGTTCAATGTTAGCCAGATTAGTCAACATTTATATGTTTCGATGCAACAACAAGATGTTATTTGTAACAAGTAGTTTTGTTGGTTGGTTAATTGGTCACATTTTATTCATGAAATGGGTTGGATTGGTATTAGTCTGGATACAGCAAAATAATTCTATTAGGTCTAATGTACTTATTAGATCTAATAAGTATAAGTTCCTTGTGTCAGAATTGAGAAATTCTATGACTCGAATCTTTAGTATTATCTTATTTATTACCTGTGTCTACTATTTAGGCAGAATACCATCACCCATTTTTACTAAGAAACTAAAAGGAACCTCAGAAACGGGTGGGACTAAACAGGACCAAGAGGTATCCACCGAAGAAGCTCCTTTTCCTTCTCTTTTTTCGGAAGAAAGGGAGGATCTGGACAAACTCGATGAAATGGAAGAAATCGGAGTGAATGGAAAAGACAAAATTAATAAGGATGATGAATTCCACGTTCGAACATACTATAACTATAAAACAGTTTCTGAAAATCGAGATGGAAATAAAGAAAATTCTAATTTAGAATTTTTCAAAATAAAAAAAAAAGAGGATCGTTAAAAAAATCAATACATAGCACAAATACAAGAACAGATAAGAAGAGATGCGACTTCCACCTATATATTTTGTTACTTCTCCTACAAAGAAACTTGTAATACCTACTCCATTTGTAATTCCATCAATGATTCGTTTATCAAAAAAATTCGTTTGTTTTGCTAATTTTCTTATACTTTCAGTTAAAGATTTTTTAAAAAAAGTATCTATGTAACCACGATTATATGACCAATTATATACAAAATTGATTGGTTTTTCCCACCTAATTCTTTTAGAACTCCACTTTTGAAATGAATTAAGTAAAGTTAAATTTAATCTAGATGAATAAAAAGGCTTATATAAACAGTATGCTATAAATATTCCAAACAAAGCTATACTGACTGAAAAAATTGCATTTTTCAAAAATTCATACCAATCTACAAAATTTTCTGAATTGGTATGCAAAAGGTTTATCGACGGCGTTAATAATTTTGATAATATATCAAAGTCTATTCCTTCTTGATTGAAAGGAATTCCTATGGCTCCAATAAACAAAGTAAATAAAAGCAATACAAGCATAGGAAATAGAATAGTATTGTCTGATTCATGGGGATAATAGAAAGTTCTTGTATTAAGTCCAAAATTTTCAACAGTAATAAAAGTTTGATTTCTTACATTATTACTAATTTTATATGTTTTATTGCCAAAAAAAGAAGCTCTTTTCGTATTATTCATTGTTAATAATGGTACTAACCCAAAATTCCTATTAAGTTTTTTATCTTCTTCTTTACCCCATAAAGAAATTGAATAGAAGGAGCGACTTTTTTTTCCACTATAATTTATAAAATAAGTGTTTAAATGGCCTTCAAAAGTAAGTAAATAAATCCGAAACATATAAAATGCGGTTAATCCCGCTGTTGAACAAGCTATTATTGCAAAAATTGGCGAAAATAACAAACTATCATTAAGAATTTCATCTTTAGACCAAAAACAAGCAAGGGGGGGAATACCACAAAGTGAGAGTGTTCCTACTAAAAAGGCAGTTTTTGTAATCGGCACATGTTTTGTCAAACCACCCATAAGAATCATATTCTGACTTTTATCAGGAGAATAGCCAACTATAGCTTCCATTGAATGAATAATGGATCCAGATCCTAAAAACAACAAAGCTTTCGAATAAGCATGAGTAATCAAATGAAATAAAGCGGATCTATAAGATCCCATACCTAGAGCTAACATCATATAACCCAGTTGAGACATTGTAGAATAGGCTAAACCTCTCTTAATATCTTTTTGAGCAAGAGCTAAAGTGGCTCCTAAGAGTACTGTTATTATACCTATCAAAGATATTATATACATTATAGAAGGGATAACTATAAAAAGAGGAAGAAGACGAGCTACAAGAAAAATTCCCGCTGCTACCATAGTAGCAGCATGTATAAGAGCCGAAATAGGAGTAGGGCCCTCCATGGCATCCGGCAACCATACATGAAGAGGAAATTGTGCAGATTTAGCAATAGGACCCACAAATAATAGAAATGCACACAAAGTAAGGAATAAGAGATTTATTCTATTATTTAATATTAAATTATTGAATATTTCGAACAAATCTTGAAATTCGAAACTGCCAGCTATCCAATAAAGACCTAAAATTCCTAATAATAAACCAAAATCCCCTACACGATTGGTTACAAAAGCTTTTTGACAGGCATTCGCTGCAATAGGTCGTGTGAACCAAAAACCTATTAATAAATACGAACACATTCCAACTAATTCCCAAAAAAAATAAACTTGGATCAAATTAGAACTAGTAACTAATCCTAACATTGAAGTATTAAAAAAACCCATATAAGCAAAAAACCTCAGATATCCTTGATCATGAGACATATAATTATCACTATAAATCAGAACCAAAATTCCAACAGTTGTAATTAATATTGACATAATAGAAGTAAGTGGATCAATAAAGTAACCGAACTCAAAAGAAAATTCATTATTTATGGTCCAAGACCATACATTTTGATGAATGCAACTTAGAAAAATTTGTTGAATAGATAGATAGAGCGAAAAGATCATAACTATACTTAACAAAAAAATACTCAGAAACGTCCACATACGTCGAAGGTTTTTTGTTGCTGTCGGAAAAAGTAGAAGTCCAGCTCCGAGTAAAATAGGTACTGGAAGTGGAATGAAAGGGATGATCCATGAATATTGATATGTATGTTCCATAAAATAAAAAACCCTTTTTATTTTATTCTTAAATTTATTATTTCTTATTCACTGGTTTGTATATATATATATATTTTTTCAAAGGGGATAATAAAAAAGCGCATTTTTTCAAACTTAAATAGAAATTTTTTCGAATTAGTATAATCCTTCATAAACCTTTGAAAAGAAATATATTCAAATCAAAAAATTAGAAGTTATTAACTAATATTACTAAGTTACTGTAAAAAAAACGATTTGTCTTTTTTTTTTTACTACTAAAAAAAATTTTGATTTTATGCAGATACAGAAAAAGTGAATTCTAATTCCGTATTACAATAATTTATATACATATATTAAGAATAGAACAAAGATTTACACGACAAAAAAATACTTAATATTAAGTATAAAAAAAAGTTATTTGGGTTTGATTATTTAGAATTATTATTGAATTATGGAATTTAGTGATTGTCTTCCAGTACACTAAGTGAGCTTTTTTTTCAAGAAATATTATTATAATCGATATTTTTTTTTACATATGAAGTGAAGAAAGTTAATAAAATTTTTTTCTAATATAATCTAAATCTATCAGTATAGATTAATTAAATGAGCACTCTCATACGGATTTAAAACGTTAAATACAAAAAATTTTTCAAGAAAAAGGGAAAAAATAGTTGGGTTTTAAACTTTTGAATGTCTGTTTTGTTTGAAAAATAATATATAATAAAATTTGAAAGAAAAAATTTACTCAATATGGAGTACGAAAGAATAGAATAATAAATGTCTTTGACATCCAATTATACCACTGAAAAACTTTTTTCATTTTTGAATGGCAGTTCCAAAAAAACGTACTTCTATCTCGAAAAAGCGTATTCGTAAAAAAATTTGGAAAAGGAAGGGATATTGGACATCGTTGAAAGCTTTTTCCTTAGGGAAATCGCTTTCTACAGGTAATTCAAAAAGTTTTTTTGTACAACAAAATAAATAAAAAACACTAGAATCATTAGAATTAGCCTAACGTAAAAACCAATTTTGTAGAATACATATAAATTAAAAAAATCTATAGGAACCAAAATAAAAAAAAAAAAGATAATATATATACGAAATATACGAAAGATTCCTATTGATTTTGTAAAAAAAAGGGGGGGTTATTACTTTCCCCATCAATAAAAAAAATAAATAAAGATCTTGTATTTCCTCTTAACTAGGAAATACAAGATCTTTTAGCGAAATCAACAGGTTCTTTAAATTAATTTAAGTCAAATAAGTCAAAAATTTTTCACTTTATACCTTTAGGAATTATTATTTCTCTTAATTCTTATATTCTTTACTTGGAATCAAAGTTATAAAAGTATCTATCCACAATTAAGTGAATATTAGATACTAATAAGTAATAATATATGATATTTTTTTTAAGCGATCAAAAAATATTATGTTTGTACAATATAAAAAGATGCATGAAAATAGATATTTTGACAATTGTTGTTTTTCATTTTTCTTGAGCAACTTAGGCAAATTTTAGTTAAAATTTCTAAGGATTTTGGAGAAGTTTTAATTTTTAGAAAAAGCATTTTTTTAGTAATAAAATCAATTTTAAATTCCATTAAATTAGCTTCTTTATTTAAAATTTGAATCTCGACGATTGAGTAAAAACTTGTTAGTATTATTTTGAACAAGTTGCCGCTATGGTGAAATTGGTAGACACGCTGCTCTTAGGAAGCAGTGCTAGAGCATCTCGGTTCGAGTCCGAGTAGCGGCATAAGATCTTATAAAAGAGATATTATAAGTTTTATAATCAAATTAATACCCGACTTGTTTTCTAAAATCGGGTAAAACCTAGTATTAATTTATTAATTTTTAACAAATTTTTTATGATTTTTTCAATTTTAGAGCATATATTAACTCATATATCTTTTTCGGTCGTTTCAATTGTACTACTAATTTATTTTTTAACTTTATTAGTTAATTTAGATGAAATCATAGGATTTTTTGATTCATCAGATAAAGGAATCGTAATTACGTTTTTTGGTATAACAGGATTATTATTTACGCGTTGGATTTATTCAGGACATTTTCCATTAAGCAATTTATATGAATCATTAATTTTTCTTTCATGGGCTTTTGCAATTATTCATATAGTTTCCTATTTTAATAAAAATAAAAAAAATCACTTAAACGCAATAACTGCGCCAAGTGCTATTTTTATTCAGGGTTTTGCTACTTCAGGTCTTTTAAACAACATGCCTCAGTCTGCAATATTAGTACCAGCTCTCCAGTCCCAGTGGTTAATGATGCACGTAAGTATGATGATATTAGGCTATGGCGCTCTGTTATGCGGATCATTATTATCAATAGCTCTTCTAGTCATTACATTTCGCAAGGTCGGATCTACTTTTTGGAAAAATAATATGAAAAATAAAATGTTATTAAATGAATTATTTTCTTTTGATGTACTTTACTACATAAATGAAAGAAATTCTATTTTAATACAACAAAATATTAATTTTAGTTTTTCTAGAAATTATTATAGATATCAATTGATTGAACAATTAGATTATTGGAGTTTTCGTATTATTAGTCTCGGATTTATCTTTTTAACCGTCGGCATTCTTTCAGGAGCTGTATGGGCTAATGAAACATGGGGTTCATATTGGAATTGGGATCCGAAAGAAACCTGGGCATTTATTACTTGGACCATCTTCGCAATTTATTTACATATTAAAACAAATAGGAATGCTCGAGGTATAAATTCTGCAATTGTGGCTTCGCTAGGTTTTCTTTTAATTTGGATATGCTATTTTGGCGTCAATCTTTTAGGAATAGGTTTACATAGTTATGGTTCATTTACATCGAATTAACTAAAACATTAACAAAAAAAGAAAAGAATCCAAATAAAAAAAATAGCATCTATATATAACTTCATATAAGTTAAGAAATCTAATTTAGTTTTAGTAGTAAATCATCAAGAACCTTTTGAATCAAGTAGTACAATGATTCAAAAGGTTCTCACAATACAAAAAGCAAAGACTTCTTATTATAATTCAATTTAATGTTTTTTTTTATTTCCTGAAAACTATCCATAAAAATAATTAGATAAAATGGATTCGACCTTGTCACTTGCTAATGAGAGCACAAAATCAGGATAAATCCCAATACCAATTATGGGTAGAAGAATAGAGATTGAAAGAAATAACTCTCGGGGTCCAGAATCAAAAAAAGAAAAGTTTTTGGCATTAATTAACTTGTATCCATAGAACATTTGACGTGACATAGATAATAAATATATAGGAGTTAATATCATTCCAATTGCCATTACAAAAATAATTAAAATTTTTGAAATTAAGAAATATTTTTGGCTGGTAATTATTCCAAAAAAAACGATTAATTCTGCAACAAAACCACTCATGCCCGGTAATGCAAGGGAAGCCATCGATAAGATAGTGAACATTGTAAATATCTTTGGAATGGAGATAGCCATTCCACCCATTTCATCAAGATAAACAAGCCGGATTCTATCATAACTAGTTCCTGCCAAGAAAAAAAGTGCAGCGCCAATAAATCCATGAGAGATTATTTGTAAAATAGCTCCATTAAGCCCAGGATCCGTTATAGAACCAATACCTATAATTATAAAACCCATATGAGATACAGAAGAATAGGCTATTCTCTTTTTTAAATTACGTTGACCGGGAGATGTTGAAGCTGCATAAATTATTTGGATTGTACCGACTACCATCAACCAAGGAGAAAACATAGAATGAGCGTGAGGTAATAATTCCATATTGATTCGAACCAATCCATATGCTCCCATTTTTAATAAGATTCCAGCGAGAAGCATACAGGTACTGTAATGTGCCTCGCCGTGGGTGTCAGGTAACCAAGTATGTAAAGGTATAATCGGTGATTTGACGGCAAAAGCAATAAGAAATCCAATATAAAAGAGTATTTCGAGTGTGACCGGATAGGCTTGATTCCCTAATAGTTCTAAATTTAATGTTGGTTCGTTCGAACCATATAAACTTATACCTAAAACTCCTATTAATAAAAAAATAGAACTTCCTGCAGTGTATAAAATAAATTTTGTAGCTGAATACAAACGTTTCTTTCCACCCCACATGGATAAAAGGAGATAAACGGGAATTAATTCTAATTCCCACATGATGAAAAAAAGTAAAATATCCCGAGAAGAAAACGATCCTATTTGGCCGCTGTACATTGCTAACATCAGGAAATAGAATAATCGGGAATCCCGAGTAACTGGAAAAGCCGCTAAAGTAGCTAAAGTAGTAATAAATCCGGTCAGTAAAATCGTTCCTATAGAAAGTCCATCTATTCCCAGTCTCCAATAAAAATCAAAAAGATTGATCCATTTATAATCTTCGGACAGTTGAATTAATGGATCGTCCAGTTTAAAATTATAACAAAAAGCGTAAGTCGTTAGAAGAAGTTCTAAGATACAAATGCATATAGTATACCACTTATTAACTTTATTTCCCCTATGCGGGAGAAATAACATTAATGAACCGGCAGATATTGGAAAAACAACAATTATTGTTAACCAAGGAAAATCATTCGTGGTAAAGACAAGATACACCAGGTCCAAAGAACGCGTACTCAAAAAAATATATAAATAAAAAAATATAATTGAACTTTTTTGAGTACGAGTACTTGTCAATAAAAAAAAATAAAATGTATTCCAAATTTATTCAAATCAGGTTTTCGGTAACGTATTAATAAGCTAGACCCATGCTTCGAGTTGTTTCATGCCATAAATAAACTCGAACGCTCAAAAAATCCGTTGGACAGGCAGATTCACATCTCTTACAACCAACACAATCCTCGGTTCTTGGGGCAGAAGCTATTTGCTTAGCTTTACATCCATCCCAAGGTATCATTTCTAATACGTCTGTAGGACATGCTCGGACACACTGAGTACATCCTATACAGGTATCATAAATTTTTACTGAATGTGACATAGGATCTATAGTTTTTTTAATGTCATAAATTTTCAATCTAGTAAACTTATAACTAAATGATATATTAAATTAAAATACTAGATGAAGCAATGATTTCTTTTAATAGAATTTTTTAATGAATTCTGGCTCAATTGGTAAAAAATGGGGCTAAAATACTTTGATTTCTTAAATTTTCACAAATTTAATCTAGTAAGTCATAACCTATCATATATGCAAATTTAAACCTATAATTTTTTGATTTATGCTACTTATTTAATAAGGTCGATTGGTTGATGCGAGTTGATTTTCTGTTACGATAAATTGACGAGACTATAGCTAATCCAATAGCTGCTTCAGCGGCTGCAATTGCTATAACAAAAATGCAGAAAATATCCCCTTTTAGTTGGGAATTATCAAAAAAATCAGCAAATGTTACGAGATTCATATTAACTGCATTGAGTATAAGTTCAAGGCACATAAGAGCCCTAACCATATTTCGACTCGTGATCAATCCATAAAGACCAATCAAAAATAAATAGGCACTCAAAACAAGTACATGTTCGAGTATCATTGAGCAACTCCTTATCAATTTTGATTCATTATCAATATGAATAATAAAAACAATTCACCGGATTCAATCAACTAGAATATAACAACAAAGTACGAATAAAAACTATATTAGATATTAGGGAAAAAATTTCAAATATATATATAATATAAAAATTTATATTTAAAAAATGAAATAGTATTCAATCAAATCAAATTGAATGAACGGAAAAAAATATCATAACATACACAAACACAAAGTTTTCTTTGGTCTTTACTAATTGGAACCTTTTTTATTGACGAGCCACAGAAATTGCACCTATCAAAGCAACTAAAAGAATTATTGAAATGAGTTCAAATGGAAGAAAAAAATCTGTTGATAAATGAATTCCTATTTGTTGACTATTACTTATTAAATCTTGTTCTAAAATCTGGTTTAATCTTGTAGTCCAAATAACCCCGTACCATGACGTATCGAGAATAGTAGAAATTAATGAAAAAAGAATAGTTGTACAAACCACTGAAGTAATCCCATTCCCAACAGTCCACAGATTGAAATCTATGGAATATTCGGAATCATTCATGAACATCACAGCAAATATGATTAAAACATTTATGGCTCCCACGTAAATAAGGAGTTGTGCAGCAGCTACAAAATGGGAATTTGCTAGAATATACAATAAAGATATACAAACAAGAACAAATCCTAAGGAAAAGGCTGAAAATATTGGGTTAGGAAGTAATACCACTCCCAGACCTCCTACTAGAAGACCAGATCCCAGAAAAACTAAAAGAAAATCATGTATTGGTCCAGGCAAATCCATTATATTATTAAAAAAAGAAAAAATAGAAATCCTTTTCATGACCTTATTAATTTAACCGGGGAATTTTTTTTTAATATGTTTTTAATAGAGTGAAATTAGAATCTAATGAATATTAATTGATGTAGATACAATTATTAGACAGTTTCGCTTTTTTATTCTAATATTTTCAACCTATCTATTTCAAGCAAGATAATAATTACGAAAATATTATATTAAAAGGATGAGCCTTAATACTTAATATTATTCTATAAATACAAGTTTTTAATTAAATTCTTTATATAATATAATTCAACAGTTTTGCATTCTTTTTTTAATAAAATTAATGGGTTTACCCATTTTTTGTTTGAGGTGAATTCAAAATTGTTCGAATAGTATAATCGTCAATTACTGACATTGGTAAACGCCCCAAAGCGATTTGATTATAATTCAACTCGTGACGATCATAAGTTGAAAATTCATATTCTTCAGTCATTGACAAACAATTTGTTGGACAATACTCAACACAATTACCACAAAATATACAAATTCCAAAATCAATACTGTAATTAAGCAATCGTTTTTTTCGAATATTGGTTTCCAATTTCCAATCAACAACCGGCAGATCTATAGGACATACTCGAACACATACTTCACAAGCAATGCATTTATCAAATTCGAAATGGATTCGACCGCGGAAACGTTCTGATGTTATTAATTTTTCATAGGGATATTGAATAGTTACAGGTAAACGATTTGTGTGGGATAAGGTAATCATGAAACCCTGACCAATATACCTTGCAGCTCGTAGGGTTTGTTGACCATAATTCATGAACCCGGTTATCATAGGAAGCATATTGTAATTATCTATGAATAATTTGATCTTTGTTTCTTTCTCTTGTTTAAAACAAGTAATGAATATCTTGGATTGATTTTCAATTTAGAGTGAAAAGAGTTGGAAAGAAGTTGTTAATAATAGATTACCAAGGGAAATCGGTAAAAGAAATTTCCATCCAAGATTTAATAGTTGATCCATTCTTAGCCTAGGTAAAGTCCATCTGGTTGCGATAGAAATGAACAAGAACAAATAAGTTTTAGCTAATGTAATAAAGATACCAATTGTTGTTCCAAAAATTTGATCCTTTTCAAATAGCTCCAGCATAGATATATACGGAATAGAAATATTCCAACCGCCTAAGTATAGAACTGTTACAAATAATGAGGAAATTAATAGATTTAGATAAGAAGCAACATAAAATAAACCAAATTTGATACCGGAATATTCAGTTTGATAACCTGCTATTAATTCTTCTTCCGCTTCTGGTAAATCAAAAGGTAACCTCTCGCATTCTGCTAGGGAAGAAATTAGAAAAATGATAAAACCTATAGGTTGACGCCACAAATTCCATCCCCAAAAACCATATTTTGATTGTGCCTCAACTATATCAACTGTACTTAAACTGTTAGATAATCCTAGTCGGTGATAACATTACTATTCTCACCGCTATTACAAAACCGTACATGAGGTTTTCGCCTCATACGGCTCCTCGGGGGCCGTAAATAAATATAAGGACCAGATTAGTATTATTTAGATGGATATGATGTGTTCTAAAATGGATTAAATAGAAATATATCTGGGGTCCCGAATTATACCAATGGAATTCTGTCTGCTCAAATTCTAAAACTAAAAAACGCGCTTCGGAATTCATCTCATCCTTTACAAATTTTAATTTCTATTTGTTGAGTAATAACTTAATCCTTTAATAAAGCACCCCTTGTAAAAATAAACCTAGGTTTTTAAGCCCGTCGTGTTTTTCAATTACGAAAAAGAATTAAACATCCTATTAGTTTCTTATTCATGATAGAAATTCTATTTTATTTTCGAAATCTATCAAAAAAAATATACTTGTTTCGTTCCTATTCTTCTTTCTTTTTTAGAAAAAAAGTAGGTGGACTTAAAAAAAAAATAAAGGATTATTTCGTTTCTGATAGTCATTACATTTATCGGTGGATGGGAGCATACTCTGAATCGGAATCTTGGGGAGTACTGCCTGATAATTTCTACAAATTTCAAGCCCCAATTAACCTTCTTTTTTTGTTATCTTATGTTATGCATAAATATCCTTTTCAATTTGGTTAATCTCTATTACAAATTCTTTGTGTATTTTGGTGTTTCTAACCATCCACGCGTTTTTACCTAATTGCCGATCACTTTGTAATATATGTATATGTATAGTAATTTATATAACTGATAGTGAAAACGTCATACGGTTAATATTTTTTTTAACCCGCTTCAAGCCCGGCTGACTAATCAACCAACCTTGGGGTAAAGCGATTCTTACGCTTACGTTTATTTCCATTTAACCTTTGTACATAGGAAATGAGACTTAATTTTTCTTTTTACTGCTAATTTCTGAGCAGTTTTTTTTCACTCATATATAACTATCAAATTCCTTTTATTAAGATAAACCCGAAAGATAAATATATATATTCCGTTTTTTTTTTTCATTTTTTTTATCTAGAAGAAACGGAATAAACCTTTCTGTTTCAACGAATCGCACGTAGAGATATTGATAAAACACATAGAGTTAATGGTATTTCATAACTAATCGCTTGAGCAGCAGCTCGCAGACCACCTAAAAAAGAATATTTATTATTTGATCCATATCCTGACATAAGAAGTCCGATCGGAGCAACACTTGAGATGGCAATCCATAAAAAAATACCGATATTGAGATCCGCTAAAACAAGGTGATTGCTAAAAGGAATTACTGAATAACTTAGTAAAATAGAGATAACTGCTATAGATGGTCCAATACTAAATAAAGGAGTATTTCCTCTAGATGGACGAAGATCTTCTTTGAAAAGTAGTTTTGTCCCGTCGGCTAAAGCTTGAAGAATTCCTAACGGGCCGGCGTATTCAGGTCCAATACGTTGTTGTATCCCTGCAGATATTTCTCTTTCTAACCACACAATTACTAGTACACCAGTTATGATTCCCAATACAAGAGAAAATATAGGGACAAATATCCATATGAGTCCATAGACCTCTTTTAAAGATTCCAATCTAAGAAAAGAATTTATAGTTTGTACTTCTGTTGCATAAATTATCATTTTAACGATCAACTTCTCCCATAATTATATCTATGCTACCGAGTATCGTCATAATATCAGCCAATTTCATTCTTTTAACTAGTTCAGGAAGAATTTGCAAATTAATAAAACCCGGCGGTCGGATTTTCCATCTCCAAGGAAAACCACTTTGATCTCCTATGAGAAAAATTCCCAATTCCCCTTTTGGAGCTTCAACTCTTACGTAAAGTTCTTGTTTCGATAATTCAAAAGTAGGGGAAGGTTTTTTACTAATGAATCGATATTCAAAATCATTCCACTCTGGATTCCTTTTTTTATCAAAGCCTCTGCTTTCTAAATTTTCATAGGGACCCCCCGGAAGTCCTTCCAGAGCCTGTTGAATAATTTTGATGGATTCTGTCATTTCGCTAAGTCGTACTAAATAACGAGCTAATGAATCTCCTTGTTTTTGCCACTGAATTTCCCATTCAAATTCATCGTAAGACTCATAACGATCAACTTTACGAAGATCCCATGGTATTCCGGATGCGCGTAACATTGGTCCGGATAAACCCCAATTTATTGCTTCTTCCCCACCAATAATCCCAACGCCTTCAACTCGTTCTAAAAAAATAGGATTTCGTGTAATAAGTTTTTGATATTCAACAACCTCTGTTAAAAAATAATCACAAAAATCCAAGCATTTATCTATCCAACCATAAGGTAAATCCGCCGCTATTCCTCCAATACGAAAAAAATTATGCATCATTCTCATACCGGTGGCAGCTTCGAATAGATCATATACAAATTCTCGTTCTCTGAAAATATAGAAAAAGGGAGTCTGTGCCCCAATATCTGCCATAAAAGGGCCAAGCCATAACAGATGAGAAGCTATACGACTCAATTCTAGCATAATTACTCTGATATAGCTGGCTCTTTTAGGAACTTGAATATTTCCTAATTGTTCGGGTCCGTTTACTGTTATTGCTTCTGTAAACATAGTAGCTAAATAATCCCACCGCGTTACATAAGGTAAATATTGTATAATTGCTCGGTTTTCTGCAATTTTTTCCATTCCTCTGTGTAAATAACCCAATATGGGTTCACAATCAACAACATCCTCACCGTCTAGAGTAACAATTAAGCGAAGAACACCGTGCATGGATGGGTGGTGAGGTCCCATATTGACTATCATAAGATCTTTTCCTGTAACTGGTCTCTTCATAAGTTTTTCCTTGATTCGTTCTGGTATGAATTAGATTGCTGAAAAAGAAGTTTATTCAAAAATTCAAGATCTAAAAAATTAACTAATTCACAATTTTGGAATTTAACGAGTTTTTAATTCCCGAATATTCAACTGATTAATTAATTCTTTATAACGTACTCTATTTTTTTTTGACAAATAAGCCAGCAGTCGTTGACGTTTTCCCAGAATTTTTCGTAGACCTCTCTGAGATAAATAATCTTTTCTGTGCAATTCCAAATGTGAAGTAAGTCTTCGTATCTTATTAGTGAAACTGACTACTTGAAATTCAACAGATCCCTTGCTTTCTTCTTTTTTTTCTTGAAATGAAATGAATGTATTTTTTATCATAAAAAGAAATCCTTCCCTTTTTAATATGAATTGAAAGATATGAATTTTACTGATCAGTAATAATAATGGTAGTTTTTTTGTACAAGGATCCGAATTTAATTATCAACTTCTTAATTCTTAATTTTATAAAAAAAAAAGTTTAAATTTCGATCTAAAAAAGGAGGATTTTAAAAATTTATTTATGAGTTCGCTCTGAGTGGTATCTATGTCATTAATTCAATGAATCTCATGTATAAAGATTGAATTAAAAAAAATCCCTCACATTTGTGCATCCAATTGTTTTCATATACCGTAACTTAATACTATCTATATATATAGTCAAAATATAGTAAAAAGGATCTACCATTAATGCATTTGAAATCGCGTATACATGTGTATTCTTATCATACTGAAATTATTTCCATTAGTCGTATTAAACCAATAGCGATTCATACAAGCTAAATCTTCTAATCGAAAATTGGGCCAAAGAAAGGATTTTAATTTAATTAGGTTTTTTTTATCCTTATCAAGATCTTTCTTTTTATTCAAAACTGTGGTCAAGTTTTGAATATTTGTATCAAATTTTGAATTTCTATCCCTTGCATTTTTTTTTTTTAAATTGAAACAAATTAGAACTCGAAATTCTTTACGTCGTTTAGGGGATAGAATAGTTTCAGGGACAAAGAAATTTAAACTTTTTTTTTTATAAATATAGCTTTTTTTTTTTGATCTTTTACTTATTTTTTGTTTATTTTTATGAACCAATGAAATCCCGATGGTTCTATATATAATAAGTTGGCCGTCGTTTTTTACAGACAAACGAACAGGTTCAACAATCAATATTCCTTTTTTCATTAATTTTGAAAAAGTTAAATTCTTCTCAATCATTAGAATATCTAAGCTCATCTCTCCTCTTTCAATACAAGATATCGTTATCTCGGTTGGATTTTTTAGTCTAACCAAGAAACAGTATGCTTTTACATTATTGAGGATTTTTTGATTAAAAAAACAATTCCATCGCAATTGAAAACGCGAATACCTTTTGAGAAATAAGTCAAGTTCCGCTTCGGTATTGCTTTTTGGTTGTTTTTTATTTTTACGTTTTTTTATCTTCGATTCTGTATAATTTTCTTCAATATTTTTTTCTTGCTTTGATAGAGCTGATTCCGTATTTATTTTTGTTTCTTTATCTGATTCAAACTCTTCTTGACCTGCTGATTCGTTTTCTTCTTTATTTAGATTAAAAAACCGAAGGAATTTTTTTTCGTTTGATGGTATAAAACCTTTTTTCTTTAGGGTGATCTGTTTATTCACATTTTTTGTTTCATTAAAATTGAAAAGAAGTAATTTAATTGGTATGACCCACGGTTTCATTTTATATGTACTAGAAAATAATAAAAATTCTGGAAAGAAAAAAAAGTCAAAATTTGTTATACGAGGATTGAGTATTTCTTCATTCATTCCCATCCAATCAAAAAAATTTATTTTTTGATTGGCAAGACTCGTCTTAGTTATTTTATAAATTCTTTTATAATTCTTAACTTTAGTCTTAATATATTCTTTTTTACTCCTAGTATCAAGCTCAATATTTACTTTTTTTCTAAACCAAAAGTTGAGAATTCTCCAATCCAAATATTTTCTATGCAAAATTTCCCCTATACTCCGAATATTATATTTTTCTAGAAAACAAGAGATTAAAAAATTTTCTAGGCCTGTAGACATATCAAAAAATTTTTCTGTAGAATGAATAGATTTGTAGCAAAAAAGATTATATATAGAATCCTTTTTGAAATTCTGTTTATGTTTTGGATTGAAAAATAAGTTAGCCTCACAAAAATTTTGTTTTTTGTAATTATCAAAAATTTTTTTTTCGTATGAATCCACTTTGTTTAAACTTGGATTTAGAACTAGAGAGTCTTGGTTTATTTTTTTTTTCCATTTTTGGGTTACTAATCTAGCCCATGCAATCTGAGGTAAATTATATTGAGAATGACTTCGTAACCAGTTTTTCCATTGATTTATTTCGGAATTTAAAAGGGTTTTATCTTTCCATTCATAATGAAAGATTCCTTGTTCTTGAAAAAAATCTTTTATTTGATTCTTAACAAAAAAGGATGTTATGTATATGTTATATTCAAAAAAAGACTTTAATTTAGAAAAGTTACTAACTTGAATTTGTGATAATTTGTAAAATACATATGCTTGTGATAAAGAGCATAAGTCATAACTAAAAAAATTTTTTTTTGATATGAAATTTTTTATAGTCGAAATAAAATAAATGGTATTTTTTTTTATTTTTTTTTCTCCATTTTCCTCATTCTTGTAAATATATACATCAAGAATTTTTTTTGTTGAATCAACAAAAAGTTGTGTAGTAATTCTTGGAATATTAATAATACCTAGAAAAATAGAAATAGACAGTTGTTCAACGCAAAATTTAAAAAAAAAAAAAGATTTACGAATTAATCGAGTTTTTTTTTTTTTTAATGTCTGCCAACTTTTTTTTGATGACTCAATTATTTTAGAATCATAACACAGTTTGTTACAACTATTAGTTAGTTTTTCTTTTTCTTTTGAAATTTTTTTCGTTTGATTTCTGATTGTCTTTATTTTATCAATCACATTTTTTATTTTGTTTTCGCTGAGTGAAGAATTTGGCCACTCCGTCGATTTTTTTTGAACAGATAGTTCATGAATCATCTGATTACTCATTATTGAATCTTTTTTAGTTTCATTTAATTCATATATTTCTCTCGGGCCAACTAATGGAATTCTATTTCGTTTTGAAAGGTTTTTTTTTTTTTCTTTTAGAAAAAGCAAGTTTTTTATAATCCAGTTTTTAATTTCTTTTTGGACTTTTAGGAAAATTGTTGCTCTTTCTTTGAAAATCCTTAAAACCGGAAAAGACTTCGTTTTGGATTTTTTGATTCTTTTTTTTAATTCTTTAAAAATAGGTTTAAAAAAAGAAGGCTTTGGTTTGGTAGAACCAAAAGGTAGGTCAGTTTCCAGCCCCCAAACTGTTAAAAAACGAAAATCATTTTTTTCTCCTTTTGTTTTTTTTAGTCGAGCCTTCTGAGATGATTGAAATTTATATTTATGCCAAGGTTTAAGATAAAACGGAAATAGGATTTTTATCTGAATACCATCCGTTAACCAGTTTCGTGGAAATTCTGTTTCGGATAGTTGAACCCCATTATAAGTACATTTAACATGCATTTCACGTTTCCACTCCTTTAAATCCTCTTCCCACTCGGGAAGTTGAAATAATACGATACGGATGCTATTTTTAATTATTATCAATAAAGGTAATATAATATATTTTCTAAGAATAGATTGAGTTACTAAAATAAAACCTCTTATTATTTGAGCAAATAAGAAGCTATCCCAAGTTTCCGCAATTTCTATACGTCTTTGTTCTTCTTTTTTTGATTGTTCTTCTTCGTTTTTATCAAAAAAAAATTTTTTTTTCCACATGAAATTTCTAAGAATTTTTTTTTTTAGCCCCCATATATCAAACGAAAAAAAAAAAATTTTATCTATTCTATCAAAAAAAAGGGGCGAATGTGCTTTTGCTTGCAACAATTCCCAAATAACAGTTTTACGCCTTTGGGAACGCGTGGATCCTTTAATTATCTCTCGACGAAAATCAGATTGTTGTGAATAACGGATCAAAGCCATTTCATCTGTTTGATCAGAATTTTGATTATCTTTGAGATTAGTATAAATCTCGTTATGCGGTTCTTTTAAATCAGTAAAAACCACTACACGTTTTGCTTTTCTTGAACGAATTCCAGGTTCGGTTGGTATATTTTCTTCAGTTTCAGCTTCCAATTCTTCCAATTCACTTGTTAATTTGTATGACCATTGAGGAACTTGTTTATTGATTTCATGGAAATCAATAAAATTTTTTATAAGAGTTTGATCATTATTATAAATTATAACGACATCAAATAAAATTTTGAAAATTTTTATTTCTTCTTCTGACTGAATTTTTTCTTCTTGGGGTTCGGAAAAAAAATAAAGTTTTTTCTCTATTGATAAAGACTTTCTTTTAAATTTTTCTATTGTTTGCTCAAATTTTTGAGAATTAATCTTCAGAAGTATAGCATGAATTTTGTTTATCCAAGATCCTCTTATATTCTTTTTTTTATAGGTTTTGGTTATGATTTGGAACGGAGATAATTTTTTGATTCTTCCGCGCGAAATCCCATGTAAAAATGGATCATAAATTTTAGGTAAATATTCTTTTTGAGTTTCGTTATGACAAAATCGAGTTGTTTTTTCCAGTATATTTTCAATAGACCCTTTTTTATCTAAAGCTTCAATTCTATTTAAAAATGCGTTTTTTAAATTTTCCTTTTTTTCTTCATTGACCAAACTCCAACAAGTATAAACTTGATCCGAGGTTTTTTTTTCTGTTGTAAATGAAGGGATCTTTTTTTGTATCATTTCAAAAAAAGTTGAAAGGTTGGGGGGATATGTAAAAGATATTCGTTCTTTTCCATCACTTTGGCATGTATAAAAAAAATATTGTGACATTTCATTTCTTACAGTATTTTCAATTTTATCATTTTTTATATATCGATTTGGTCTATTCCATCTTTTATAATCGAAAACTAGAGTTACAAAAGGTTTTTCAAACCATAAAAAACGATCCTCTTTTTTTTTTATTTTGAAAAATTCTAAATTAGAATTTTCTTTATTTCCATCTCGATTTTCAGAAACTGTTTTATAGTTATAGTATGTTCGAACGTGGAATTCATCATCCTTATTAATTTTGTCTTTTCCATTCACTCCGATTTCTTCCATTTCATCGAGTTTGTCCAGATCCTCCCTTTCTTCCGAAAAAAGAGAAGGAAAAGGAGCTTCTTCGGTGGATACCTCTTGGTCCTGTTTAGTCCCACCCGTTTCTGAGGTTCCTTTTAGTTTCTTAGTAAAAATGGGTGATGGTATTCTGCCTAAATAGTAGACACAGGTAATAAATAAGATAATACTAAAGATTCGAGTCATAGAATTTCTCAATTCTGACACAAGGAACTTATACTTATTAGATCTAATAAGTACATTAGACCTAATAGAATTATTTTGCTGTATCCAGACTAATACCAATCCAACCCATTTCATGAATAAAATGTGACCAATTAACCAACCAACAAAACTACTTGTTACAAATAACATCTTGTTGTTGCATCGAAACATATAAATGTTGACTAATCTGGCTAACATTGAACTTGGTAAAATGAAATGGTTGAATAATTGAAAAATGAAATTATTCAGGAATACACATTGAATGCGAAGATTACGCATTTCATTTCTGGTAGTAGATCCATAATCAAAAAAGTGTTTGTGATTGTTCCAGAAGAAATGAAACAAAAGATACGGTAGAGCTAGGACAGTTATTGTATGAGGTCTACCCAATGCTAAATGCAGAGGCGCATAATAGATCGATATGAACATCATGAGCTGTCCCGCAATAAAACCAGTTGTTGCTGATACTTTCTTCTCGGTTCCTTCTTCTCCTTCGTCCATAACCCGAGCTCGGAGAAGGAAGAGATAAGAGGGCCCTATGGAGAATGTGGTCAGAAATCCATAATAGAGTCCGACCACAACGACCGAATTGATTATCTTCATGCATAAGGATACTAGATTACCTAGTATAAAAGATTGAAAAACCATCACAAACCTCCCTTTTTTCTTTTCTATTTCAATTTCTGGATTATTATATGATGATTTTGCAACTTTCCATATATAGAAATAGAAAGAGATAGACTAGAAACGACATCTCTTATGTCAATGACACCAAAGGGATATTAAATGAATGGAATTGGGATATGGATGGAATATAATGAAATAGAGCCGCTTTGAGGTTCCCTATGAAATGAGGCATGGAACGGAGCCACTACGAAGAAGTTCCGGGGGTTACGAAGGAAACTTCGAGTTCATATTGGTCATGGGTTGAGAACGGGAATTGAACTCTAGGAGATCTAATCTCCCGTTGTTCCTCAGTAGCTCAGTGGTAGAGCGGTCGGCTGTTAACTGATTGGTCGTAGGTTCGAATCCTACTTGGGGAGATTTGATTCATTCCGAATTCAAGAATTCAGAATGTAAGAGTAGGTAACCCGTTCCCTGTGTCTTTGTTTCTATTGCATTCTATCTCATCGTATCACATTCTGTTCTGTGATATTTGAGAATCACCGTCAATACCTCGGTGTAGGTCCGGGATAATCCTTTATTCCATAGTCCTGGGGCTATTTACAACTAGCCAATTCAGAATTTGCAGGTGTACTAACAAGTGCATCTTTGATGCAGTCATCGATTCTCCCGAGAGTTCACAATTACCGCGCGCAAACATATTAATGACTTAATGATGAGGAACGCATTTTTTCTATGCTACTAATACTTGTACTTGCTCTGCTATTCTGCCCAAGCCTGGCTGAGGAAGAGTTACGGGGCTTCGAAAAATATGCTGATTCGGCCGATAATCATACTATATGTAAAAAAAAAAGCGATAGATATAATAGATATATATATCTAAATTCAAATAAAAAAGAAGGCCACTCCACTCTATTTCGACAAAAGACCCATCCCCAACCAAGTTCCATAGCTTTGAGTCCGCTATCCCGAGCATGATTTTCCTACCCCCCGGAGGGAAAGGTCCTTCCCTTTTGGGCCGGTTGTGGGCGAGGAGGGATTCGAACCCCCGACACCGTGGTTCGTAGCCACGTGCTCTAATCCTCTGAGCTACAAGCCCCACCCCGTCTCCACTGGATCTGTTCCCAGGAGTACCCTACAAAAAAAGGAACCTTTCCTCTCCCCAGCCATTTCGGGTTAAGAAGATGTGAAAGTGCCTTTCTCTCTATAAGAACGGTGCGTTCCGGGGTGTGAAGTGGGATAGAAGGGATTTCATAATTGGGGTTTTGAATAAGACAACCTTTTCATTTTGCATTTTTTTTCAATATGAAAAAGTAATAAGAATGAGAGGTGTTAAGCTTTTCATCATCCTGGCGTCGAGCTATTTTTCCGCAGGACCTCCCCTACAGTATCGTCACCGCAGTAGAGTTTAACCACCAAGTTCGGGATGGATTGGTGTTGTTCCTCTACGCCTAGGACACCAGAATATCGAACCATGAACGAAGAAAGGCATGCGAGAAAAGCATATTGGCTAGTGATTGTGAGGCTCCAATTCTTGACTGGAGTGGACACCAAAGGCCTCCGCCCCTCCATCCTTTGGATAGAAGGGCAGAATTTTTGGTTTTTTCATGTTGTCAAAGAGTTGAACAATGGTTTTTTCGTGTTGTCAAAGAGTTGAACTATGAAAATAGATGGCGAGTGCCTGATCGAATTGATCAGGTCATGTAGGAACAAGGTTCAAGTCTACCGGTCTGTTAGGATGCCTCAGCTGCATACATCACTGCACTTCCACTTGACACCTATCGTAATGATAAACGGCTCGTCTCGCCGTGACCTTCTCTTGAATTCTCAAAACTTCTGTCACTCCATCCCCGCAGGGGCGGAGAACCCGTTGCTGTCTCGGCTGTGCTACCGGAGGCTCTGGGGAAGTCGGAATAGGAGAGCACTCATCTTGGGGTGGGCTTACTACTTAGATGCTTTCAGCAGTTATCCGCTCCGCACTTGGCTACCCAGCGTTTACCGTGGGCACGATAACTGGTACACCAGAGGTGCGTCCTTCCCGGTCCTCTCGTACTAGGGAAAGGTCCTCTCAATGCTCTAACGCCCACACCGGATATGGACCGAACTGTCTCACGACGTTCTGAACCCAGCTCACGTACCGCTTTAATGGGCGAACAGCCCAACCCTTGGAACATACTACAGCCCCAGGTGGCGAAGAGCCGACATCGAGGTGCCAAACCTTCCCGTCGATGTGAGCTCTTGGGGAAGATCAGCCTGTTATCCCTAGAGTAACTTTTATCCGTTGAGCGACGGCCCTTCCACTCGGCACCGTCGGATCACTAAGGCCGACTTTCGTCCCTGCTCGACGGGTGGGTCTTGCAGTCAAGCTCCCTTCTGCCTTTGCACTCGAGGGCCAATCTCCGTCCGGCCCGAGGAAACCTTTGCACGCCTCCGTTACCTTTTGGGAGGCCTACGCCCCATAGAAACTGTCTACCTGAGACTGTCCCTTGGCCCGTAGGTCCTGACACAAGGTTAGAATTCTAGCTCTTCCAGAGTGGTATCTCACTGATGGCTCGGGCCCCCCCGGAAGGAGGCCTTCTTCGCCTTCCACCTAAGCTGCGCAGGAAAAGCCCAAAGCCAATCCCAGGGAACAGTGAAGCTTCATAGGGTCTTTCTGTCCAGGTGCAGGTAGTCCGCATCTTCACAGACATGTCTATTTCACCGAGCCTCTCTCCGAGACAGTGCCCAGATCGTTACGCCTTTCGTGCGGGTCGGAACTTACCCGACAAGGAATTTCGCTACCTTAGGACCGTTATAGTTACGGCCGCCGTTCACCGGGGCTTCGGTCGCCGGCTCCCCTGTCATCAGGTCACCAACTTCCTTGACCTTCCGGCACTGGGCAGGCGTCAGCCCCCATACATGGTCTTACGACTTTGCGGAGACCTGTGTTTTTGGTAAACAGTCGCCCGGGCCTGGTCACTGCGACCCCCTTTGTGAGGAGGCACCCCTTCTCCCGAAGTTACGGGGCTATTTTGCCGAGTTCCTTAGAGAGAGTTGTCTCGCGCCCCTAGGTATTCTCTACCTACCCACCTGTGTCGGTTTCGGGTACAGGTACCCTTTTGTTGAAGGTCGTTCGAGCTTTTCCTGGGAGTATAGCATGGGTTACTTCAGCGCCGTAGCGCCTGGTACTCGAACATTGGCTCGGGGCATTTTCTCTACCCCTTCTTACCCTGAAAAAACAGGGACACCTTGCGTTCTTGAACCGATAACCATCTTTCGGCTAACCTAGCCTCCTCCGTCCCTCGGGACCAACAAGGGGTAGTACAGGAATATTCACCTGTTGTCCATCGACTACGCCTTTCGGCCTGATCTTAGGCCCTGACTCACCCTCCGTGGACGAACCTTGCGGAGGAACCCTTGGGTTTTCGGGGCATTGGATTCTCACCAATGTTTGCGTTACTCAAGCCGACATTCTCGCTTCCGCTTCGTCCACCGCCGCTCGCGCGGTTGCTTCCCCCTAAGGCGGAACGCTCCCCTACCGATGCATTTTGACATCCCACAGCTTCGGCAGATCGCTTAGCCCCGTTCATCTTCGGCGCAAGAGCGCTCGATCAGTGAGCTATTACGCACTCTTTCAAGGGTGGCTGCTTCTAGGCAAACCTCCTGGCTGTCTCTGCACCCCTACCTCCTTTATCACTGAGCGGTCATTTAGGGGCCTTAGCTGGTGATCCGGGCTGTTTCCCTCTCGACGATGAAGCTTATCCCCCACCGTCTCACTGGCCGACCTTGACCCCAGTTATTTTGAGGTCATATCTAGTATTCAGAGTTTGCCTCGATTTGGTACCGCTCTCGCGGCCCGCACCGAAACAGTGCTTTACCCCTAGATGTCCAGTCAACTGCTGCGCCTCAACGCATTTCGGGGAGAACCAGCTAGCTCTGGGTTCGAGTGGCATTTCACCCCTAACCACAACTCATCCGCTGATTTTTCAACATCAGTCGGTTCGGACCTCCACTTAGTTTCACCCAAGCTTCATCCTGGTCATAGATAGATCACCCAGGTTCGGGTCCATAAGCAGTGACAATTGCCCTATGAAGACTCGCTTTCGCTACGGCTCCGGTGGGTTCCCTTAACCAAGCCACTGCCTATGAGTCGCCGGCTCATTCTTCAACAGGCACGCGGTCAGAGCCCAGGGCTCCTCCCACTGCTTGGGAGCTTACGGTTTCATGTTCTATTTCACTCCCCGATGGGGGTTCTTTTCACCCTTCCCTCACGGTACTACTTCGCTATCGGTCACCCAGGAGTATTTAGCCTTGCAAGGTGGTCCTTGCTGATTCACACGGGATTCCACGTGCCCCATGCTACTCGGGTCAGAGCATAAGCTAGTGATGCTTTCGGCTACTGGACTCTCGCCATCTAGGGTGCGGCACTCCACCGCTTCGCCTAGCAGCACGACGCTTTTATTGCTCTCCCACAACCCCGTTTTCACGGTTTAGGCTGCTCCCATTTCGCTCGCCGCTACTACGGGAATCGCTTTTGCTTTCTTTTCCTCTGGCTACTAAGATGTTTCAGTTCGCCAGGTTGTCTCTTGCCTGCCCATGGATTCAGCAGCAGTTCAAAAGGTTAACCTATTCGGGAATCTCCGGATCTACGCTTATTTTCAACTCCCCGAAGCATTTCGTCGCTTACTACGCCCTTCCTCGTCTCTGGGTGCCTAGGTATCCACCGTAAGCCTTTCCTCGTTTGAACCTCGCTCTTAACTTTAAGGCTATGCCATCCTAAGGTGCTGCTAAATGGAAAGATCTTATCAACGTCCATGAATGAAAAATAATAGATCGAACTGCCGAATCGGAAAAATTGGGTGCTATCATATAGCTTTGTATCGGCTAAGTTCACGAGTTGGAGATAAGCGGACTCGAACCGCTGACATCCGCCACAGGGTAAACCACCGCCTATCAGGCCCCTGACTGATTCTACCATAGAGGCCAACGATAGACAAGAACTCCCCCCCGAACACAGCTTACAACTTTCATCGTACTGTGCTCTCCAAAGAGCAACTCTTCTCAAAATCTCAAAAGGTGATGAGTTGGAATCCCATTCCAACTCAGGATTCTTGTGGTTCGAGAGGATCCAGCTACAGGAGAACCAGGAACGGAGAGCTTTCCCCCCTTTTCCGCCCCAACTCTTTCGAATGCTGGTTTTAAGAATGAGTGATTGCCCTTCTCCGACCCTTACTGCCCAACCTGCGAGCGGACAGCTAATGCATTCCACTTATTGAACAGGGTTCTATGGTCGGTCCGCGACCCCTGGATACCGAAGGCGTCCTTGGGGTGATCTCGTAGTTCCTACGGGGTGGAGACGATGGGGTCGGTCCATGGATTTTCCTTCCTTTTGCCGCATTTCGCTCAAAGGGTTGAAGGGAGATAGTGCATCAAGCTGTTCGCAAGGGCCAACTTGATCCTCTTCCCCAGGGATCAATCCCAGACGAGGGAACCCTAGGAGAGCCGCCGACTCCAACTACCGTCCATGTACGATCCATACTAGATCTGACCAACTGACCATCCTACCTCCTCTACGTTCTTGACAGCCCATCTTTGTCTCAGTAGAGTCTTTCAGTGGCATGTTTCGGTCCTTTTCCCCATTACTTAGAAAAAGTGAGCCACCGGTTCAGGTACAAGATACTATCATTACCGCCTGGACAATTAGACACCCAACCCGTAATCGCAACGACCCAATTGCAAGAGCGGAGCTCTACCAACTGAGCTATATCCCCCCGAGCCAAGTGGAGCATGCATGAAGGAGTCAGATCCTTCTTATATTCTTTTCCTTGGCGCAGCTGGGCCATCCTGGATTTGAACCAGAGACCTCGCCCGTGAAGTAAATCATCGCACCTATGGTCCAACCAATTGGGAGAGAATCAATAGATTCCTTTTCGGGAGCGATTCATCCTTCCCGAACGCAGCATACAACTCTCCGTTGTACTGCGCTCTCCAAGTGTGCTTGTTCCCCCCCTCTTCCTTACCATAGGAAGTATTTGTGAAATAACTTCGATGAGAAGAAAAAAGAGGGCGTTAAGAGACCCTACTGGCCTAACCCTAGACACTCTAAGATCCTTTTTCAAACCTGCTCCCATTTCGAGGCGGAAAGGAAAAAGAATTTCACGTTCTTCCTTTCGGGAAGGGAGGATTAGGAAAATCCTATTGATTGCAGCTTTCTCCAGACCCCTGGGAAAAGCATGAAAAAAAGGCTCGAACGGTACGATCCCTCCGTCACCCCAGAATGAAAGGGGTGATCTCGTAGTTCTTGGTCTGTGAAGATACGTTGTTAGGTGCTCCATTTTATTTTCCCATTGAGGCCGAACCTAAACCTGCGCTCGAGAGATAGCTGTCCATACACTGATAAGGGATGTATGGATTCTCGAGAAGAGAGGAGCCGTGGTGGTCCCCTCCGGACCGCCCGGATCCCACGAGTGAATAGAAAGTTGGATCTACATTGGATCTCACCTGAATCGCCCCATCTATCCTCCTGAGGAGAAGTTTGGTTTCAAACCCCGGTTCGAACAGGAGGAGTACGCCATGCTAATGTGCCTTGGATGATCCACATCTCAGGGTCAGGCGCTGATGAGCACATTGAACTATCCATGTGGCTGAGAGCCCTCACAGCCCAGGCACAACGACGCAATTATCAGGGGCGCGCTCTACCACTGAGCTAATAGCCCGTCGTGCGGGCCTCCTGCTGGGGGCCCGCTATGCCAAGCCAAAAGCGAGAGAAACCCCATCCCTCTCTTTCCTTTTTACGCCCCCCTGCCGCCACACGAGAGGGACATGGGGGCGTAAAAGGGGATCCTATCAACTTGTTCCGACCTAGGATAATAAGCTCATGGGCTTTGGGTTTGAAGCTGTGTCAAACCTAAATACCCAAGAAGCATTAGCTCTCCCTGAAAAGGAGGTGATCCAGCCGCACCTTCCAGTACGGCTACCTTGTTACGACTTCACTCCAGTCACTAGCCCTGCCTTCGGCACCCCCCTCCTTGCGGTTAAGGTAACGACTTCGGGCATGGCCAGCTCCCATAGTGTGACGGGCGGTGTGTACAAGGCCCGGGAACGAATTCACCGCCGTATGGCTGACCGGCGATTACTAGCGATTCCGGCTTCATGCAGGCGAGTTGCAGCCTGCAATCCGAACTGAGGACGGGTTTTTGGAGTTAGCTCACCCTCGCGGGATCGCGACCCTTTGTCCCGGCCATTGTAGCACGTGTGTCGCCCAGGGCATAAGGGGCATGATGACTTGACGTCATCCTCACCTTCCTCCGGCTTATCACCGGCAGTCTGTTCAGGGTTCCAAACTCAACGGTGGCAACTAAACACGAGGGTTGCGCTCGTTGCGGGACTTAACCCAACACCTTACGGCACGAGCTGACGACAGCCATGCACCACCTGTGTCCGCGTTCCCGAAGGCACCCCTCTCTTTCAAGAGGATTCGCGGCATGTCAAGCCCTGGTAAGGTTCTTCGCTTTGCATCGAATTAAACCACATGCTCCACCGCTTGTGCGGGCCCCCGTCAATTCCTTTGAGTTTCATTCTTGCGAACGTACTCCCCAGGCGGGATACTTAACGCGTTAGCTACAGCACTGCACGGGTCGATACGCACAGCGCCTAGTATCCATCGTTTACGGCTAGGACTACTGGGGTATCTAATCCCATTCGCTCCCCTAGCTTTCGTCTCTCAGTGTCAGTGTCGGCCCAGCAGAGTGCTTTCGCCGTTGGTGTTCTTTCCGATCTCTACGCATTTCACCGCTCCACCGGAAATTCCCTCTGCCCCTACCGTACTCAAGCTTGGTAGTTTCCACCGCCTGTCCAGGGTTGAGCCCTGGGATTTGACGGCGGACTTAAAAAGCCACCTACAGACGCTTTACGCCCAATCATTCCGGATAACGCTTGCATCCTCTGTATTACCGCGGCTGCTGGCACAGAGTTAGCCGATGCTTATTCCCCAGATACCGTCATTGCTTCTTCTCTGGGAAAAGAAGTTCAGGACCCGTAGGCCTTCTACCTCCACGCGGCATTGCTCCGTCAGGCTTTCGCCCATTGCGGAAAATTCCCCACTGCTGCCTCCCGTAGGAGTCTGGGCCGTGTCTCAGTCCCAGTGTGGCTGATCATCCTCTCGGACCAGCTACTGATCATCGCCTTGGTAAGCTATTGCCTCACCAACTAGCTAATCAGACGCGAGCCCCTCCTCGGGCGGATTCCTCCTTTTGCTCCTCAGCCTACGGGGTATTAGCAGCCGTTTCCAGCTGTTGTTCCCCTCCCAAGGGCAGGTTCTTACGCGTTACTCACCCGTCCGCCACTGGAAACACCACTTCCCGTCCGACTTGCATGTGTTAAGCATGCCGCCAGCGTTCATCCTGAGCCAGGATCGAACTCTCCATGAGATTCATAGTTGCATTACTTATAGCTTCCTTCTTCGTAGACAAAGCTGATTCGGAATTGTCTTTCATTCCAAGTCATAACTTGTATCCATGCGCTTCATATTCGCATGGAGTTCGCTCCCAGAAATATAGCTACCCCTACCCCCTCACGTCAATCCCACGAGCCTCTTATCCATTCTTATTCGATCACAGCGAGGGAGCAAGTCAAAATAGAAAAACTCACATTCATTGGGTTTAGGGATAATCAGGCTCGAACTGATGACTTCCACCACGTCAAGGTGACACTCTACCGCTGAGTTATATCCCTTCCCCCATCAAGAAATAGAACTGACTAATCCTAAGTCAAAGGGTCGAGAAACTCAAGGCCACTATTCTTGAACAACTTGGATTGGAGCCGGGCTTTCCTTTCGCACTATTACGGGTATGAAATGAAAATAATGGAAAAAGTTGGATTCAATTGTCAACTACTCCTATCGGAAATAGGATTGACTACGGATTCGAGCCATAGCACATGGTTTCATAAAACCGTACGATTCTCCCGATCTAAATCAAGCCGGTTTTACATGAAGAAGATTTTACTCAGCATGTTCTATTCGATACGGGTAGGAGAAACGGTATTCTTTTCTTAAACTTCAAAAAATAGAGAAATCAGAACCAAGTCAAGATGATACGGATTAATCCTTTATTCTTGCGCCAAAGATCTTCCTATTTCCAAAGGAACTGGAGTTACATCTCTTTTCCATTTCCATTCAAGAGTTCTTATGTGTTTCCACGCCCCTTTAAGACCCCGAAAAATTAACAAATTCCCTTTTCTTAGGAACACGTGCGAGATAAAAAAAAAAAGAGAGAATGGTAACCCCACGATTAACTATTTCATTTATGAATTTCATAGTAATAGAAATACATGTCCTACCGAAACAGAATTTGTAACTTGCTATCCTATAATCTTGCCTAGCAGGCAAAGATTTCACTCCGCGAAAAAGATGATTCATTCGGATCAACATGAAAGCCCAACTACATTGCATTGCCAGAATTCATGTTATCTATTGGAAAGAGGTTGACCTCCTTGCTTCTATGGTACAATCCTCTTCCCGCTGAGCCTCCTTTCTTCCGTGATTAACTGTTGGCACCAGTCCTACATTTTGTCTCTGTGGACCGAGAAGAAAGGACTCACTGCGCCAAGATCACTAACTAACACTAATCTAATAGAATAGAAAATCCTAATATAATAGAAAAGAACTGTCTTTTCTGTATACTTATGTATACTTTCCCCGGTTCCGTTGCTACTGCGGGCTTTACGCAATCGATCGGATCATCTAGATATCCCTTCAACACAACATAGGTCGTCGAAAGGATCTCGGAGACCCGCCAAAGCACGAAAGCCAGGATCTTTCAGAAAATAAATTCCTATTCGAAGAGTGCATAACCGCATGGATAAGCTCACACTAACCCGTCAATTTGGGATCCAATTCGGGATTTTCCTTGAGGGATATTGGTAAGGAATTGGAATGTAATAATATCGATTCATAATGGATTCATATCGATACAGAAGAAAAGGTTCTCTATCGATTCAACAAGTGCTGTACTTATGGGAAAGCGATAGAGAAAGAGAAAAAAAAAAACGAAGATTTCACATAGTGATTTTTTTTTGATCAAAAAAAAATATGATTGAATTTATTTCGTACCCTTCGCTCAATGAGAACATGGGTCAGATTCTATAGGATCAAACCTATGGGACTTAAGAATGATGGAAGGGAATAAAATCAAAAAAGAAATCAAATAAAGAAAAGAGAGGGAAAATAAAGAAATAATAAGTAAATAAAAATGAAGTAGAAGAACCCAGATTACAAATGAACAAATTCAAACTTGAAAAAGTCTCTTTCTGATTCTCGAAGAATGAGGGGCAAAGAGATTGATCGAGAAAGATCTCTTGTTCTTATTATAAGATCGTGTGATTGGACCCGCAGATGTTTGGTAAAAAGAATAATCTTATCCTTTGAGAATAATCAAAAATAGAAAGTGTTCAATTGGAACATGAAAACGTGACCGAGTTTATCCTAGTTACTCTTCGGGACGGAGGAGATTCGCGAACGAGGAAAGGGACCCAATGACTTCGAAAGAATTGAACGAGGAGCCGTATGAGGTGAAAATCTCATGTCCGGTTCTGTAGAGTGGCAGTAAGGGTGACTTATCTGTCAACTTTTCCACTATCACCCCCAAAAAACCAAACTCTGCCTTACGTAAAGTTGCCAGAGTACGATTAACCTCGGGATTTGAAATCACTGCTTATATACCTGGTATTGGCCATAATTTACAAGAACATTCTGTAGTCTTAGTAAGAGGGGGAAGGGTTAAGGATTTACCCGGTGTGAGATATCACATTGTTCGAGGAACCCTAGATGCTGTCGGAGTAAAGGATCGTCAACAAGGGCGTTCTAGTGCGTTGTAGATTCTTATCCAAGACTTGTATCATTTGATGATGCCATGTGAATCGCTAGAAACATGTGAAGTGTATGGCTAACCCAATAACGAAAGTTTCGTAAGGGGACTGAAGCAGGCTACCATGAGACAAAAGATCTTCTTTCAAAAGAGATTCAATTCGGAACTCTTATATGTCCAAGGTTCAATATTGAAATAATTTCAGAGGTTTTCCCTGACTTTGTCCGTGTCAACAAACAATTCGAAATGCCTCGACTTTTTTAGAACAGGTCCGGGTCAAATAGCAATGATTCGAAGCACTTATTTTTACACTATTTCGGAAACCCAAGGACTCAATCGTATGGATATGTAAAATACAGGATTTCCAATCCTAGCAGGAAAAGGAGGGAAACGGATACTCAATTTAAAAGTGAGTAAACAGAATTCCATACTCGATTTCATAGATACATATAGAATTCTGTGGAAAGCCGTATTCGATGAAAGTCGTATGTACGGTTTGGAGGGAGATCTTTCATATCTTTCGAGATCCACCCTACAATATGGGGTCAAAAAGCCAAAATAAAAGATTTGAGCCCTTATAAAAAGAAAACAGATTCTTGAACCCCTTTCACGCTCATGTCACGTCGAGGTACTGCAGAAGAAAAAACTGCAAAATCCGATCCAATTTATCGTAATCGATTAGTTAACATGTTGGTTAACCGTATTCTGAAACACGGAAAAAAATCATTGGCTTATCAAATTATCTATCGAGCCTTGAAAAAGATTCAACAAAAGACAGAAACAAATCCACTATCTGTTTTACGTCAAGCAATACGTGGAGTAACTCCCGATATAGCAGTAAAAGCAAGACGTGTAGGCGGATCAACTCATCAAGTTCCCATTGAAATAGGATCCACGCAAGGAAAAGCACTTGCCATTCGTTGGTTATTAGGGGCATCCCGAAAACGTCCGGGTCGAAATATGGCTTTCAAATTAAGTTCCGAATTAGTGGATGCTGCCAAAGGGAGTGGCGATGCCATACGCAAAAAGGAAGAGACTCATAGAATGGCAGAGGCAAATAGAGCGTTTGCACATTTTCGTTAATCCATGAACAGGATCTATATAGACACATAGATCCGTGGATCCATACATCTCGATCCGAAAAGAATCAATAGAAAAAGAAAAAATCGGAATTGATCGATCTCTTTCTCGAAACAAACGAAAAGGAAAGAAAAGACGAAACATAAATCATGGATCAACTAAGCCCTCTCGGGGACTTGCTTAAGAATAAGAAAGAGCAATCTCATGTAAATACCATGGAATAAGGTTTTAACCTATTCATGGGGATTCCGTAAATATTCCATTCAAAAAAAAAAAAAATTGGTTTTTTTTTGGAGATTGGATGCAGTTACTAATTCATGATCTGGCATGTACAGAATGAAAATTTCATTCTCGATTCTACGAGAATTTTTATGAAAGCCTTTCATTTGCTTCTCTTCGATGGAAGTTTTATTTTCCCAGAATGTATCCTAATTTTTGGCCTAATCCTTCTTCTGATGATCGATTCAACCTCTGATCAAAAAGATATACCTTGGTTATATTTCATCTCGTCAACAAGTTTCGTAATGAGCATAACGGCCCTATTGTTCCGATGGAGAGAAGAACCTATGATTAGCTTTTCAGGAAATTTCCAAACGAACAATTTCAACGAAATCTTTCAATTTCTTATTTTACTATGTTCAACTCTCTGTATTCCTCTATCCGTAGAGTACATTGAATGTACAGAAATGGCTATAACAGAGTTTCTGTTATTCGTATTAACAGCTACTCTAGGAGGAATGTTTTTATGTGGTGCTAACGATTTAATAACTATCTTTGTAGCTCCAGAATGTTTCAGTTTATGCTCCTACCTATTATCTGGATATACCAAGAAAGATGTACGATCTAATGAAGCTACTATGAAATATTTACTCATGGGTGGGGCAAGCTCTTCTATTCTGGTTCATGGTTTCTCTTGGCTATATGGTTCATCCGGGGGAGAGATTGAGCTTCAAGAAATAGTGAATGGTCTTATCAATACACAAATGTATAACTCCCCAGGAATTTCAATTGCGCTTATATTCATCACTGTAGGAATTGGGTTCAAGCTTTCCCTAGCCCCTTCTCATCAATGGACTCCTGACGTATACGAAGGAGTGCGGTTCGTTTGAGAAATTCCTACCTCTCTATCTATCTCTGAGATGTTTGGATTTTTCAAAACTCCATGGACATGCAGAAGAGAAATGCTATCCCCACGCAGACCAAGACAGAACTTTGACTTGTTCAAATAACAATTAATGTGAAGCAGGGTCAGGAACAACGAATCTCTTTATGATAAACGGATCCATTTTGCAAGTTTGTTATTACGGGTAGTTCCTACAAAGGATCGGACTAATGACGTATACAAGAAAGACTTGAATTCTCGATGTAGATGCTACATAGTTGGTTCTCATCCTTCAGAGACTACGAGTGTAATAGGAGCATCCGTCGACAAAAGGATCACCCTAAGATGATCATCTCATGGCTATTGAGAACGAATCAAATCAGATGGTTCCATTTCTCAATCTTTCGGACGTGCTCCTACGGAACCAAGGTCGAAACGATTGAGAAAAATCAGTCATTCACAACCACTGATGAAGGATTCCTCGAAAAGTTAAGGATTAGTCATCCGTTTTAGAAAGGATTCGATCTTATACATACGCGAGGAAAGTAATCAAAAAAGAAAGAAGATGAGTTCTTCTTTACTTTTATCACTTAGGAGCCGTGCGAGATGAAAGTCTCATGCACGGTTTTGAATGAGAGAAAGAAGTGAGGAATCCTCTTTTCGACTCTGACTCTCCCACTCCAGTCGTTGCTTTTCTTTCTGTTACTTCGAAAGTAGCTGCTTCAGCTTTAGCCACTCGAATTTTCGATATTCCTTTTTATTTCTCATCAAATGAATGGCATCTTCTTCTGGAAATCCTAGCTATTCTTAGCATGATATTGGGGAATCTCATTGCTATTACTCAAACAAGCATGAAACGTATGCTTGCATATTCGTCCATAGGTCAAATCGGATATGTAATTATTGGAATAATTGTTGGAGACTCAAATGGTGGATATGCGAGCATGATAACTTATATGCTGTTCTATATCTCCATGAATCTAGGAACTTTTGCTTGCATTATATTATTTGGTCTACGTACCGGAACTGATAACATTCGAGATTATGCAGGATTATACACAAAAGATCCTTTTTTGGCTCTCTCTTTAGCTCTATGTCTCTTATCCCTAGGAGGTCTTCCTCCACTAGCAGGTTTTTTTGGAAAACTCCATTTATTCTGGTGTGGATGGCGGGCAGGCCTATATTTCTTGGTTTCAATAGGACTCCTTACGAGCGTTCTTTCTATCTACTATTATCTAAAAATAATCAAGTTATTAATGACTGGACGAAACCAAGAAATAACCCCTCACGTGCGAAATTATAGAATATCCCCTTTAAGATCAACCAATTCCATCGAATTGAGTATGATTGTATGTGTGATAGCATCTACTATACCAGGAATATCAATGAACCCGATTATTGCGATTGCTCAGGATACCCTTTTTAGCTTCTAGAATCTATTTCTTAGTTCAAGATCCCTCTTACTAACTGGAATCAAAGAATTAGTAGATCGGTTCCGCCCAAAATGGGAATGGACTAAGGTTATGAACTTATAATCTATAATCTGATGATCGAGTCGATTCCATGATTATAAGTTCATTCCATACCGGACCAGACCGGAATAAGGTTATATACATTCTCATTATGAGAAGGGGTCATTCGAGCGTATCTAAATAGATACTATGTTTACATAGGGATCCCTACGTCGTTACATTCCATTTAGGATTAGGAATAGGCGAAATCTGACCTACTTTTTACATATCTCTCGTTATTTGGGACCCTATTCACCTCTTTGGTTGGACTTCTATTGAATCGAGAAATAGGTTTGATTGTCCATCTTTTTGATATAATATTAATATATATATAAGGCATCCTCCGGATAAGGATAATTAAAATCTAAGCAATTAGATGTCCGACTCGGGCCTATATGACATGACCGATCAATAGAAATACTTCAACACTCCACCTTTGTCATATATTCAATACACCGTACTAGATAGATATCATATTTATGGAATACGATTCACTTTCAAGATGCCTTGGTGGTGAAATGGTAGACACGCGAGACTCAAAATCTCGTGCTAAAAAGCGTGGAGGTTCGAGTCCTCTTCAAGGCATAATATTGAAATGGAATAAGTTCGGCAGCGGATCGCGAAATCTTGGCGATCTTCTCTATCTAATGAATGGGGAGGGGGAGTCCGCTTTGAAATCGTCCGCCCTGCGCCCCGCAGTATATGATTCAACAGGAATCACACAAGGGTAGATTGATACAATCTAAACCTCTGGTAAAATGCCCCCGTAACCCAGCAGATAAAGTACATAGTCCGTTTTAGGGATTGGTGACTTACCCATTCAGTGACTTTGGCACTGGATGGACGTTACCAAAATTGGTACTATCGGGTCGGGTGAATTCAATAATAGACGCCTGGCGGCATTCCAGCCTTCCTTCTCCTTTCAGGACCTATCCTAAACAGAATCCAGTACTTCTTGGTCGTGAATATCTGAATAGGGCGAACCACTCCGTGGATATCTTTACTTCGGAACAAAACAATTAGAATTAGGCTCGGTCAACTGGAATGTGTATTATCCATATAGGGGATCTTCCAATTGAGAAGATCTATCGACCTGAGACGAAGAGAAAGGTCTATCTATTTTATTTAGTTATTCAGTTGATTCGTTATTGGAACAGATAGCAACAACAATTTCATCCGACATGCGTATTTTTGATTTTCCAATGGATTTCCATCCTTCATTAATGGAAATTTTTTTGATGTAGTGAGTAATAGCTCTGGTTGTTCGCTGTTCAAGAATTCTTGTTTAGGCAGTTCGTACCATCCATACATAGTGTTTTGATCTAAGATTTCAATTCTTCCATGTTTCCGTCGTAGCATATTGTTCCATGGAGCTAAGTGGAAGAAACAGGTGTTTCTACAACTCTACCACCCAGTCAATTCCGTTCCACTTAATCCCTATTTCATGGACACATATCTTTCCGGCTAAGTAATGGGAAACCTTTCTCCTGTTACATTACATGAATCCTATTTTCATTTCATCCGGAAAAAGCCATCTTTTTTTCAACAATGTCTTTGTCATTCGATCCACTAGCGTTCCGTTAGATAGGAACAGATTTGATAAATACTGATAACTCTCGGATAGAGTATTAGAACGGAAAAATCCATTAGATAATGAACTATTGGTTCTAAGCCATCTCTGGCGCTGAATCAACAATTCGAAGTGCTTTTCTTGCGTATTCTTGATAAACCAGCGTTTATATATAGATGTAGGAGGATCTGTTTGGGAAGTAAGAAGCCCCTTTGACATCTCTTCATCTGCAAAGAATTCTCGATGTGAAAACACAGAGACAAAGGGCTGATCTTTGAATAGGAAAAAGAGTGGATCTGCGGGGTCCCAAATGAATTGGCTTATTCTAAAAAAGCCTTGTTCTTTGGAAGACCTATCTCGTCTCTGGTACTGCATGGTTCCGCTCTGCAAGAACTCCGAATCATTCTCTTGAAGCTCATACTTTTCATCATAAATGATCCGCTTGCCCCGAAATGACCCGGCCAAATAGGGAAATCCCAATTCATTAGGCCTTTCGATACAATCAAATAGAAAGCCCCGAGGGCGCCATATTCTAGGAGCCCAAACTATGTGATTGAATAAATCCTCCTCTATCTGTTCCGGGTCGAGGACTGCTTCTCCTTCCCCTTCTTCAAACTCCGATTCGTATTTTTCATAGAGAAATCTCTGATCAACGATAGAACAAGATCCATCTTGCATCATATATAAGGGATCCCTTGGTTCGGAGCGAAAAAGCAATGTCACTCGATCATTATCAAACTGACTGCAATCTTTTTCTGTCCGTGAGGATCCCACCAAAGCGCCTTGCACTTCTAATAGGCCATGAAATAGATCCGAATCATTCTCAATGAATCCATAAGAAGTGATCCTATTTTTTTCATCGGGTCCGGGTAGAGACCAAAGGTCTTGAGCGACCGATCCGGCAGAACAACTCAAAAGATAAAGAAGTATCGTGAATTTCTTCATGCTCGTTCCAAGTTCGAAGTACCATTTGTACAAATAAGAATCCCCTTCGTTACATGATTTCTTCTTCATATAGATAGATATAGGATCTATGGGGCAATTACTTATAAGTACATTTTGTGCAACAACCCTTCCTATCTGATAGAAAAGGATCCCATGATCCTGAACCGATCTTACCTGGGATCGCAAATCCCAAGTTTGTCTATGAAGAGCAGATCTAATTGTATTAGTGTCTATAATTGATTTCTTCTGTGTAATACTAATTGATAAGGCCTCATTGGTAAGTGCTACAAGATCTCGTGCACTGGAACCCATGGTTATGGACTCGAATCCATTAGTATGGAACATTTTCTTTTCCAAGTGAAATCCCCTAGTATAGGAAAGAGTGAAAAAGTGCTTTCGTTGTTGTGGAATAAGAAGCCTTCTTATTTTAATGCATGTATTTAATTTATTCGGGGCTATTAGAGCGGGATCCACTTTTTGGGGAATATGAGTCGAAGCAATAACAAGACTATTTCTAGTCGAACATCTTTCACAATCCCTGGAGAGAGAGTTCACCAAGAGACCGAGGGCTAAGTAATTCGACTCATTCACATCAAGATCATGAATGTTTGGAATCCATATTATGCAAGGAGACATTGCTTTTGCTAATTCGAATTGAAGGGTGATATAAAATCGGTCTATTTCCGACATCATATCCATAGTTAGCGCATTCATCATAGTTAGAAGCTCCAGCTCCGTATCAAGTTCACGATCAATATCGTTACTAGCATCAATATCGTCACTATCATCAATATCGATATCATCAAGAAAAAAACCTTTCGGCTTGTTATCCAGGAACTTGTTCAGACATACTGTAATGAAAGGAACATAGGAGTTTGTCGCTAGGTATTTGACCAAATAGGATCGTCCGGTTCCTATAGAACCTATCACTAAAATACTCCTAGAGGGGGATAGGGCTAAGCGGAGCGAAAAGGGTTTTCCATGAGACGGGAAATGAAAACTATTAGCCCCACACGAAGTTTGTGAATAAGTGATTGTCTGATAATGAGCAAGGAATATCCGTCTTTCTGCTAAACAGGATGTATTGAACTCATAATTCATTAGATACTTTTTATGAATGTCAACTAAGTATCGTAAGTAAATTGTTCCCGGTTGTTCAATCATTTGATAACCAGAGTCATTCTTTGATAAATGATCACTATGAGTCAGACTCAATAGAATTTGATCAATCCTTTTTTCTGCCCTTAAGGTGGAGAACTGAACCAAGAATTCTCTTTCTTTATCATCAATCGAATCACTGTTCGCGACCCAGGATTCTATTTTATCATCAATCCAATCACCGCTCACGTTTTTTCTTTTTCTTATCAATGAATAGATGTCTTTACTTGTATGACTTAGATGTCTCGTATTTCTCGAAAAAGTGATTCGATTGATGGGATTTGGTATGATACTTATGAGATCGATGATATCGATGAAGTTTATTTTCAAATCTGTCTTCTTAGAACGTATTGATTTGACCCCATAAGCGGGATCACCACCCCATAGCATGTTGCCGCCAGAACCCCGTATTTCTTCTAGACAATCTCCTAATTGTTCCAGAGCAACTAGAAAAAGATTCTTTAACCAGAAAGAATTCTGTTCAGATGTAGGATACCTATCCAGAAGTTTTCGCAACTCAATCATGTATGATGGAATCATCAAAGATTTGATCTTTTCGAACTCTGTCTGTAACTCACTATAGGCTCGGGAAACAAAGAGAAGATGTGTACGAACGATATATCCAGCAACAAGAAGAAGGAAAAGGATTGAATAGAGGACCTCACGAACATTTGGCGATCTCAGATGTGTCGATATCAACGATGACTCATTATTTCGATGAATCATTTCTTCGGACAGAAGAAGATTATGTAAAGACTTACTCGAAATCTCACTTATCAGATTCCTTTGTGGAAGACACAATTTTTTCTGAAGAATTCGCCATGATATATCTAATCCATACATAATATCATGAAAAATGGATACAAATTTTTGACTGCTACTTAGTATCCGCAATAGGTCTGAAAAAATATCTAAAAATATCAAATTTAGATATTTGTACCCTGTCGAAGTAAAGAACCATGGCATATATGTTTGGAATAGATTCCATTTTGAGAGAGTTGAAAAAGCACTATCTCGTTGAAAGGTTCTATCCATCTGCCCTTTGTCAACGCATTTTTTTAGGCAAAGACTCCGTTTTTTCCTCTGTAAATATTTCTCAGAACATGGAGTGTGAATCAAACCCACGTTTGAATTGAAATTGAGATACTGATGCAAGCTCTTCTCTTCTGAATCGGATAGATTCATATCTGAAAGAGTTTGACAATACGTTCTTTCCAAATTTACTCTTTGTCCCTCTATTAGAGGTGTTCCAGAAATGTCTGCAATCGAGTAAATAGCTCTACGAACTAATGGATCGGATCGAATTGGAAAATGGAAAGATTTGTACAAGTTATACCTTTCGTCACCACTTTGTGGAAAATCGTTAGATATGAATATGTTAGATACCTGTGACTCGATTGACGAAGGTGAAATAGTATCTCTCTCCAAAAAAGCATGTTTTTTTTTACCACCACACGAAGAAAATATTTTGTTGTGAATGAACAAGATAGTGAGGAATTGTCCATACGTAAAATCAGAATTATTGAGACGGGCCTTTTCCACATAAAAAGGGAATCTTTTGTTACAATAGAAGCAGAAGTGATGTGGATTATTCAAGAATCGAAGTCGATTTGCTTTAGAAAAAGAAGATATCAATGAACTTCTCTGAAATGGTTTCACGGGATTCAGCCAATTGTCTTGATCGTGGGATACGATTGAGAAATAGGAATCCGTGTTATCAAAAGATTTCCTGCGATTCTTTCTAGTATGGAATGAGTCAATCATCCACTTTGGTATCTTATTGAACAAAAATGGTGATATTGTTCCTCCATTGATCAAGAATTTCGATTTTTGAGAAGTATTATGATCATCCAATAAAAAGGGTTTCAATTTTTTAAAATGAACGATTTGAAGACCTATTGATTCTAACAACTGATTGCAGGGTTGATCGTTCGGACCTTTCAATTCATAGATGTGGATCTCAGACCTATGAATGGGGATATTCTCGAAACTCACAAAGAAAAAAGGAAGTGAATTAGACAAAAAGAGAAGTAACTTGGACAAAAAACGAAGTAACTTGGACAAAAAGAAACGAAGTGACTTAGACAAATCTTTTTTATCAATAACCTCAGACCAATCAATCGAATATTGATTAATACATAATCGATCGAACACTACTTGAAAACGGCTCTTCCGCTCAGAAACGAAATGTTTCAAATGCTCCTGGAAATTCTTGCTCCCATTGGACCATTTGTATCTATATGCATTAGGATCCCGATTTATGGATCTCTCGGTTCGAGAAAGAAAAATAAGAGGATCGAACCATTTCTTCTGACTCTTTTTCAAATTCGATAAATGTTGGTTGATCGTATCTTTCATTATAGTTCTATGATTCAGAGTATCATTTCCTATTAGATCCCTTTGAATTCCATATTCGAAGTTGCGATCAGGTCTCTTCATTAAAAAGAATCGATTCAATACATTTCTTATGTACCCATAGGGACTATATTGGAATTGGATTTGAATCAGATTTCGGATCAATCTATATTGATTGACTGCCTCCATTATGTTGTTGCTAGCAAATACCACTCTTTTTGGTTTTGGATCTTCAAAAAAATTCCCGCAGGAGATCCGGACCCAATTTTTTCTGATCCTTCGATAAAAAGATTCATTTTCTTCATAAAAAATAGGAGGTAGAACCAATAAAGATTTCTTTTTCAATTCATCCCTGGAGTTGAAAACCTCCTTCAAGAATTGTCTTTGATCCAATCCGTAGGAATCAATAGAAAAGGCAAATCCCGTATGATACACCAGATCCGGCTCGGTTATTGATAGAGTGAATAGATCTGCCATTTCTTGAAATCTCTCTTCTGACTCAAAATCGTGGCGTAACGTGTATCCCCCCCTCTTCCGTTCATGGAATAGATGAAATAAATAAAAAAATGGATTTTTGTTCAAGAATGAAATCTTATTGGAACTGTCCATATCCAGTTCATCCTTCGGAACCGTATCACATCCCAGATCTGATGAAATAGGATGAATTGAGACGGTATTTTGTAAATACGTAATTATCTTGAATATATTAACTATTTCTTTATTTTCCGATCGCCTGGAAGGGACAAAAGAAACATCTTGTTCTTTCTTCAACAATTTCTGATCCCTAGTGGACCTCTCAGTAGGATTCGAACCCAGATGAAGTTCTGACCATCTGTCAGAGAAAAAAGAACGAATGGCTCTTGTAGAATTCCAAAAAAATTCTTCGCTTTCTTCCGGAAGCAGATGATTATTCATTCGCTTTTCACGTTCCGTGAATAGCCGGGGCATTGAGGAATATCCAGAAAGGTATTTAGGGAATCGGTCTGATTCTATCTCTCTTCCTTCCGTTTGAATAAAGGAAGGATCCCAAAGAATCGATCTTTCTTTTAGTTGTTGAATCTCTCTTTGATTGATCAATGTGTGATAGTGATATTCCGAATCCTCATTACTAATGGAATCGAAAGGATCTATGAATTGATCAGAAGATCCGTTCAATTGGCTAGAATCCGTTACTTGAACGAAACTAGATCTTGTAGAATCATATTGAATATTTGACGATACATTTCGTACCTTGCTAAAAAATCTATCCTTGTTTACCAACCACACATTGTCTAACCAAATCCAATTCTCTCTCGATATTTTCCTCAAAAAATCCGATTCGTGCGGATTCTTCCCCCAACTAACGAAGAGATCTTGGTGGAATTGCCACATATGAAATTGAGCACAATTTTGCAAAGAAATAGCCCGCTTGTTTCTCGAGAAGAGATGGGAAACATGCTCAATATCATTTGATTGAATAGTTGACCCAGCTCCTTGTTGTTTGAAGAAACCCTCCACTTCAATTGGTATTTTTTCACGAAAAGCAAACATGAGATAACAAATCCAGTCTTTCACTAAGATTTCGAATAGCTGTCCCGAATTCAAGTTGATTATGTTTCGCCTCTTATTCGGAGAAAGACGATCAAACAATTCCCAATCATGGCCCTTGCGGATCGGATCATCCATATAATATACAAAAAGAAACTCCAGATATTTGATATCTTTCTCTTTAAATGAGATATCAATTCCAGCGACGGTTTCATTAGATATCTTACAACAAAAATCCCTCTTTTTTCCGATCCAGTTCCTCCACCACCGCGAACTCCAGTTAGATTCAGGCATGATACACTTTTTAGTTATTGGGAGAACCCGAGTACTCTCTTTCGGATCCCGGAAACAGCTCTCAGAGATCTTTTTTCCTTTTGTAAAATACAGGAGCGAAACAATCAACCTATTGATATTGGAAGACCCAAAAGATTCTTCCGATGTATCATTTCTGGGTCCAATGGAATTCATAGGTATAGGAAGAAGCCCTTTCAAATAGAGATTTTTGCTTTCGACCATATTTCGATTGTTAATACGATATAGAAGGGCCGCTACTACAAATAGTACTACACCCTTGATCGTGAAATATCGATTGCTTGTTGAACCCTGTGAATTGCGCAAAAGTAGGATACTAAAAATTCGAGGGTCCAAGAGTTTTCTAAAACGTTCTTGGTGGAAAAAAATATGAATGAAAGATCCCACTGAATTGATTTGGGTCCATGAATCTAAGAAATAGTGAGAATTCTTGATCTCTCTCACTATTTCTCTCAATTCGAAAATCCAGGATTTGAATTGATGTCCTTTCATTGATTCCTCCTAAATTGCATTGATTTATCCTAAAGATTTCATTTCAATTGGAATTTGGTTATTCACCATGTACGAGGATCCCCACTAAGCATCCATGGCTGAATGGTTAAAGCGCCCAACTCATAATTGGCGAATTCGTAGGTTCAATTCCTACTGGATGCACGCCAATGGGACCCTCCAATAAGTCTATTGGAATTGGCTCTGTATCAATGGAATCTTCTCATCATCTATACATAACGAATTGGTGTGGTATATTCATATCATAACATAACATATGAACAGTAAGAACTAGCATTCTTATTGAGACTAGAACTCATAGGGAAGAAAATCGATTTATGGATGGAATCAAATATGCAGTATTTACAGACAAAAGTATTCGGTTATTGGGGAAAAATCAATATACTTTTAATGTCGAATCAGGATCAACTAGGACAGAAATAAAGCATTGGGTCGAACTCTTCTTTGGTGTCAAGGTAATAGCTATGAATAGTCATCGACTCCCCGGAAAGGTTAAAAGAATGGGACCTATTCTGGGACATACAATGCATTACAGACGTATGATCATTACGCTTCAACCGGGTTATTCTATTCCACCTCTTAGAAAGAAAAGAACTTAAATCAAAATACTTAATAGCATGGCGATACATTTATACAAAACTTCTACCCCGAGCACACGCAATGGAGCCGTAGACAGTCAAGTGAAATCCAATCCACGAAATAATTTGATCTATGGGCAGCATCATTGTGGTAAAGGTCGTAATGCCAGAGGAATCATTACCGTAAGGCATAGAGGGGGAGGTCATAAGCGTCTATACCGTAAAATAGATTTTCGACGAAATACAAAAGACATATATGGTAGAATCGTAACCATAGAATACGACCCTAATCGAAATGCATACATTTGTCTCATACACTATGGGGATGGTGAGAAGAGATATATTTTACATCCCAGAGGGGCTATAATTGGAGATACCATTGTTTCTGGTACAGAAGTTCCTATAAAAATGGGAAATGCCCTACCTTTGAGTGCGGTTTGAACTATTTGATTTACGTAATTGGAAGTAACCAATTAGGTTTACGACAAAACCTAGAAATCGATCACTGATCCAATTTGAGTACCTCTGCAGGATAGACCTCAACAGAAAACTGAAGAGTAACGGCAGCAAGTGATTGAGTTCAGTAGTTCCTCATATAAAATTATTGACTCTAGAGATATAGTAATATGGAGAAGACAAAATTGTTTCAAGCACCGACAGAACCATAAGCGCCCCTTGTTTCAAAGAGAGGAGGACGGGTTATTCACATTTCATTTGATGGTCAGAGGCGAATTGAAAGCTAAGCAGTGGTAATTCTAAAGATTCCCCCGGGGAAAAATAGAGATGTCTCCTACGTTACCCATAATATGTGGAAGTATCGACGTAATTTCATAGAGTCATTCGGTCTGAATGCTACATGAAGAACATAAGCCAGATGACGGAACGGGAAGACCTAGGATGTAGAAGATCATAACATAAGTTATTCGGCAGATTTTGATTCCTATATATCCACTCGTGTGGTACTTCTACCATATATAGAAGAATTCTACGATATATATAAGATAAGATCCATCCGTATAGATATCATCATCTACATTCAGAAAGCCGTATGCTTTGGAAGAAGCTTGTACAGTTTGGGAAGGGGTTTTGATTGATCAAAAAGAAGAATCTACTTCAACCGATATGCCCTTAGGCACGGCCATACATAATATAGAAATCACACTTGGAAAGGGTGGACAATTAGCTAGAGCAGCGGGTGCTGTAGCGAAACTGATTGCAAAAGAGGGGAAATCGGCCACATTAAAATTACCTTCTGGAGAGGTCCGTTTGATATCCAAAAACTGCTCAGCAACAGTCGGACAAGTGGGAAATGTTGGGGTAAACCAGAAAAGTTTGGGTAGAGCCGGATCGAAATGTTGGCTAGGTAAACGTCCTGTAGTAAGAGGAGTAGTTATGAACCCTGTCGACCACCCCCATGGAGGTGGTGAAGGGAGGGCTCCAATTGGTAGAAAAAAACCCGTAACCCCCTGGGGTTATCCTGCGCTTGGAAGAAGAACTAGAAAAAGGAAAAAATATAGTGAGACTTTGATTCTTCGTCGCCGTAGTAAATAGGAGAGAAAATCGAATTTCTTTCTTCGTCTTAAAAAAAATAGGAGTTAATTAACTGTGACACGTTCACTAAAAAAAAATCCTTTTGTAGCAAAGCATTTATTAAGAAAA

# A66 chloroplast genome

ATAGAGAAGCTTAATACAAAGGCGGAAAAAGAAATCATAATAACTTGGTCCCGGGCATCACGGGCGAACGACGGGAATTGAACCCGCGATGGTGAATTCACAATCCACTGCCTTAATCCACTTGGCTACATCCGCCCCTACTATACTATACATCATATCTTGTTTGTATTGTCTAAAATAAAAACGCAGCAATATTTTTTTTGATAAAAAAAAAATTATAAATTATAATAGTATTTTTTTCTATTTTATATATAATAGAAAAAATTATATAAAAAAAATGATTTGTTCCGTTTTATAGAAAAAAACGAGCGATATAAGCCTTAAAAATAAGGCTTATATCGCTCGTTTTTAATATTACTAAACTAGGTCTAGACTAACACTAAAGAATTATCCATTTATAGATGGAGCCTCAACAGCAGCTAGGTCTAGAGGGAAGTTGTGAGCATTACGTTCATGCATAACTTCCATACCAAGGTTAGCACGGTTAATAATATCAGCCCAAGTATTAATAACACGTCCTTGACTATCAACTACTGATTGGTTGAAATTGAAACCATTTAGGTTGAAAGCCATAGTACTAATACCTAAAGCAGTAAACCAAATACCTACTACCGGCCAAGCCGCTAAGAAGAAATGTAAAGAACGAGAATTGTTGAAACTAGCATATTGGAAGATCAATCGGCCAAAATAACCGTGAGCAGCTACAATGTTGTAAGTTTCTTCTTCTTGACCGAATCTGTAACCTTCATTAGCAGATTCATTTTCTGTGGTTTCCCTGATCAAACTAGAAGTTACCAAAGAACCATGCATAGCACTAAATAGGGAGCCGCCGAATACACCAGCTACACCTAACATGTGAAATGGGTGCATAAGAATGTTGTGCTCAGCCTGGAATACAATCATAAAGTTGAAAGTACCAGAGATTCCTAGAGGCATACCATCAGAAAAACTTCCTTGACCAATTGGGTAGATCAAGAAAACAGCAGTAGCAGCTGCAACAGGAGCTGAATATGCAACAGCAATCCAAGGACGCATACCCAGACGGAAACTAAGTTCCCACTCACGACCCATATAACAAGCTACACCAAGTAAAAAGTGTAGAACAATTAGTTCATAAGGACCACCGTTGTATAGCCATTCATCAACGGATGCAGCTTCCCAGATCGGGTAAAAATGCAAACCAATAGCTGCAGAAGTAGGAATAATGGCACCTGAAATAATATTGTTTCCGTAAAGAAGAGATCCAGAAACAGGTTCACGAATACCATCAATATCTACTGGAGGAGCAGCAATGAATGCGATAATAAAAACGGAAGTTGCGGTCAATAAGGTAGGGATCATCAAAACACCAAACCATCCAATGTAAAGACGGTTTTCAGTACTAGTTATCCAGTTACAGAAGCGACCCCATAGGCTTTCGCTTTCGCGTCTCTCTAAAATTGCAGTCATGGTAAAATCCTTGGTTTATTTAATCATCAGGGACTCCCAAGCACACAAATTCTCTAAAACTATAAGTAGATAATTGAGAGCTTGTTATTGAACAGTATAACATGACTTATATAGCCATGTCAACCAATGTAAAATGGCTAAGATCCTTTTAGTTTAGATTCATAATAATTTTTTTATCGAGGAGAGAAATTATAAACGAATCTATATACATAGAAATATAATTTCTCTATGAATATTATTTCAAAATCATATGAATATGATCCATAGTGGGTTGCCCGGGACTCGAACCCGGAACTAGTCGGATGGAGTAGATAATTTCCTTGTTAAAATGAAAAAAAAAGTAAAAAACCCCTCCCCAAACCGTGCTTGCATTTTTCATTGCACACAGCTTTCTCTATGTATACATAGAAAACTCAGTTTCTTTGTTTCCTTATAAATAGGACTGCGAATTCAATACTCAGTAAATTTCATCTTAGTCTTACTGTATGAACATTTAATAATAGAAATAAATGACTTTTGATAATACAAAATAATTAATTTTTTTGTTATCTCCGCATTCCGTTTACGTTCTGATAAATTTTGATTTAATTTATGGAGCCTCAGAACCCCATTATTCATGATTGACTAAATCATTAAGATAAAGAATATCCAAATACCAAACCCGCACTCGATATAATCTTTTAGAAGCATAATCACTTCTTGGGAAGATTAAAGAAAGAACTTGGTCTTCCCCCGTAAGGAATTCTTCTAATAAACCCGAGCCCAACCTTTTTAAAAAAGCGCGTACAGTACTTTTGTGTTTACGAGCCAAAGTTTTAACACAACAAAGACGAAGTATATATTTTATTCGATACAAATTCTTTTTGTTTGAAGATCCGCTGTAATAATGCGAAATATTTCTGCATATACGCACAAATCGGTTGAGAATATCAGAATCTGATGAATCCGTCCAGGTCGCTTTACTAATCGGATGCCCTAATACATTACAAAATTTATCTTTAGCCAACGACCCAATAATAGAAGAAATTGGAATGTTGCTATCCAATTTTATTCTAACATTATCTATTAGAAATGAGTTTTCTAGCATTTGACTACGTACCACTAAAGGGTTTAGTCGCAAACTTGATAGATAACCCAGAAATTCTAAATTATCTTTAGATAATTGATTTATATTAACCTTTTGCGATTGAAACCATACGGAAAAATAACATTGCCATAAATTAACAAAATAATATTTCCATTTATTCATCAGAAGTGGCGTATCCTTTGTTGCCAGAATGTATTTTCCATGATATCGAACATAATGTAGGAAAGGATCCTTGAGCAACCCTAAGAGCGCCGAAAAATTATTAACAAAGACTTTAAAAAAATGTTGTATTTTTCCATAGAATAAAATTCGCTCAAAAAAGACGTCATAAGATGTCGATCGTAAATGAGAAGACTGCTTGCGTAGAAAAAAAAAGATGGATTCGTATTCACATACATGAGAATTATATAAGAACAATAAAAATCTTGGATTCAAAATTGATTTTTTTTTACTATCAAAATTCTTCCAATTGCAATACTCGTATAGACAGAACCGAAAAAAATGCAAAGAAGAGGCATCTTTTACCCGGTAACGTAGGGTTTGAACCAAGATTTCTAGATGGATGGGGTAAGGTATTAGTACATCTAATACATAATTAAAATGTGAGAGTTTGTCTTCTAAAAAGGGAAATATTGAATGAAGTGATTGTAAATTGTAAGATTTTTTTACATTTTTTCCTTCGATAGAGGATCCCAACCTTAGGGAAAATGGAATTTCTACAATCACTGCAAATAAAACAGATATCATTTGATAATAGAAATTATTGGTATGCCCCAGATTTTTGTTCAAATCCTTAGTGGGAATAATCAAACGATTCTGTTCGTACATTCGCAAAATTAAGCGTTTCACAATTAGTGAACTATATTTTTTGTCATAATCCGCATTTTCCAAGAAAATAGGGCGGTTTCTATTTAATCTATTTAAACCGTGATCATAAGCAAGTACATAAATATAGTCCCGAAAAAAAAGTGGATATAGAAAACTCTGTTGACGAGCCCCATCGAACTCTAAATATCCTTGAAATTTCTCCATTTGGATTAAAATTCGATTTGAACTAAAAGTAAAGTCTTTATTTTCTTGAGTTCTGAAATGACACATAGTGCGATACAGTCAAAATAAGGTATTAGATTACGAAAGCACTAAATACCTCATAAACAGGTAGACTGCTAACTGGATTCTCTATCTTTAATAGGTTTCTGTTCGTTATATTATAAAATAACAAAACAAGATGATTAGAAATCCTTTATTTTTTTAACCTAATCGCTCTTTTGATTTTGGAAATATATATATATATATTTTTTTTTATCAATATACTGCTTCTTTTACACATCCATCTACAACCTAACCCAAACGGACTAGGGAAAAAATAATTAGGACTCACGAAAAAATTGATAATAACACGCAAGAAAAAAATTCCTTCCCATACCCGTATTAGGTACTAATCTATTTTTAACATTTAATTAGATCGGGTAATTTTTCAAATTACGAATGGAAGCTCGTTTCTTTTTTTTTCTTAGAATAAGGAAAACTTGTTTTTTATCCATCCATTTATATTTATTCACTCGACCCAAATTGGAATTCTTCTTTTTTTTTTTTTTTTCGACAAGGTTGTACCGATTAGAAAATAAAAAAAATTTTTATCTGAATTCTCCCTTGATACGACATGCTATTTTTTCCGTTCATTCCTTTCAGGATCAGTCGTGGTCTTACAAACTCTACCGCGGATCTGGACGAATCCTTTTCTTCATACAAATGTGTAAAAGATGCTAGTCGCACTTAAAAGCCGAGTACTCTACCGTTGAGTTAGCAACCCCCCCCACAAAAAAAAGCAAGTACTGCAAATATGTAGATACAACCAGAATAAAGAAAAAAAGAAAAATCCAGTCATGTGTGCGTCAGGGAGAAATAGATCTATTTCTCTATGAGAGAATTATATTTGGTCCATACACTGTTGTCAATATGATTGTAAATTTTTAATATAGCGAAAAGAATAGAAAAAATAAAAAAGTTTAACCCCCTGGTTTGTGAGTTCATACAAGGAATGAAAACTAAGCCGAATAGGATAATCTCAAATCTTTCTATTTCTATATATATTATGATTCAGAATTAATATATTATTTATTATATTAATATATTATTTATTATACAGTATGATTTTATATATTTTCTATACAATAATTCTATACAATAAAATTTTGTATTTATACAAAATTTAGAATTTCTATAAACCCAAAAATTTTTTAATAAATTTGTTTTTTATTATAAAACATGGTAGTTTTTAGCAGGATATTTGTTAGTTTTCATACCTTTAGGAAGAATACTAATAATAAATGGAAATTCTAATAAATCAAAATAAATATGATGGAAACGAAAGAGGAGGAAAGAAAAGAGTAGATCAAATTTGATACCAAGCTATATATGAGTCTTTAACATCCTCTTTTTTATAGTTCATTAATTCAATTTCGTTTTATTAAGACTTAATTCCGTAAAAATCCCTGCCTTCTTTGAAATATCATGAACTGTTCTTGTTGGTTGAGCGCCCTTTTTAAGGAAATCGAGAATAGCAGGAAGATTTAAATAAGTTTGATTAGTTATCGGATCATAAAACCCCACCTTCCGAAGATCTCTTCCTTCTCTTCGGGATCGAACATCAATTGCAACGATTCGATAAATGGCTCATTGGGATAGATGTATATGAATAATACCCCCCCCCGAGAAACGTATACGAGGCTTTGGCCTCATACGGCTCGAGAAAAAAATGCAATGAGTATAAGTTAACTCTCTTTATAAAATTCAAACATTAAATTAATTAGCCAGTATTGAGTATTGAAACCCTAACTATTTTTTTCATAAAAAGCGTTCGTAACATTCGTACTCTCGTAACTCAAGTTAAATAACTCTCAAATATCTCAACAGAGACTCCTTAAGTACTCTTTTTATTGAGTAGTCTCTAACCTTTTTTTGTTTGTCTCATTTTTTCGAATCAATTTTGATTCTTCATTCTGATCTAGTTGTTCAAACAATTGAAAAAGGGATTTCCTTGTTTCAGGATTCTTTATCCTTACTTTGAATCTTTGGGTTTAGACATTACTTCGGTGATCTTGATCGTTTTATTAAAAAAGGGCAGCAACAAGCCCCTTATTTTGTTTATGATTTCTTTTCTTTCTATCAAAGAATCATACAAACGCTTGATTCACGCATGATAGACTTTTAATTCAAAGAATTTTAAAATTTTACGAAAATTTCCTTTTCCATTGTAAAATTACTTGAAAGGGCTTTTTTTTCAATATAAAAATAAAAAGACTTACGAAGTTGTTCCAACTTATTGATTCGCACTAACCCTAGATCCTTACTCCGGCGAAAGGAATAAAAACTTTCTATTCTCCTCGAGCTCCATCCTGTACTCTTTTTTATATTCAAAAAGGTGTAGGACTCTCGTAAAATAGAACACAAAATGTCGAGCCAAGAGCACCTATATTCCTAATATAAAAGGTGGCGGATCAAAACATCCACAGCAGATCATGTCCTTCAATTCAAGTCGCACGTTGCTTTCTACCACATCGTTTTAAACGAAGTTTTACCATAACATTCCTTTAGTTTGTGTAATTGATTCAATTATGGAATCATGAATAGTCATAGTTCAGTCAGTATATCGTAATCTATACTTTTTCTTTCTCTATGAATGGAATAGTGAATCTATGCGTAAAAGGTTCAGTCAGAATTCAAATGAATCCCACATTAAATTCTATATATGTAAAAATATGTAAAATCGAAATTTGAATAGAAATCTATATTTCTATATATAAATATATTTTTTTTTATTAAAACTCGTAGAATCTACGGTTCTACCTTACTTACCTACATCACACACAACTAAAAAAAGCAAATAGATTTTTTGTAATTTCGGGTGAAATAATGAAAAAGAAGTTTATTCTTCTTTTCATTTCAATATTTTATTCTTAAAAAATATTGTTTTTTTAAACAGAAAGAAAAAGATGGTGTACAAAGGCAATAGAAGATTTATTTCGTAATGACTGGACTCTGGGACGGAAGGATTCGAACCTCCGAATAGCGGGACCAAAACCCGTTGCCTTACCGCTTGGCTACGCCCCATTTTTATTTTTATTCAAGACTACTAAAAGAGTAATATTGCTATTGGTTGTTCGTCAATTCAATTTAAGCCCAAATGAAATATAGATTACATTGGTGCTATAGTTTTGACACGTGTAGATAGCAAATCAAACTTACTTTATTGATCATTACATAGAATTCAATTAAGATATTGTATGAAAATATTATTTCTTTCATTCTCTTATGAGAATGAAAGGATTTTTGATTGAGTAAGTTCAACAAAGTCTTTTTAGACTATCTTTCTTTATTTATTTATTTTTTTCCTTATATAAAAAATATATTAATAACTCAATCAAAATTAAATTATCCACAAGAACACCAATTTTTGTTATGCTTAATATATTTAATTTGATCTGTATTTGTTTTAATTCGGCCCTTTTTTCAAGCACTTTTTTAGTCGCCAAATTGCCGGAGGCCTACGCCTTTTTGAATCCAATCGTAGATGTTATGCCCGTAATACCTCTTTTCTTTCTTCTCTTAGCCTTTGTTTGGCAAGCAGCTGTAAGTTTTCGATGAAATTATTAATACTGTCTTAGAAAAATTCACGATTTTGATTCTTCCAACAATTCAAATCAAAAGATCAAAAAATCTTGACGTAGGAAGGAACTCTCAATTCAAACATTGAATTTTTTTGGTAGCCATACTAAAATCTGGATCATTTGATTTCCTCAGTTTTATCCTCTTTTCTCTAAATGAAAGAACTTAATTAGATTCGAGTTCACTCACAAAAAAAGTATCTAGATATTTAGTATAAAAATAGAGAATCTATTCTCTTTTTTTTTTTTTGAAAAAAAAAAGTAAGATCTTGGAGATTGTGTAATGCTTACTCTCAAACTTTTTGTATACACTGTAGTTATATTCTTTGTTTCTCTCTTCATATTTGGATTCCTATCTAATGATCCAGGACGTAATCCGGGACGTGAAGAATAAAAAAGAAAGGTTTTTTATTACTTTAATTTAATATTTAAATAGTGGAAATGTGCGAATTTTATTAGGATTTTATCTATTTCACATCATCAAAAAGGGGAAGGGAAAGAGAGGGATTCGAACCCTCGGTACGATTAACTCGTACAATGGATTAGCAATCCAACGCTTTAGTCCACTCAGCCATCTCTCCTAATCGAAAAGGAATACTTTTTAGGTTCCATTAGACAAAAAAACGGCTTAAAAAAAAACTTTCTCCACTTTATTCTTAAAAAACTTTTTTTTTTTTAGATTATTCTTTAATAATACTTTAATTATATATATATTTTCATTTTCTATATTTTATTATATATATTATTTTATTATATATAAAAAATATAATATTATTTTTTCTTTTTTATTATCTAAATAATATTTATATAATATTTATATATAATTAATATATATATTACTATTATATAACCTTTTTTATAGAACTTTCTCAGTAATTCTATTTACATAAAAACTGTAAATAAAGATTCAATAAAGAAAAGGCTCGAAAGAGAAATAAATAAAATCACAAAAATAGAAATAGAGAATCCTTTTTGATTTTGTCTCGTCCAAACACAAATAAAAGATCTTTTTTATTTTAATAGCCTGGCCTGGTCAGTCCCCAGCCGGGCCTTTTTTTGTTAAAGTTAAAAAGACCCATCCGATGGGTTTTTAGACAAAAAAGATCTGAAATAAAAAAAAGGAATCCTGCTTTGCCTAATTTTATTAAGTCTACGCTAGAATTTTCTCATTTTTTTTTCAGATTTTTTTTCTCCCGATTACTTTGTTCGACAAAAAGTAAATTTATATACAATAATTGGATTGTAGCGGGTATAGTTTAGTGGTAAAAGTGTGATTCGTTCCTTTAACCCCTTTAATAGTTAAAGGGTCTCTCGGTTTGATTAATCTTCCGATCAAAAACTTTATTTCTGAAAAGGATTTAGTCCTTTACCTTTCAATGAAAAATTCAAGGAAGATTATAGATTCTCGTAATTTGTATCCAAAGACTCTAATTAATTGTCAATTTGGATTATGAAATTTCGAAACATAATTTTTGAATTGGATGACTATTTACAATTCAATAAGTATAACAAGAGGATCCATGGATAAAGCCAGAAAAGTTTCTTTCTAATCGTAACTAAATCTTCAGTTCTATTTTTTGTTTGGTATAGAAAAAATTGAAGCAAAATAGCTATTAAACGAGAACTTTGGTTTACTAAAGACATCGACATATTATATTGTTTTAGCTCGGTGGAAACAAAATACTTTTCCTAAGGATTCCGTTAAATAGAAATAAAGAACGAAGTAACTAGAAAGATTTTTTGAGTTCGCCTTTTCTATCTTCTAGAAGGATCATCTATAAAGCAAAATTTTCTGTGAAAGCCTCCAAACGGGAAAAAAGCTAACATAGATGTTATGAGTCAAATTTTGATTTCGTTCCCATCTTATTTTATTTGGGAATTTCGCCATCCATCATAAAGGAGCCGAATGAAACCAAAGTTTCATGTTCGGTTTTGAATTAGAGACGTTAAAAATATAAAACTGATCGATCGACGTCGACTAAAACCCTTAGCCTTCCAAGCTAACGATGCGGGTTCGATTCCCGCTACCCGCTCTAAATTCTAAATTGTCCCCTTTTTATTAGAGACAATTTTCTCTATTAGAATTGTCTAATAGCAATTGTGTAGTGAATTCACTACACAATTGCTAAAAAGATTTCGCACATTTAACAAATGGGAAGTTAAAAAAAGCGAAAAGCGTCCATTGTCTAATGGATAGGACATAGGTCTTCTAAACCTTTGGTATAGGTTCAAATCCTATTGGACGCAATATCAATATAGATATAAATATATTGATATTTATCTATTATTTCCATTTCTATATATTATAAGAATATTTTTATTCTGTTTAGAAAATTTATAAAAAAGAAATATAAGTAAGAAATAAATTCTGAATGCTTTAAGATTTAATATTAAACATATACAGATAATTAGTTCTATACTTATATATATTATATATAGACTTAACTTAAATATACTTCTATTTTTTATAATTTCTCCTGAAGTAGAAAACGTTCCAGTTGCTCTTTAATACCTTCTTTCAAAAAGCTTTCTGCTTCAGCGGTTAATGTCTTGGTAGAGGCTATTATTTCTTGGAACTCAGGTTTATTTGTTTTTAAATAAGTGCGTAGCTGAACGAGAAATTTTCTTACTTGTCCAATTTCTAATCCATCCAGATAACCATTTGTTCCGGTATAAATGGTCATTATCTGTTCTTCCACTGTGAGAGGGGCTGATTGGGATTGTTTCAGTAACTCACGCAATCGTTGACCTCTTGCCAATTGATTCTGAGTAGCTTTATCGAGATCAGAAGAAAATTGGGAAAAGGCTTCTAATTCCGCGAATTGAGCCAATTCCAATTTTAATTTTCCAGCTACCTGTTTCATAGCTTTAATTTGAGCGGCAGATCCTACTCTCGAGACAGAAATCCCTACATTAATAGCAGGTCTAATTCCAGCATTAAAAAGATCAGCGGATAAGAATATTTGTCCATCTGTAATGGAAATTACATTAGTAGGAATATAAGCTGAAACATCTCCTGACTGGGTCTCGACGATTGGTAAGGCAGTCATACTTCCTTCACCTAATTCAGAGCTTAATTTAGCGGCTCTTTCTAAAAGACGTGAATGTAAATAAAAAACATCTCCTGGATAAGCTTCACGCCCGGGCGGTCTTCGTAATAGAAGAGACATTTGTCGATAAGCTTGTGCTTGTTTGGAAAGATCATCATAAATGATTAAAGTGTGTTGTTCACGGTACATAAAATATTCAGCCAAGGCGGCTCCTGTATAAGGCGCGAGGTATTGTAACGTAGCTGGGGAATCAGCCGTTTCAGCTACCACAATAGTGTAGTCCATTGCCCCTCGTTCCTGTAAACTAGTCACTACCTGAGCCACGGAAGAAGCTTTTTGACCAATAGCCACATAAACACATATTACATTTTGACCTTGTTGATTGAGAATTGTATCTGTGGCTACTGCTGTTTTACCGGTCTGTCTGTCACCAATAATTAATTCCCGCTGGCCGCGTCCTATAGGGATCATGGAATCAATAGCAATAAGTCCTGTTTGAAGAGGCTCATATACAGAACGTCTCGAAATAATACCTGGGGCAGGAGATTCAATTAACCGAGATTCAGAAGCTGAAATCTTACCTCGACCATCAATAGGGTTAGCCAAGGCGTTTATAACACGCCCCAAATAAGCCTCACTCACGGGTATCTGAGCAATTTTTCCCGTAGCTTTGACTGAACTTCCTTCTTGGATCATCAAACCGTCACCCATTAATACAACACCAACATTATTTGATTCTAAATTAAGGGCAATACCTATAGTACCCTCCTCAAATTCTACTAATTCACCTGCCATTACTTCATCAAGACCATAAATCCGAGCGATGCCGTCGCCCACTTGAAGTACGGTACCGGTATTTACAATCGTCACTTCTCTATTATATTGCTCAATACGTTCACGGATAATATTACTAATTTCATCGGCTTTAATGGTTACCATGAGTATTGTCCTAATTCTTTTTTAGAAGAAAAAAAAAAATAATGCCTATCATAATCGTAAGGAAAGGGCTAATCAGTAATTTCTTTCATCGTACCAAACATACCAATATTTGCATTAATAGTACGTAAATGTAACTCATTACTCAAACAACTATTTAGGGTTCCTATAGCTCCCTGTAAAGCTTGTTGGAAAACCCGTTCACGGACTTGATTAATTGTTCTTTGTTGCTCAAAAAGAATGGTTTCGTTTTTGTAATTTTCTAATTGTTTCAAAGTCCTAGAAGTTGAATTAATCAAATTGACTTTTTCTCGTTCGATTTCAGAGTATCCATTTACGCGAAACTGATCCGCCTCCATTTCTACTTTACGCAGGCGAGCTCGGGCGTTTTCTAATTGTTGAATAGCTCCTTCACGTAGTTCTTCTGAATTTCGAATAGTATTTAATATCCTCTGCTTTCGGTTATCTAATAAATCATTTAATGAAAGTAGATTATTCAGTAAAAAAAAAGTTCTATGATCCCTTCCCGAACCAAACATGAATCTTTCGATTCATTTGGCTCTCATGCTCACGTATTCCAATCATTTATCAATTATGTATGAGACTTTCATTCCCATATTTTTCATGTAATGAGCCTATCCTCTCCCAATTTTGTTGTATTCAATTCATATTCAATATATATTTCTATCGAAAAAGATCACCAATCCAAGACAAAACTATTTGGAGGATTCTTCTGACCAATAAAAAATTGATAATTGTCAGCAAAGTTGTTTCTTTTTTTCTTGAAATCCAAAGAATTTTTATTACTTTATACGTAGGTTATCAATTCTGCATTATACAAAAAGACTCAAAAATTTTTATCGACATGAGTGTTTTATATCGAAAAAAGCCGAACTATTCTTTTTGAAAATCTTATTCATTTTTTAATTAGACTACATATGGTAGAAAGAGTACCATGTTGCATCTGAACTTCAAACGGTTTAGTTTTAACCATGTTAATTAATGGTCCCAAATTTTTGGTTGATAGAGAATCAAAGTCAAGTAGACTTACCAAAGAATAACGAAATGCTATGGTTCTAAAATATGATTTTTTATTGAATTTTGTATTCAGAAGTAATTCGCGGGATTAGGCACTCTTTGCTAGTTATAGTGCCACTGGACGAATCCAGCCTATTCTTGAAATGAACAACTCACACACACTCCCTTTCCAAAAAAGATCAATACACCGAAGACTACACTTAGATTTATTGGATTTGTTGCTAAAATATCGGTATTAAACCCGAAACTCCCGGCGGATGGCCAGTGACCCAAGTAAACGAAAGAATCGGTTAAATTTTTCATATAATCTCCTCTTCTAGCTAAACTATAAAAAAAAAGAACTCTGTCCTTTTTTTTTTTATTCTTTGTTTTTTTGAATAAAAAGAAAATTTCGTTTAATAATTTATAATTTAATTTACCTATTTGGATATTTATAAACAGAATCAAAAACCTATTCTATTTACAAATTTATTTTCCAAAAATTTTTAATTTTCAATAATAATAATGAGACTTAATTAAAATTAAGCTAGAATTTGAGACCAAGTTTTATATCAATTTTAAAAAACCTAAACCTCCTTTTTGCGCAACACTCCTTAAAAAAAAATTTCCATTAAACTAAAAAGAATAAGGGGAAGGAAGAAAGCGAATCGATGTGTTAATTCCCCATCCTCAAATTAGTCCTTCCCAAGGGTTGTTGTCTCAATGAATAATTGTAGGAGTGAAATCTTGATAGAATAAAAAAAACTACGAAAAAAAAAATTCCTAATTTTATTATTTCTAGGATTAAACAAAAGGATTCGCAAATAAAAGCGCTAATGCTACAACTAGGCCATAAATTGTTAAAGCTTCCATAAAAGCCAAACTAAGCAATAAAGTACCTCGTATTTTTCCTTCTGCCTCAGGTTGTCTCGCGATACCTTCGACAGCTTGACCCGCAGCTGTACCTTGACCAACCCCAGGTCCAATAGAAGCAAGCCCAACAGCCAACCCAGCAGCAATAACCGAAGCAGCAGAAACCAGTGGATTCATGATAAGTTCCTCACACCAAAATAAAGAAATAGTTAATGATACAATCATCCAACGACTTAGGACTTAATTATAATTAAGTCATCGCTAAGATTCATCCAGCCAAAATAACCAAAAACTTGATAAGAATTACTTTGATATTAGTTCCTATCCACGGGATTTTGAAAAATGCATAATATATATATATACGACTTTTTTATGCCGTTTCTTTTTTGTGAACCATTCTTTTCTTTTAATTCTTCGTTCTTTTTTTGATCGTTTTTTTCAGCCAATTAACAGATAAAAAGTAAGAACTTATAATCGAATCGTTATCTAAATAGAAATTCACAAAAATAGTGGGGCAGATTATATAGATCTTTAACTTATATATACCTAGTCAATATCAAATATGACATATACAAGTGTTTCTTACATAACGTAAACCAACTATTCGATAATTGGGCTAACCTAAATTTGAAAAAAAAAATAGTTAATGATGACCCTCCATAGATTCACCTATATAAGCCGCAGCTAAAGTGGCAAAAATGAGAGCTTGAATCCCGCTTGTAAATAATCCAAGGAACATGACAGGTATAGGAACCACTAAAGGTACTAAAGAAACAAGAACAACAACTACTAATTCATCGGCTAATATATTTCCGAAAAGTCGAAAACTCAGTGATAGGGGTTTTGTAAAATCTTCTAAGATGTTAATGGGTAAAAGAATTGGAGTTGGTTGAATGTATTTACTGAAATACCCTAATCCTTTTTTGCTAAGACCCGCATAAAAATATGCTACTGATGTGAGTAAAGCTAAAGCAACCGTCGTATTTATATCATTCGTTGGTGCTGCTAACTCCCCTTGAGGTAACTGGATAATTTTCCACGGTAAAAGGGCTCCTGACCAGTTAGAAACAAAAATAAATAAAAACAGGGTTCCAATAAAGGGAACCCATGGACCGTATTCTTCTCCAATCTGGGTTTGACTCACATCTCGAATGAATTCAAGGACAAATTCAAAGAAGTTTTGGCCGCCAGTTGGAATGGTTTGTGGATTGCGAACCGCTAGAGCTGCGGAACCTAATAAGATAGCAATTACAACCCAAGAAGTAATAAGGACTTGCGCATGGACTTGGAACCCCCCTATTTGCCAATAGAAATGTTGGCCTACTTCTACACCAGATATCTCATATAACCCTTCTTTTATTAGTGTATTGATGGAACATGATAAAACATTCATATTGCCCTCTGACAGAAATAAGAACTTTAAATTATTTTGATTCAAGACCCCCCCTTTTTTTTTACTTATTTACTTGAATTTTCTATTTTAGTTTTGGATACCAACTAAACGAATCACACAATATACCCAGTTTTTTATCTCTTTTTCTTTTGTATGATTCAGGAATAGTAACCGATTTTATAAATCGAAATACAGGGAGCCCCTCCCTCAAAAAAAATTGATTTATTTATCTTATTATTAATCAAGAATTTTGTATATAGCTAGAACGACCCTCACAAATTGCGAATACTAATTTGTTAAGAATGAATCGAATTGAAGCTATAGCGTCATCATTTGCTGGAATAGAAATATCCGCGAGATCGGGATTACAATTTGTATCGATTAAAGAAATGGTTGGAATTCCCAAAGTTATACATTCTCGAAGAGCCGTATATTCTTCTTGCTGATCGATGATGATTACAATATCAGGCAATCCCGTCATATATTTAATCCCGCCTAGATATGTTTCCAAGCGAGATAATTGTCTCTTCAACACAGCTGCATCCCTTTTCGGAAGACGGTTGAATCCCTCTGTCTTTTGTTCAGTTCTCAAGTCCCTAAACTTATGAAGTCTTTTTTCTGTAGTAGACCAATTTGTTAACATGCCGCCGAGCCACTTTTTATTAACATAATGACACCGAGCCCTTATTGCAGCCCGCGACACTAAATCAGCTGCTTTATTTTTTGTCCCAACAATTAAGAATTGTTTTCCCCTACTTGCTGCATCAAAAACTAAATCACAAGCTTCTGATAAAAAACGAGCAGTTCTAGTCAGATTTATAATATGAATACCTTTACGCTTTGCAGAAATATAAGGTGCCATTCTAGGATTCCATTTCCTAGTACCATGCCCAAAATGAACTCCTGCTCTCATCATCTCTTCCAAATCGATGTTCCAATATCTTTTTGTCATTTCTTTTCACACTTAAAAGGGGGGTACCCAAAACTAAAATAAAAATTTGTTCCAATGGAACCTTCTCTTGTCCGTTTATGCACGAGCCGAGCCATTATTTTGTATTCATTATTATCTTTATTAGTGTTAACAAATTATTAAAGCAAATGACTACAGCAAACAATAAAACATGAAATTCAAAACAGGAATCTGCTATTAGGAATTATTCAATTCTAGAAAAGGCAGATTTGTAAATAGAAGAGTCACAAAATTCCCTGTGATAAAATAAAATATCTCTCATATCTCCCTCTAATAAAGATAAATTCTTTGTTTTTTTTTCAAAAAGAATATTGGTATGTTGCCGTGAACAATGCACCAATCCTTTGTTGAACCCGGTCCCGGCGGGGATCACACCCCCTAGAACAACATTTTCTTTCAGGCCTTTCAACCAATCGATACGACCCCGAAGAGCAGCTTTTGCTAAAACTCTAGCAGTTTCTTGAAAACTTGCTTCGGATATAAAACTTTGAGTATTCAAAGATGCTCGAGTTATTCCTAATAAAACGGCTCGATAACAGATTGCTTCTTCTAAAGCACGCCCCGTGCGTTCTGCTCGTAACAATCCAATCAATTCTCCAGGTAAAAAAACATTAGACATTCCCTCTTCTGAAACCAAAACTTTTGATGTTATTTGACGTACAATAATTTCGATATGCCTATTATGAATCTGCACCCCCTGGGATCGATAAACCTTTTGAATCTTATTAACCAAAGAAATACGACTTTGCACTATAGTTAGCTCAGCACCAATCAAGAATCCCCAAGGAATTCCAAGAATTCTTGTTATACACCTGTTCCAACCCTTAATCCGCTTTTCTAAGTTCAGTGATATTGAATCAATCGAGCGGACTTCTAACACCTGTTCTACTTTTGGAAGACCTTGGGTTATATCACCGGATCTCGATTTTTCATATATAAATGTAACTAATGTATCCCCTTCGTAAAGAATTTCTCTATAATGCCCGTGAACTTTTGCTCCCGGAGTAGCCAAATAGGGCTTAGCGGATCTTATTACTACAGAATCCCTTTGAACAATTAAAACTTGACCCGATTTTAGGTATGGTTCTTTTTTGGCTATACATAGATTTTCACAAAAAAATTGTCCAAGACTTATTATTGTGGACGTTTCCTCACAATAATAATTATTATAATTTTGATGAAGAAAATACCAATTCAATTTGAATGGATTCAAAACAAGGTTACTGTATGGATCTAGATTAAAAATTCTTCCGTTTTCATCTATTAAATAAGAGTGAATTATTTGAAAAATATATTTGAAGTTATCAAGTTGCAAATATTTAATTACAGAGATCTGATTATAAGTTAGTAAAGGCAAAAATGAATAAAAATTCGAAATTTGAATGGCTGTTCCTAAGGGGCCCGACGAATTTTGAATTGTAATTAGAGGTTTTTTTTTTATTGATTGGTTTATAACATTGTGATATTTTACATGATTAAATGGACCGATTCTAAAACAATTAGAGGATGATAAAATTAACAAAGATTGGGATTCCTTATTTCTGAACATACGAATAGTTCCGTGATTTTGTCTAAGCGATTGTTGAAGAATGCCAGCCTTGGGAGAAAGCGAATAAAACGGATTCACGGAATCTGCAGAGATCAATCCCGAATCCGGCGGATTATTCCTTTTTCTTATATACGAAATATGGGATTTCACTAAGCCAATTCTTATGAAATCTCGAATCAAACCCTTTGTACTTACTTCAACAACGAAAGCGCGGACCTCCTCGAGGGAAGAATTTTTGTTGTCTTGGTCCCAATTCAAGACTAAACAAGTGCGAACCAATTGAATACTTGTGTCAGAAATTCCTCGAGTTGGTTTACCATTTCCATAAAGGATATAGTTGAAAACTCGAAGTTGAATATTATCCTTTTCCCGAAAGAGATCTTGTGGGAAGAGTGTTGCTAAATTTATACTGTCCATTATCTCATAGGTGGCTACGGGCCGCACCAAAACAAAAAACTTTTTCTTGGTTGGTGTGATCCGTTGGGCATAAATCCAATTTTTTAAATTTTTTGATTCTTTAGAGTTTGTTTTTCCCCTTCCTGGCGGTATCAAGATGCCACTATGTCGGGATATCTTATCTGTCTTGTCCGGAAAATGGATATCCCCCGAAAATATTTTGAGTTCAATCCTTTTTTTTTTTCTCTCCACTCGGATCAACCCGCCGACTTGGCTTCTTATATTTAAAGTGATTCGTGTATCGACTCCAATGATACTATAGTTCTGTACCATTATGGCGGAGGATTCGGGTAAAATATGCACTTCCTCAGGAATGAAAAAAAAGCGATCTACTTTCATTTCGTATTTTGTCTTAAATTTTTGGACTCCTCGATACTCAATCATATCCTCTTTTTGGACGATTGAGTCCGCCTTTAGAGTTCCATATTTAAGAATTCCGGAACTCTTTCTTCTGTATCTAGGATCATCAAAAAAAGCAAAAATACTGTTTCTACGGAAAATACCATTTATGGGTATTTCAATCGAGATACCTGAATGTGGTATGAACTCTTTCGCTTGCTCTTGAATCGATTGGAATGGAATGAGAAATCTATTTCTTCGCCTTTTTGCTAATAAATCCGAATTCTCATGAAAAATAGCAGAATATATGAAATTATAATGACTAGTACCTACGATTCCATTCAATTCTGAATAATTGGGAATCCCAGATTTTTTTTTATCAGAAAAATCTGAACTGAAAAATTTTTTGCTCACTTGATCATTATTCACTGAGAGGCTAGAAATAGATTTTCTTTCGACGGAAAGAAAGGGTATGTTCATTTGATCTTGATCTTTGTGGATCGAAAAAAGAATTAGACTAGATCCACAAGAACCTCCTGATAATATCCATAAATGACTTGTTTTTGGTAAAAGATGGACATTACTATATGTAAATTCGGGTGCATGGGATACATCAGTACTCCAATGCATTTCGCCCTCGGAGTCAGAATAAATATATTTTCTAACCCTCTCTTTAAAATGAAAAGTGGATGTTCCCTCGCGAATCTCAGCAATCACTTGTTCTGATTCCACATATTGATCATTTTGAACTAAAAGAAAACTTTTTGGTGGAATAGTCACGCTATGTATAATATCTTCGCTCTCAATAATTACAGACAAGTCTATATAACATAGAAAGGCAGGATGCCCGTGACGTGTACGTGTAGGATGAACCAAATCCTCATTAAATTTGATTTTTCCATTATAAGGGGCTCGTACATGTTCGGCAGTACCTCCTGTAAATACTCCACCGGTATGAAAAGTTCTTAATGTTAGTTGAGTCCCCGGTTCGCCAATAGATTGACCCGCAATAATACCTACAGCTTCCCCCAATTCAACTAGGTCACCATGAGTGGGACTCCGGCCATAACATAATCGACAGATCCAAGATGTACTCCGACAAGTAAAGGGAGTTCGAATAGATATTGATTGTGTTCCAAAGGTTATGAATCGATTGACAAGTCCAATCCCAAGATCTTGATTTCGAAAGGCGACACATCGGGAACCTATATATATATCGTCTGCTAAGACACGACCAATTAATGTTTGGATAAAAATTCTTTCTGACATCATCCGACTTTTATTTCGAGGACTCACAGAAATCCCTCGGATAGTGCCACAATCCGTTCGACGTACAACAATATGTTGAACTACTTCAACAAGTCGACGCGTAAGATATCCAGCATCTGATGTGCGGACCGCAGTATCTACAACTCCTTTACGGGCTCCATAGCAAGAAATAATATATTCTGTTAAAGACAGTCCTTCGCGTAAATTGCTTTGAATAGGTAAATCAATCATTTGTCCTTGGGGATCCGACATTAATCCTCTCATACCTACTAATTGATGTACTTGAGATGCATTTCCTCTAGCTCCCGAAAAAGACATCATATGGACTGGATTGAAAGGGTCCGTCATCCTAAAATTAGGATTCATTTCCTGTCGCAAATATTCACTTGTAGCATACCATATCTCAATAGATTGGCGTAATTTTTCTACCGCATGTACATTCCCATAATGATGGTGTTTTTCCAAAATCAAACTTTGTTGTTCAGCATCTTGGACAAGCCAGCCCTTAGAAGGTATCGTTAAAAGATCATCAATTCCTAATGAAATGGATGTAGCAGTTGCTTGCTGGAAACCCAGAGTCTTTACTTGATCTAGGATGTGTGATGTATATGCCATCCCGAAGTGATCTATTAATCGGCTAATAAGTCGTTTAATAGCAGTTCCATCTATCACTTTATTGTGAAATACCAGATTGGCCCGTTCCGCCATAAGTACCTCCATATTCTGCTGAATGGGATTCGACAATGAGTTTGAGTCAATGATTGCAAAACTTCCTTTTCTCGATCTTGATTTTTTAGGTCAGGAACTATGTCCGAGTTGACTCGGAGAGGTCCGAATTCACACGGGTGTCCTATAATTCTTTTTTATGAATACCATATTATTAGGTATCATATGAACAAGCTTGAGAAAAACCTTGTATAGCTTCCTCGATTTCTCGATAAAAAGAAATATGACCAACTGTGGTTCGAATATATATAAAAAAAGTTTGTTTTTTTACACTTCTTACTATCAGATAGTGTGCATAAATCTCATGATAGTTACCAAAAGATTCATAGTGAACTTCGATAGGAACTTCTTTTGAAGCAATAACGCGTTGATCTAATTGCCACCGAAGCCACAAAGGACTATCTAAATTGATTCTTTTCTGCCGATAAGCTCCAATTGCATCATAGGAATTGCAAAAAAAGGGTTCTTTCATATACTTATAGTTTGTTTCGTAAATTCTTTCATTTTGATAGTTTTTTCGATTACATGGATTATATCTGTTTGCACAAATACCTCGACGAGTGCCGCTCGTTAATACATAGAGTCCAATCAGCATATCTTGAGTCGGTACAGAAATGGGATCTCCAATAGCTGGAGATAAGAGATTCATATGAGAAAACATAAGTAAACGAGCCTCTGCTTGAGCTTCTAAAGATAAAGGCACATGAACAGCCATTTGATCCCCATCAAAGTCTGCATTGAACCCCTTACAAACTAATGGATGTAAACAAATAGTGCGTCCTTCCACTAAAATGGGTTGGAATGACTGTATGCCTAATCTATGTAGAGTAGGTGCTCTATTCAGTAATACGGGATGCCCCTGCATAACTTCTTGAAGGATTTCCCAGACAATCGGCTTTTTTTCACGAATTTGACTCTTAGCAACTCCTATGTTCGAAGCCAGATGTTGTCTAATTAGACCACGAATTACAAATGTCTGGAAGAGCTCTATTGCTATTTCGCGAGGCAATCCACAGCGATGTAATGAAAGTGAGGGTCCAACGACAATCACCGAACGCCCCGAATAATCGACCCGTTTGCCAAGCAGAGTCTCGCGAAATCTTCCCTCTTTTCCTTCAATTACATCTGAAAATGACTTGTAAACCTTATTATGACCATCCCTCATGGGTTGTCCACGGATTCCATTATCAAGAAGTGTATCCACGGCTTCTTGTACCAATTTTTCCTGACACATTACTAATTCCCCTGGTGTAGATCTACTTGTTGTTAATAGATCAGTAAGAGTATTGTTCCGATAGATAACTCTTCTATAGAGTTCATTAATATCTGAACTCATCAGTTTACCCCCTTCTATCTGAATGATGGGTCTCAACTCGGGAGGCAGAACCGGTAAGAGACATAAAACCATCCATTCCGGTTCTATATTTGTTCGAATAAAATGCTTAGCTAATTCCATACGTCTAACTAAAAAATCTTTTCTTCTTACAATTTTTCGATCTTCCCATTCATTCCCCGTGGGACCTTCTTCTCCTAATTGTTTCCATTCTACCAACGAATTTTCTATAATAATTCGCAAATCTAAATCGGCTAATTGTTCTCGGATAGCACCCGCCCCAGTAGAAATTTCTCGATTTCTAAATATATCGAAACCTTGAGTAGTAAAAAAAAGTGGGATGCTGTATTTCCAGGATTGAATTTCATATTCAAATGAACCTCGTAATCGTAAGAAAGTAGGTTTTTTCGTTATGGGCCTAGCAAAAGAAAAATTGGGATAGGGTCCACTATATGATCTCCCCCCCTCAAAACCGGACATGAAAGTTTCCTCTCATCCGGCTCAAGTAGTTATATCAAATAAAGATAAAGAAAGGGGTCGCACTTTCCAATTGTATTTTATAAAATCAAGTGAAAACCCAAAAAGAATCTACGCCTTACTCAAGTTCTCAGTGCAAACCAACCACCATTTCATTGATTCAATTAATTCTTCTTTGATTTCTATTTAGATTCTTTAGTGAATTCAAAATTACGACAGAAAAAAGAAAAAAATGTCAAATTCTTGAGTAGTCTACTTCCCTTCGAATGCCGGAATACTTTTTACCTTAAGTGAAAGGAATGCCTTAGAATTCATACGGGATTTATTTGTCTATGTATTGTTCCATTCGATCTTTTAGGTCCTGCGTTACCTCGATGGTTATGCCACAATATTCTTAAAGCTTATATGCGATGTATAGACTTCTCCAACCATGACATATTTGTTTACTTCAATATAAAAAACCAAATTTCTTTTCGTTTAGAAAGATAAGGGAATGCTTAATTCGACAAAAAAAAGGTCTTCTTTTCACGAGGTACGACTATCAATTTGAAGTTACTTTTTTTTTACTGAATCGACCATAGACCAATCGCCCTTGTTATTTGGGAGTATTGAATACACCCACAAGTCTGAGCTTCATGTTACTCTTTTCAAGAGACATGTCAGATCGAGGGCATCCCAAATTGATTGAAGGGGATGAGAGTTTATCATTCTTAAAAATAAAAATTTCGATCAAATCACACATCGCAGTATACTAGACCTTCTAATTCTTTAAGAGGTTTATCTAAAAGATTCGCAATATAACTAGGAAGACGTTTCAAATACCATACATGAGTTACAGGACATGTCAGTTTTATGTATCCCATTTGATATCTTCGTATCCGAGAATCAACAAATTCAACTCCACATTGTTCACAAAATTGCGAGTCTTCTTTTTCATCTCCGATCACTCGATAATTTCCACAAGCGCAAATTCCACTCTTTATAGGCCCAAAAATCCTTTCACAAAATAATCCATCTTTTTCCGGTTTATTGGTTTTGTAATGAAAAGTATAGGGTTTTGTCACCTCTCCAACTATCTCTCCATTAGGTATTTTTTTAGTGGCCCAAGCACTTATTTGCTGAGGAGAAACTAATCCAATTCGGAGTTGTTGATGTTTATACCGATCGATCATATAAGAAATTTTGTGATTCATTCCGATTAAACTTCCTTCCTATTAATCTGGAAATTCTTCTCAGATACAAGGAAATGATTCAGTTCCAGAGCCAAAGATCGTAGTTCTCGAACAAGTAATCGAAAAGATTCTGGAGCATCTTCTGGTTTAGGTATTGCTCCTCCAATGATAGTGGTACCAAGTACTTCTTGGCGAGCTCTAATATGATCAGATTTATAAGTAAGCATCTCTTGTAAAATATGAGCAACACCAAACCCCTCTAGAGCCCAAACCTCCATTTCGCCTACCCGCTGCCCCCCCTGCTTAGAACGGCCTCTAAGGGGTTGTTGTGTAACAAGTGCATAATGTCCACTAGAACGTCCGTGTATTTTATCATCAACCTGATGAATTAATTTCAAGATATAGGGCTTTCCTATTATCACAGGCTGTTCAAAAGGATCTCCCGTTCTTCCATCAAAAATGCGGCTTTTTCCTGGATACTCGGGTTCAAATACCCATGGATTGGCTGTTTGCTTACTAGCTTCATATAATTCAGAAAATACGAGTTTTCTCGAAGCCTCTTGTTCATATCTCTCATCAAAAGGGGCTATTCGATAATGTCTATCTAGCAAACTTCCCGCTAATCCAAGCGAGCATTCAAATATCTGTCCTACATTCATGCGTGAGGGTACTCCTAATGGGTTGAAGACCATATCCACGGGTCTCCCGTCTTGCAAATAAGGCATATCCTGTCTAGGCAAAATTTTGGAAATGATACCTTTATTTCCATGTCTTCCGGCTACTTTATCACCTACTTTGATTTCACGTTTCTGTGAAATATATACACGAATTATTTCGGGGTTATAACTTGAACCCCCCTTTTTCTGAACCCATCTCACATCAATAACTCGACCTCTACCACCTATAGGCAATTTTAAACAAGTTTCTTTTGAAGTCGATACCTGAATGCCAAGTATGGCCCGTAATAATCTATCTTCCGGAGCATACGAGGATTCTTTCGCCATCTGAGGCGTTAATTTACCTACTAAAATATCACCCGTTTCAACCCACGATCCTAGCATCACAATTCCATTTTTGTCTAAATTTCGGAGTAAACGGCCCTCTAGATGCGGTATTTCCTTAGTGATCCTTTCAGGACCTTGGGTTGTCACATGCGTCTGAATTTCATATTTCCGTATGTGGAAAGAAGTATAAATATCACCATATACTAGACACTCACTAATGAGTACCGCATCTTCAAAATTGTATCCTTCCCATGGCATATAAGCCACTAATATATTTTTCCCCAAGGCGAGTTCCCCACCAACTGTAGCAGCACCATCCGCTAAAATCTGTCCCTTTTTAATACATTTACCCCGGCGAACCTGAGGTTTTTGATGCATACAAGTATTTTTGTTTGAGCGTTGATACATAATTAATGGAATACTTAAAGTATTCTCATTTCCCGATAAAATTATCTTCTCAGTGTCAGTATAAAGGATTTTTCCCTCGTGTTCGGCTATAGCGGGAACCCCCGAATCTAAAGCCACTTGGCGTTCCAATCCAGTTCCAACAATGCACTTTTCGGACCGAGAAAGTGGAACTGCTTGACGTTGCATATTAGAACTCATTAAAGCTCGATTCGCATCATTATGTTCGATAAAAGGAATTAGGGAAGCTCCAATGGAAAAATATTGGAAAGGAAAAATGCTTCGAAGATGAACCTCTTCCCATGCGATAGTCAAAAATTCTTGGCGGTATCGAGCTGGTACAGCCTGTTCTTCTTGAATGCCCCGATTAAGAGCCAAAGAATTTCCTGCCGCTATCATATAATATTCATCTTGACTTGGTGATAAAAAAAGCATCCGTATCCGCGCCTTTTTTGATTTCTCAACGAGTTCATAAAACGGACTTTCTAACGACCCCCAATCACCAATCCTGGCATGAATTGATAAAGATCCAATAAGTCCCACATTGATTCCTTCAGACGTGTCAATGGGGCAAATACGCCCATAGTGACTAGGATGGATATCTCGTATTCGAAAATTAGCAGTTCGCCCTGTTAATCCGCCAGGGCCCAAATAACTCAACTTTCTCCCATGAACGATTTGTGTCAATGGATTAGTGCGATCCAAAACTTGAGATAATGGATGTAATCCGAAAAAGGATTCATAAGTAGTTGTTAACGGAGTTGAAGTTACCAAATTCTGAGGAGTCGGTATCAATTTATGCCTAATTGCTCCGCCTATAGTTCCCTTAACTACATTTTCTAAACGAGCCAGAGCCAACCCGAGCTGGTCTTGTAAAAGATCCGCTACAGAGCGAATACGTTTATTTTTCAAATGATTCATATCATCAAGTGTACCCATTCCAAATTTCATCCCAATCAAATGATCGGCAGCTGCTAATATATCTCGTGGTAACAAAAATATATTGTTCTGAGGTATATTAAGATTCAGTCTCCAGTTAATATTTCGGCGACCAATCCTCCCCAATTCACACCTTTGGTGAAAGAATTTTTTTTGTAATTCCTTACATAAGGATTCAGAAAATATTGGATCCCCACCTACACAAGAAAATTGTTGATAAAACTCCAAAATAGCATTTTCTTTTGACCCAATTTTTTTTTTCTCCTTATCGGTTAAGAAAGATAAGAAAATTTCAGGGTAGCAAACATTCTCTAGAATTTCTCTTAGATTCGAACCCATAGCTGATGATAGAACTAGAATAGATATTTTCTGTTTCCTACTCACACGAGCCCATATTCTTGCTTTTTTATCAATCTCTAATTCTAGCCTGCCCCCCCAATCTGATATTATGGTGCCGGTATAGACCGAAATCCCGTTATGATCCAATTCTGACTGGTAATAGATACCAGGACTTTGTAATATTTGATTGATCACAACTCGGTATATTCCGTTTACTATAGAAGTTCCAAGGGAATTCATTAAAGGAATGTTTCCAATAAAAATTCTTTGTTCTTGCATATTCCTATTGGTTTTCCAAATTAATCCCGCGGATACATATAATTCAGAAGAATATGTAAGTAATTCATAGACAGCATCTCGTTCTTTTATCAGAGGTTCTACCAATTGATATGTTTCCACAAATAATTGAAATTCAATTTCGTGATCTATATCTTCAATTTTTGGAAATTGCGAAAGTTCTTCTATTAAACCCTGATCAATAAACCGATAAAACCCTTCAAATTGTATCTGATTAAATCCGGGTATTGTAGATGTTCCCTCTTTTCCATCCCCGAGCATCTTTTTTGAATTTATCATTTATCCGTTTATTTTAAAAATCCCATATCTCATTCTTCACCGAATCATATAGATAGAATTCGATCTAGCAATAATGGAATTTCTATTCTGTTTACTGAATCACATGAAATTTTATCCAACTCCAAGATATATGGAATGTATGAAATCCGTATGAACGGAGACTAGATTCAATTGGAATTTTTTTTATAAGAAAGAGATCCAAATGGAACAGAATTTAGAAATACCGCTGGAACTTATGGAGTTTTGTAACGACTAGAAAAAAAGTAATTTCATTTTCACCTATGATATTACATATTCCAATTCGATCGCATACCATAAAAAACGGTATTCATGATAGGATCTGTTCGAGCAGATAAACATATAAGAAATAGAAAACTTTTTTTTAAACACTTTACTTTTTCATGTATTTGTATTTCATTGTTCAAAAAAATAGTTGCAGAAAAAAGATGGATTTTTACCTATTTTGAATAGAATATTTAGAATATCATTGAATTGAAGTAGGTAAGAAAACGTATGTTTTTTTATTTATTAATTTTTATTATTATTAAAATAAAAAAGAATGCACAGGTATATATATATGTCTCTTTTTCTTCTTTTATTGTGGTACAGTTCTATTTGGAACAGCACATGCTGTGCTCTACCAAAAATTAAAAATTTTTTTTCAATGTATTCAATGAAAAATTTCAATACAAAAATTTATTGAGAATTACTCCTCAAAAGCATCCCTAGAGAGATAAAATACCCCATTATAGAGCTATACAAGGTAACGTATGTTCTGATTCTGGGGTTTACATATACTCATTATTAGTGTTATAATTCAAATGGAAGAAGATTTCTTTTTAATTGAAAAAACTCAATATAGATTAGTTATAAATCTATTTCTAATGATTTTCTTATCTTATATTATTAGAAATAAAAAATGTAAATTTGAATTCAAAAAAGGTCATGAATTTACAGTCAATAGTTAATGGTTCTGATTTGTACTAGATTCTATATTTTGTGACTGAAAATCTATATTTTTTTCGGAGTTGAAAAAAAAAACAAGAGAAAATTTGAATCTAGTACAAATCATTTTGGCGGCATGGCCGAGTGGTAAGGCGGGGGACTGCAAATCCTTTTTCCCCAGTTCAAATCCGGGTGCCGCCTCAACAGGAGACTTGAAATCTCCTGTTATAAAACTATAACAAACGTAGGAAAAGACTCTTGATACTTTCTTTTCGTGATTCTAAGCCCCTGGCTCTCGAGGTTCTATTCTCTAACCTAAAGTTTTACCTATCAGATTAGAGGAAAACTAAAACGAGTGGAGGGAAATCCATTAGATTGGATAGGCAGAGAGGGAATTAAATTAATAGTTTTGGAAAGGATCTAAGATACTTTGGATATAGACTCATGAAAGTGGATATAGACTCATGAAAGTGGATATAGACTCATGAAAGTGTCGGAATGCTCAGACATTCAATCAATATTAGATTAGATGAAGAATTGCCTTTCGTTTTACTTCAAATAAAAATAAAAAACGATAAAAGAAAGAAAAAAGATTATTCTTTCTACATATGAGTCAGATTTTTTGGATACTTCGAAAAGTATCTGTTTACTTGTGTTTACATCTTGTCGATTCTACTAGAAATTCTATAATTAAGAATAACTCATTATAAGATAAGTGGATTTTTTGGAGTAGTTCATCAATGGTGACCAAATATCTCTCCCTTTTTTTGACTCTGCACCAGTGATTTCACTATTATTAGTGAACAATAATGGAAAAGTTTCTTCATATTCATAGGGGACAGAATTCACATGGATATAGTAAGTCTCGCATGGGCTGGTTTAATGGTAGTTTTTACATTTTCCCTCTCTCTCGTAGTGTGGGGAAGAAGTGGACTCTAGAAGTACTCCTAATTGCGATAATAATCAAACTCTATCAACCTGTATCAATTGTTTTAGTTTTCTAGACCGGCCGGCAATTTTTTTTAAGATCTTTTTTTAGAAATTGGATTTATGTTTTGTTTTATTGACTCATTTTTTTTTTTGATATCAGAGTTTATACCGTTAACCATTCATGGGATAACCCCCTTTCGAAATCTCAAGAGGTTTCCATCGAATTCGGATTATCCGTATTAAATGGATCAAACAAACAAATGAAATTGAGAAAGTATGTACATAGATTTCATATTCTATATTAATTTATATTTACATTTATAAAGAAAAAGAGAGATATGGGTGGATTCCTTTATATTAAAATATTTCACTTGTATCTTTATACATTACAATAACCATAATGGCTAGTATGGTAGAAAGAGATCTCTTTCTACCATACTAGCGGGCCCCTTAGGATACTACTGAATCTAATGCATTCCTTTCATTTAAGACGAGAAATTGACATCCTTTTTTGTCATTGATAGTCAAATTGTATTCAAATTAATTATTTTGACTAACCGTTTTTACGTAAATTATAAGCAAAAAAGCAGTAGGAACGAGAATGAAGAGTGCAGTAGCAATAAATGCAAGAATATTGACTTCCATAATTAAATCGTTTATTATTTATTTTTTTTTCTTTGGAATATCTCGGGATTTAATCCCATAGAGATGAGAAATCTTTCGCTTGTAAACTCACTCAGATGAATTAGATTTCGATGATATCGAATGAAAGAAATATCATGAATAACAATATCGGAGCTATAAAATCGATTCATCGTCAAGAATTTAATAGTATAACATAGGAAGATCTTTTATCCACACCAAATACATAATGAGATTCCTGATCCAATAAAAAACTATTTATTTATGATTCTTTTTCACCGCTTTCTTTTCTACAACCTAGTACTTTCCTTGTACAATCATCTGATGAAATATCATAAAAAACCTTTTCTACTTCGATTGTTTATAAAAAGAGTTTCTAAAGAACCTTAAATAAACAATAGAAATCAAATAGAGAAAACAAGTACGAAATTTCAAATTGAAATTTTCAAAATTTTGTTTTTGTAAGGGTCTATGATCTTTTTGTAAAACAAAGGAAATGTGATAAAGACGAGTCCCGATAAAAAAAAACTAAAATATTCCAAAAAATTAACTATATTAAATTTTCTTACGAGTTTTTCTTCGACATCGACTCTAATCTTTAAAAAGAGCATATTCATTAGGGAAGACTAATTTGATCTTTTTTTTTGAAACATCCTCTTTACTTGGTTGGATTCGAACTATTTTCACTTCCTTGACTTCATAGAAACAAAAGTATATATAGGTACTCTTGGCAAACGTATTATACGCTATCCTATTTGATTTTCCTACACGAGTTAATGGGAGATTAATTGACAAAAAGAGGAAACCCCATACAGTATCTCGTTCTTGAAGTGGTGAATGCTCTCAATAATTATAATTATACTAATTTACATATGTCTTTAAATTGGTGCTACAGAAATACCCCTTTCTTTTGCTTGATGAAAAAATAAAAATAAAACAAAAAGATAACCGAAACCATTTTGATCCCCTTGCCCAGAAACAAAAAGGGGAGTTTATGTCTTTTTTTTTTAATTGAATCCGCCGGGACTGACGGGGCTCGAACCCGCAGCTTCCGCCTTGACAGGGCGGTGCTCTGACCAATTGAACTACAATCCCATGGAAATAAAGCGGGTAGCTTACATATTCCTTCTTATGATTTCATTTGAATCATTTCAATTTTAGATTCAAATTAGTGTTTTGTAACAAAGAAAATCACAAGTAATATATTGATATCTATATGGATATCACTAAAGTGATATCAAGGCCGATTACTAGTAATCCTTGCATTATTCTAAAATCGATTGATAATCTATTTTTTATTGTCATTTTTTATGGAAACAAAAAGTAACGAGCCACAAGAAATAAAGAAAAAAGTAAAGTCGAAATATACCCAGATATTTGACTTTTTTCTTACCCTTCTCTGTCAATTATGCAAAACAAAAAAGGTTATGTAGACAGCGAATTATTGGGCCGAGCTGGATTTGAACCAGCGTAGACATATTGCCAACGAATTTACAGTCCGTCCCCATTAACCGCTCGGGCATCGACCCAGGAAGACTCTATTCGAACTTTATGGATAATCCATAATCAACTTCCTTTCGTAGTACCCTACCCCCAGGGGAAGTCGAATCCCCGCTGCCTCCTTGAAAGAGAGATGTCCTGAACCACTAGACGATGGGGGCATACTTGCTCAACCGCCATCATACTATGATCATAGTATGATCAGTTTTTTAAAATTGTCAATATAATCAAATGGTATGACTAGCTTATAAGATTTTTGATTTTTTTCTATAGCATTCTATATCATTTTTTTATTTTACATTTATATTCATATTCTAATCACAATTCTATAAAAAAAATCGATATATTTTCTTTTTATATTTGAAGTGGAAATATGAAAAAAAAAAAAAAATCGAAAAAAAGTCTAGTCTAAAATCTTTTAAAGAAGTGATTGGTCTGACAGAAAAAAAATAAAAAAGAGGGTTAAGTTTCGTTTTTTTTACTTTACTTCATAGATTGCCTCATCTCATTGTTAAGAAATAGTAGTGTCCCTATCTAACACTAACCCAAGAAAGTCAGACAGAATCCATCTTTCTTCCCTAATTAGACGATGGATTGATAAGTTAAGTTATCGATTCTCGCTTCTAGTTGCGAAATGAGCTACTAACCACTATGCGTCTATTGTATATATATTTAATATATATATATATTTGATTTACCTATCGACTCAGTCAGGAATTAAATCAAGACGGCCCTTTTAACTCAGTGGTAGAGTAACGCCATGGTAAGGCGTAAGTCATCGGTTCAAATCCGATAAGGGGCTTTTACTTTCTTTAACTTTCTATATAGGAAAAATTTCATTCGAAAGTCTATAATTTCGAATTTTTTGAATTTCATTCTAATGAATTTCATTTTAATAATAACTAATAATAAAGTGAGAGAGTAATTTAGAAAATCAAATTGAACATTTTTATATTATAATACAATGAATAATAATAAGTCGGCTTTTGAATCGCCAAATAGATATTCGTTGTTTCCCTTTTTCGATAGATTAGAAATCAACAAATCCAAAAGAAAAAGTAAGTGGACCTAACCCGTCGAATCATGACTATATCCACTATTCTGATATTCAAATTCGATAGAGATAAAATTGAAACAGTAGATTTGGTTTATTTCATATTTTTTTATTCGGAAATCTGTCGATATCTCTTATTTAATCTTCTTGTTTCTATATTTCATAGGAAATATATTGCGTTCCTGCCTAGAGAAAGAAAGTCTTATTCCAAATTTTTTAATACCTAAAGGGTATTTCAATATCTTGTTTTGATTCCAGAACATAACAAGAGCCTAAATTCTAGTTGTATAAGAATCAAATTGTATTAAGAATCAAAAAATCGAATCATAAAGAATGGCTTCAGATATCAATCAAATATTTCCATATTGATGCTTACAAGATGACAATGTAATGGGATTGAAGGTGTATGTGAGAAAGAAACTCTCATTTACAGTTTGCTATTATTTTATTTAAATATTGTATTGAATTAGATATAAATAATAAATTTTCCCTTTTTTTACCGGCATGGACATGTAGATATCAAATAAAATAGAAAAAAAGATTTCTTTATCTGAGTAATGAGTCATCTGACAATTCATGATTTAGATTCAACTACTTATTAAGAAACTAATAGCAAGGAAGAAACAATTTGAGTTGATGCGTTTACCTAAGTAAGGACCAATAAAATCAAATATTTTGATCTTCGAAACCAATTAAATGAAATTCTAAAGGTTAAATTTTATGGGGCAGTGCGCGAGAAATCAAATCATAAATAAATGATAGAATTTTGAGCGTCCTGAACATAATATATAACATTAAGATATATAAAGGTGTTCGGAAATGGTTGAAGTAGATGAATAGGAGGATCGCTATGACTATAGCCCTTGGTAAATTTACCAAAGACGAAAAAGATTTATTTGATATTATGGATGACTGGTTACGGAGGGACCGCTTCGTTTTTGTAGGTTGGTCTGGTCTATTGCTCTTTCCTTGTGCCTATTTCGCTTTGGGGGGTTGGTTCACAGGTACAACCTTTGTAACTTCATGGTATACTCATGGATTGGCTAGTTCCTATTTAGAAGGTTGCAATTTTTTAACCGCTGCAGTTTCTACTCCTGCTAATAGTTTAGCGCATTCTTTGTTGTTACTGTGGGGTCCTGAAGCACAAGGAGATTTTACTCGTTGGTGTCAATTAGGCGGTCTGTGGGCTTTTGTTGCTCTCCACGGTGCTTTCGCATTAATAGGTTTTATGTTACGTCAATTTGAACTTGCTCGATCTGTTCAATTGCGACCTTATAATGCAATCGCATTCTCTGGTCCAATTGCTGTTTTTGTTTCTGTCTTTCTAATTTATCCACTAGGTCAATCTGGTTGGTTCTTTGCGCCTAGTTTTGGTGTAGCGGCTATATTTCGATTCATCCTCTTTTTCCAAGGGTTTCATAATTGGACATTGAACCCATTTCATATGATGGGAGTCGCTGGTGTACTGGGCGCGGCTCTGTTATGCGCTATTCATGGTGCTACTGTAGAAAATACTTTATTTGAAGATGGTGATGGTGCAAATACATTCCGTGCTTTTAACCCAACTCAAGCCGAAGAAACTTATTCAATGGTCACCGCTAACCGCTTTTGGTCACAAATCTTTGGGGTTGCTTTTTCCAATAAACGTTGGTTACATTTCTTTATGTTATTTGTACCAGTAACTGGTTTATGGATGAGTGCTCTTGGAGTAGTCGGTCTAGCTTTGAACCTACGTGCCTATGACTTCGTTTCCCAGGAAATCCGTGCAGCGGAAGATCCGGAATTTGAGACTTTCTATACTAAAAATATTCTTTTAAACGAAGGTATTCGCGCTTGGATGGCGGCTCAAGATCAGCCTCATGAAAACCTTATATTCCCTGAGGAGGTTCTACCACGTGGAAACGCTCTTTAATGGAACTTTAGCTTTAGCTGGTCGTGACCAAGAAACCACTGGTTTCGCTTGGTGGGCCGGGAATGCCCGACTTATCAATTTATCTGGTAAACTATTGGGAGCTCATGTAGCCCATGCCGGATTAATCGTATTCTGGGCCGGAGCAATGAACTTATTTGAAGTGGCTCATTTTGTACCTGAAAAGCCCATGTATGAACAAGGATTGATTTTACTTCCCCACCTAGCCACTTTAGGCTGGGGGGTAGGTCCTGGGGGAGAAGTTATAGACACCTTTCCATACTTTGTATCTGGAGTACTTCACTTAATTTCTTCTGCAGTTTTGGGCTTTGGCGGTATTTATCATGCACTTCTGGGACCCGAAACTCTTGAAGAATCTTTTCCATTTTTCGGTTATGTATGGAAAGATAGAAATAAAATGACCACCATTTTGGGTATTCACTTAATTTTGTTAGGTGTAGGTGCTTTTCTTCTAGTATTCAAGGCTCTCTATTTTGGGGGCGTATATGATACCTGGGCTCCAGGAGGGGGGGATGTAAGAAAAATTACAAACTTGACTCTTAGCCCAAGTGTTATATTTGGTTATTTACTAAAATCTCCCTTTGGGGGAGAAGGATGGATTGTTAGTGTGGACGATTTGGAAGATATAATTGGAGGGCATGTATGGTTAGGTTCCATTTGTATATTTGGTGGAATCTGGCATATCTTAACCAAACCTTTTGCATGGGCTCGCCGCGCACTTGTATGGTCTGGGGAGGCTTACTTGTCTTATAGTTTAGCTGCTTTATCTGTTTGTGGTTTCATTGCTTGTTGTTTTGTCTGGTTTAATAATACTGCTTACCCTAGTGAGTTTTACGGACCTACAGGGCCAGAAGCTTCTCAAGCTCAAGCATTTACTTTTCTAGTTAGAGACCAACGTCTTGGAGCTAACGTGGGGTCTGCTCAAGGACCTACAGGTTTAGGTAAATACTTAATGCGTTCCCCGACTGGAGAAGTTATTTTTGGAGGAGAAACAATGCGTTTTTGGGATCTGCGTGCTCCCTGGTTAGAACCTTTAAGGGGTCCTAATGGTTTGGACTTAAGTAGGTTGAAAAAAGACATACAACCTTGGCAAGAACGACGTTCTGCAGAATATATGACTCATGCTCCTTTAGGTTCCTTAAATTCTGTAGGGGGCGTAGCTACTGAGATCAATGCAGTCAATTACGTCTCTCCGAGAAGTTGGTTATCTACCTCTCATTTTGTTCTAGGATTCTTCCTATTCGTGGGTCATTTATGGCACGCGGGAAGAGCTCGGGCAGCGGCAGCAGGATTTGAAAAAGGAATTGATCGTGATTTTGAACCTGTTCTTTCTATGACTCCTCTTAACTAAAGTAGTAGTTAAAATAGGAAAGTAAAAATCGGGTCATATTAAAAAGTCTTCTTTCTTTCTTTCAATTCAATCTCGTTTTTTCTGGCTCGGCTGGATAGTATAGCCGAGCCATTCTCCTTTTTTATGATGCTAAGAAGTAAAAAAAGCCAATAAAGAAAAAAATCTATTCATCCAACAAAAGGAGAGAGAGGGATTCGAACCCTCGATAGTTATTTTTTATGAACTATACCGGTTTTCAAGACCGGAGCCATCAACCACTCGGCCATCTCTCCAAAAGATAATTTCTATTTTATCTTTTTTTTTCGCCAAATAGAACATAGCTCGATGAGTTAATACGATCACTATGTAGAAAAAGATATAGGGTGTGACTTTCTTTATAAGTCTATCAATTGTATCTATATAAATGAGATACATGATCCAGTCTACCCATTTGTGAAGTAAAAAAGAACCTTTAACTTCATGTCCGAATAGAATAAAAGTGGTAAAAAGAAGTTGGAAATAAGGCATCTCGAATAAACGGATTCATGATAAAATCCCTTTATTTATTAAAATTTTTTAGTGGGTAAGAGGATTAAATGGTGTATATTGTTAATAGCTTGGAGGATTAAAAACATGACTATTGCTTTTCAATTGGCTGTTTTTGCATTAATTATTACTTCATCAATCTTACTGATTAGTGTACCCGTTGTATTTGCGTCTCCTGATGGGTGGTCGAGTAACAAAAATGTTGTATTTTCTGGTACATCTTTATGGATTGGATTAGTCTTCTTGGTGGGTATCCTTAATTCTCTTATCTCTTGAATTCATTCGTTGCAGATCAAAAAATGAGATGACCCCTCCCATTCCACGAATTACACATTCAAATTCAATATAAGTCCATAAAATGCAAATAAAGAAAACAAAAAAATTAGAGGGGGGGTCGAACTTCTGTAACTTGAGTGAAATATGAATCAAATATTAATAAATAGCAATTTACTAAATATAACTATGAAATAGTAATAACTAATTAAATAAAAAAAAAACGAATCAAAAATTGATATCTGATATCAATATAGAATATAATATTTTATGGAAATAGAGAATAATATATTATTGAATATGGAATTCTATATATAGATATAGAATAAATATATTATTAATATATAATAAATATATATATATTTATATATATTAATAGAATTGTTAATTGAACTTTTTTGGTAGTAGAGTTTTATCAAATGACCCCAAACCAAAGAGTGTATCTCGTATAGCTTTGAACAAATATTATCCATAAATTTCTTATCAAGAAGGCAAAAAAATGCGGATATAGTCGAATGGTAAAATTTCTCTTTGCCAAGGAGAAGACGCGGGTTCGATTCCCGCTATCCGCCCAAATAGAAATGGATTCAAAAAGATCAAAGATTCGGTATAGTTGACCGGGAAATATAGTAATTTTTGCCTCGCGTCCCAAAAGATAAGTATTAATTATAGTTAATAGAATCAAACTTACATTTGTTGAAAAAAAAATGTTGCGGAGACAGGATTTGAACCCGTGACCTCAAGGTTATGAGCCTTGCGAGCTACCAAACTGCTCTACCCCGCGATGAAACAAAAAAACTTGGACTAAACTCTAATAAACAAAGACGAATTGAATGCGCCCCTATTCCATATCTGTACAAATAGAATAGCCTATTTAGACAGAATGGTAAAGGGGCCTCGTCGAGCATAGAAAAAATAGAAAAATTAAAGGATACTTAAATCTTTACCAGCTTGATCTTGTTGCCCCTGGCAATAAACATGCCTGAACCATTTCCCGAAGGATGTGTCCAGATAGTCCAAAGTCTCGATAGTTAGCTCTCGGTCTTCCGGTCGAGAAGCAACGTCGATGAAGACGTGTAGGTGCACTATTACGCGGTGGGGATTGTAATTTTCCATGAATTTTCCACTTCTCACTTAGCGACGGAATCTCACTTATTTCCTTTTTTAAGGATCGACGAATCAAATGATATTTTTGTTCTAATTTTTGCCTCTTCTTCTCCCTATAAATCAAACTTTTCTTTGCCATAATGCTTAAGTTCCTCTTATTATCAATGATAATGATACAAATCGGATCCTAGATGTAGAAATAAATATAAGAGTGCATACCTATATTTTTATTATTTTAATAAAATTAATAAAAAAAAATATATTATTGCGGATAGAATAATTAAATAATTAACCGAATTTGCCCGACGTGGAGGCAATCAAGAAAGCCGCATAAGTGAATATATAACCTACAGAAAAGTGAGCTAATCCAACCAATCTTGCTTGCACAATTGAAAGAGCTACTGGTTTATCTTTCCATCGAATCAAATTTGCCAAAGGTGTACGTTCATGAGCCCATGCTAAAGTTTCAATCAATTCCTGCCAATAACCACGCCAGGAAATTAAGAACATAAATCCAGTAGCCCAAACAAGATGCCCAAATAAGAACATCCATGCCCAGACTGATAAACTATTCATACCAAACGGGTTATATCCATTGATAAGTTGTGAAGAGTTTAACCATAGATAATCTCTTAACCATCCCATCAAATAAGTGGAAGATTCATTAAACTGTGAAACGTTACCTTGCCATAATGTGATGTGTTTCCAATGCCAATAAAAAGTAACCCATCCAATAGTATTTAACATCCAAAAAACTGCCAAATAAAATGCGTCCCAAGCCGAAATATCACAAGTACCACCTCGTCCCGGACCATCGCAAGGAAAACTATACCCGAAATCCTTTTTATCTGGCATTAACTTGGAACCACGTGCATCTAAAGCACCTTTTACTAAGATCAATGTAGTTGTATGTAAACCTAAAGCAATAGCATGATGAACCAAGAAATCTCCAGGACCTATTGTTAAGAATAATGAATTACTATTCTCATTAATAGCATTTAACCAGCCGGGCAACCATATGCTTCGACCCGCATTAAATGCTGGGCCATTTGTCGAAGATAAAAGTACATCAAATCCATATGAAGTTTTCCCATGAGCGGATTGTATCCATTGGGCAAATATGGGTTCGATCAAGATTTGTTTTTCGGGAGTACCAAAAGCAAGCATGACGTCATTATGAACATAAAGTCCCAAAGTATGGAACCCTAGAAAGAGGCTGGCCCAACTTAAATGGGATATGATAGCTTCTTTATGGTCTAACATTCTTGCCAATACGTTATCCTCATTCTGTTCTGGATTGTAATCTCTAATAAAAAATATAGCTCCATGAGCAAAAGCTCCTGTCATGATGAACCCTGCAATGTATTGGTGATGGGTATATAACGCAGCTTGAGTCGTAAAATCTTGCGCTATGAACGCATAAGCAGGTAAAGAGTACATGTGTTGAGCTACCAAGGAAGTAATAACTCCTAAGGAGGCTAGAGCAAGGCCTAATTGAAAATGAATCGAATTATTGATTGTGTCATAAAGACCCTTATGCCCACGCCCCAACCGTCCTCCCGGAGGAATATGTGCTTCTAAAAGATCTTTTATACTGTGTCCGATTCCAAAGTTAGTTCTATACATATGACCCGCAATGAGGAAAAGAATTGCGATAGCTAGATGATGATGTGCCATATCGGTTAGCCATAAACTTTGCGTTTGTGGATGGAATCCCCCAAGAAGGGTTAGAATGGCAGTTCCTGATCCTTGGGAGGTACCAAATAAATGACTACTTGAATCGGGGTTTTGAGCATACAGATTCCACTGACCCGTAAAAAGTGGGCCTAACCCTTGGGGATGCGGTAATACACTTAAGAAATTATTCCATCGAACATATTCCCCCCTGGATGCAGGAATAGCGACATGTACTAAATGACCTGTCCAAGCCAAGGAGCTTACCCCGAATAGTCCTGACAAATGATGATTCAGACGAGATTCAGCATTTTTGAACCATGAAACTCTTGGTTTCCATTTTGGTTGTAGGTGTAACCAACCCCCTATTAAGGATAGGGCAGAAAGAAATAATAGAAAAAGAGCTCCAGTATAAAGATCTTCATTAGTACGTAAACCGATTGTATACCACCACTGATAAACACCAGAATAAGCTATATTCACCGGGCCAAGAGCACCTCCTCGAGTAAATGCTTCCACAGCCGGTTGACCAAAATGAGGATCCCAAATAGCATGAGCAATCGGTCTTACATGTAAAGGGTCTTGTATCCATGTCTCAAAATTTCCTTGCCAAGCTACATGAAACAAATTTCCGGAAGTCCACAGAAAAATTATTGCTAATTGCCCAAAATGAGAAGCAAAAATATTCTGATAAAGACGTTCTTCAGTAATATCATCATGACTCTCGAAGTCATGTGCGGTAGCAATACCAAACCAAATACGACGAGTAGTGGGGTCCTGAGCTAAGCCTTGGCTAAACCTTGGAAATCTTAATGCCATAATGCCTTTCAAATCCTCCTAGCCATTATCCTACTGCAATAATTCTTGCTAAGAAGAACGCCCATGTTGTGGCAATTCCACCCAGAAGGTAATGGGTTACTCCTACAGCACGTCCTTGTACAATGCTCAAGGCTCTAGGCTGAGTAGCAGGAGCAACTTTTAATTTATTATGAGCCCAAACAATGGATTCAATAAGTTCTTGCCAATAACCACGCCCGCTGAATAGAAACATTAAACTGAAAGCCCATACAAAATGAGCACCTAGGAAAAAAAGACCATATGCAGATAACGAAGAACCATAAGATTGAATTACCTGAGATGCTTGTGCCCATAAGAAATCGCGGAGCCACCCATTAATAGTAATGGAACTCTGTGCAAAGTTTCCTCCGGTAATATGAGTTACCACCCCTTGATCGCTTATACTACCCCAAACATCTGACTGCATTTTCCAACTGAAATGGAATATTACTACCGAAATAGAATTGTACATCCAGAATAGTCCTAAGAAGACATGATCCCAAGCAGATACTTGACACGTTCCTCCTCTTCCAGGCCCATCACAAGGGAAACGAAAACCAAGATTTGCTTTATCTGGTATTAACCGCGAGCTACGAGCAAATAAAACACCTTTCAACAGTATCAATACCGTCACATGAATTGTAAATGCATGAATATGATGTACCAAAAAGTCGGCCGTTCCTAATGGAATAGGTAGCAAAGCTACTTTGCCACCCACTGCTACTAACTCACCGCCCCCCCAAGTCAAACTGGTGCTCGCTGTTTCACCAGGGGCTGTTACACCAGGTGCTAAAGCATGGGTATTTTGTATCCATTGAGCAAAGACTGGTTGTAATTGTATAGCAGTATCTGAAAACATATCTTGTGGACGCCCTAAAGCACTCATGGTATCATTATGAATATACAAACCAAAACTGTGGAAGCCTAGAAATATACATACCCAGTTGAGGTGTGATATGATTGCATCGCGATGCCTCAGGACACGATCTAATAAATCGTTGTATCGATTAGTTGGATCATAGTCTCTTACCATAAAAATGGCTGCATGCGCAGCAGCACCAACTATGAGAAATCCACCAATCCACATGTGATGTGTGAACAATGATAGTTGTGTAGCATAGTCAGTAGCTAGATATGGATAAGGGGGCATGGAATACATATGGTGAGCTACAACAATAGTTAAAGAGCCTAACATAGCCAGGTTAAGAGATAATTGAGCATGCCATGATGTTGTTAGAATTTCATATAAACCTTTATGGCCTTGGCCTGTAAATGGACCTTTATGAGCCTCTAAAATATCTTTTAGACCATGACCAATACCCCAGTTGGTCCTATACATATGACCTGCTATTAGGAAAAGAATTGCGATAGCTAAATGATGATGTGCTGTATCGGTTAACCATAGACCCCCGGTCACTGGATCTAATCCACCACGAAAAGTAAGAAAGTCTGAGTATTTTGACCAATTCAAGGTAAAAAAGGGGGTTGCTCCTTCAGCAAAACTTGGATAAAGTTGAGCCAAAAGATCCCGATTCAAGATAAATTCATGAGGAAGTGGTATTTCTTTAGGATCTACTCCAGCATTTAGAAATTGGTTAATCGGTAAAGATACATGTACTTGATGTCCTGCCCACGAAAGGGACCCAAGTCCTAGTAGCCCTGCTAAATGGTGATTCAACATAGATTCTACATCTTGGAACCAAGCCAATTTTGGAGCTGCTTTGTGATAATGGAACCAACCAGCAAAAAGCATTAAGGCTGCGAAGACCAATGCGCCAATTGCGGTACAATAAAGTTGTAATTCACTAGTTATTCCAGATGCTCGCCAAAGCTGAAAAAAGCCAGAGGTTATTTGTATTCCTCGGAAGCCTCCGCCCACATCTCCATTCAGGATTTCTTGGCCCACTATTGGCCAAACCACCTGAGCACTAGGTCCAATGTGAGTAGGATCACTCAGCCATGCTTCATAATTGGAAAAACGAGCACCGTGGAAATACATGCCACTCAGCCAAAGAAAGATGATAGAGAGTTGGCCAAAATGGGCACTAAATACTTTTCTAGAGATTTCCTCCAAATCACTGGTATGACTATCAAAATCGTGAGCATCAGCATGTAGGTTCCAGATCCAAGTGGTAGTATCAGGTCCCTTAGCTATTGTTCTTGAGAAATGACCGGGTTTAGCCCATTCCTCGAAAGAAGTTTTTATGGGATCCCTATCTACCAAAATTTTGACTTCTGGTTCCGGCGAACGAATAATCATTGAGTCCTCCTCTTTCCGGACAACACATACAAAGAAACCCGCCAACAGTCACTCAAATAATTAGTGAACCGATGATAGATGCTTAGAATTTTGTTCTTTCTCTTCTATCTCCCATCTATTCATCCATTTTCTTTAGTTATTCACTAGAGCAATTATGATCTGGAAGTCGATCTGGGGCAAGTGTTCGGATCTATTATGACATATCCATAGGGTGCTCAACGGACCCCCCCCCCCCTTTTTTTTTTTATTAAAAAGCGTTTTCGCACCTTTACATTAGTATTGGTACACAAATAATTTTTTTTTATAACCTAATCTAGTGTATTCATATTTCAATTATAAGTTCCGAAATATAGCCTATTTTTTATGTTTTAAATAGAGGATATTATCCTATTTCAATAACCGCTTATTAGTCATTACTAAGAAACATTCTAGTATTGATATTTAGTCATTTTCAAATCCCTTTTATTCGTTTTAATAGTCGAAAAGAAAAAAATAGAAAAAACTAGATATAGATATTATAGATATTCTCATATTCATGTACTACTTATCCCTAGAGAATACCAGATTAAATAGAACGATTTGAGAAAAGGATATAATGAAATTTTTTTCTGGGATTGGTTCTTCTGATAGAAAAAAGAATCTGGTTTATTTGACCGAGAGGGCCAAGAAACTAAAAAACAATTAATTGTAAAAACAAATAAAGATATATTATAGAATAAAAAAAAAAGAAACAAAGAAAAAGTTCTTATTCGAAGCGCCTCGTGATCGTCAACCAATTCTGTGCTTCAATATAATTACCAGGAGTAAGCGTTATAGCCTGTTTCCAATACTCGGCGGCTTGAGCAAACCAAGCCTCCGCCATTTCAGAATCTCCTTGTTGAATGGCCTGTTCTCCACGGTCGGAATAGGCGGGTCAATTCCCTCCCTGAGAACCGTACTTGAGAGTTTCCTACCTCATACGGCTCGACAACCAACTCTTTTGTTTTGGTGTACCAGTTTTTTTCACTTTAACCTACTTTCACTTTATATCTAATTGAATGAGATTTCTTATAGATATTCATTCGGTTTTTCTTGGATTAAACAAAAGAGAGTAATTACATGAGTTTCAAACTTTCGTTTTGATTTAATTAATATATTAATTAATCTAATAATAAGTTTTATCTTTTCTCCTACCTTCAGAAAAAAAAAGGCATGTCCACTGTTATTAGATATTAGAATTTTCTGAAAGGTAACTATCCCGCTTTCATATATAAATTTATATAGAATCGTTGAAAAAGACTTTTTTTCATACTTCATAAAAAAGAAAAAGACTTACTGTCTTTAGGATCTGATGCTACACCGCTGCTCAATACCTTAGGGGATCCACTCTATTACATAAGTAGATTCCTAAGATTTATCTCATATTATGATATAAATAAACAGCTCTTGTTGTATCGGTCCAAAACCTTTCCAGTTGATCTTTACGGTGCTTCCTCTATCAATTAAATCTTTTTTTATCCATAGAAATAAAGTATTTAGGCATATCTAGTCTTCACTTCATATTTCGATCCATGAAGTTTATTTATTTGCTACAGCTGATAAAAAATCGTTTTGGACGATGCTTATGTAGAAAGCCTTTTTTTTTTCTAGTATTTCATTGACTAGCTGTTCGTTCTTTTTTTCTATAGTGGAGATAGTCGCACGTAATGACAGATCACAGCCATATTATTAAAAGCTTGTGGTAAAAAGGGGTTTCGTTCTAATGCCCGAAAATAATATTCTAAAGCTTTGGTATGTTCCCCATTACTTGTGTGGATAAGGCCTATATTATAGAGTATATAACTTCGATCATAGGGGTCAATTTCTAGTCGCATAGCTTCATAATAATTCTGTAATGCTTCCGCATAATTTCCTTCAGATTGAGCCGACATCCGTTACGGTCGTCATTCGCTTTAACGAATTCTCCGTTTCAGAACCGTATGTGAGATTTTCATCTCATACGGCTCCTCCTTTAGGTGCATAATGAAAATAATAAATATATGGATATGGAAAAATTTGATGTCATTATGAACTAAGCGGGGCTAATGTTTTTACAAGAAATCCCTAGCCAACCTTCTTGTAAAAGATCTTTTCTTACTACCAAGTGGATTCATATTCATACTAGATAAAAATAAAAAAGGAAACTCTAACAATTTCTTTGTTCTCAACGCCCCTAAATTTCCAGGAATTAGTCACTTCAACAGTCTTCAATGGTTATACGGGTATCCAAAGTACGGACGAGATGGATGTTTATTGTTCCAACCATTTTAATTAGTCCCAATCCCAAAGAAGAAGAAAGAAAAGGAATCTTTTTGAAGAAAGTTTTCGTGTTGTTGATTTCTCGGCGTAGTGCTTCTTCCCCTGTGCCTCCTATTCGTATATTGTATTAGTCTAGTAGGATTGATCTGTAATACGGGAACCATAGGTAAAAACCTTTTGCTCAATACTAGAATTCATAATTGAAGCATCTAAGGCTGCACTAATCGTGGATACATGACAGAAGGGATTGCTTTTTTATATTATAAACTTCACCTTCAAAAGCGTAGATTTTTTTCAATACTCATTTTTTTCTATTCCAAATCGGTGAGAAATAGAAAAAAATGATAATGATAATCAAATCGCACCATCTCTGTAATAAGTAAATGCCTCTTTTTCTCCGGAAGTTGTCGGAATGACTCGTAATAAGATATCGGCTACAATTGTAAAGGTTTTATCAATAAAATTTCCATTTATACGCGATCTTGGCATAGGTAGTAATCCATTCTATAACTCTTTTTATTTCCTTTAACTTTTCTTTTGTGAGAAAATTTTCTCACAAACAAAGGAATTTTATAGTACGAACTAACATAAAAGCGGACTCGTTTTTTATAAAAAAATATTCTATCTACTTCCAATTTTTCCGATCAAAAAAGGTATCTATTAACCATAATCTAAAAAACGATGAATAACTCGCTATTCACCCAGGTACTCAGTCATAATCCTGATGTCGGAGAGATGGCCGAGTGGTTGAAGGCGTAACATTGGAACTGTTATGTAGACTTTTGTTTACCGAGGGTTCGAATCCCTCTCTTTCCGTACTTTCAACTAAATAACCAATCTTACGTGATTGACCACAACGTATCAAATCAAATAAAAAAAATAGATAAAAAATCTACATTTCTTTGCTATGGAAAATGCTGGGAAGAGCAAACAAGGGATCCAAACCTCCCTACCAATCTATGATACATGAATAGGAAAAAGATTCCCCGACAAAATCCCTTACCTTGTCCCTTTTTAGTTTTAATCAAAAAAGCGGGCAAAGGGGAGTTGTCCGAACTCTTGTTTTTAGTGATTTTTTTTTACTTCACTTAGGTTTTTTAAGTCTGGCGAGAGTAATATTCTACGACAAGCAATTCATTTATTTTCAAACCGACGCATTTCCTATCTATTATTTGATTGACTAACCCTTCATATTGGAATGTGTGAAGAGTCAGATGGTTTGGCAATTCCTCGGGGGCAGATGACTCAAGAAGATTTTGAACCAAAGTTCTAGAGTTTTGTTCATCCTTCACTGTAATAATATCTCGGGGTTTGCATCGATAACTTGGTATATCAACTATACGACCATTAACTAAAATATGCCCATGGTTAACTAATTGGCGCGCTTGAGGAATAGTCAAAGCCATACCCAACCGAAAAAGGATGTTATCCAAACGCATTTCAAGTAATTGTAATAAAACTTGACCCGTTGACCCCTTGGCTTTTCCGGCGATACGAACATATTTAAGTAATTGGCGTTCTGTAAGACCATAATGAAAACGCAATTTTTGTTTTTCTTCTAAACGAATACGATATTGAGATTTTTTTCCGGAGCGTGATTGGTTTCTAAGATCGCTTCCTGCCCTAGGCCTTTTACTAGTTAGTCCCGGTAAAGCCCCCAGACGGCGTATTTTTTTAAAACGAGGCCCTCGGTAACGTGACATAAAGACTCCTTTTTTTATTGAAATTGTACAAAAACTAAACAAAATTAAAACTGAACTAAATGATAATGATAAATAACGTAAAATCCACTCCAATAGTTTATTGGAATACAAAGAGTCAGAAGATATATTCTCTCAATATACAGATTTTTTTTATTGTATATACAATATATATAAATCAATAAATCACAAAAATTTTCCTTTATTTTCTTCATTTATTTTGCCAAGATCTAACCCTTTTACCCCAATATATATTCCTATATGGAAGTTTATATGACATAATATAAATGGCGTGGTAACTCTTGGAAAAAGGTGAAAGAAGTCTTTTCAATCTTATTTTTTTTGAAAGTACATTAAAAATCATGTAAAAAAAATGAAAAACTATGTAAAAGCCGGCTATCGGAATCGAACCGATGACCATCGCATTACAAATGCGATGCTCTAACCTCTGAGCTAAGCGGGCTCAACTAAAATAGTGTATACAAATTCACTAAACTACTAGATCGTATTAATTAACTATTCTATTCATATTTTTCCTTATCTATTTAGAATTCATCATATTTTCGATATTCTAGAACAGAATATAGCTCAAATAAATAGTGACTATCATTAAATAAATAAAACAAAACCTTAATGAATTAATATAATATAGCAATATATCGACTTTCTAATTTTGATTTCATGAGTTTCTAAATAAGAAAATTTTAATTAGACCGGAAAGCTTTTTTTTTAAGTTAAATGATATCTGATTTGAAATTCTTGGTTTTTTTGTTCTAACCTCATGCAATTATTATTATTTGATACTTTTTCTCTTTTTATATTCTTTATTATTTTATAGAATTATTAGAATGAATATTCGAATATTCATTTCGAATATAATTTTTTAGAATTATTCGAATTTCAAATCTACGAAGTAGACTTATAATCTTTTTCCATTGCACATTCTAGAATTCTAAGTTTCAATAATGATCATAAATTTCTTTTCATGGAAGTAAAAAAAACGAATCGACCGTTCGACTATTTCTTAAAATTGAAGACAACGATGAGAAAAGGAAGAACATATATATGTTCTCTAATATATAACCATATTGAATTGCAAATACAAAAATGATAGAATCTTTGTTGATTAAACTAAATCAATATGGATGGGGCTAAAAAAAATGCAAGAAGATACCAAAGAAATAAAATAAGTATCTGTATGTAATGAATTCCAAGGTTTCGTCATAAGAAAAAGTGGAAAGACATCATAATGAGATCCTAATCTCAAAGCAAAAAGGGGGATATGGCGGAATTGGTAGACGCTACGGACTTAATTGGATTGAGCCTTGGTATGGAAACCTACTAAGTGATAACTTTCAAATTCAGAGAAACCCTGGAATTAACAATGGGCAATCCTGAGCCAAATCCTGGGTTACGCGAACAAACCAGAGTTTAGAAAGCGGGATAGGTGCAGAGACTCAATGGAAGCTGTTCTAACAAATGGAGTTCAATCCCTTGTGTTGAATCAAACGATTCACTTCATAGTCTGATAGATCCTTGGTGGAACTTATTAATCGGACGAGAATAAAGATAGAGTCCCATTCTACATGTCAATACTGACAACAATGAAATTTATAGTAAGATGAAAATCCGTTGACTTTTAAAATCGTGAGGGTTCAAGTCCCTCTATCCCCAACCCTACTCCCTAAAAAAGTCTGTTTGACACCTTACCCTTTTTTTAGTTATTCAAGAATTCATTGATCTTTTTTCATTCATCCGACACTTTTACAAACTCGAATTTCTTTTCTTATTATATACAAGTCTTGTGGGATATATCATACATATACAAATGAGAAAGAACTATCGATTTGAATTATTTCGAATCTAAATAATTTTTCATTCTAAAACTTAGAAAGTCTTCTTTTCGAAGATCCAATAAATTCCCGGTCCAAAACTTTTTTCATTTACTACTTTTGCGTTTCTTTTAATTGACATAGACCTAAGTCATCTCATAAAATGAGAATGATACTTCGGTAATGGCCGGGATAGCTCAGTTGGTAGAGCAGAGGACTGAAAATCCTCGTGTCACCAGTTCAAATCTGGTTCTTGGCATAGGGCAGAGGACTGAAAATCCTCGTGCCACCAGTTCAAATCTGGTTCTTGGCATAGGATTGATTAATTTTGATAAGTTTATAGTCTTCAAATTAAACGTATCTTTAGTAAAAAAAGTGCAATAATCCTTTATCCCCCTCTCTTTTTTGTTCATGTTGTGGATCCATCCGTTCAAAAAAAATGTATAAGACTTTATACCTAATACATATTCGAAAGGAAAGTTCTGGTTGAAAGAATAAAAAAAAGTAAAAAAAAAGATCTATATCTATCTATCTATATCTATCTATAGTATCTATCGTTGAAGGGCAGAAATACCCCCAAGATTCATTAGATTAGATACAATAGAAATAGAATTTTAACCCCCCCCCATTTATTGTATTGCTTTCCAATCTTATTTATTCATTCCCAGTTATGTGACTAAAGTTGACTAAGTTATGTGCGCGATACAAAGTTCATAATGCAGAACTCTTTTTTTTTTTTTTAGTTCATCCTATTGGCTCGGCTTTTAGGAAAAAAGTATCTTTCAAATTGGAGATTAAGCTATCTATAATAATATGAATAAGACCTTAATTCTTCTGTTTGTTTGATCTAAAAACGACTCGAATTCGAAATATTCCGCGAAGGTCCGTAGTTGTAGAAACTAAGACTCATTTTTATCATTCAAATTTTTTATCATTCAATAAGCATCTTGTATTTCATAAAAATTGGGGGCAATATAATCCTTACGTAAAGGCCACCCTATCCAACTTTCGGGCATTAAGATCCGTTTCAGCCGCGGATGGCTATCATAAGTGATTCCTAACATATCATAAGATTCCCGTTCTTGAAAATCCGTACTTTTCCAAACCCAGAAAACAGATGGAATTCTGGGATTACTCCTGTGAGTAAATACTTTTATGCAAACTTCTTCCGCTTGATTGACACCATATTCTATTCTCGTAAGATGATACACACTGGCTAAGAGGCCACCTGGTGCCACATCATAGGCACATTGGGAACGTAAATAATTGTAACCATATACATATAAAATTACAGCAATAGAATGCCAATCTTCGGGCTTTATTTGTAAAGTCTCTATTCCTTGGTAATCGAAGCCCAACGATCTATGAACCAGCCCGCGTTTGGCTAGCCAAACGGACAAAGTGCCCTGCATCTTTTTTATTTCCCCCACACCTTTTTTATATAAATTTAAGTATTTCACATTTACCATGAGTTCTAATTTATGAAGATTTTTTTCTTATTCTCTCAAATCCTCCCTAATTCACTAATTCGTGGGAAGATACTGGGCTTTTGTATTTAAAAAATGTTTCAGTAGAGATCTCTGAAGTAGATGATGGTGGATAGAGTAATTCTTGATCATAATTTCCAGTCTGTGTACTGCGTACAACAAAAAACTTGTGATTGGTAGTAAAACACCGATTACCCCGTTGAGGTCTAATTCGATCCTTATAGATTTCTCTAGCTATTTTCTTACGAAGCTTTGTTATAGCGTCTATAACAGCCTCTGGTTTAGGTGGACAACCCGGCAAATAGACATCTACAGGAATTAGCTTATCAACCCCTCGAACAGTACTATAAGAATCGGTACTGAACATCCCCCCTGTAATTGTACACGCTCCCATAGCAATAACATACTTTGGTTCAGGCATTTGTTCATATAATCTCACTAAAGAAGGAGCCATTTTCATTGTTACTGTACCTGCTGTTAAAATAAGGTCCGCCTGTCTAGGACTTGATCTTGGTACTAGCCCATAACGATCAAAGTCAAATCGGGAGCCTATTAATGAGGCAAATTCAATAAAACAACAACTGGTACCATAAAGAAGCGGCCATAGGCTGGAAAGTCTTGACCAATTTGAAAGATCATTTAACGTAGTTGAAATAACTGAGTTTTTTGTTGTTCGATCAAGTACGGGAAACTTAATGGAATTCATAATTGTTTCAATGGTTTTTTTTTACTTTTTTTTGATTGTTATTGTACAAGTATTCAGGAAACGAACTAAGACCATTCCAACGCTCCTTTTCGCCATGCATAAACTAAACCAAGAATTAGGATAAGCACGAAAATGAAAGCTTCTATAAAAGCGGATACCCCTAGTACATCGAAACTCATTGCCCACGGATACAGAAAAACGGTTTCAACATCAAAAACAACAAAAACTAGAGCAAACATATAATAACGGATTCTAAATTGTAACCAAGCATCCCCGATCGGTTCTATACCTGATTCATAACTAGAAAGTTTCTCCGGCCCCTTCGTAATTGGAGATAAAACCCCGGAAATTAGAAATGCCAAAACAGGAATAGCACTTGATATTATTAAAAATGCCCAGAAAATATCATATTCGTAAAGCAGAAACATAGACGAACTCCTATGAATGTGGAAAAAATACCCGCTTAGTCAATTCCAATCGGAGTGGATTGGGCAAGGTATATATAACTCTTGCGTCAAAACAAAAATTCAGGTTAATCGAATCATTTATTTTCGTTTGGTTGCTGTGGTAGACGTCTCCTTTTAAGATTTATTGATTGTAATCTTATTTTCAGTACACTTATTACTTAATATTTCCATGTTTCTATTACTAATAGTTTCTCATATTAATAATATAATATTAATATGATTAATAACTAGTAATTTTTTTTATTTCTGTTTCTTAAATTTGCTTTATGTTTTATTTAAAAATAAAACAAATTGATAAAAATATCTTCGTTTTTAAAATTATGACGTATCAAAAAATCCACTTACGACTATGAAAATGAATGAATAAAAAACGTTTATTCTAAATTATAAGTATCTATCTAGATATATCGATAGATAGTGATTGGATCCACTGAAATCAAATTTGGTTTTCCGTTTTATTCTGAACGACCCCCAGGACTTATGGTTTAGGGTCTGGGAGTTTTTTTTATGAACCAACAAATTGAAAGTAACCAGTTAGAAATAAAGAATACAATAATAAGTCAAAAATTATCCAATTATTTGGATTTGAATGTCATTTATTAGAATAAATTTATTAGTTAGGGCTATACGGATTCGAACCGTAGACCTGCTCGGTAAAAGAGCTCGAACTTATTATTATCAAAATGATTCGAACTCTTTCAAAGACCCAACATGCATTTTTTTTTGCATTGGGCTCTTTCATTAACTGATAGAAAGATCAGTTAGTCTACCATATTTTTTCTTAAAAAAAAAAGATAAGAAATGGTTCCAAGTACTCTGATTGATTATTTTTTAATTCTATAATTCTAATACAATACAGAATAACTACCAAAGTGTTTCAAAGAAGGGTTCTCTTGACGTAGGTTTGCTTTTGGTCTAGATCAACTTAAGTTAAATATAGTCTCTAACATCCTGATTAAAAAATCAAATATGAAACTTGCTACACCTTAAGGTTCATAGGACGAAAAGATCATTTTTGAGTTCCTTATACTCATTCTGCCTAGCATTAAGTAGACTGGGTATTCACCCTATCAATATCTCAAATCAATGATGGGTTCTATTAATTCCCTACCGAAATGGGGTACTTTAATAGGACCTAATGTCAGGCTATTGTTCTCCTCTTTTTCCTAAAAAAAAGTCATGGAGTAAGACATCGATTTATTAATAAGATCAATCAATTGGTTTGATTGCGTGATGGACTCCTCTGAAAAACTTTGGCGCACGTGTAAACGAGGTGCTCTACCTAACTGAGCTATAGCCCTTGTGTTTATGATCCACATTTTATCTTATCATGTAGATAATTTCTTGTCAAGATTAATATTATATGATCGAACATTATATCTCTTTCATCTCGTTGTTTATTGGTATTGCTTAGAAATAATATTGGATTTATAATCCTATCGATGTGATAAGTATCCCCGTGCCTTCTCTTTACGATGATAAATAACCTACTTAACTCAGTGGTTAGAGTATTGCTTTCATACGGCAGGAGTCATTGGTTCAAATCCAATAGTAGGTATAACTTATTAGACACCATGATCAATGGTGTCTAATAAGTTTTTGTAGCCAGCTTTTTTTTTTTTTTTTTCTCGCTTTTGGATCCTATTTTTTTATACGTCAGCTAGTTACAAAATCAAATCGTATTGAGAGCCTCGACGCGTGTCCGAGCTCGTCTGAGAGCTAGATTTGCCTCAATTGTTTGTCTCTTGCCTTCAGCTTTTCTCAAGTTCGCCTCTGCTATTTCAAGAGTTTGCTGAGCTTCTTGTGGATCAATGTCACTATTCTTCTCTGCATCATTTACTAAAATAGTAATTTCATTATTGCCTATTCTAGCAAAACCGCCCATCAGAGCCATTGTTAACCATTGGTTATTAAGGCGTATTTTCAAAATACCTATATCAACAGCTGTGGCAATCGGCGCGTGATTTGGTAATACGCCAATTTGTCCACTATTAGTAGATAAAATGATTTCTTTTACTTCTGAATCCCAAACAATTCGATTCGGAGTCAGTACACAAAGATTTAAGGTCATTTCTTCAATTTACTCTCCATTTCTAAGTTCGTAGCCTTCGCAGTAGCTTCATCGATGTTACCCACTAAGTAAAAGGCCTGTTCAGGAAGAGAATCAAATTCTCCGGAAAGGATCAAATTAAACCCTCTAATTGTTTCCGCTAGCCCAACATATTTTCCCGGAGAACCTGTAAATACTTCTGCTACGAAAAAAGGTTGTGATAAGAAACGCTCAATCTTTCGTGCTCTTGCGACGGTTAAGCGATCCTCTTCGGATAATTCGTCCAACCCCAGGATAGCTATAATGTCCTGAAGCTCCTTGTAACGTTGTAAAGTTTGCTTTACTTGTTGCGCAGTTTCATAATGTTCCTCGCCAACGATTCGAGGTTGTAGCATAGTTGACGTTGAATCTAAAGGATCTACCGCTGGATAGATACCTTTAGCAGCTAATCCTCTTGATAGTACGGTAGTCGCATCTAAATGTGCAAATGTGGTGGCAGGAGCAGGGTCAGTCAAATCGTCTGCAGGTACATAAACTGCTTGAATAGAGGTTATGGACCCTTTTTTCGTAGAAGTAATTCTTTCTTGTAAAGAACCCATTTCGGTACTAAGGGTGGGTTGGTAACCCACAGCAGAAGGCATTCTACCCAATAAAGCGGATACCTCGGATCCTGCTTGTACAAAACGGAAGATATTGTCGATAAATAGAAGTACGTCTTGCTCATTAACATCTCGGAAATATTCTGCCATAGTTAAGGCAGTCAGACCAACTCTCATACGAGCTCCCGGCGGTTCATTCATCTGACCGTAGACTAGGGCTACTTTGGAGTCCGCAAGGTTTAGTTCATTAATGACTCCAGATTCTTTCATTTCCATGTAAAGATCATTTCCTTCACGAGTTCGTTCGCCTACTCCACCAAATACGGATACACCACCATGAGCTTTGGCAATGTTGTTGATCAATTCCATAATTAGTACTGTTTTACCCACGCCAGCCCCACCGAATAGTCCGATTTTTCCCCCACGACGATAAGGGGCCAAAAGATCTACTACTTTAATTCCTGTTTCAAAAATAGATAAGGTTGTATCTAAGTCTATAAAAGCAGGCGCGGATTTATGGATAGGAGATGTTGTGAGAGTATCGACAGGACCTAAATTATCAACAGGTTCCCCAAGTACATTGAAAATTCGTCCTAGAGTCGCTCCGCCGACTGGAACACTTAGAGGATTTCCCATATCAACCACGTCCATCCCTCTCTTTAAACCCTCGGTCGCGCTCATAGCTACAGCTCTAACTCGGTTGTTTCCTAATAATTGCTGTACTTCACAAGTCACATTAATTTCTTGACCAAGCGTATCTCGACCCTTAACCACCAGAGCATTGTAAATATTAGGCATCTTGCCCGGGGGAAAGGCTACATCCAGTACCGGACCAATGATTTGGGCAATACGTCCCAGGTTGTTTTTTTCACGTATTGAAACCGCTGGATCCGAAGTAGTAGGATTTATTCTCATAATAAAAAATATGTTCAATTTTGTTGCGAAATTTTTCGAATACAGAAAAAATCTTCGTATAGTAAATTCATTGGTTAATTCAATAATAAATGGGAGTAAGCACTCGATTTCATTGGTACCACCCAAGCGAATATGCAATTCAATTTTTTACTTAATTAAATTTCAATGAAGGAATAGTCGTTTTCAAGCTCAACTAACCAAAACCTAGTTTTAAAATAAAAAATATATGAATAAAAAAAATTTTTGTGGAAAGTCTTTGATTTATTTGTCATAATAGGCAAGACTTTGTTTTATCTAGCCAATTCCGAAATGGAACTCTATTTATGATTCATTATTTCGATCTCATTAGCTTTTTTTTTTTCATATTTTCATTTTAGCATATCCGGTTATGCGTCCCATCGATATCAACCCCCCCCTTGTTTTTCATTTTCATGGATGAATTCCGCATATTGTCATATCTAGGATTTACATATACAACATATATTACTGTCAAGAGTGATTTTATTATTATTTTAATATTAAATATTTCGATTTATAAAAAGTCAAAGATTCAAAACTGGAAAAACAAGTATTAGGTTGCGCTATACATATGAAAGAATATACAATAATGATGTATTTGGCGAATCAAATATCATGGTCTAATAAAGAATCATTCTGATTAGTTGATAATTTTGTGAAAGATTCCTGTGAAAAAGGTTAATTAAATCTATTCCTAATTTATGTCGAGTAGACCTTGTTGTTTTGTTTTATTGCAAGAATTCTAAATTCATGACTTGTAGGGAGGGACTTATGTCACCACAAACAGAGACTAAAGCAAGTGTTGGATTCAAAGCTGGTGTTAAAGAGTATAAATTAAATTATTATACTCCTGAATATGAAACCAAGGATACTGATATCTTGGCAGCATTCCGAGTAACTCCTCAACCCGGAGTTCCACCTGAAGAAGCAGGGGCTGCGGTAGCTGCTGAATCTTCTACTGGTACATGGACAACTGTGTGGACCGATGGGCTTACCAGCCTTGACCGTTACAAAGGACGATGCTACCACATCGAGCCCGTTCCAGGAGAAGAAACTCAATTTATTGCGTATGTAGCTTACCCATTAGACCTTTTTGAAGAAGGGTCTGTTACTAACATGTTTACCTCAATTGTGGGTAACGTATTTGGGTTCAAAGCCCTGGCTGCTCTACGTCTAGAGGATCTGCGAATCCCTCCGGCTTATACTAAAACTTTCCAGGGACCACCTCATGGTATCCAAGTTGAAAGAGATAAATTGAACAAGTATGGACGTCCCCTATTAGGATGTACTATTAAACCTAAGTTGGGGTTATCCGCGAAGAACTATGGTAGAGCAGTTTATGAATGTCTACGTGGTGGACTTGATTTTACCAAAGATGATGAGAATGTGAACTCTCAACCATTTATGCGTTGGAGAGACCGTTTCTTATTTTGTGCCGAAGCTATTTATAAATCACAGGCTGAAACAGGTGAAATCAAAGGACATTATTTGAATGCTACTGCGGGTACATGCGAAGAAATGATGAAAAGAGCTATATTTGCCAGAGAATTGGGAGTTCCTATCGTAATGCATGACTACTTAACAGGGGGATTCACCGCAAATACTAGTTTGGCTCATTATTGCCGAGATAATGGCCTACTTCTTCACATCCACCGTGCAATGCACGCTGTTATTGATAGACAGAAGAATCATGGTATGCACTTCCGTGTACTAGCTAAAGCTTTACGTCTATCGGGTGGAGATCATGTTCACGCGGGTACAGTAGTAGGTAAACTTGAAGGAGACAGGGAGTCAACTTTGGGCTTTGTTGATTTACTGCGCGATGATTATGTTGAAAAAGACCGAAGTCGTGGTATCTTTTTCACTCAAGATTGGGTCTCACTACCAGGTGTTCTACCTGTGGCTTCAGGGGGTATTCACGTTTGGCATATGCCTGCTTTGACCGAGATCTTTGGAGATGATTCCGTACTACAATTTGGTGGCGGAACTTTAGGCCACCCTTGGGGAAATGCACCGGGTGCCGTAGCTAACCGAGTAGCTCTAGAAGCATGTGTACAAGCTCGTAATGAGGGACGTGATCTTGCAGTCGAGGGTAATGAAATTATCCGTGAGGCTTGCAAATGGAGTCCTGAACTAGCTGCTGCTTGTGAAGTATGGAAGGAGATCACATTTAACTTCCCAACCATCGATAAATTAGATGGCCAAGACTAGAAATTAGATTAGTAATTCACGTCCGTTTTATTAGTTTAATTGCAATTAAACTCGGCTCAATCTTTTTTTTACTAAAAGGATTGAGCCGAGTTTATCTAGTGTATATACTGTTTTTGATAGATACATACTTAATCTAGATATACAAAATCTGAAAAAAAAAGAAGATTAAACACAACTACACTTTTGTATTGTAGTGTCCACAAGAAATTCTATACGAAATATGGATTCTTAGGATTTTTTTATTCTTTTTTTAAGTTTCGTGTCAGGGCTTGAACCAAGTATCCCCACTTCTTCTACCCATTCTGCATGTTGTCCTTTTCTTTTCATTCCGTATTGGAATAAAAACCTTTTTTTTATATTAGTATACGAGATTTTACTAAAAAAGTTCTTCATATCGTTATATTCATAAGCGAAGAACAAATATTTCTTTTTTTTAATGAGAATTTTACACAATATAAGAAAATCCTTATTTTCATTTAGAATTGAAATTTATTAATTTCAATTGCTTTTACTTAATAATCTTAGCAATTAGCAATTGCATTGACATGCTTTGCTTACTCTGAATAGAAAATGAACTATTCAAATTTTTTTTTTTGCATTTTTCAATTTTTTCATTGAATGACTATTCATCTATTGTTATTTTATTTTCATGTAAATAGAGGCCAGAAGCTCTATGGAAAAATCGTGGTTCAATTTGATGTTTTCTAAGGGAGAATTGGAATACAGAGGCGAGCTAAGTAAAGCAATGGATAGTTTTGCTCCTATTGAAAAGACTACTATAAGTAAAGACCGGTTTATATATGATATGGATAAAAACTTTTATGGTTGGGGTGAGCGTTCTAGTTATTACAATAATGTTGATCTTTTAGTTAACTCCAAGGACATTCGGAATTTCATATCGGATGACACCTTTTTTGTTAGGGATAGTAATAAAAATAGTTATTCTATATATTTTGATATAAAAAAGAAAAAATTTGAGATTAACAATGATTTGAGTGACCTAGAAATTTTTTTTTATAGTTATTGTAGTTCTAGTTATCTGAATAATAGATCTAAAGGTGACAACGATCTGCACTATGATCCTTACATTAAGGATACTAAATATAATTGTAATAATCACATTAATAGTTGCATTGACTCTTATTTTCGTTCTCACATCTGTATTAATAGTCACTTTTTAAGCGATAGTAATAATTCCAATGAAAGTTACATTTATAATTTCATTTGTAGTGGAAGTGGAAAGATTCGTGAAAGCAAAAATGACAAGATAAGAACTAATAGTAATCGTAATAATTTAATGAGTTCTAAGGCTTTCGATATAACTAAAAACTACAATCAATTGTGGATTCAATGCGACAATTGTTATGGATTAATGTATAAGAAAGTCGAAATGAATGTTTGTGAAGAATGTGGACATTATTTGAAAATGACCAGTTCAGAGAGAATTGAGCTTTCGATTGATCCGGGTACTTGGAATCCTATGGATGAAGACATGGTCTCTGCGGATCCCATTAAATTTCATTCGAGGGAGGAACCTTATAAAAAGCGTATTGCCTCTGCTCAAAAAAAGACAGGGTTGACTGACGCTATTCAAACAGGTACAGGTCAATTAAACGGTATTCCGGTAGCTCTTGGGGTTATGGATTTTCAGTTTATGGGGGGTAGTATGGGATCCGTAGTAGGCGAAAAAATAACTCGTTTGATCGAGTATGCTACCAATCAATGTTTACCTCTTATTTTAGTGTGTTCTTCCGGAGGAGCACGAATGCAAGAAGGAAGTTTAAGTTTGATGCAAATGGCTAAAATTTCTTCGGTTTTATGTGATTATCAATCAAGTAAAAAGTTATTCTATATATCAATTCTTACATCTCCTACTACCGGTGGGGTGACAGCAAGTTTTGGTATGTTGGGGGATATCATTATTGCCGAACCCTATGCCTATATTGCATTTGCGGGTAAAAGAGTAATTGAACAAACATTGAAAAAAGCCGTGCCTGAAGGTTCACAAGCAGCTGAATCTTTATTACGTAAGGGCTTATTGGATGCAATTGTACCACGTAATCCTTTAAAAGGTGTTGTGAGTGAGTTATTTCAGCTCCATGCTTTTTTTCCTTTGAACAAAAATGAAATCAAATAAAACAGTTAGTTTATCATAATTAAACGAAAACCCTGAAAAATTCATTTTTCTTTAGAATCATTTTTTTATCGATATTCTTGTTTACTACTCAGTAAACCTTTATCAACAAGATAAAAAGTGAATTTTTGCTTTCGGGAAGTTCAAATTCGACTAGAAAAATAAAACAAAGTTTTTTTCCTCTCTTGCTTGCATATGGATAGATAATTCAAATAGAGATATAGATCTATAGAGAGTCTTGCATCGTTTTGCATTTCCCGAAAATTCCCTGTTGGTGGATCAGATTCCAATCAATTTTGTATAAAATTTTAATGGAATAAAATTTTTTCTTTATTAATGACTATTAGAAGACAAAAAGAACAAAAAGAATAATAAATCTAACAGGTAGATTATGATAATACATCTATTTTATTTTGAAAGATTAATAAGTCCATTTATTTAGTTTGGCGTTTCTTGTACCTATTTTTTTATTCTATTTCTAGTAGGTTCTATTCTATATATTTCTATTAGGTTGTATATTAGTATTCGATATATATTTACTTAAAGATACTTAGTATAATTATATAATATATATAATAGAAATAATAAAACTACAAGATATTCTAAGATATCTTTAGAATTCAGAATATAACAATAACAGGTACAAATATTAAATTGAGGTACCCCATTTTATGACAACTTTCAACAACTTACCCTCTATTTTTGTGCCTTTAGTAGGCCTAGTCTTTCCGGCACTTGCAATGGCTTCTTTATTTCTTCATATTCAAAAAAATAAGATTTTTTAGATCGGATGAGACCGAATCGTATAACTCCCCTTTTTATTTTAAAAACTTCGATTTGATAAGACCCATTTGGTAGAATATTGTATAACACATAGATTCCTACAAACATAACTAAAAAAAGTTTTTATGCATGTGTAAACGTATTATATGGGGTAACTCAATTTGCGCTCTTTTGAAAAAATGGATCATCATCGGACCGCTGGATGAAATTCAAGTCAATGTATTTATTTGTATGTATATAGTTATAGGGGATCATATAAAGGAAGGAGATTTTATTATTTTAGATATAAACAATTATATAAATTATTCCTAAAGTAAAGGTTCACAACAAAATAGTTATAGTTGATGAGAGTTACTTTGAAAACAAAAAAAGGAAAGTCATATTTTCTCAATTCCAAAAAATTGTATAACTGGATCTAATATATATGAGTTGGCGATCAGAATCTCTATGGATAGAATTTATAACGGGGTCTCGAAAAACAAGTAATTTCTGCTGGGCCTTTATCCTATTTTTAGGTTCATTGGGATTCTTATTGGTTGGAACTTCCAGTTATCTTGGTAAAAATTTTATATCGTTAGTTGCATCTCAGGAAATCCTTTTTTTTCCACAAGGGATTGTGATGTCTTTCTATGGGATCGCGGGTCTCTTTATTAGTTGCTATTTGTGGTGCACTATTTTGTGGAATGTGGGTAGTGGTTATGATCTTTTCGACCGAAAAGAAGGGATAGTACGTATTTTTCGTTGGGGATTTCCTGGAAAAAGCCGTCGCATCTTTTTACGATTCCTTATGAAAGATATTCAGTCGATCAGAATCGAAGTTAAAGAGGGTGTTTCTGCCCGGCGTGTCCTTTATATGGAAATTAGAGGTCAAGGGGCTATTCCTTTAATTCGTACTGATGAGAATTTTACTACACGAGAAATTGAGCAAAAAGCTGCTGAATTGGCTTACTTCTTGCGTGTACCAATTGAAGTATTTTGAAATGAATTCATTTTTAAAGTTTAAAGACTAAATCCTTTGGCAGTAGGAAGAAAAAACGAAAGAATTGCTTTCTTTTTTTTCAATTGAACATTAATCTATTCTTTTATGCTCGTTTTTTTATATATTTGATAGAAAAGAAAGGGAGTTTATTCGTCTCGAAAATAGAATCATATTTTTTATTTTAAAAATTCAAAAAAGTTCTTTTAGTATTGATCGAAAAAAGGGGGAATAACATCCTGGAAATACAATTTTTTCTTTATTCAAATTGTAAGTGTATTCTGAGTCTATTTCTGTATTCTTTCTAGATTCAAAACAAAGACTAAGTATTGAATCAAAAGAAAAAGATAAAAGGGATTATAGGCTCAATACATTCTATTTGAATTAGAATAGAAACTCATGCTCGATAGAAATAGTAGATCTAATAGAATCCACAAATGCGGTAGGTTCATTAACAATTCACAGATTCAAAATGGCAAAAAAGAAAGCATTCATTCCTTTTTTTTATTTTACATCTATAGTCTTTTTGCCCTGGTTGATCTCTCTCTGCTGTAATAAAAGTTTGAAAACTTGGATTACTAATTGGTGGAATACTAGACAATGCGAAACTTTCTTGAATGATATTCAAGAAAAAAGTGTTCTAGAAAAATTCATACAATTAGAGGAACTATTCCAGCTGGATGAAATGATAAAGGAATACCCAGAAACCGATTTACAACAATTTCGTCTAGGAATCCACAAAGAAACGATCCAATTCATCAAAATACACAATGAGTATCGTATCCATACAATCTTGCACTTCTCGACAAATCTAATATCTTTCGTTATTCTAAGTGGTTATTCCTTTTGGGGTAAGGAAAAGCTTTTTATTCTCAATTCTTGGGTTCAAGAATTCCTATATAATTTAAGTGATACAATTAAAGCTTTTTCGATTCTTTTATTAACTGATTTATGTATCGGATTCCATTCGCCTCACGGTTGGGAACTAATGATTGGTTATATTTACAAAGATTTTGGGTTTGCTCATTATGAGCAAATTTTATCTGGTCTAGTTTCTACCTTTCCAGTCATTCTTGATACAATTTTTAAATATTGGATCTTTCGTTATTTAAATCGTGTATCTCCGTCACTTGTAGTGATTTATCATGCAATAAATGACTAAAAAACGATTCACTGATCCAATTCTACTCTTTCTTACTTTATACATCATAACCAAATCAAAGTCGTATTTACTTTACTCTTTTTTACCCACGAGGGATTCCTTGTATATTAAAAAAAAAAAAAATTTCTTTTTTCAGTAAATGTAAATAACAGAATTGTGGCTAGGGAAGTATATTATCGACCTACCTAACTTTATTGTAGAAATTTTCGGGATAAACGATTGGACCATGCAAACTAGAAATACCTTTTCTTGGATAAGGGAAGAGATTACTCGCTCCATATCTGTCTCACTCATGATATATATAATAACTTGGGCATCCATTTCAAGTGCATATCCAATTTTTGCCCAGCAGAATTATGAAAATCCACGAGAGGCAACTGGGCGTATTGTATGTGCCAATTGCCATTTAGCTAGTAAGCCCGTGGATATTGAGGTTCCACAAGCGGTACTTCCTGATACTGTATTTGAAGCAGTTGTTAAAATTCCTTATGATATGCAGCTAAAACAAGTTCTAGCTAATGGTAAAAAAGGAGCTTTGAATGTGGGAGCTGTTCTTATTTTACCGGAGGGGTTTGAATTAGCCCCCCCCGATCGTATTTCACCCGAGATGAAAGAAAAGATAGGAAATCTGTCTTTTCAGAATTATCGCCCCAATAAAAAAAATATTCTTGTGATAGGTCCTGTTCCTGGTCAAAAATATAGTGAAATAACCTTTCCTATTCTTGCCCCAGACCCTGCTACTAATAAAGATGTTCACTTCTTAAAATATCCTATATACGTAGGTGGAAATAGGGGAAGGGGTCAGATTTATCCTGATGGTAGCAAAAGTAACAATACAGTTTATAATGCTACGGCAGGAGGGATAATAAGTAAAATTTTACGAAAAGAAAAAGGGGGATACGAAATAACCATAGTGGATGCATCGAATGAACGCCAAGTAATTGATATTATCCCTCGAGGCCTAGAACTTCTTGTTTCAGAGGGCGAATCCATTAAACTCGATCAACCATTAACAAGCAATCCTAATGTGGGTGGATTTGGTCAGGGGGATGCGGAAATAGTACTTCAAGATCCATTACGTGTCCAAGGCCTTTTGTTCTTCTTAGGATCTGTTGTTTTGGCACAAATCTTTTTGGTTCTTAAAAAGAAACAGTTTGAGAAGGTTCAATTATCCGAAATGAATTTTTAGATCTGTCTATTTCGCCTTATCAAATTCGTAAAAAAGAAAGAACCAAAAAAATTATCAAAAGCCTTTTTGCCTCTCTTTAGACTTTCGATTTCGACCAGGTGTCAGGAATTACTTGTCTGATAGTCCTAATCCTAGTATGTATATTAAGAAGAATTCACTTTACCCCCCCTTTTCTTTATTTTTCAATACAAATTTGGATTGAAAAATGGGAGGGGGTGTGATGTAACTCTGTCGTTAGTGACCAATTGAAATTGATAGAATGTATCAATAATCAAGAGTTTTTTTCTAATGTAATTTAGAATGGAAAAATTTGACTAGATACTAAAATAAGGAAAGCAAGCGCAGAAAAAAGGGAACTAGAAATTGGCGAAATAACAAATTTTAGGGACTATAGGGAGTATTACTTGTCTTGCGAGTCTTCGACACAAGAAAAGGAATTTTAGACATCCTTTTCTTGTGTCGATCTTGTCATTCTTAATTGCATTCGTTAAAAAATCCTATTCTTAGTTTACATATATATTACTGTTTCTATATATATAAGGTTTTAATATATCTATATTAATATATATCGTATATATAATTATTAATTAATAGTATAATAATAGTTACTTTTTGTAGTTTTATTTATTTTAATTTCAAGTTTGATGAAATACTAAAAAAATAAGAAAAAACTGCTATTAGTATAGAGTATAGACAAAATTTTAATGATAGATCTAATGATAAAAAATATTGTGTCAGTCGGGAAAGCAGAAAAATAAAATTAAGTGATCCCCCCCCTTTTTTTCGATCTTTGAAAGGTTAATAATAAATACTATATGCTCGTAACCTACTAATCTAATTAAGTTCATTTTTCAAAAACACGATAAAAATTGTTCTTATTATTAGCAGTTCAACGGGACCCCCTCGAATCAGACAAAGAAGGAAGAGTTGGGCCCCGTTGAGTTCTTATGTTTTCACGTCTATAACTCAGTTCATCCAATTTCTACAGGGATGAACCTAATCCTGAATATGAACCATAAAAGAAAATACCTATTAAACCGATCACAAGAATACCAGCTACAGTACCTATTACCCAAAGAGGAATCCTTCCAGTAGTATCAGCCATTTATCCCGCTTCCCTCCACATTTCATCGAGTGGTCATGCTAGAAACATAAACAGTCAGAGATAATTATGATATATAATCCATCCGAATGGGATAAGAAAATTACTACTCTTTGTTTTTTATTTTATTATTCTACGCTCTTTTCTTAATTTTACTTAATTTTAATTGAAGAAATAATTTGAAAATAAAACAGCAAGTACAAAAATGAGTAATAACCCCCAATAGAGACTGGTACGATTTAATTCAACATTTTGTTCGTTCGGATTTGATTGTGTCATAGCTCTATAAATAATTGAATTCGGTTTATCGTTGGATGAACTGCATTGCTGATATTGACCCCAAAAAAGAAACGGTAGGTACAGCTAGTCCATGAACAGCCAACCAGCGCACTGTAAAAATTGGATAGGTCCTATCTATAGTCATTGGGTCCTCCTAAAAAGATCTACTAAATTCGTCGAGTTGTTCCAAAGAATCAAAACGGCCTGTTATTAATGGAATGCCCTGTCGGCTCTCTGTAAAATACTCGTTTGGACGAGGGCTCCCAAACACATCGTAAGCTAAACCGGTGCTGACGAATAACCAGCCCGCAATGAATAGGGAAGGTATAGTAATGCTATGAATGACCCAGTATCGAATACTGGTAATAATATCAGCAAAAGAACGTTCTCCTGTGCTTCCAGACATACTGAACTCCAGATATTCTTGTAGGGAATCGATTCTGTAAAAGATGAATCAGTAAATTCAAATTCACTGAGATTACATCTTTGTGAGATCGTCAATAAAGTACCAAGGGTATTTTTAGAGTCTACCGAATCAGTATAGCTATCCTTCTTCTGACACAGCAACGCAATTTGAATTAGTATAGAACTGAAGTGTTAGATAATTTATTTCGTTTTTTTTTTTTGCTTGTCGATGTATAACCATGTTCCACTTCTTCAAATTCCTGTATCTGTAATCTATAGGGGCTTTGATCCTTTATTTGTTTTGGACTAGAAAATAAACTAAAGATTAGATAAAATGAAAATTCAAGAGGGTGGTTTCTAATTCTAATAATTCATTAAGGAAATTCTCATATTGTCCCAAGTCAATTGAATCCAAAAACCATCAATTTCATTTTTGTTGCATATGCATAATTGTAGAAGAGATTTTTTTGTAATGATAATGTGACCCCCCCCGTTTTTGCTTTTTATTTCATTTTATTTTAAGGAATTAATTTACAGTAACAAGAAAAGGAAGAATAGTTTTGGATCAATTCAAAAGAACAACAAATAATAAAAAAATAATAATACTAAATCTTTGGAATGCGTGCATTGTTTTGTTGTATTCAATTTAAAGGTTTTTTCTTTCTTTAACTAACTACAAAGATGATGGGTTTTTCACTCTATTTTCTATATAATATCGAAAGAAAAAAACCTAAAACCTAGAAAGGAAACGATAATAGAAATTGCTAATTACAAGTTTTAAAAATCTAGTTAAAATAAATAACTCTTTTTTTGACTGATCTCGTTCTCCGTGTAGGATACTGCTTTTTGGTTTAATTTCATAGATTAAATGAAGAAAACTGCTCTACTATATTAATCTACTTTATTCAAAATTGAATTTATTTCAATTTGAATAAATGTAGAAGGGGGCGTATTCTAGGTTCTAAGGTCACTTAAAAAAAAAAGAATCAAACAACTTTTTTTTTAATTTTTACATGTTTATTCAAAAAAAGTTCTATGTTTTAACTGAAGTTATATAATAGAGTTATTTTTTTTATGTATTATTTTTTCTTATTAATTTCTTAAAGAGTTTTTCAATGAATCAGTTACGTGAATTCTGAATCCTGAGATGGTGCAGATGCCAAAGACGATGAATTTCGTTTTTTCTTTTTCTCTATTTTTGTTCATACCACCGATAATGCTTGATAACTCACAAATTTTCAATTTAATTTTTTGATTCTTGGAACTAGTATTTGTATCTATCTTTACTTTTAAAAATTTTTTTATTGAAACTTCGGGAAGTACTTTAGAAACATATGTATAAAAAAACATATTTTATTGAGTCCCTTCATGCCTACTATAACTAGTTATTTCGGTTTTCTACTAGCAGCTTTAACTATAACCTCAGTTCTATTTATTGGTCTAAGCAAAATACGACTTATTTGAAATTAATTGAATGAATCTTTTTTGATCAAAAAAGATTTATATGGTATTTCATATGTTCGATAGTTCCTTACCGTGTTAATTACCCAATTTTGGTCATTGAGATTCGTCGGCAATACAGATTAAGAGCTAGGAATAGATAGTACCTCTCTTTTCTCCCTTTCAAAAATGAAAACAAAAGAAAATTGAAATGATTGAAGTTTCTTTATTTGGAATCGTCTTAGGTCTAATTCCTATTACTTTGGCTGGATTATTCGTAACTGCTTATTTACAATACAGACGTGGTGATCAGTTGGACTTTTGATTAATTAACATCTCTTTTTTTTTACTGACCTCCTTCTTGCTTTCATATGCGGGAGGTCGAATTCAGATTGCTGCTCAATTATTTGCGAACAGTGGAATTTTGACACAATCTAATAAACAAGAGTGACATCACGCTCTGTAGGATTTGAACCTACGACATTGGGTTTTGGAGACCCACGTTCTACCGAACTGAACTAAGAGCGCTTTTCTTGTTTTTTCTAAAAAAACGAAAAGGCTAGAAAGAGGACATTCTTTAACTCGAATCGATTTTGTACGTATATACTATATCATAGTATATCATAAAATTCAGAATTATATGTATGTCCAATTTTATTAAAAAAAGATAAATCTAAAATGGATTCCTCGTTACTGCTCTTCTGAGCAGTAATTAGGTAGGGATGACAGGATTTGAACCCGTGACATTTTGTACCCAAAACAAACGCGCTACCAAGCTGCGCTACATCCCTTTCGATTGGTTTACAGTGTCATTGTAAACAATTCCTATCTTGTTTTCCACATCCTTCTTTTTTTTTGTTTCATATCAGATAACAAACATATATATAAGTATAATTAAAAAAATTACTTTTTTTAGGCAAATCCTATCAATTTCAAATTTACATAAAAAGGCGTTTCCATTTTCAAATGGAATCTATAAGATCGTTCTAGTAGACAATATTTCAATTCTAATTTTGAAAATGGGGGGTTACATATACAAATACAAGAACTTCTTAACTACATGTACATCTATAGTTATATATATTACTATATATATTGTAATACAATAAAGAAGAAAGAAGGAGGATTTCAAATGCGAGATCTAAAAACATATCTTTCCGTAGCACCGGTACTAAGTACTCTATGGTTCGTTTCGTTAGCAGGTTTATTAATAGAGATTAATCGTTTATTTCCAGATGCATTAACATTTCCCTTTTTTTAATTCTAGTTATTAACATCAGAAAGGATAAAAAAATTTAGAGATACGATCAACGATCGGGGAATAACCCCCCCTTTTTTTTCTAATTCTTTTTTAAGAATAAAAAAGAATTAGAAAAAAAAAAAGGGGGCCGAAAGGGTCATAAAAACGAGGGTTCAGAATCCAATAAAAAAAAAAGTGTTGCTAGGGAAAGAGTATCCTACGAGATACTTAAAAAAAATACTGTACAAAGATTTGAAATATAGTTTTCAAAAAATCATTATATTACTTATTATTTTCTTTTTATTTAATTACTAATTAATATTCATTGCAACGAAATATTTAAGACATTTTTTTGAGTTAATTAACAGCTTCTATTTTTTTTTGTTCTTGTTCTTTATGGACCCTAAAATTAAAATAGAAGATTGGGGGTGAATCATAAATCCAAAGGAGGTTTCATGGCCAAAGGTAAAGATGTTCGAGTAACAATTATTTTGGAATGTACCAGTTGTGTTCGAAATGATATTAAGAAAGAATCGGCTGGAATTTCCAGATATATTACTCAAAAGAATCGGCATAACACTCCTAGTCGATTGGAATTGAGAAAATTCTGTCCCTATTGTTATAAACATACAATTCATGGGGAAATTAAGAAATAGATAAAATTGAGTGCTTGTATGTCAAATTTTATTTTAAGAACAGGAATAATGAGAGTATCTACGTATTATTACATATATATAAATATAAACAAATAAAATAATAGAAATAAATCAAATCCTATATTCTTAATTCTATATAGAAACTCTATCCTATATAGAAATAGAAATCGTTTTTATTTTGATCCAATCAAAATAGGATTTTATAGGTAAGGAATAAAAAATTATGAATAAATCTAAGCGACCTTTTACTAAATCCAAGCGATCTTTTCGTCGGCGTTTGCCCCCGATCCAATCGGGGGATCGAATTGATTATAGAAACATGAGTTTAATTAGTCGATTTATTAGTGAACAAGGAAAAATATTATCTAGACGGGTGAATAGAGTAACTTTAAAACAACAACGATTAATTACTATTGCTATAAAACAAGCTCGTATTTTATCTTTGTTACCTTTTCTTAATAATCAGAAACAATTTGAAAGAAGTGAGTCGACCCCTAGAACTACTAGCCTTAGAACCAGAAAAAAATAGACTTATTCTTCAATTGAATAACTAATCTGAAGGAATTAAAAAAGAGGTTAATATTTTGTTCGACAAATCCAATTAAGAATCAAAATTTGATTGTTACGTCTGTTTCTGTCATAAAAAAAAAAAAAAGAAAAGAATCGTCGAAAAGAAAAAGAATAAGTCTTTTTTTAGCGACTATATACCCTCGTTTTGTTTTGACGACTTTTTTTATAATACTAATTTCTACTCTACCCTCCCCGAGCTTATTCTACTTAAGAACTCTATTTCAAATATTTTAGTGGATTTCTTCCAATCCCCTCATTTTTTGATCTCATTTGAAATCGTATAAAGACAACTCCTATTTAATAGAGCTATTTGTGCAAGTATTTTTCGATTAAGAAGTAATTGCTTCTTGTACAGATTGTGTATGAATCGGTTATAACTATAGAATACCTCCGTTTCGTGAATTACGGCATTTATTCGAGTGATCCATAAACGACGAAAATCCCTTTTTCTTTTACCCCTATCCCGACGAGCCGAAACTAAAGCTCTTATTCTCTGTTGAGTCATAGTTCGTGTAAGTCGTGAATGAGCCCCTTGAAAGCTTGATGCAAATAAACGAAGTTTTGTTCTACGCCTCCGAGCTATATATCCGCGTTTAATTCTAGTCATTGAATAAATCAAACTTTGATGAATAACTAATTCTTTTTTTAGTTTTAGTTATTCTTTTCCCCTTTACTAGTCATTAATAACCAAACGAATTATTCCAATGTATAAAAAAAAATTCCAATGGCTTTTGCTACTCTAACCTTCCCCACCACTATTTTTGGCTAGGTATTTTCCTTTGCTTTGAAAGGATAAATTGCCTTGGTACTTGATATAAAAAAAATAGAATAACTACAAAAAGTAGTAAATAGAAATGGATAAATAGTGGGTTCCTTCGTTTCTATGGTTACTTCTAAAACGGTGAGGTCCTCTCTATACACCGGAGCTCCTTCTTTTAATTAATCAATACTATTGGTAACTTGTACAATTCACATTCTTTGGCTCTACCCCATTAATATTCCAGTAATAGATCTTTCACAATGAGATCCACTTTATACAGTAACGGTATTTTATTTTAAAATTGATTTGGTCATTTACCCTGTTAGTCCGTTTTTTCTTTCAAGAGTGGAATCTTTTTAATAAAAAATGGGATTTCCCCCGCTTAATTGATAACCATTTGTTATCATTGGGGGTTTTCTAAAAAATGGAGTTGATTGGATTTGCACCAATGTAAACCATAAGTTTCAGACACAATAGAAGATATGAATGATCTATCTTTTTGAAATAATGAATCGAGTTCCTCCATTCTATTTTATTTTATTAACAGGTACTGATCCTTGATATTTCAAAAAGAATTTCCTTTTTGTGTTTCAGTCTATGATCTAAACGAGTCGCACATACACCCGAGTACATGTTCCTCGTCGCTGAGGGCATCCCCGAAGCGCTGGGGATTTTGTGACATTTCGGATTGGCTGTCTTGTATTTCTAATAAGTTGTTTAATGGTTGGCATACGGAATCATATAAATAATGGGCTGGTTTAGATGGGTTCTAACCGGCTAATTCTGAATTACTTCTCTTCAAGATTCTCTTCAATATATTTTTTTTATATTGAAGAGAATATGAAACTAAACCTTTAATCTAAAAAGATATAAAATTAGCAGTGGTAGATTTGCATGAAATCGCTCCTATTTTTATTGAACCGCTACAAGATCAACAATGCCATGAGCTTGGGCTTCTGTTGCTGACATAAAAACATCCCTTTCCATGTCTTCGGATACAACCCATATAGGTTTGCCCGTTCTTTGTACATAAACCCTTGTGATGGTTTCGCGAAGTTTTAGTAGTTCTTCCGCTTCCAAGATAACTTCTCCCGTTTGTGCCTCATAAAACGAACTAGCGGGTTGATGGATCATTACCCTGATGATATAATAGAAAAGCTTTTTCTATTTCGCAGAATGAGGCGAGATAACCAAAAAAACAGAGAAATTTGAATAACCGTACAGGCTTTTTTTGTGCGTTGCATACGGCTCTAGAATGGAATTTACGTTTTTGACCTTTCCTTTCGGCGAAAGAAAACAAAATATAGGTTCTATTATACGCGGATCCATAAATGATCCAATTACCATCCTTCTTTTTTGTTTTGTAGGAGTTAAAAAAATACTATGATGGTTCCGTTGCTTTATATATCATTTTTTTTGATCCGTCTATGATTCAGCAATCCCAAAGTGTCTTTTTTTTTGTTTTTGTAAATAAGCTTCCGGTGTGAAAACAAAGTTTGTGACGCTGGGATGTGCCCGAATAGGGAAGATATCATTTTAAATACCCCTTTCTTATCCCATACTACTCTTTCAATATATAATCTAATTTTTTTAATCTAAAAAATTTCATATCGAATTCGAAGTGCCATGCTATTATTACTTAACTAATTCATATTTCCGAGGGCGAAGGCATAGTATTTTTTCTCTAAAATAAAAAAACTCATTGGCGCCAAGCGTGAGGGAATGCTATACGTTTGGTAATTTCTCCTCCGACTAGGATAAAGGATGCTATTGAAGCGGCCAATCCCATGCATATTGTCTGTACATCGGGTCGCACAAATTGCATAGTATCATAAATAGCCATTCCAGATATTACCCATCCACCAGGAGAGTTTATAAACAAATAAAGATCTTTGGTATCCTTTTCTATACTGAGATATATCATAAGACTAATAAGTTGATTCGAAATTTCGGTATCAACCTCTTGGCCTAAAAAAAACAATCTTTCTCGATAAAGTCGGTTGATTAGGATAAAATTTTATTCCTTAGGAGCCGTACAGGCACCTTTTGATGCATACGGTTCAACAAAAATTGTTAAAAAATCAATGTGTCGATTCCAACCCCCTTTTTTTCAGAGAAGGCTTTTCTTTCTAACTTAATAAGGGAAGGGCTTGCTTCCCTTTTAAAAGTAAAAGAAAAAATAAATAAGTTTTGGCCCCTTTTATTTATTAGATATTATAATCCTAATAATAAAATAATAAAACGATTGATTAGGCCTGTCAGACTAACTTGATTCATTGATATTTTTTTTTCATCGAGATTCAGTTGAAATGGGGATGGTTTTTTCTTGTTCCTGAATGGGCTTCTTCCTTTTTTTATTCTGTTTTTTTAGGTTTATGCTCTACTCCGAGTAAAAGGAAAAATTTGCCCGATTTTGATTTGCACATATAGGACAAATGAACCAAATACCGCGTCTTTTTTTTTTTTACTACTCCTTCTTTTTTTTTCAATTCATTTCTTTCACATGTCTTCTGTCAAATAGTCAATAAATTTTTAATTATATTATTTTATTTGATCAACAGTTTTAGATCACCCTGTTTCAATTTTTTGTATTTTTTTTATTTTTTAATAGAATTTTTATCATAATTTTGATATCATATTCATATCATATTAAGTAGTAATTATAAAAATATTATATATTAATTATCAATTGGATTTTTGCTAAACGGAGCCTGGATACTTAATTTTATTAGTCCGATCACGTAAACCATAAAAAATTTTTGATAATCTAATATCAATCTAAATACTCCCTGCATTTAATTCTAATTTATTTTTTGCGCTTCGCGTTACAAATTTTTGATAATTCAATCAATCTTTTTGAGCGAAACAGAGGATATCTCGATCGAGGGAGAAAATGGGGAAATCCCATATAGCCCAATATATCTGACAAGTCGCACTATATGTCAACCCAAGATGTATCTCCTTCTCCAGGACTTCGAAAAGGTACTTTTGGAACGCCAATAGGCATGAAATGAAAAAAAAAGAGAATGAAGTTCTCTATTTCACTTTGATGTGGAAACGTAAGACTGGGGTTTCATTTTTTTTATCATATTATCCTTTTTTCCTACTTTATTAATATTAATCATATTTAAATTAATAATATTTAATACAGAAGTTGAATAAGCTAAAATAAAATATAAAATAAAAGTAAAGTAAGAGAGAATGAATAAAAATGAAAGGAAACTTTTTACGAACGGGCTTCTGAACGATGAACAACAATAGCTATCTTGGTTCATATAACATAGGATTCACCCCCATTGCGTATTGGTACTTATCGGATATAGAATAGATCCGCTTCCCTTTTTTCCTATGAATCGAATTGTTCCATTATTACTAACAGAATAGAACAAATATTAATCCTTTCTCCGAAATAATTACCTAAAAAGGGGGGGTCCGTAACATAGTTTTTTCCAATGCAATAAAGTTACATAGTGTCTATTTTTCATTGATAAAGGGGTATTTCCATGGGTTTGCCTTGGTATCGTGTTCATACTGTTGTATTGAATGATCCCGGTCGTTTGCTTTCGGTTCATATAATGCATACTGCTCTGGTTGCTGGTTGGGCCGGTTCCATGGCTCTATATGAATTAGCTGTTTTTGATCCCTCCGACCCTGTTCTTGATCCAATGTGGAGACAAGGTATGTTCGTTATACCTTTCATGACTCGTTTAGGAATAACCAATTCATGGGGCGGTTGGAATATTACAGGAGGGACTATAACGAATCCGGGTCTTTGGAGTTACGAAGGGGTAGCCGCAGCACATATCGTGTTTTCTGGCTTGTGCTTCTTGGCAGCTATTTGGCATTGGGTATATTGGGATCTAGAAATTTTTTGTGATGAACGTACAGGAAAACCTTCTTTGGATTTGCCCAAGATTTTTGGAATTCATTTATTTCTTTCAGGAGTGGCTTGCTTTGGTTTTGGCGCATTTCATGTAACAGGATTATATGGTCCTGGAATATGGGTATCCGACCCTTATGGACTAACCGGAAAGGTCCAACCCGTAAATCCGGCGTGGGGCGTGGAGGGTTTTGACCCTTTTGTTCCGGGAGGAATAGCCTCTCATCATATTGCAGCAGGGACGTTGGGTATATTAGCGGGCTTATTCCATCTTAGTGTTCGTCCGCCTCAACGTCTATACAAAGGATTACGTATGGGAAATATTGAAACCGTCCTTTCCAGTAGTATTGCTGCTGTCTTTTTTGCAGCTTTTATTGTTGCTGGAACTATGTGGTATGGTTCTGCAACTACTCCCATCGAATTATTTGGTCCTACTCGTTATCAATGGGATCAGGGATACTTTCAACAAGAAATATATCGAAGAGTTAGTGCCGGACTAGCTGAAAATCAAAGTGTATCAGAAGCTTGGTCTAAAATTCCTGAAAAATTAGCTTTTTATGATTATATTGGTAATAATCCAGCAAAAGGGGGATTATTCCGAGCGGGTTCAATGGACAATGGGGATGGAATAGCTGTTGGATGGTTAGGACACCCCGTCTTTAGAAATAAAGAAGGGCGTGAACTTTTTGTACGCCGTATGCCTACTTTTTTTGAAACATTTCCGGTTGTTTTGGTAGACGGAGACGGAATTGTTAGAGCCGACGTCCCGTTTAGAAGGGCAGAATCTAAATATAGTGTCGAACAAGTAGGTGTAACTGTTGAGTTTTATGGTGGTGAACTCAATGGAGTAAGTTATAGTGATCCCGCAACTGTGAAAAAATATGCTAGACGGGCTCAATTGGGTGAGATTTTTGAATTAGATCGTGCTACTTTGAAATCCGATGGTGTTTTTCGTAGCAGTCCAAGAGGTTGGTTTACTTTTGGGCATGCTTCGTTTGCTCTACTTTTCTTCTTTGGACACATTTGGCATGGTTCTAGAACCCTCTTCAGAGATGTTTTTGCTGGTATTGATCCAGATTTGGATGCTCAGGTGGAATTTGGGGCATTCCAAAAACTTGGAGATCCAACTACAAAAAGACAAGCAGTCTGATGCAACATTGCTTTTTTCTTTTAGTTTCTGTTTGCGATTTTTTTGATTTCATTTTATTTAATAGGTAGGGTACTGTAGGAATCTTGATTTAAATCGCTGCCGTTTCTTTGACTCTTTTTTGTTCTTTATCCGGAGGTATACTCCTTCAGTAAACATAAACAAAACAGGTATGAAAGCTATAATTGTAAACCACGATCAAATTTATGGAAGCATTGGTTTATACATTTCTCTTAGTATCGACTTTAGGGATCATTTTTTTCGCTATTTTTTTTCGGGAACCGCCTACAATTTCAACTAAAAAATGAAATAATTTTTCATTCTCTTCATTGACGTAATCAGCCTCCAACTATTTGGAGGCTGATTACGTCAACTAGTCCCCGTGTTCCTCGAATGGATCTCTTAGTTGTTGAGAGGGTTGCCCAAAGGCAGTATATAGAGCATACCCAGTAAAACTTACAAGTAACCCAGATATAAAGATGGCGACTAGAGTTGCTGTTTCCATTATTATATAATTGAAAGACCACAATGGATCTATGCTAAGATCGTTTATTTACAACGGAATGGTATACAAAGTCAACAGATCGTAATGAATACAAAATAAGATTTATGGCTACACAAACTGTTGAAGATAGTTCTAGATCTGGTCCAAGAAGCACTACTGTAGGGAAGTTATTGAAACCGTTGAATTCTGAATATGGTAAAGTAGCTCCTGGATGGGGAACGACCCCTTTGATGGGTGTTGCAATGGCACTATTTGCGGTATTCCTATCTATTATTTTGGAGATTTATAATTCCTCTGTTCTACTGGATGGAATTTCAATGAATTAGACTGAGAAGAATCTTGAAGTCCTAGCTTTTTGTTCGATACAAAAAAGTAAAGTATGTAGGTCTAAAATTTTGCACCTATTCTCCTTTGGTAGTTCGACCGCGAAATTTTTTTCTGCATTGTATATTTCCGGAATATGAGTGTGTGACTTGTTAGAATTGACCCTATGGATAGTACAGAGAAGGGGGTCTGTCATCTTTATCAAGATGGTTTTATTTCGTCGGATATTCATTCGAGTATCTGGAGCACGAAATAGATCAAATAGATCACAAAGTTTTCGAACTATGATTCATACTTAATACTTAGACCTCGTAGCCGGACTTCTTTCCGTTCTATCTTATAAATTTTCATAAATCAATTTTTTTCTGCTTTTAAACTCTTATTTAGATCAAAGGACAAACGCTTCTTTGTATTTTATGTTTTTAATCATTATAGCTCTTTTTTTTTTTTATTGAATAAGTGATGATCCAATGGTTCTCACTCAGTGAACTTTGGACTTTGAAGGTTTCATTGAATTATCGTGGTTTTCGTATGAATCTGAGGTTTCAATTAATAAGTAGGGTCTTAACAAGAAAATTCCTATCAATAATAAAGAAAACAAGAAGAAATCCGTATTCCCATTCCATACAAATACCAACTAAAAAAGACAATAACGATAGGTAATCTAGAAGATTCAAGAGGCCTGTAACGATCAACACAACATAAAGACGTATGAGCTGACTTGAGTTTTTGGCATTTAACCACAAAGAAGAGCTTTCGCATTTTGACTCTTAAATAATATTGAATGAGAGAGAAGTTTAAAACTTTATATTCCATATCCGTTTCAATCAGTATTTGGGTCTTTTTTTTGTTTGAGCTGTACGAGATGAAATTCTCATATACAGTTCTTGGAGGGGGAGGAACCTTGGTTTACCTATCTCAATAAAGTTTATGATTGGTTCGAAGAACGTCTTGAGATTCAAGCGATTGCAGACGATATAACTAGTAAATATGTTCCTCCGCATGTCAACATATTTTATTGTCTAGGAGGAATTACCCTTACTTGTTTTTTAGTACAAGTAGCTACGGGATTTGCTATGACTTTTTATTACCGTCCAACTGTTACTGAGGCTTTTGCTTCTGTTCAATATATAATGACTGAAGCTAACTTTGGTTGGTTAATCCGATCAGTTCATCGATGGTCGGCAAGTATGATGGTCCTAATGATGATCCTGCACGTATTTCGTGTATACCTCACCGGTGGTTTTAAAAAACCTCGCGAATTAACTTGGGTTACTGGTGTGGTTCTGGGTGTATTGACCGCATCTTTTGGTGTAACAGGTTATTCTTTACCTTGGGATCAAATTGGCTATTGGGCAGTCAAAATTGTAACAGGTGTACCTGACGCTATTCCGGTAATAGGATCGCCTCTTGTAGAATTATTACGCGGAAGTGCTAGTGTTGGACAATCCACTTTGACTCGTTTTTATAGTTTACACACTTTTGTATTACCTCTTCTTACGGCTGTATTTATGTTAATGCATTTCTTAATGATACGTAAGCAAGGTATTTCTGGTCCCTTATAAATAATATAGATTCTAGATATTTTTAATTACTAATTTATCTTATTACTTGGTGAAGGAACAATCGTATTTTATTGCTATAAATATGGATTATTAAAAAAATAAGACATGTATTTGGATATTTCCCTTCAACTCCACAATATTGTATTATTTTTTTGACATAAAAAGTTGAAGGGAATTCTATGAAGAGAAAATGGATTATGGGAGTGTGTGACTTGAACTATTGATCGGGCCGTGCAGAAATATGACTTTATCTGCTACATTGGAATTCACAACCAAATGTGTCTTTGTTCCAACCACTGTGTAAGCCCCATACAGGGGATAGGCTGGTTCACTTGAAGAGAATCTTTTCTATGATCATAATACCCGACGATGTCGTGGATGAGTGGGCTCCGTAAAATCCAAAAATCCAGGAGATTAAGGGATGGAACATAATCAGGATTATGTTTTTAGCTATTTTTTAGTAAAAAATAGCTAAAAACATAAAAAATTAATAGTATGTAAATGCATTCATTTCCTCTGCATCGACTCGATTTCTGATACTATCGGAGTGAATACAGGATCTAATGAAGAGTAGAGGGTAGACTTCATTAGTAACAAGTAAATCCTTTGTATTTGAAAAATCTCGATATAATTTTTGAGATTAAGGATTAATTGATAAGGTATGAGACGATCCAGAAAGCACTTAATCATGATCAACTTTTAAGCTTACGTGGGTGTTGAGCATTTACCTGTAAGAATGGAATTTATGGTAATCTTTAGTTGCAATAACTTTGGAATCGGATAATTCTTTTTTTACATATTAAATACTTGTTGATAACATATATATTTTTTGTATGTATTAATTTAGTTTGGTTAATTCTTGCTCGAGCCGGATGATGAAAAATTATCATGTCCGGTTCCCTCGGGGGATGGATCCATAAGAATTCACCTATCCCAATAACAAAAAAACCAGATTTGAATGATCCTGTATTACGAGCTAAATTAGCTAAAGGTATGGGTCATAATTATTACGGAGAGCCCGCATGGCCCAATGATCTTTTATATATTTTTCCAGTAGTCATTCTTGGTACCATTGCCTGTAACGTAGGCTTAGCGGTTTTAGAACCATCAATGATTGGTGAACCTGCGGATCCTTTTGCAACTCCTTTGGAAATATTACCTGAATGGTATTTCTTTCCTGTATTTCAAATACTTCGTACAGTGCCTAACAAATTATTGGGTGTTCTTTTAATGGCTTCAGTACCGGCGGGATTATTAACCGTACCCTTTTTGGAAAATGTTAATAAGTTCCAAAATCCATTTCGTCGTCCAGTCGCGACAACCGTCTTTTTGATTGGCACCGTGGTGGCCCTGTGGTTAGGTATTGGAGCAACATTACCAATTGATAAATCTCTAACTTTAGGTCTTTTTTAATTAAATTTATTCAATTGTAAAATAAAAGGCGTGGGTATCTAGGGAGTAGTCATTTCAAAATGAATTCTCCCTAGATACATATCTAAATTAATTTTATTAAGTAAAATAGGTTTGACTGGAAAATCGAAATTACGTTGAAGGTTTAAAATCCATTTCAATTTTAAATTGACTTTTTAGTCAAATTTTTTTTAATGCTTTTTTTATTTTTTTTCTAAAATGTCTAATATCTTTTTTACATCTTCTATGTGAAAATGTTCCATTTTGATAAGGTCTTCTTGACTGTTATTCAAAAGATCCAATAATGTATGTATATTGGACTTTTTGAGACAATTATAGATTCTGGGAGGCAATTCTAATTGGTCAATAAAAATATATTGAAACGCTAGTTCTTTTTTGTTTTTTCTTAGGTTAACTAATCTATTATGAAAAGGAAAAAGGGGTAAAGTAACTTGATGTTGATTGTTCTCTAAATAGAACGTTTCTTCTTCTACATGTAGAAAAGGAATAAATAAATTAATCAAATTCCGGGAGGCTTCATGAAGTGCTTCTTTAGGAGTTAAACTTCCATTTGTCCATATTTCTAGAAAAAGAATCTCTTGTTTTTCATTCCCATTCCCATAAGAATGAATACTATGATTCGCATTTTGAACAGGCATGAATACAGCATCTATAGGATAACTTCGGTCTTCAAAGTTATTTGACATTTTTAAACTATATCCGCGATTCCTCTCGATTTTTAATCCAATACACAAATTTATTGGTTCCGTTAAGGTAGCTATATGCTGTGTATTATCAACGATTTCCACAGAGGGCGGTAAAACTATGTCTCGAGCAGTTATATATCCGGGACCTTGGACACAAATAAGCGCGTTGCGCGTTCCATATAGATTACTTTTTAATACAATCTCGTTCAAATTCATTAAAATTTCATGTACTGATTCTTGAATACCGACTATGTTAGAATAGTCATGTGGTATGTTCTCAGATTTTGCACGTGTAATACATGTTCCTTCTATTTCGCCAAGTAAAGCTCTTCGCATCGCAATGCCTATTGTGTCGGCTTGACCTTTCATAAGTGGAGACAGAATAAAGCGTCCATAATAAAGACGCTTACTGTCTCTTCTTGATTCAACACACTTCCACTGTAGTGTCCGAGTAGATACTTTGACTTTCTCTCGAACCATAGTAATTTTATTTGATCAGATCATTGAATCATTTATTTCTCTTGAAACCCTTTCAGCCTTTATTTAGTTCTATACACGTCGTTTTTTAGGGGGTCTACAACCATTATGTGGCATAGGGGTTACATCTCGTACGAAACTTAAAAGTATACCGCTTCTACGAATAGCTCGTAATGCTGCATCTCTTCCTAGTCCAGGGCCTTTTATCCTTACTTCAGCTCGTTGCATACCTTGATCCACTACTGCTCGAATAGCATTTCCTGCTGCGGTTTGAGCAGCAAAAGGTGTTCCTCTTCTTGTACCCCTGAATCCACAAGTACCCGCGGAGGACCAAGAAATCACCCGACCCCGTACATCTGTAACGGTCACAATGGTATTGTTGAAACTTGCTTGAACATGAATAACTCCCTTTGGTATTCTACGTACATTTTTACGTGAACCACTACGGGTATTTTTACGTGAACCAATTCTTAATATAGGTTTTGCCATATTTTTTCATTTCACAAGAAATATATGGATATATCCATTTCATGTCAAAACGGACCTTTTTTTTACTAGCTCCTTGGAAGTGCCTTTTCCTTTAGTAAGATTATCCTTGTCTTTGTTTATGCCTCGGGTTGGAACAAATTACTATAATTCGTCCCCTCCTACGGATTAGCCGACACTTTTCACAAATTTTACGAACGGAAGCCCTTATTTTCATAGTTGTTATTCCTTAATTCTCTTAATATACTTATTGTTGGACGAAAAAAAGGTTTCTTGATATTTTTGAATCTTGAATTGTATCTTCGTGAAAGGAATGTTGAATTTCAAAAAACCACTGACTTATTTGAATCCTTGTTATGGAGTCTAGAAAGTGGCTGTTCCCCGATTAACTTAATACCTAAGAACTTACTAAAATTTTTACCCCTTTTTCTCCTATAGGTATACCTATACAAAAATATGTCGAATCCTTTCAGAAGCATGACCTAAAATAAAAAAAATCTTTAGTATCTAAACAAAATCGAACCATACCGTTCGGAAGTGATTCATAAAGAAAACTTTCATTAATTCATTTTTTTCTTTAATTTCATTCGGGGTAAAAAATTCTAAACTTTTTTAGCAGGGGTGGTATTACACAACCCCCCCCCTTTTTTTTCACAAATGCTAAGTTCCGGATATCCAATTTTGATATTAGAAGGATTACCATATATAACACAAAATTTCTCCGCCGATTCTTTTTAGTCGAGCTTCTCGATCTGTCATTATACCTTGAGAAGTTGAAAGGATTACAATTCCTATTCCGCCTAAAATTCGTGGAATTCGTTGAGAGTTAGAATAGATTCGTAGACCCGGTCGGCTTATTCTCTTTAAATTTAAAATCGTTTTATAGGATTCTTTCTTATTTCGTCTATGTCTTAGGGTTAAAATCAAAAAATATTGATTGTTTTCGCGATGTTTCCTTACGTTTTCGATAAAACCCTCTTGTAAAAGTATTTTAACAATGCTTTCGGTGATGTTAGTCGATCCTATCCGAACTGTTCCTTTTCTATTCATGTCAGCATTTCGTATAGAGGTTATTATATCAGCAATAGTGTCTTTCCCCATGATAAGTTAAAATTCCTTAATTGTTCTATAATTTTGATATAATCAACATGTTATTTTTCTTTTATTTATATAAAAATAGAGACGAATTATATATTAATATATGAATTCAATTATTAATATATAAAATTATTAAGGGTATATGCGTGATACACAATCTATTAATTATAATTAATTTGATTTCAATACCATTTTTTTAATCCTATCCTATATTAACTATCGATATTTAGGTCTTATAATACTTCAGGAGCTAATGAAACTATTTTAGTAAAGTTTAATTGTCTCAATTCCCGTGGGATCGCCCCAAAAACGCGAGTTCCTTTTGGATTTCCTTCTTGATCAATGACAACTGCGGCATTGTCGTCATATCGTATTATCGTCCCATTCTTACGTTTGAGTTCTTTACAAGTACGTACAATTACAGCTCTGACCACTTCTGATCTTTCTAGAGTAGTATTTGGGATTGCTTCCTTGATTACAGCAACAATAACGTCACCAATATGAGCATAGCGGCGATTACTAGCTCCTATTATTCGAATACACATCAATTTTCTAGCCCCGCTGTTGTCTGCTACATTCAAATAGGTTTGTGGTTGAATCATATTTTTGTATCTCTTCTTTTAGTGCAAAGGACGAAGTAAAAAAAATATTGTTTGTCAAAAAAAAACTTATAATCTTTTTATCCTTAAATGTTATTTAGCTTTTTCATTCTATATTCCTATTCAGAAATAATGAATTGGGTTTTTATAGGCATTTTTGATGCCGCGATTGAAATAGCTTTTCTGGCTATATTTTCTGGTACACCACCCATTTCATAAAGGATTTTACCTGGTTTAACCACAGCTACCCAGTACTCTGGGGATCCTTTCCCAGAACCCATACGCGTTTCCGCGGGTCTTACTGTAACTGGCTTGTCTGGAAATATACGTACCCAAATTTTTCCACCACGTCGTATATTTCGTGTCATTGCTCGTCGCCCTGCTTCTATTTGTCTAGATGTAATCCAAGCGGGTTCAAGTGTTTGAAGAGCATATCTGCCAAAACAAATACGATTCCCACGAGAGGATATTCCTTTTAGTCTTCCTCGATGTTGTTTACGAAATTTGGTTCTTTTTGGGTTATAGTTGATGGGTTTTTTCTAAATGAGAAATTCCATCTCTACTGCAGAACTGGACGTGAGAGTTTCTTCTCATCCAGCTCCTCGCGAATAAAAGGATTAATTAAGATATAGATGTAGTTAATGATTAATCCTATTAATCATGGTATTTTTTTTTTATTTCATCTTATCTCTTCTAAATTTGTGTATGTCTTTTTTGAAATAGAATCAAAGATCAATTTTATTTCGATTTATTTAAAAATAACGTAATATCATCATTACAAATGTAATTTTTATTAGAGTTAGAATATTATAACAAATCCTTATTTTATTTTTTTCATTGTTTTTTTCATCTTTTATTACTGTTTTTATTTGAAAAAAAAACCAATTTTTCGCCGGCGAATATTTACTCTTTCAATATCTATTTAAGTTTGCTGTTTATCCCCCGAGGTCTCAGAATCAAAATCAGAAATCAGAATAGATAATAAAGTTTCTGGTTTATTCCGCCATCCTGTCCAATGAATTACTAAGATTTCTTGTTCACTAGAATCCTATATATTCATGGGTTCCGTCGTTCCCATCGCTTCTTGATTAATCATTAGGCCTGAATTCTACAATGGAGCTTTTACATGAAATTTTGAATTTCTTTTTTTTTTTTTGAGGCAATTTTCTCAGTTTTTATTGGCTCAAGGCTCTTAATTTTTTGTTTTCGGAACAGATTTATCTAATTATTATGAATGAATCTGTATTGATGCTTTATTACATTGCTTTTCTTACAGTGACCTCATAGATTTTCCAAATTGGAATCATATATCATTAATATTCAATTTTTTCGCTCTTTCTTTCATCCTTCCATTTATCCGCATACTTTTTGATTACCTTTCATAACTTAATAATCATCTTTCTTTATTCTTTTTTTTTAGTCAGTTGCTCCAATGATATGATCAGCCTATCATATCTTGACTAATTTTTTGGATCCAGATAATGCGAAGCAATGAGTTGCTTAGGTTATTTATTAATGCTGTAGTTATTAGTTGGTAAGTTCTTTTTTTTTTTATCGTAATCTAACCCTAAACCAACGAGTCACACACTAAGCATAGCAATTATATCAAAGGAGTTTTGATGGAAATGTTTATTCAACCTTATAGAATTGCTCATTTTTTTTTCTTAAACATAAAAAAAAAGACTACAAGTTTTTATTTTTATTTCTTTATAGTGTTATACTACATAGTTTTCGTTTTTTATCATTGGATAAAATGTAAAGACAAATAAAGTTTTTTTATTCTTCGTCTACGAATATCCAAATTTTTATTCCTAAAACCCCATAAATAGTTCGAACTGTATAGGAACAATAATCAATTTTAGCTTCAATTGTTTGTAAAGGAACTCTGCCTTCTCTGATCCATTCAACACGTGCAATTTCTTTTCCGTCGATACGTCCTGCAATTTGTACTTGAATTCCTTTTGTATTCGCCTGTTCAGTTAATTCAATAGCTTTTTTCATTGCTTTTCGAAAAGAAACGCGATTTTTTAATTGGCCAGCTATAAATTCTGCAAGAATATTAGGATGCCCATACGGATTGGAAATTCGGGTAATAGCAATGTTGAGTTTTCTATTGACACAATTAAGTTCTTTTTGAACATTCATCTGTAATTCTTCGATTCTTCGGGGTTTATCTTCAATTAATAATTTAGGAAATCCCATATAGATTATGATCTGAATGAGATCGATTCTTTTTTGAATTTCGATACGTGCAATTCCCTCCATACCAGAGGATATTCTTATATTTTTTTGGACATAATTTTTAATACAGTCTCGTATTTTTTTATCTTCTTCTAAACCTTCAGAATACTTTTTTGGTTGTGCAAACCAAATAGAATGATGACTTTGGGTTGTACCAAGTCTGAAACCAAGTGGATTTATTTTTTGTCCCATGGGCCTCCACTACTATATGTATCGTAACATGTTAGATTTATGTTTTCATTGCTGCATCCAGGTTTTTTTAAATACATTAAATATTCTTCATATTGTTGATATAAGGATATATCTTCCAATACGATAGTTATATGACAAGTGGATCTTTTTATTGGGTAACTCCGTCCTCGTGCCCGAGGTTTTAATTTTTTCACCGTATTTCCTTGATTCACTTCAGCTTTACTAATGACTAAATTGGTTTCTTTGAAACCCTTATTGTGACTAGCATTTGCTGCTGCAGAATAAACTAATTTAAAAATGGGATAACATCCTCGATACGGCATAAGTTCTAATATCATAAGTGCTTCTTCGTAGGAACGTCCACGGATCTGATCAATAACTCTCCGTGCTTTGTGGGCAGACATAGATATATATTGCCCTAAAGCATATACGGAAGTATATGATTTCTTCTTTTTCTTCTTTATCATAAGGTTTACCTCTCACTAAAAAAAAAAATTATATTCATTATTCATTTTTTTGAATTCATTTAATTAACGACGAGATCTATTATCATTTTTCGCGTGTCCTCTAAAATTTATAGTAGGTGAAAATTCTCCCAATTTATGTCCTACCATAAGGTCGATTATATAAACGGGTAAGTGTTCCCTTCCATTATGTATAGCGATAGTATGGCCAATCATTGTGGGTATAATAGTAGATGCCCGGGACCAAGTTATTATGATTTCTTTTTCCGCCTTTGTATTAAGCTTCTCTATTTTTCTTAATAAATGCTTTGCTACAAAAGGATTTTTTTTTAGTGAACGTGTCACAGTTAATTAACTCCTATTTTTTTTAAGACGAAGAAAGAAATTCGATTTTCTCTCCTATTTACTACGGCGACGAAGAATCAAAGTCTCACTATATTTTTTCCTTTTTCTAGTTCTTCTTCCAAGCGCAGGATAACCCCAGGGGGTTACGGGTTTTTTTCTACCAATTGGAGCCCTCCCTTCACCACCTCCATGGGGGTGGTCGACAGGGTTCATAACTACTCCTCTTACTACAGGACGTTTACCTAGCCAACATTTCGATCCGGCTCTACCCAAACTTTTCTGGTTTACCCCAACATTTCCCACTTGTCCGACTGTTGCTGAGCAGTTTTTGGATATCAAACGGACCTCTCCAGAAGGTAATTTTAATGTGGCCGATTTCCCCTCTTTTGCAATCAGTTTCGCTACAGCACCCGCTGCTCTAGCTAATTGTCCACCCTTTCCAAGTGTGATTTCTATATTATGTATGGCCGTGCCTAAGGGCATATCGGTTGAAGTAGATTCTTCTTTTTGATCAATCAAAACCCCTTCCCAAACTGTACAAGCTTCTTCCAAAGCATACGGCTTTCTGAATGTAGATGATGATATCTATACGGATGGATCTTATCTTATATATATCGTAGAATTCTTCTATATATGGTAGAAGTACCACACGAGTGGATATATAGGAATCAAAATCTGCCGAATAACTTATGTTATGATCTTCTACATCCTAGGTCTTCCCGTTCCGTCATCTGGCTTATGTTCTTCATGTAGCATTCAGACCGAATGACTCTATGAAATTACGTCGATACTTCCACATATTATGGGTAACGTAGGAGACATCTCTATTTTTCCCCGGGGGAATCTTTAGAATTACCACTGCTTAGCTTTCAATTCGCCTCTGACCATCAAATGAAATGTGAATAACCCGTCCTCCTCTCTTTGAAACAAGGGGCGCTTATGGTTCTGTCGGTGCTTGAAACAATTTTGTCTTCTCCATATTACTATATCTCTAGAGTCAATAATTTTATATGAGGAACTACTGAACTCAATCACTTGCTGCCGTTACTCTTCAGTTTTCTGTTGAGGTCTATCCTGCAGAGGTACTCAAATTGGATCAGTGATCGATTTCTAGGTTTTGTCGTAAACCTAATTGGTTACTTCCAATTACGTAAATCAAATAGTTCAAACCGCACTCAAAGGTAGGGCATTTCCCATTTTTATAGGAACTTCTGTACCAGAAACAATGGTATCTCCAATTATAGCCCCTCTGGGATGTAAAATATATCTCTTCTCACCATCCCCATAGTGTATGAGACAAATGTATGCATTTCGATTAGGGTCGTATTCTATGGTTACGATTCTACCATATATGTCTTTTGTATTTCGTCGAAAATCTATTTTACGGTATAGACGCTTATGACCTCCCCCTCTATGCCTTACGGTAATGATTCCTCTGGCATTACGACCTTTACCACAATGATGCTGCCCATAGATCAAATTATTTCGTGGATTGGATTTCACTTGACTGTCTACGGCTCCATTGCGTGTGCTCGGGGTAGAAGTTTTGTATAAATGTATCGCCATGCTATTAAGTATTTTGATTTAAGTTCTTTTCTTTCTAAGAGGTGGAATAGAATAACCCGGTTGAAGCGTAATGATCATACGTCTGTAATGCATTGTATGTCCCAGAATAGGTCCCATTCTTTTAACCTTTCCGGGGAGTCGATGACTATTCATAGCTATTACCTTGACACCAAAGAAGAGTTCGACCCAATGCTTTATTTCTGTCCTAGTTGATCCTGATTCGACATTAAAAGTATATTGATTTTTCCCCAATAACCGAATACTTTTGTCTGTAAATACTGCATATTTGATTCCATCCATAAATCGATTTTCTTCCCTATGAGTTCTAGTCTCAATAAGAATGCTAGTTCTTACTGTTCATATGTTATGTTATGATATGAATATACCACACCAATTCGTTATGTATAGATGATGAGAAGATTCCATTGATACAGAGCCAATTCCAATAGACTTATTGGAGGGTCCCATTGGCGTGCATCCAGTAGGAATTGAACCTACGAATTCGCCAATTATGAGTTGGGCGCTTTAACCATTCAGCCATGGATGCTTAGTGGGGATCCTCGTACATGGTGAATAACCAAATTCCAATTGAAATGAAATCTTTAGGATAAATCAATGCAATTTAGGAGGAATCAATGAAAGGACATCAATTCAAATCCTGGATTTTCGAATTGAGAGAAATAGTGAGAGAGATCAAGAATTCTCACTATTTCTTAGATTCATGGACCCAAATCAATTCAGTGGGATCTTTCATTCATATTTTTTTCCACCAAGAACGTTTTAGAAAACTCTTGGACCCTCGAATTTTTAGTATCCTACTTTTGCGCAATTCACAGGGTTCAACAAGCAATCGATATTTCACGATCAAGGGTGTAGTACTATTTGTAGTAGCGGCCCTTCTATATCGTATTAACAATCGAAATATGGTCGAAAGCAAAAATCTCTATTTGAAAGGGCTTCTTCCTATACCTATGAATTCCATTGGACCCAGAAATGATACATCGGAAGAATCTTTTGGGTCTTCCAATATCAATAGGTTGATTGTTTCGCTCCTGTATTTTACAAAAGGAAAAAAGATCTCTGAGAGCTGTTTCCGGGATCCGAAAGAGAGTACTCGGGTTCTCCCAATAACTAAAAAGTGTATCATGCCTGAATCTAACTGGAGTTCGCGGTGGTGGAGGAACTGGATCGGAAAAAAGAGGGATTTTTGTTGTAAGATATCTAATGAAACCGTCGCTGGAATTGATATCTCATTTAAAGAGAAAGATATCAAATATCTGGAGTTTCTTTTTGTATATTATATGGATGATCCGATCCGCAAGGGCCATGATTGGGAATTGTTTGATCGTCTTTCTCCGAATAAGAGGCGAAACATAATCAACTTGAATTCGGGACAGCTATTCGAAATCTTAGTGAAAGACTGGATTTGTTATCTCATGTTTGCTTTTCGTGAAAAAATACCAATTGAAGTGGAGGGTTTCTTCAAACAACAAGGAGCTGGGTCAACTATTCAATCAAATGATATTGAGCATGTTTCCCATCTCTTCTCGAGAAACAAGCGGGCTATTTCTTTGCAAAATTGTGCTCAATTTCATATGTGGCAATTCCACCAAGATCTCTTCGTTAGTTGGGGGAAGAATCCGCACGAATCGGATTTTTTGAGGAAAATATCGAGAGAGAATTGGATTTGGTTAGACAATGTGTGGTTGGTAAACAAGGATAGATTTTTTAGCAAGGTACGAAATGTATCGTCAAATATTCAATATGATTCTACAAGATCTAGTTTCGTTCAAGTAACGGATTCTAGCCAATTGAACGGATCTTCTGATCAATTCATAGATCCTTTCGATTCCATTAGTAATGAGGATTCGGAATATCACTATCACACATTGATCAATCAAAGAGAGATTCAACAACTAAAAGAAAGATCGATTCTTTGGGATCCTTCCTTTATTCAAACGGAAGGAAGAGAGATAGAATCAGACCGATTCCCTAAATACCTTTCTGGATATTCCTCAATGCCCCGGCTATTCACGGAACGTGAAAAGCGAATGAATAATCATCTGCTTCCGGAAGAAAGCGAAGAATTTTTTTGGAATTCTACAAGAGCCATTCGTTCTTTTTTCTCTGACAGATGGTCAGAACTTCATCTGGGTTCGAATCCTACTGAGAGGTCCACTAGGGATCAGAAATTGTTGAAGAAAGAACAAGATGTTTCTTTTGTCCCTTCCAGGCGATCGGAAAATAAAGAAATAGTTAATATATTCAAGATAATTACGTATTTACAAAATACCGTCTCAATTCATCCTATTTCATCAGATCTGGGATGTGATACGGTTCCGAAGGATGAACTGGATATGGACAGTTCCAATAAGATTTCATTCTTGAACAAAAATCCATTTTTTTATTTATTTCATCTATTCCATGAACGGAAGAGGGGGGGATACACGTTACGCCACGATTTTGAGTCAGAAGAGAGATTTCAAGAAATGGCAGATCTATTCACTCTATCAATAACCGAGCCGGATCTGGTGTATCATACGGGATTTGCCTTTTCTATTGATTCCTACGGATTGGATCAAAGACAATTCTTGAAGGAGGTTTTCAACTCCAGGGATGAATTGAAAAAGAAATCTTTATTGGTTCTACCTCCTATTTTTTATGAAGAAAATGAATCTTTTTATCGAAGGATCAGAAAAAATTGGGTCCGGATCTCCTGCGGGAATTTTTTTGAAGATCCAAAACCAAAAAGAGTGGTATTTGCTAGCAACAACATAATGGAGGCAGTCAATCAATATAGATTGATCCGAAATCTGATTCAAATCCAATTCCAATATAGTCCCTATGGGTACATAAGAAATGTATTGAATCGATTCTTTTTAATGAAGAGACCTGATCGCAACTTCGAATATGGAATTCAAAGGGATCTAATAGGAAATGATACTCTGAATCATAGAACTATAATGAAAGATACGATCAACCAACATTTATCGAATTTGAAAAAGAGTCAGAAGAAATGGTTCGATCCTCTTATTTTTCTTTCTCGAACCGAGAGATCCATAAATCGGGATCCTAATGCATATAGATACAAATGGTCCAATGGGAGCAAGAATTTCCAGGAGCATTTGAAACATTTCGTTTCTGAGCGGAAGAGCCGTTTTCAAGTAGTGTTCGATCGATTATGTATTAATCAATATTCGATTGATTGGTCTGAGGTTATTGATAAAAAAGATTTGTCTAAGTCACTTCGTTTCTTTTTGTCCAAGTTACTTCGTTTTTTGTCCAAGTTACTTCTCTTTTTGTCTAATTCACTTCCTTTTTTCTTTGTGAGTTTCGAGAATATCCCCATTCATAGGTCTGAGATCCACATCTATGAATTGAAAGGTCCGAACGATCAACCCTGCAATCAGTTGTTAGAATCAATAGGTCTTCAAATCGTTCATTTTAAAAAATTGAAACCCTTTTTATTGGATGATCATAATACTTCTCAAAAATCGAAATTCTTGATCAATGGAGGAACAATATCACCATTTTTGTTCAATAAGATACCAAAGTGGATGATTGACTCATTCCATACTAGAAAGAATCGCAGGAAATCTTTTGATAACACGGATTCCTATTTCTCAATCGTATCCCACGATCAAGACAATTGGCTGAATCCCGTGAAACCATTTCAGAGAAGTTCATTGATATCTTCTTTTTCTAAAGCAAATCGACTTCGATTCTTGAATAATCCACATCACTTCTGCTTCTATTGTAACAAAAGATTCCCTTTTTATGTGGAAAAGGCCCGTCTCAATAATTCTGATTTTACGTATGGACAATTCCTCACTATCTTGTTCATTCACAACAAAATATTTTCTTCGTGTGGTGGTAAAAAAAAACATGCTTTTTTGGAGAGAGATACTATTTCACCTTCGTCAATCGAGTCACAGGTATCTAACATATTCATATCTAACGATTTTCCACAAAGTGGTGACGAAAGGTATAACTTGTACAAATCTTTCCATTTTCCAATTCGATCCGATCCATTAGTTCGTAGAGCTATTTACTCGATTGCAGACATTTCTGGAACACCTCTAATAGAGGGACAAAGAGTAAATTTGGAAAGAACGTATTGTCAAACTCTTTCAGATATGAATCTATCCGATTCAGAAGAGAAGAGCTTGCATCAGTATCTCAATTTCAATTCAAACGTGGGTTTGATTCACACTCCATGTTCTGAGAAATATTTACAGAGGAAAAAACGGAGTCTTTGCCTAAAAAAATGCGTTGACAAAGGGCAGATGGATAGAACCTTTCAACGAGATAGTGCTTTTTCAACTCTCTCAAAATGGAATCTATTCCAAACATATATGCCATGGTTCTTTACTTCGACAGGGTACAAATATCTAAATTTGATATTTTTAGATATTTTTTCAGACCTATTGCGGATACTAAGTAGCAGTCAAAAATTTGTATCCATTTTTCATGATATTATGTATGGATTAGATATATCATGGCGAATTCTTCAGAAAAAATTGTGTCTTCCACAAAGGAATCTGATAAGTGAGATTTCGAGTAAGTCTTTACATAATCTTCTTCTGTCCGAAGAAATGATTCATCGAAATAATGAGTCATCGTTGATATCGACACATCTGAGATCGCCAAATGTTCGTGAGGTCCTCTATTCAATCCTTTTCCTTCTTCTTGTTGCTGGATATATCGTTCGTACACATCTTCTCTTTGTTTCCCGAGCCTATAGTGAGTTACAGACAGAGTTCGAAAAGATCAAATCTTTGATGATTCCATCATACATGATTGAGTTGCGAAAACTTCTGGATAGGTATCCTACATCTGAACAGAATTCTTTCTGGTTAAAGAATCTTTTTCTAGTTGCTCTGGAACAATTAGGAGATTGTCTAGAAGAAATACGGGGTTCTGGCGGCAACATGCTATGGGGTGGTGATCCCGCTTATGGGGTCAAATCAATACGTTCTAAGAAGACAGATTTGAAAATAAACTTCATCGATATCATCGATCTCATAAGTATCATACCAAATCCCATCAATCGAATCACTTTTTCGAGAAATACGAGACATCTAAGTCATACAAGTAAAGACATCTATTCATTGATAAGAAAAAGAAAAAACGTGAGCGGTGATTGGATTGATGATAAAATAGAATCCTGGGTCGCGAACAGTGATTCGATTGATGATAAAGAAAGAGAATTCTTGGTTCAGTTCTCCACCTTAAGGGCAGAAAAAAGGATTGATCAAATTCTATTGAGTCTGACTCATAGTGATCATTTATCAAAGAATGACTCTGGTTATCAAATGATTGAACAACCGGGAACAATTTACTTACGATACTTAGTTGACATTCATAAAAAGTATCTAATGAATTATGAGTTCAATACATCCTGTTTAGCAGAAAGACGGATATTCCTTGCTCATTATCAGACAATCACTTATTCACAAACTTCGTGTGGGGCTAATAGTTTTCATTTCCCGTCTCATGGAAAACCCTTTTCGCTCCGCTTAGCCCTATCCCCCTCTAGGAGTATTTTAGTGATAGGTTCTATAGGAACCGGACGATCCTATTTGGTCAAATACCTAGCGACAAACTCCTATGTTCCTTTCATTACAGTATGTCTGAACAAGTTCCTGGATAACAAGCCGAAAGGTTTTTTTCTTGATGATATCGATATTGATGATAGTGACGATATTGATGCTAGTAACGATATTGATCGTGAACTTGATACGGAGCTGGAGCTTCTAACTATGATGAATGCGCTAACTATGGATATGATGTCGGAAATAGACCGATTTTATATCACCCTTCAATTCGAATTAGCAAAAGCAATGTCTCCTTGCATAATATGGATTCCAAACATTCATGATCTTGATGTGAATGAGTCGAATTACTTAGCCCTCGGTCTCTTGGTGAACTCTCTCTCCAGGGATTGTGAAAGATGTTCGACTAGAAATAGTCTTGTTATTGCTTCGACTCATATTCCCCAAAAAGTGGATCCCGCTCTAATAGCCCCGAATAAATTAAATACATGCATTAAAATAAGAAGGCTTCTTATTCCACAACAACGAAAGCACTTTTTCACTCTTTCCTATACTAGGGGATTTCACTTGGAAAAGAAAATGTTCCATACTAATGGATTCGAGTCCATAACCATGGGTTCCAGTGCACGAGATCTTGTAGCACTTACCAATGAGGCCTTATCAATTAGTATTACACAGAAGAAATCAATTATAGACACTAATACAATTAGATCTGCTCTTCATAGACAAACTTGGGATTTGCGATCCCAGGTAAGATCGGTTCAGGATCATGGGATCCTTTTCTATCAGATAGGAAGGGTTGTTGCACAAAATGTACTTATAAGTAATTGCCCCATAGATCCTATATCTATCTATATGAAGAAGAAATCATGTAACGAAGGGGATTCTTATTTGTACAAATGGTACTTCGAACTTGGAACGAGCATGAAGAAATTCACGATACTTCTTTATCTTTTGAGTTGTTCTGCCGGATCGGTCGCTCAAGACCTTTGGTCTCTACCCGGACCCGATGAAAAAAATAGGATCACTTCTTATGGATTCATTGAGAATGATTCGGATCTATTTCATGGCCTATTAGAAGTGCAAGGCGCTTTGGTGGGATCCTCACGGACAGAAAAAGATTGCAGTCAGTTTGATAATGATCGAGTGACATTGCTTTTTCGCTCCGAACCAAGGGATCCCTTATATATGATGCAAGATGGATCTTGTTCTATCGTTGATCAGAGATTTCTCTATGAAAAATACGAATCGGAGTTTGAAGAAGGGGAAGGAGAAGCAGTCCTCGACCCGGAACAGATAGAGGAGGATTTATTCAATCACATAGTTTGGGCTCCTAGAATATGGCGCCCTCGGGGCTTTCTATTTGATTGTATCGAAAGGCCTAATGAATTGGGATTTCCCTATTTGGCCGGGTCATTTCGGGGCAAGCGGATCATTTATGATGAAAAGTATGAGCTTCAAGAGAATGATTCGGAGTTCTTGCAGAGCGGAACCATGCAGTACCAGAGACGAGATAGGTCTTCCAAAGAACAAGGCTTTTTTAGAATAAGCCAATTCATTTGGGACCCCGCAGATCCACTCTTTTTCCTATTCAAAGATCAGCCCTTTGTCTCTGTGTTTTCACATCGAGAATTCTTTGCAGATGAAGAGATGTCAAAGGGGCTTCTTACTTCCCAAACAGATCCTCCTACATCTATATATAAACGCTGGTTTATCAAGAATACGCAAGAAAAGCACTTCGAATTGTTGATTCAGCGCCAGAGATGGCTTAGAACCAATAGTTCATTATCTAATGGATTTTTCCGTTCTAATACTCTATCCGAGAGTTATCAGTATTTATCAAATCTGTTCCTATCTAACGGAACGCTAGTGGATCGAATGACAAAGACATTGTTGAAAAAAAGATGGCTTTTTCCGGATGAAATGAAAATAGGATTCATGTAATGTAACAGGAGAAAGGTTTCCCATTACTTAGCCGGAAAGATATGTGTCCATGAAATAGGGATTAAGTGGAACGGAATTGACTGGGTGGTAGAGTTGTAGAAACACCTGTTTCTTCCACTTAGCTCCATGGAACAATATGCTACGACGGAAACATGGAAGAATTGAAATCTTAGATCAAAACACTATGTATGGATGGTACGAACTGCCTAAACAAGAATTCTTGAACAGCGAACAACCAGAGCTATTACTCACTACATCAAAAAAATTTCCATTAATGAAGGATGGAAATCCATTGGAAAATCAAAAATACGCATGTCGGATGAAATTGTTGTTGCTATCTGTTCCAATAACGAATCAACTGAATAACTAAATAAAATAGATAGACCTTTCTCTTCGTCTCAGGTCGATAGATCTTCTCAATTGGAAGATCCCCTATATGGATAATACACATTCCAGTTGACCGAGCCTAATTCTAATTGTTTTGTTCCGAAGTAAAGATATCCACGGAGTGGTTCGCCCTATTCAGATATTCACGACCAAGAAGTACTGGATTCTGTTTAGGATAGGTCCTGAAAGGAGAAGGAAGGCTGGAATGCCGCCAGGCGTCTATTATTGAATTCACCCGACCCGATAGTACCAATTTTGGTAACGTCCATCCAGTGCCAAAGTCACTGAATGGGTAAGTCACCAATCCCTAAAACGGACTATGTACTTTATCTGCTGGGTTACGGGGGCATTTTACCAGAGGTTTAGATTGTATCAATCTACCCTTGTGTGATTCCTGTTGAATCATATACTGCGGGGCGCAGGGCGGACGATTTCAAAGCGGACTCCCCCTCCCCATTCATTAGATAGAGAAGATCGCCAAGATTTCGCGATCCGCTGCCGAACTTATTCCATTTCAATATTATGCCTTGAAGAGGACTCGAACCTCCACGCTTTTTAGCACGAGATTTTGAGTCTCGCGTGTCTACCATTTCACCACCAAGGCATCTTGAAAGTGAATCGTATTCCATAAATATGATATCTATCTAGTACGGTGTATTGAATATATGACAAAGGTGGAGTGTTGAAGTATTTCTATTGATCGGTCATGTCATATAGGCCCGAGTCGGACATCTAATTGCTTAGATTTTAATTATCCTTATCCGGAGGATGCCTTATATATATATTAATATTATATCAAAAAGATGGACAATCAAACCTATTTCTCGATTCAATAGAAGTCCAACCAAAGAGGTGAATAGGGTCCCAAATAACGAGAGATATGTAAAAAGTAGGTCAGATTTCGCCTATTCCTAATCCTAAATGGAATGTAACGACGTAGGGATCCCTATGTAAACATAGTATCTATTTAGATACGCTCGAATGACCCCTTCTCATAATGAGAATGTATATAACCTTATTCCGGTCTGGTCCGGTATGGAATGAACTTATAATCATGGAATCGACTCGATCATCAGATTATAGATTATAAGTTCATAACCTTAGTCCATTCCCATTTTGGGCGGAACCGATCTACTAATTCTTTGATTCCAGTTAGTAAGAGGGATCTTGAACTAAGAAATAGATTCTAGAAGCTAAAAAGGGTATCCTGAGCAATCGCAATAATCGGGTTCATTGATATTCCTGGTATAGTAGATGCTATCACACATACAATCATACTCAATTCGATGGAATTGGTTGATCTTAAAGGGGATATTCTATAATTTCGCACGTGAGGGGTTATTTCTTGGTTTCGTCCAGTCATTAATAACTTGATTATTTTTAGATAATAGTAGATAGAAAGAACGCTCGTAAGGAGTCCTATTGAAACCAAGAAATATAGGCCTGCCCGCCATCCACACCAGAATAAATGGAGTTTTCCAAAAAAACCTGCTAGTGGAGGAAGACCTCCTAGGGATAAGAGACATAGAGCTAAAGAGAGAGCCAAAAAAGGATCTTTTGTGTATAATCCTGCATAATCTCGAATGTTATCAGTTCCGGTACGTAGACCAAATAATATAATGCAAGCAAAAGTTCCTAGATTCATGGAGATATAGAACAGCATATAAGTTATCATGCTCGCATATCCACCATTTGAGTCTCCAACAATTATTCCAATAATTACATATCCGATTTGACCTATGGACGAATATGCAAGCATACGTTTCATGCTTGTTTGAGTAATAGCAATGAGATTCCCCAATATCATGCTAAGAATAGCTAGGATTTCCAGAAGAAGATGCCATTCATTTGATGAGAAATAAAAAGGAATATCGAAAATTCGAGTGGCTAAAGCTGAAGCAGCTACTTTCGAAGTAACAGAAAGAAAAGCAACGACTGGAGTGGGAGAGTCAGAGTCGAAAAGAGGATTCCTCACTTCTTTCTCTCATTCAAAACCGTGCATGAGACTTTCATCTCGCACGGCTCCTAAGTGATAAAAGTAAAGAAGAACTCATCTTCTTTCTTTTTTGATTACTTTCCTCGCGTATGTATAAGATCGAATCCTTTCTAAAACGGATGACTAATCCTTAACTTTTCGAGGAATCCTTCATCAGTGGTTGTGAATGACTGATTTTTCTCAATCGTTTCGACCTTGGTTCCGTAGGAGCACGTCCGAAAGATTGAGAAATGGAACCATCTGATTTGATTCGTTCTCAATAGCCATGAGATGATCATCTTAGGGTGATCCTTTTGTCGACGGATGCTCCTATTACACTCGTAGTCTCTGAAGGATGAGAACCAACTATGTAGCATCTACATCGAGAATTCAAGTCTTTCTTGTATACGTCATTAGTCCGATCCTTTGTAGGAACTACCCGTAATAACAAACTTGCAAAATGGATCCGTTTATCATAAAGAGATTCGTTGTTCCTGACCCTGCTTCACATTAATTGTTATTTGAACAAGTCAAAGTTCTGTCTTGGTCTGCGTGGGGATAGCATTTCTCTTCTGCATGTCCATGGAGTTTTGAAAAATCCAAACATCTCAGAGATAGATAGAGAGGTAGGAATTTCTCAAACGAACCGCACTCCTTCGTATACGTCAGGAGTCCATTGATGAGAAGGGGCTAGGGAAAGCTTGAACCCAATTCCTACAGTGATGAATATAAGCGCAATTGAAATTCCTGGGGAGTTATACATTTGTGTATTGATAAGACCATTCACTATTTCTTGAAGCTCAATCTCTCCCCCGGATGAACCATATAGCCAAGAGAAACCATGAACCAGAATAGAAGAGCTTGCCCCACCCATGAGTAAATATTTCATAGTAGCTTCATTAGATCGTACATCTTTCTTGGTATATCCAGATAATAGGTAGGAGCATAAACTGAAACATTCTGGAGCTACAAAGATAGTTATTAAATCGTTAGCACCACATAAAAACATTCCTCCTAGAGTAGCTGTTAATACGAATAACAGAAACTCTGTTATAGCCATTTCTGTACATTCAATGTACTCTACGGATAGAGGAATACAGAGAGTTGAACATAGTAAAATAAGAAATTGAAAGATTTCGTTGAAATTGTTCGTTTGGAAATTTCCTGAAAAGCTAATCATAGGTTCTTCTCTCCATCGGAACAATAGGGCCGTTATGCTCATTACGAAACTTGTTGACGAGATGAAATATAACCAAGGTATATCTTTTTGATCAGAGGTTGAATCGATCATCAGAAGAAGGATTAGGCCAAAAATTAGGATACATTCTGGGAAAATAAAACTTCCATCGAAGAGAAGCAAATGAAAGGCTTTCATAAAAATTCTCGTAGAATCGAGAATGAAATTTTCATTCTGTACATGCCAGATCATGAATTAGTAACTGCATCCAATCTCCAAAAAAAAACCAATTTTTTTTTTTTTTGAATGGAATATTTACGGAATCCCCATGAATAGGTTAAAACCTTATTCCATGGTATTTACATGAGATTGCTCTTTCTTATTCTTAAGCAAGTCCCCGAGAGGGCTTAGTTGATCCATGATTTATGTTTCGTCTTTTCTTTCCTTTTCGTTTGTTTCGAGAAAGAGATCGATCAATTCCGATTTTTTCTTTTTCTATTGATTCTTTTCGGATCGAGATGTATGGATCCACGGATCTATGTGTCTATATAGATCCTGTTCATGGATTAACGAAAATGTGCAAACGCTCTATTTGCCTCTGCCATTCTATGAGTCTCTTCCTTTTTGCGTATGGCATCGCCACTCCCTTTGGCAGCATCCACTAATTCGGAACTTAATTTGAAAGCCATATTTCGACCCGGACGTTTTCGGGATGCCCCTAATAACCAACGAATGGCAAGTGCTTTTCCTTGCGTGGATCCTATTTCAATGGGAACTTGATGAGTTGATCCGCCTACACGTCTTGCTTTTACTGCTATATCGGGAGTTACTCCACGTATTGCTTGACGTAAAACAGATAGTGGATTTGTTTCTGTCTTTTGTTGAATCTTTTTCAAGGCTCGATAGATAATTTGATAAGCCAATGATTTTTTTCCGTGTTTCAGAATACGGTTAACCAACATGTTAACTAATCGATTACGATAAATTGGATCGGATTTTGCAGTTTTTTCTTCTGCAGTACCTCGACGTGACATGAGCGTGAAAGGGGTTCAAGAATCTGTTTTCTTTTTATAAGGGCTCAAATCTTTTATTTTGGCTTTTTGACCCCATATTGTAGGGTGGATCTCGAAAGATATGAAAGATCTCCCTCCAAACCGTACATACGACTTTCATCGAATACGGCTTTCCACAGAATTCTATATGTATCTATGAAATCGAGTATGGAATTCTGTTTACTCACTTTTAAATTGAGTATCCGTTTCCCTCCTTTTCCTGCTAGGATTGGAAATCCTGTATTTTACATATCCATACGATTGAGTCCTTGGGTTTCCGAAATAGTGTAAAAATAAGTGCTTCGAATCATTGCTATTTGACCCGGACCTGTTCTAAAAAAGTCGAGGCATTTCGAATTGTTTGTTGACACGGACAAAGTCAGGGAAAACCTCTGAAATTATTTCAATATTGAACCTTGGACATATAAGAGTTCCGAATTGAATCTCTTTTGAAAGAAGATCTTTTGTCTCATGGTAGCCTGCTTCAGTCCCCTTACGAAACTTTCGTTATTGGGTTAGCCATACACTTCACATGTTTCTAGCGATTCACATGGCATCATCAAATGATACAAGTCTTGGATAAGAATCTACAACGCACTAGAACGCCCTTGTTGACGATCCTTTACTCCGACAGCATCTAGGGTTCCTCGAACAATGTGATATCTCACACCGGGTAAATCCTTAACCCTTCCCCCTCTTACTAAGACTACAGAATGTTCTTGTAAATTATGGCCAATACCAGGTATATAAGCAGTGATTTCAAATCCCGAGGTTAATCGTACTCTGGCAACTTTACGTAAGGCAGAGTTTGGTTTTTTGGGGGTGATAGTGGAAAAGTTGACAGATAAGTCACCCTTACTGCCACTCTACAGAACCGGACATGAGATTTTCACCTCATACGGCTCCTCGTTCAATTCTTTCGAAGTCATTGGGTCCCTTTCCTCGTTCGCGAATCTCCTCCGTCCCGAAGAGTAACTAGGATAAACTCGGTCACGTTTTCATGTTCCAATTGAACACTTTCTATTTTTGATTATTCTCAAAGGATAAGATTATTCTTTTTACCAAACATCTGCGGGTCCAATCACACGATCTTATAATAAGAACAAGAGATCTTTCTCGATCAATCTCTTTGCCCCTCATTCTTCGAGAATCAGAAAGAGACTTTTTCAAGTTTGAATTTGTTCATTTGTAATCTGGGTTCTTCTACTTCATTTTTATTTACTTATTATTTCTTTATTTTCCCTCTCTTTTCTTTATTTGATTTCTTTTTTGATTTTATTCCCTTCCATCATTCTTAAGTCCCATAGGTTTGATCCTATAGAATCTGACCCATGTTCTCATTGAGCGAAGGGTACGAAATAAATTCAATCATATTTTTTTTTGATCAAAAAAAAATCACTATGTGAAATCTTCGTTTTTTTTTTTCTCTTTCTCTATCGCTTTCCCATAAGTACAGCACTTGTTGAATCGATAGAGAACCTTTTCTTCTGTATCGATATGAATCCATTATGAATCGATATTATTACATTCCAATTCCTTACCAATATCCCTCAAGGAAAATCCCGAATTGGATCCCAAATTGACGGGTTAGTGTGAGCTTATCCATGCGGTTATGCACTCTTCGAATAGGAATTTATTTTCTGAAAGATCCTGGCTTTCGTGCTTTGGCGGGTCTCCGAGATCCTTTCGACGACCTATGTTGTGTTGAAGGGATATCTAGATGATCCGATCGATTGCGTAAAGCCCGCAGTAGCAACGGAACCGGGGAAAGTATACATAAGTATACAGAAAAGACAGTTCTTTTCTATTATATTAGGATTTTCTATTCTATTAGATTAGTGTTAGTTAGTGATCTTGGCGCAGTGAGTCCTTTCTTCTCGGTCCACAGAGACAAAATGTAGGACTGGTGCCAACAGTTAATCACGGAAGAAAGGAGGCTCAGCGGGAAGAGGATTGTACCATAGAAGCAAGGAGGTCAACCTCTTTCCAATAGATAACATGAATTCTGGCAATGCAATGTAGTTGGGCTTTCATGTTGATCCGAATGAATCATCTTTTTCGCGGAGTGAAATCTTTGCCTGCTAGGCAAGATTATAGGATAGCAAGTTACAAATTCTGTTTCGGTAGGACATGTATTTCTATTACTATGAAATTCATAAATGAAATAGTTAATCGTGGGGTTACCATTCTCTCTTTTTTTTTTTATCTCGCACGTGTTCCTAAGAAAAGGGAATTTGTTAATTTTTCGGGGTCTTAAAGGGGCGTGGAAACACATAAGAACTCTTGAATGGAAATGGAAAAGAGATGTAACTCCAGTTCCTTTGGAAATAGGAAGATCTTTGGCGCAAGAATAAAGGATTAATCCGTATCATCTTGACTTGGTTCTGATTTCTCTATTTTTTGAAGTTTAAGAAAAGAATACCGTTTCTCCTACCCGTATCGAATAGAACATGCTGAGTAAAATCTTCTTCATGTAAAACCGGCTTGATTTAGATCGGGAGAATCGTACGGTTTTATGAAACCATGTGCTATGGCTCGAATCCGTAGTCAATCCTATTTCCGATAGGAGTAGTTGACAATTGAATCCAACTTTTTCCATTATTTTCATTTCATACCCGTAATAGTGCGAAAGGAAAGCCCGGCTCCAATCCAAGTTGTTCAAGAATAGTGGCCTTGAGTTTCTCGACCCTTTGACTTAGGATTAGTCAGTTCTATTTCTTGATGGGGGAAGGGATATAACTCAGCGGTAGAGTGTCACCTTGACGTGGTGGAAGTCATCAGTTCGAGCCTGATTATCCCTAAACCCAATGAATGTGAGTTTTTCTATTTTGACTTGCTCCCTCGCTGTGATCGAATAAGAATGGATAAGAGGCTCGTGGGATTGACGTGAGGGGGTAGGGGTAGCTATATTTCTGGGAGCGAACTCCATGCGAATATGAAGCGCATGGATACAAGTTATGACTTGGAATGAAAGACAATTCCGAATCAGCTTTGTCTACGAAGAAGGAAGCTATAAGTAATGCAACTATGAATCTCATGGAGAGTTCGATCCTGGCTCAGGATGAACGCTGGCGGCATGCTTAACACATGCAAGTCGGACGGGAAGTGGTGTTTCCAGTGGCGGACGGGTGAGTAACGCGTAAGAACCTGCCCTTGGGAGGGGAACAACAGCTGGAAACGGCTGCTAATACCCCGTAGGCTGAGGAGCAAAAGGAGGAATCCGCCCGAGGAGGGGCTCGCGTCTGATTAGCTAGTTGGTGAGGCAATAGCTTACCAAGGCGATGATCAGTAGCTGGTCCGAGAGGATGATCAGCCACACTGGGACTGAGACACGGCCCAGACTCCTACGGGAGGCAGCAGTGGGGAATTTTCCGCAATGGGCGAAAGCCTGACGGAGCAATGCCGCGTGGAGGTAGAAGGCCTACGGGTCCTGAACTTCTTTTCCCAGAGAAGAAGCAATGACGGTATCTGGGGAATAAGCATCGGCTAACTCTGTGCCAGCAGCCGCGGTAATACAGAGGATGCAAGCGTTATCCGGAATGATTGGGCGTAAAGCGTCTGTAGGTGGCTTTTTAAGTCCGCCGTCAAATCCCAGGGCTCAACCCTGGACAGGCGGTGGAAACTACCAAGCTTGAGTACGGTAGGGGCAGAGGGAATTTCCGGTGGAGCGGTGAAATGCGTAGAGATCGGAAAGAACACCAACGGCGAAAGCACTCTGCTGGGCCGACACTGACACTGAGAGACGAAAGCTAGGGGAGCGAATGGGATTAGATACCCCAGTAGTCCTAGCCGTAAACGATGGATACTAGGCGCTGTGCGTATCGACCCGTGCAGTGCTGTAGCTAACGCGTTAAGTATCCCGCCTGGGGAGTACGTTCGCAAGAATGAAACTCAAAGGAATTGACGGGGGCCCGCACAAGCGGTGGAGCATGTGGTTTAATTCGATGCAAAGCGAAGAACCTTACCAGGGCTTGACATGCCGCGAATCCTCTTGAAAGAGAGGGGTGCCTTCGGGAACGCGGACACAGGTGGTGCATGGCTGTCGTCAGCTCGTGCCGTAAGGTGTTGGGTTAAGTCCCGCAACGAGCGCAACCCTCGTGTTTAGTTGCCACCGTTGAGTTTGGAACCCTGAACAGACTGCCGGTGATAAGCCGGAGGAAGGTGAGGATGACGTCAAGTCATCATGCCCCTTATGCCCTGGGCGACACACGTGCTACAATGGCCGGGACAAAGGGTCGCGATCCCGCGAGGGTGAGCTAACTCCAAAAACCCGTCCTCAGTTCGGATTGCAGGCTGCAACTCGCCTGCATGAAGCCGGAATCGCTAGTAATCGCCGGTCAGCCATACGGCGGTGAATTCGTTCCCGGGCCTTGTACACACCGCCCGTCACACTATGGGAGCTGGCCATGCCCGAAGTCGTTACCTTAACCGCAAGGAGGGGGGTGCCGAAGGCAGGGCTAGTGACTGGAGTGAAGTCGTAACAAGGTAGCCGTACTGGAAGGTGCGGCTGGATCACCTCCTTTTCAGGGAGAGCTAATGCTTCTTGGGTATTTAGGTTTGACACAGCTTCAAACCCAAAGCCCATGAGCTTATTATCCTAGGTCGGAACAAGTTGATAGGATCCCCTTTTACGCCCCCATGTCCCTCTCGTGTGGCGGCAGGGGGGCGTAAAAAGGAAAGAGAGGGATGGGGTTTCTCTCGCTTTTGGCTTGGCATAGCGGGCCCCCAGCAGGAGGCCCGCACGACGGGCTATTAGCTCAGTGGTAGAGCGCGCCCCTGATAATTGCGTCGTTGTGCCTGGGCTGTGAGGGCTCTCAGCCACATGGATAGTTCAATGTGCTCATCAGCGCCTGACCCTGAGATGTGGATCATCCAAGGCACATTAGCATGGCGTACTCCTCCTGTTCGAACCGGGGTTTGAAACCAAACTTCTCCTCAGGAGGATAGATGGGGCGATTCAGGTGAGATCCAATGTAGATCCAACTTTCTATTCACTCGTGGGATCCGGGCGGTCCGGAGGGGACCACCACGGCTCCTCTCTTCTCGAGAATCCATACATCCCTTATCAGTGTATGGACAGCTATCTCTCGAGCGCAGGTTTAGGTTCGGCCTCAATGGGAAAATAAAATGGAGCACCTAACAACGTATCTTCACAGACCAAGAACTACGAGATCACCCCTTTCATTCTGGGGTGACGGAGGGATCGTACCGTTCGAGCCTTTTTTTCATGCTTTTCCCAGGGGTCTGGAGAAAGCTGCAATCAATAGGATTTTCCTAATCCTCCCTTCCCGAAAGGAAGAACGTGAAATTCTTTTTCCTTTCCGCCTCGAAATGGGAGCAGGTTTGAAAAAGGATCTTAGAGTGTCTAGGGTTAGGCCAGTAGGGTCTCTTAACGCCCTCTTTTTTCTTCTCATCGAAGTTATTTCACAAATACTTCCTATGGTAAGGAAGAGGGGGGGAACAAGCACACTTGGAGAGCGCAGTACAACGGAGAGTTGTATGCTGCGTTCGGGAAGGATGAATCGCTCCCGAAAAGGAATCTATTGATTCTCTCCCAATTGGTTGGACCATAGGTGCGATGATTTACTTCACGGGCGAGGTCTCTGGTTCAAATCCAGGATGGCCCAGCTGCGCCAAGGAAAAGAATATAAGAAGGATCTGACTCCTTCATGCATGCTCCACTTGGCTCGGGGGGATATAGCTCAGTTGGTAGAGCTCCGCTCTTGCAATTGGGTCGTTGCGATTACGGGTTGGGTGTCTAATTGTCCAGGCGGTAATGATAGTATCTTGTACCTGAACCGGTGGCTCACTTTTTCTAAGTAATGGGGAAAAGGACCGAAACATGCCACTGAAAGACTCTACTGAGACAAAGATGGGCTGTCAAGAACGTAGAGGAGGTAGGATGGTCAGTTGGTCAGATCTAGTATGGATCGTACATGGACGGTAGTTGGAGTCGGCGGCTCTCCTAGGGTTCCCTCGTCTGGGATTGATCCCTGGGGAAGAGGATCAAGTTGGCCCTTGCGAACAGCTTGATGCACTATCTCCCTTCAACCCTTTGAGCGAAATGCGGCAAAAGGAAGGAAAATCCATGGACCGACCCCATCGTCTCCACCCCGTAGGAACTACGAGATCACCCCAAGGACGCCTTCGGTATCCAGGGGTCGCGGACCGACCATAGAACCCTGTTCAATAAGTGGAATGCATTAGCTGTCCGCTCGCAGGTTGGGCAGTAAGGGTCGGAGAAGGGCAATCACTCATTCTTAAAACCAGCATTCGAAAGAGTTGGGGCGGAAAAGGGGGGAAAGCTCTCCGTTCCTGGTTCTCCTGTAGCTGGATCCTCTCGAACCACAAGAATCCTGAGTTGGAATGGGATTCCAACTCATCACCTTTTGAGATTTTGAGAAGAGTTGCTCTTTGGAGAGCACAGTACGATGAAAGTTGTAAGCTGTGTTCGGGGGGGAGTTCTTGTCTATCGTTGGCCTCTATGGTAGAATCAGTCAGGGGCCTGATAGGCGGTGGTTTACCCTGTGGCGGATGTCAGCGGTTCGAGTCCGCTTATCTCCAACTCGTGAACTTAGCCGATACAAAGCTATATGATAGCACCCAATTTTTCCGATTCGGCAGTTCGATCTATTATTTTTCATTCATGGACGTTGATAAGATCTTTCCATTTAGCAGCACCTTAGGATGGCATAGCCTTAAAGTTAAGAGCGAGGTTCAAACGAGGAAAGGCTTACGGTGGATACCTAGGCACCCAGAGACGAGGAAGGGCGTAGTAAGCGACGAAATGCTTCGGGGAGTTGAAAATAAGCGTAGATCCGGAGATTCCCGAATAGGTTAACCTTTTGAACTGCTGCTGAATCCATGGGCAGGCAAGAGACAACCTGGCGAACTGAAACATCTTAGTAGCCAGAGGAAAAGAAAGCAAAAGCGATTCCCGTAGTAGCGGCGAGCGAAATGGGAGCAGCCTAAACCGTGAAAACGGGGTTGTGGGAGAGCAATAAAAGCGTCGTGCTGCTAGGCGAAGCGGTGGAGTGCCGCACCCTAGATGGCGAGAGTCCAGTAGCCGAAAGCATCACTAGCTTATGCTCTGACCCGAGTAGCATGGGGCACGTGGAATCCCGTGTGAATCAGCAAGGACCACCTTGCAAGGCTAAATACTCCTGGGTGACCGATAGCGAAGTAGTACCGTGAGGGAAGGGTGAAAAGAACCCCCATCGGGGAGTGAAATAGAACATGAAACCGTAAGCTCCCAAGCAGTGGGAGGAGCCCTGGGCTCTGACCGCGTGCCTGTTGAAGAATGAGCCGGCGACTCATAGGCAGTGGCTTGGTTAAGGGAACCCACCGGAGCCGTAGCGAAAGCGAGTCTTCATAGGGCAATTGTCACTGCTTATGGACCCGAACCTGGGTGATCTATCTATGACCAGGATGAAGCTTGGGTGAAACTAAGTGGAGGTCCGAACCGACTGATGTTGAAAAATCAGCGGATGAGTTGTGGTTAGGGGTGAAATGCCACTCGAACCCAGAGCTAGCTGGTTCTCCCCGAAATGCGTTGAGGCGCAGCAGTTGACTGGACATCTAGGGGTAAAGCACTGTTTCGGTGCGGGCCGCGAGAGCGGTACCAAATCGAGGCAAACTCTGAATACTAGATATGACCTCAAAATAACTGGGGTCAAGGTCGGCCAGTGAGACGGTGGGGGATAAGCTTCATCGTCGAGAGGGAAACAGCCCGGATCACCAGCTAAGGCCCCTAAATGACCGCTCAGTGATAAAGGAGGTAGGGGTGCAGAGACAGCCAGGAGGTTTGCCTAGAAGCAGCCACCCTTGAAAGAGTGCGTAATAGCTCACTGATCGAGCGCTCTTGCGCCGAAGATGAACGGGGCTAAGCGATCTGCCGAAGCTGTGGGATGTCAAAATGCATCGGTAGGGGAGCGTTCCGCCTTAGGGGGAAGCAACCGCGCGAGCGGCGGTGGACGAAGCGGAAGCGAGAATGTCGGCTTGAGTAACGCAAACATTGGTGAGAATCCAATGCCCCGAAAACCCAAGGGTTCCTCCGCAAGGTTCGTCCACGGAGGGTGAGTCAGGGCCTAAGATCAGGCCGAAAGGCGTAGTCGATGGACAACAGGTGAATATTCCTGTACTACCCCTTGTTGGTCCCGAGGGACGGAGGAGGCTAGGTTAGCCGAAAGATGGTTATCGGTTCAAGAACGCAAGGTGTCCCTGTTTTTTCAGGGTAAGAAGGGGTAGAGAAAATGCCCCGAGCCAATGTTCGAGTACCAGGCGCTACGGCGCTGAAGTAACCCATGCTATACTCCCAGGAAAAGCTCGAACGACCTTCAACAAAAGGGTACCTGTACCCGAAACCGACACAGGTGGGTAGGTAGAGAATACCTAGGGGCGCGAGACAACTCTCTCTAAGGAACTCGGCAAAATAGCCCCGTAACTTCGGGAGAAGGGGTGCCTCCTCACAAAGGGGGTCGCAGTGACCAGGCCCGGGCGACTGTTTACCAAAAACACAGGTCTCCGCAAAGTCGTAAGACCATGTATGGGGGCTGACGCCTGCCCAGTGCCGGAAGGTCAAGGAAGTTGGTGACCTGATGACAGGGGAGCCGGCGACCGAAGCCCCGGTGAACGGCGGCCGTAACTATAACGGTCCTAAGGTAGCGAAATTCCTTGTCGGGTAAGTTCCGACCCGCACGAAAGGCGTAACGATCTGGGCACTGTCTCGGAGAGAGGCTCGGTGAAATAGACATGTCTGTGAAGATGCGGACTACCTGCACCTGGACAGAAAGACCCTATGAAGCTTCACTGTTCCCTGGGATTGGCTTTGGGCTTTTCCTGCGCAGCTTAGGTGGAAGGCGAAGAAGGCCTCCTTCCGGGGGGGCCCGAGCCATCAGTGAGATACCACTCTGGAAGAGCTAGAATTCTAACCTTGTGTCAGGACCTACGGGCCAAGGGACAGTCTCAGGTAGACAGTTTCTATGGGGCGTAGGCCTCCCAAAAGGTAACGGAGGCGTGCAAAGGTTTCCTCGGGCCGGACGGAGATTGGCCCTCGAGTGCAAAGGCAGAAGGGAGCTTGACTGCAAGACCCACCCGTCGAGCAGGGACGAAAGTCGGCCTTAGTGATCCGACGGTGCCGAGTGGAAGGGCCGTCGCTCAACGGATAAAAGTTACTCTAGGGATAACAGGCTGATCTTCCCCAAGAGCTCACATCGACGGGAAGGTTTGGCACCTCGATGTCGGCTCTTCGCCACCTGGGGCTGTAGTATGTTCCAAGGGTTGGGCTGTTCGCCCATTAAAGCGGTACGTGAGCTGGGTTCAGAACGTCGTGAGACAGTTCGGTCCATATCCGGTGTGGGCGTTAGAGCATTGAGAGGACCTTTCCCTAGTACGAGAGGACCGGGAAGGACGCACCTCTGGTGTACCAGTTATCGTGCCCACGGTAAACGCTGGGTAGCCAAGTGCGGAGCGGATAACTGCTGAAAGCATCTAAGTAGTAAGCCCACCCCAAGATGAGTGCTCTCCTATTCCGACTTCCCCAGAGCCTCCGGTAGCACAGCCGAGACAGCAACGGGTTCTCCGCCCCTGCGGGGATGGAGTGACAGAAGTTTTGAGAATTCAAGAGAAGGTCACGGCGAGACGAGCCGTTTATCATTACGATAGGTGTCAAGTGGAAGTGCAGTGATGTATGCAGCTGAGGCATCCTAACAGACCGGTAGACTTGAACCTTGTTCCTACATGACCTGATCAATTCGATCAGGCACTCGCCATCTATTTTCATAGTTCAACTCTTTGACAACACGAAAAAACCATTGTTCAACTCTTTGACAACATGAAAAAACCAAAAATTCTGCCCTTCTATCCAAAGGATGGAGGGGCGGAGGCCTTTGGTGTCCACTCCAGTCAAGAATTGGAGCCTCACAATCACTAGCCAATATGCTTTTCTCGCATGCCTTTCTTCGTTCATGGTTCGATATTCTGGTGTCCTAGGCGTAGAGGAACAACACCAATCCATCCCGAACTTGGTGGTTAAACTCTACTGCGGTGACGATACTGTAGGGGAGGTCCTGCGGAAAAATAGCTCGACGCCAGGATGATGAAAAGCTTAACACCTCTCATTCTTATTACTTTTTCATATTGAAAAAAAATGCAAAATGAAAAGGTTGTCTTATTCAAAACCCCAATTATGAAATCCCTTCTATCCCACTTCACACCCCGGAACGCACCGTTCTTATAGAGAGAAAGGCACTTTCACATCTTCTTAACCCGAAATGGCTGGGGAGAGGAAAGGTTCCTTTTTTTGTAGGGTACTCCTGGGAACAGATCCAGTGGAGACGGGGTGGGGCTTGTAGCTCAGAGGATTAGAGCACGTGGCTACGAACCACGGTGTCGGGGGTTCGAATCCCTCCTCGCCCACAACCGGCCCAAAAGGGAAGGACCTTTCCCTCCGGGGGGTAGGAAAATCATGCTCGGGATAGCGGACTCAAAGCTATGGAACTTGGTTGGGGATGGGTCTTTTGTCGAAATAGAGTGGAGTGGCCTTCTTTTTTATTTGAATTTAGATATATATATCTATTATATCTATCGCTTTTTTTTTTACATATAGTATGATTATCGGCCGAATCAGCATATTTTTCGAAGCCCCGTAACTCTTCCTCAGCCAGGCTTGGGCAGAATAGCAGAGCAAGTACAAGTATTAGTAGCATAGAAAAAATGCGTTCCTCATCATTAAGTCATTAATATGTTTGCGCGCGGTAATTGTGAACTCTCGGGAGAATCGATGACTGCATCAAAGATGCACTTGTTAGTACACCTGCAAATTCTGAATTGGCTAGTTGTAAATAGCCCCAGGACTATGGAATAAAGGATTATCCCGGACCTACACCGAGGTATTGACGGTGATTCTCAAATATCACAGAACAGAATGTGATACGATGAGATAGAATGCAATAGAAACAAAGACACAGGGAACGGGTTACCTACTCTTACATTCTGAATTCTTGAATTCGGAATGAATCAAATCTCCCCAAGTAGGATTCGAACCTACGACCAATCAGTTAACAGCCGACCGCTCTACCACTGAGCTACTGAGGAACAACGGGAGATTAGATCTCCTAGAGTTCAATTCCCGTTCTCAACCCATGACCAATATGAACTCGAAGTTTCCTTCGTAACCCCCGGAACTTCTTCGTAGTGGCTCCGTTCCATGCCTCATTTCATAGGGAACCTCAAAGCGGCTCTATTTCATTATATTCCATCCATATCCCAATTCCATTCATTTAATATCCCTTTGGTGTCATTGACATAAGAGATGTCGTTTCTAGTCTATCTCTTTCTATTTCTATATATGGAAAGTTGCAAAATCATCATATAATAATCCAGAAATTGAAATAGAAAAGAAAAAAGGGAGGTTTGTGATGGTTTTTCAATCTTTTATACTAGGTAATCTAGTATCCTTATGCATGAAGATAATCAATTCGGTCGTTGTGGTCGGACTCTATTATGGATTTCTGACCACATTCTCCATAGGGCCCTCTTATCTCTTCCTTCTCCGAGCTCGGGTTATGGACGAAGGAGAAGAAGGAACCGAGAAGAAAGTATCAGCAACAACTGGTTTTATTGCGGGACAGCTCATGATGTTCATATCGATCTATTATGCGCCTCTGCATTTAGCATTGGGTAGACCTCATACAATAACTGTCCTAGCTCTACCGTATCTTTTGTTTCATTTCTTCTGGAACAATCACAAACACTTTTTTGATTATGGATCTACTACCAGAAATGAAATGCGTAATCTTCGCATTCAATGTGTATTCCTGAATAATTTCATTTTTCAATTATTCAACCATTTCATTTTACCAAGTTCAATGTTAGCCAGATTAGTCAACATTTATATGTTTCGATGCAACAACAAGATGTTATTTGTAACAAGTAGTTTTGTTGGTTGGTTAATTGGTCACATTTTATTCATGAAATGGGTTGGATTGGTATTAGTCTGGATACAGCAAAATAATTCTATTAGGTCTAATGTACTTATTAGATCTAATAAGTATAAGTTCCTTGTGTCAGAATTGAGAAATTCTATGACTCGAATCTTTAGTATTATCTTATTTATTACCTGTGTCTACTATTTAGGCAGAATACCATCACCCATTTTTACTAAGAAACTAAAAGGAACCTCAGAAACGGGTGGGACTAAACAGGACCAAGAGGTATCCACCGAAGAAGCTCCTTTTCCTTCTCTTTTTTCGGAAGAAAGGGAGGATCTGGACAAACTCGATGAAATGGAAGAAATCGGAGTGAATGGAAAAGACAAAATTAATAAGGATGATGAATTCCACGTTCGAACATACTATAACTATAAAACAGTTTCTGAAAATCGAGATGGAAATAAAGAAAATTCTAATTTAGAATTTTTCAAAATAAAAAAAAAAGAGGATCGTTAAAAAAATCAATACATAGCACAAATACAAGAACAGATAAGAAGAGATGCGACTTCCACCTATATATTTTGTTACTTCTCCTACAAAGAAACTTGTAATACCTACTCCATTTGTAATTCCATCAATGATTCGTTTATCAAAAAAATTCGTTTGTTTTGCTAATTTTCTTATACTTTCAGTTAAAGATTTTTTAAAAAAAGTATCTATGTAACCACGATTATATGACCAATTATATACAAAATTGATTGGTTTTTCCCACCTAATTCTTTTAGAACTCCACTTTTGAAATGAATTAAGTAAAGTTAAATTTAATCTAGATGAATAAAAAGGCTTATATAAACAGTATGCTATAAATATTCCAAACAAAGCTATACTGACTGAAAAAATTGCATTTTTCAAAAATTCATACCAATCTACAAAATTTTCTGAATTGGTATGCAAAAGGTTTATCGACGGCGTTAATAATTTTGATAATATATCAAAGTCTATTCCTTCTTGATTGAAAGGAATTCCTATGGCTCCAATAAACAAAGTAAATAAAAGCAATACAAGCATAGGAAATAGAATAGTATTGTCTGATTCATGGGGATAATAGAAAGTTCTTGTATTAAGTCCAAAATTTTCAACAGTAATAAAAGTTTGATTTCTTACATTATTACTAATTTTATATGTTTTATTGCCAAAAAAAGAAGCTCTTTTCGTATTATTCATTGTTAATAATGGTACTAACCCAAAATTCCTATTAAGTTTTTTATCTTCTTCTTTACCCCATAAAGAAATTGAATAGAAGGAGCGACTTTTTTTTCCACTATAATTTATAAAATAAGTGTTTAAATGGCCTTCAAAAGTAAGTAAATAAATCCGAAACATATAAAATGCGGTTAATCCCGCTGTTGAACAAGCTATTATTGCAAAAATTGGCGAAAATAACAAACTATCATTAAGAATTTCATCTTTAGACCAAAAACAAGCAAGGGGGGGAATACCACAAAGTGAGAGTGTTCCTACTAAAAAGGCAGTTTTTGTAATCGGCACATGTTTTGTCAAACCACCCATAAGAATCATATTCTGACTTTTATCAGGAGAATAGCCAACTATAGCTTCCATTGAATGAATAATGGATCCAGATCCTAAAAACAACAAAGCTTTCGAATAAGCATGAGTAATCAAATGAAATAAAGCGGATCTATAAGATCCCATACCTAGAGCTAACATCATATAACCCAGTTGAGACATTGTAGAATAGGCTAAACCTCTCTTAATATCTTTTTGAGCAAGAGCTAAAGTGGCTCCTAAGAGTACTGTTATTATACCTATCAAAGATATTATATACATTATAGAAGGGATAACTATAAAAAGAGGAAGAAGACGAGCTACAAGAAAAATTCCCGCTGCTACCATAGTAGCAGCATGTATAAGAGCCGAAATAGGAGTAGGGCCCTCCATGGCATCCGGCAACCATACATGAAGAGGAAATTGTGCAGATTTAGCAATAGGACCCACAAATAATAGAAATGCACACAAAGTAAGGAATAAGAGATTTATTCTATTATTTAATATTAAATTATTGAATATTTCGAACAAATCTTGAAATTCGAAACTGCCAGCTATCCAATAAAGACCTAAAATTCCTAATAATAAACCAAAATCCCCTACACGATTGGTTACAAAAGCTTTTTGACAGGCATTCGCTGCAATAGGTCGTGTGAACCAAAAACCTATTAATAAATACGAACACATTCCAACTAATTCCCAAAAAAAATAAACTTGGATCAAATTAGAACTAGTAACTAATCCTAACATTGAAGTATTAAAAAAACCCATATAAGCAAAAAACCTCAGATATCCTTGATCATGAGACATATAATTATCACTATAAATCAGAACCAAAATTCCAACAGTTGTAATTAATATTGACATAATAGAAGTAAGTGGATCAATAAAGTAACCGAACTCAAAAGAAAATTCATTATTTATGGTCCAAGACCATACATTTTGATGAATGCAACTTAGAAAAATTTGTTGAATAGATAGATAGAGCGAAAAGATCATAACTATACTTAACAAAAAAATACTCAGAAACGTCCACATACGTCGAAGGTTTTTTGTTGCTGTCGGAAAAAGTAGAAGTCCAGCTCCGAGTAAAATAGGTACTGGAAGTGGAATGAAAGGGATGATCCATGAATATTGATATGTATGTTCCATAAAATAAAAAACCCTTTTTATTTTATTCTTAAATTTATTATTTCTTATTCACTGGTTTGTATATATATATATATTTTTTCAAAGGGGATAATAAAAAAGCGCATTTTTTCAAACTTAAATAGAAATTTTTTCGAATTAGTATAATCCTTCATAAACCTTTGAAAAGAAATATATTCAAATCAAAAAATTAGAAGTTATTAACTAATATTACTAAGTTACTGTAAAAAAAACGATTTGTCTTTTTTTTTTTACTACTAAAAAAAATTTTGATTTTATGCAGATACAGAAAAAGTGAATTCTAATTCCGTATTACAATAATTTATATACATATATTAAGAATAGAACAAAGATTTACACGACAAAAAAATACTTAATATTAAGTATAAAAAAAAGTTATTTGGGTTTGATTATTTAGAATTATTATTGAATTATGGAATTTAGTGATTGTCTTCCAGTACACTAAGTGAGCTTTTTTTTCAAGAAATATTATTATAATCGATATTTTTTTTTACATATGAAGTGAAGAAAGTTAATAAAATTTTTTTCTAATATAATCTAAATCTATCAGTATAGATTAATTAAATGAGCACTCTCATACGGATTTAAAACGTTAAATACAAAAAATTTTTCAAGAAAAAGGGAAAAAATAGTTGGGTTTTAAACTTTTGAATGTCTGTTTTGTTTGAAAAATAATATATAATAAAATTTGAAAGAAAAAATTTACTCAATATGGAGTACGAAAGAATAGAATAATAAATGTCTTTGACATCCAATTATACCACTGAAAAACTTTTTTCATTTTTGAATGGCAGTTCCAAAAAAACGTACTTCTATCTCGAAAAAGCGTATTCGTAAAAAAATTTGGAAAAGGAAGGGATATTGGACATCGTTGAAAGCTTTTTCCTTAGGGAAATCGCTTTCTACAGGTAATTCAAAAAGTTTTTTTGTACAACAAAATAAATAAAAAACACTAGAATCATTAGAATTAGCCTAACGTAAAAACCAATTTTGTAGAATACATATAAATTAAAAAAATCTATAGGAACCAAAATAAAAAAAAAAAAGATAATATATATACGAAATATACGAAAGATTCCTATTGATTTTGTAAAAAAAAGGGGGGGTTATTACTTTCCCCATCAATAAAAAAAATAAATAAAGATCTTGTATTTCCTCTTAACTAGGAAATACAAGATCTTTTAGCGAAATCAACAGGTTCTTTAAATTAATTTAAGTCAAATAAGTCAAAAATTTTTCACTTTATACCTTTAGGAATTATTATTTCTCTTAATTCTTATATTCTTTACTTGGAATCAAAGTTATAAAAGTATCTATCCACAATTAAGTGAATATTAGATACTAATAAGTAATAATATATGATATTTTTTTTAAGCGATCAAAAAATATTATGTTTGTACAATATAAAAAGATGCATGAAAATAGATATTTTGACAATTGTTGTTTTTCATTTTTCTTGAGCAACTTAGGCAAATTTTAGTTAAAATTTCTAAGGATTTTGGAGAAGTTTTAATTTTTAGAAAAAGCATTTTTTTAGTAATAAAATCAATTTTAAATTCCATTAAATTAGCTTCTTTATTTAAAATTTGAATCTCGACGATTGAGTAAAAACTTGTTAGTATTATTTTGAACAAGTTGCCGCTATGGTGAAATTGGTAGACACGCTGCTCTTAGGAAGCAGTGCTAGAGCATCTCGGTTCGAGTCCGAGTAGCGGCATAAGATCTTATAAAAGAGATATTATAAGTTTTATAATCAAATTAATACCCGACTTGTTTTCTAAAATCGGGTAAAACCTAGTATTAATTTATTAATTTTTAACAAATTTTTTATGATTTTTTCAATTTTAGAGCATATATTAACTCATATATCTTTTTCGGTCGTTTCAATTGTACTACTAATTTATTTTTTAACTTTATTAGTTAATTTAGATGAAATCATAGGATTTTTTGATTCATCAGATAAAGGAATCGTAATTACGTTTTTTGGTATAACAGGATTATTATTTACGCGTTGGATTTATTCAGGACATTTTCCATTAAGCAATTTATATGAATCATTAATTTTTCTTTCATGGGCTTTTGCAATTATTCATATAGTTTCCTATTTTAATAAAAATAAAAAAAATCACTTAAACGCAATAACTGCGCCAAGTGCTATTTTTATTCAGGGTTTTGCTACTTCAGGTCTTTTAAACAACATGCCTCAGTCTGCAATATTAGTACCAGCTCTCCAGTCCCAGTGGTTAATGATGCACGTAAGTATGATGATATTAGGCTATGGCGCTCTGTTATGCGGATCATTATTATCAATAGCTCTTCTAGTCATTACATTTCGCAAGGTCGGATCTACTTTTTGGAAAAATAATATGAAAAATAAAATGTTATTAAATGAATTATTTTCTTTTGATGTACTTTACTACATAAATGAAAGAAATTCTATTTTAATACAACAAAATATTAATTTTAGTTTTTCTAGAAATTATTATAGATATCAATTGATTGAACAATTAGATTATTGGAGTTTTCGTATTATTAGTCTCGGATTTATCTTTTTAACCGTCGGCATTCTTTCAGGAGCTGTATGGGCTAATGAAACATGGGGTTCATATTGGAATTGGGATCCGAAAGAAACCTGGGCATTTATTACTTGGACCATCTTCGCAATTTATTTACATATTAAAACAAATAGGAATGCTCGAGGTATAAATTCTGCAATTGTGGCTTCGCTAGGTTTTCTTTTAATTTGGATATGCTATTTTGGCGTCAATCTTTTAGGAATAGGTTTACATAGTTATGGTTCATTTACATCGAATTAACTAAAACATTAACAAAAAAAGAAAAGAATCCAAATAAAAAAAATAGCATCTATATATAACTTCATATAAGTTAAGAAATCTAATTTAGTTTTAGTAGTAAATCATCAAGAACCTTTTGAATCAAGTAGTACAATGATTCAAAAGGTTCTCACAATACAAAAAGCAAAGACTTCTTATTATAATTCAATTTAATGTTTTTTTTTATTTCCTGAAAACTATCCATAAAAATAATTAGATAAAATGGATTCGACCTTGTCACTTGCTAATGAGAGCACAAAATCAGGATAAATCCCAATACCAATTATGGGTAGAAGAATAGAGATTGAAAGAAATAACTCTCGGGGTCCAGAATCAAAAAAAGAAAAGTTTTTGGCATTAATTAACTTGTATCCATAGAACATTTGACGTGACATAGATAATAAATATATAGGAGTTAATATCATTCCAATTGCCATTACAAAAATAATTAAAATTTTTGAAATTAAGAAATATTTTTGGCTGGTAATTATTCCAAAAAAAACGATTAATTCTGCAACAAAACCACTCATGCCCGGTAATGCAAGGGAAGCCATCGATAAGATAGTGAACATTGTAAATATCTTTGGAATGGAGATAGCCATTCCACCCATTTCATCAAGATAAACAAGCCGGATTCTATCATAACTAGTTCCTGCCAAGAAAAAAAGTGCAGCGCCAATAAATCCATGAGAGATTATTTGTAAAATAGCTCCATTAAGCCCAGGATCCGTTATAGAACCAATACCTATAATTATAAAACCCATATGAGATACAGAAGAATAGGCTATTCTCTTTTTTAAATTACGTTGACCGGGAGATGTTGAAGCTGCATAAATTATTTGGATTGTACCGACTACCATCAACCAAGGAGAAAACATAGAATGAGCGTGAGGTAATAATTCCATATTGATTCGAACCAATCCATATGCTCCCATTTTTAATAAGATTCCAGCGAGAAGCATACAGGTACTGTAATGTGCCTCGCCGTGGGTGTCAGGTAACCAAGTATGTAAAGGTATAATCGGTGATTTGACGGCAAAAGCAATAAGAAATCCAATATAAAAGAGTATTTCGAGTGTGACCGGATAGGCTTGATTCCCTAATAGTTCTAAATTTAATGTTGGTTCGTTCGAACCATATAAACTTATACCTAAAACTCCTATTAATAAAAAAATAGAACTTCCTGCAGTGTATAAAATAAATTTTGTAGCTGAATACAAACGTTTCTTTCCACCCCACATGGATAAAAGGAGATAAACGGGAATTAATTCTAATTCCCACATGATGAAAAAAAGTAAAATATCCCGAGAAGAAAACGATCCTATTTGGCCGCTGTACATTGCTAACATCAGGAAATAGAATAATCGGGAATCCCGAGTAACTGGAAAAGCCGCTAAAGTAGCTAAAGTAGTAATAAATCCGGTCAGTAAAATCGTTCCTATAGAAAGTCCATCTATTCCCAGTCTCCAATAAAAATCAAAAAGATTGATCCATTTATAATCTTCGGACAGTTGAATTAATGGATCGTCCAGTTTAAAATTATAACAAAAAGCGTAAGTCGTTAGAAGAAGTTCTAAGATACAAATGCATATAGTATACCACTTATTAACTTTATTTCCCCTATGCGGGAGAAATAACATTAATGAACCGGCAGATATTGGAAAAACAACAATTATTGTTAACCAAGGAAAATCATTCGTGGTAAAGACAAGATACACCAGGTCCAAAGAACGCGTACTCAAAAAAATATATAAATAAAAAAATATAATTGAACTTTTTTGAGTACGAGTACTTGTCAATAAAAAAAAATAAAATGTATTCCAAATTTATTCAAATCAGGTTTTCGGTAACGTATTAATAAGCTAGACCCATGCTTCGAGTTGTTTCATGCCATAAATAAACTCGAACGCTCAAAAAATCCGTTGGACAGGCAGATTCACATCTCTTACAACCAACACAATCCTCGGTTCTTGGGGCAGAAGCTATTTGCTTAGCTTTACATCCATCCCAAGGTATCATTTCTAATACGTCTGTAGGACATGCTCGGACACACTGAGTACATCCTATACAGGTATCATAAATTTTTACTGAATGTGACATAGGATCTATAGTTTTTTTAATGTCATAAATTTTCAATCTAGTAAACTTATAACTAAATGATATATTAAATTAAAATACTAGATGAAGCAATGATTTCTTTTAATAGAATTTTTTAATGAATTCTGGCTCAATTGGTAAAAAATGGGGCTAAAATACTTTGATTTCTTAAATTTTCACAAATTTAATCTAGTAAGTCATAACCTATCATATATGCAAATTTAAACCTATAATTTTTTGATTTATGCTACTTATTTAATAAGGTCGATTGGTTGATGCGAGTTGATTTTCTGTTACGATAAATTGACGAGACTATAGCTAATCCAATAGCTGCTTCAGCGGCTGCAATTGCTATAACAAAAATGCAGAAAATATCCCCTTTTAGTTGGGAATTATCAAAAAAATCAGCAAATGTTACGAGATTCATATTAACTGCATTGAGTATAAGTTCAAGGCACATAAGAGCCCTAACCATATTTCGACTCGTGATCAATCCATAAAGACCAATCAAAAATAAATAGGCACTCAAAACAAGTACATGTTCGAGTATCATTGAGCAACTCCTTATCAATTTTGATTCATTATCAATATGAATAATAAAAACAATTCACCGGATTCAATCAACTAGAATATAACAACAAAGTACGAATAAAAACTATATTAGATATTAGGGAAAAAATTTCAAATATATATATAATATAAAAATTTATATTTAAAAAATGAAATAGTATTCAATCAAATCAAATTGAATGAACGGAAAAAAATATCATAACATACACAAACACAAAGTTTTCTTTGGTCTTTACTAATTGGAACCTTTTTTATTGACGAGCCACAGAAATTGCACCTATCAAAGCAACTAAAAGAATTATTGAAATGAGTTCAAATGGAAGAAAAAAATCTGTTGATAAATGAATTCCTATTTGTTGACTATTACTTATTAAATCTTGTTCTAAAATCTGGTTTAATCTTGTAGTCCAAATAACCCCGTACCATGACGTATCGAGAATAGTAGAAATTAATGAAAAAAGAATAGTTGTACAAACCACTGAAGTAATCCCATTCCCAACAGTCCACAGATTGAAATCTATGGAATATTCGGAATCATTCATGAACATCACAGCAAATATGATTAAAACATTTATGGCTCCCACGTAAATAAGGAGTTGTGCAGCAGCTACAAAATGGGAATTTGCTAGAATATACAATAAAGATATACAAACAAGAACAAATCCTAAGGAAAAGGCTGAAAATATTGGGTTAGGAAGTAATACCACTCCCAGACCTCCTACTAGAAGACCAGATCCCAGAAAAACTAAAAGAAAATCATGTATTGGTCCAGGCAAATCCATTATATTATTAAAAAAAGAAAAAATAGAAATCCTTTTCATGACCTTATTAATTTAACCGGGGAATTTTTTTTTAATATGTTTTTAATAGAGTGAAATTAGAATCTAATGAATATTAATTGATGTAGATACAATTATTAGACAGTTTCGCTTTTTTATTCTAATATTTTCAACCTATCTATTTCAAGCAAGATAATAATTACGAAAATATTATATTAAAAGGATGAGCCTTAATACTTAATATTATTCTATAAATACAAGTTTTTAATTAAATTCTTTATATAATATAATTCAACAGTTTTGCATTCTTTTTTTAATAAAATTAATGGGTTTACCCATTTTTTGTTTGAGGTGAATTCAAAATTGTTCGAATAGTATAATCGTCAATTACTGACATTGGTAAACGCCCCAAAGCGATTTGATTATAATTCAACTCGTGACGATCATAAGTTGAAAATTCATATTCTTCAGTCATTGACAAACAATTTGTTGGACAATACTCAACACAATTACCACAAAATATACAAATTCCAAAATCAATACTGTAATTAAGCAATCGTTTTTTTCGAATATTGGTTTCCAATTTCCAATCAACAACCGGCAGATCTATAGGACATACTCGAACACATACTTCACAAGCAATGCATTTATCAAATTCGAAATGGATTCGACCGCGGAAACGTTCTGATGTTATTAATTTTTCATAGGGATATTGAATAGTTACAGGTAAACGATTTGTGTGGGATAAGGTAATCATGAAACCCTGACCAATATACCTTGCAGCTCGTAGGGTTTGTTGACCATAATTCATGAACCCGGTTATCATAGGAAGCATATTGTAATTATCTATGAATAATTTGATCTTTGTTTCTTTCTCTTGTTTAAAACAAGTAATGAATATCTTGGATTGATTTTCAATTTAGAGTGAAAAGAGTTGGAAAGAAGTTGTTAATAATAGATTACCAAGGGAAATCGGTAAAAGAAATTTCCATCCAAGATTTAATAGTTGATCCATTCTTAGCCTAGGTAAAGTCCATCTGGTTGCGATAGAAATGAACAAGAACAAATAAGTTTTAGCTAATGTAATAAAGATACCAATTGTTGTTCCAAAAATTTGATCCTTTTCAAATAGCTCCAGCATAGATATATACGGAATAGAAATATTCCAACCGCCTAAGTATAGAACTGTTACAAATAATGAGGAAATTAATAGATTTAGATAAGAAGCAACATAAAATAAACCAAATTTGATACCGGAATATTCAGTTTGATAACCTGCTATTAATTCTTCTTCCGCTTCTGGTAAATCAAAAGGTAACCTCTCGCATTCTGCTAGGGAAGAAATTAGAAAAATGATAAAACCTATAGGTTGACGCCACAAATTCCATCCCCAAAAACCATATTTTGATTGTGCCTCAACTATATCAACTGTACTTAAACTGTTAGATAATCCTAGTCGGTGATAACATTACTATTCTCACCGCTATTACAAAACCGTACATGAGGTTTTCGCCTCATACGGCTCCTCGGGGGCCGTAAATAAATATAAGGACCAGATTAGTATTATTTAGATGGATATGATGTGTTCTAAAATGGATTAAATAGAAATATATCTGGGGTCCCGAATTATACCAATGGAATTCTGTCTGCTCAAATTCTAAAACTAAAAAACGCGCTTCGGAATTCATCTCATCCTTTACAAATTTTAATTTCTATTTGTTGAGTAATAACTTAATCCTTTAATAAAGCACCCCTTGTAAAAATAAACCTAGGTTTTTAAGCCCGTCGTGTTTTTCAATTACGAAAAAGAATTAAACATCCTATTAGTTTCTTATTCATGATAGAAATTCTATTTTATTTTCGAAATCTATCAAAAAAAATATACTTGTTTCGTTCCTATTCTTCTTTCTTTTTTAGAAAAAAAGTAGGTGGACTTAAAAAAAAAATAAAGGATTATTTCGTTTCTGATAGTCATTACATTTATCGGTGGATGGGAGCATACTCTGAATCGGAATCTTGGGGAGTACTGCCTGATAATTTCTACAAATTTCAAGCCCCAATTAACCTTCTTTTTTTGTTATCTTATGTTATGCATAAATATCCTTTTCAATTTGGTTAATCTCTATTACAAATTCTTTGTGTATTTTGGTGTTTCTAACCATCCACGCGTTTTTACCTAATTGCCGATCACTTTGTAATATATGTATATGTATAGTAATTTATATAACTGATAGTGAAAACGTCATACGGTTAATATTTTTTTTAACCCGCTTCAAGCCCGGCTGACTAATCAACCAACCTTGGGGTAAAGCGATTCTTACGCTTACGTTTATTTCCATTTAACCTTTGTACATAGGAAATGAGACTTAATTTTTCTTTTTACTGCTAATTTCTGAGCAGTTTTTTTTCACTCATATATAACTATCAAATTCCTTTTATTAAGATAAACCCGAAAGATAAATATATATATTCCGTTTTTTTTTTTCATTTTTTTTATCTAGAAGAAACGGAATAAACCTTTCTGTTTCAACGAATCGCACGTAGAGATATTGATAAAACACATAGAGTTAATGGTATTTCATAACTAATCGCTTGAGCAGCAGCTCGCAGACCACCTAAAAAAGAATATTTATTATTTGATCCATATCCTGACATAAGAAGTCCGATCGGAGCAACACTTGAGATGGCAATCCATAAAAAAATACCGATATTGAGATCCGCTAAAACAAGGTGATTGCTAAAAGGAATTACTGAATAACTTAGTAAAATAGAGATAACTGCTATAGATGGTCCAATACTAAATAAAGGAGTATTTCCTCTAGATGGACGAAGATCTTCTTTGAAAAGTAGTTTTGTCCCGTCGGCTAAAGCTTGAAGAATTCCTAACGGGCCGGCGTATTCAGGTCCAATACGTTGTTGTATCCCTGCAGATATTTCTCTTTCTAACCACACAATTACTAGTACACCAGTTATGATTCCCAATACAAGAGAAAATATAGGGACAAATATCCATATGAGTCCATAGACCTCTTTTAAAGATTCCAATCTAAGAAAAGAATTTATAGTTTGTACTTCTGTTGCATAAATTATCATTTTAACGATCAACTTCTCCCATAATTATATCTATGCTACCGAGTATCGTCATAATATCAGCCAATTTCATTCTTTTAACTAGTTCAGGAAGAATTTGCAAATTAATAAAACCCGGCGGTCGGATTTTCCATCTCCAAGGAAAACCACTTTGATCTCCTATGAGAAAAATTCCCAATTCCCCTTTTGGAGCTTCAACTCTTACGTAAAGTTCTTGTTTCGATAATTCAAAAGTAGGGGAAGGTTTTTTACTAATGAATCGATATTCAAAATCATTCCACTCTGGATTCCTTTTTTTATCAAAGCCTCTGCTTTCTAAATTTTCATAGGGACCCCCCGGAAGTCCTTCCAGAGCCTGTTGAATAATTTTGATGGATTCTGTCATTTCGCTAAGTCGTACTAAATAACGAGCTAATGAATCTCCTTGTTTTTGCCACTGAATTTCCCATTCAAATTCATCGTAAGACTCATAACGATCAACTTTACGAAGATCCCATGGTATTCCGGATGCGCGTAACATTGGTCCGGATAAACCCCAATTTATTGCTTCTTCCCCACCAATAATCCCAACGCCTTCAACTCGTTCTAAAAAAATAGGATTTCGTGTAATAAGTTTTTGATATTCAACAACCTCTGTTAAAAAATAATCACAAAAATCCAAGCATTTATCTATCCAACCATAAGGTAAATCCGCCGCTATTCCTCCAATACGAAAAAAATTATGCATCATTCTCATACCGGTGGCAGCTTCGAATAGATCATATACAAATTCTCGTTCTCTGAAAATATAGAAAAAGGGAGTCTGTGCCCCAATATCTGCCATAAAAGGGCCAAGCCATAACAGATGAGAAGCTATACGACTCAATTCTAGCATAATTACTCTGATATAGCTGGCTCTTTTAGGAACTTGAATATTTCCTAATTGTTCGGGTCCGTTTACTGTTATTGCTTCTGTAAACATAGTAGCTAAATAATCCCACCGCGTTACATAAGGTAAATATTGTATAATTGCTCGGTTTTCTGCAATTTTTTCCATTCCTCTGTGTAAATAACCCAATATGGGTTCACAATCAACAACATCCTCACCGTCTAGAGTAACAATTAAGCGAAGAACACCGTGCATGGATGGGTGGTGAGGTCCCATATTGACTATCATAAGATCTTTTCCTGTAACTGGTCTCTTCATAAGTTTTTCCTTGATTCGTTCTGGTATGAATTAGATTGCTGAAAAAGAAGTTTATTCAAAAATTCAAGATCTAAAAAATTAACTAATTCACAATTTTGGAATTTAACGAGTTTTTAATTCCCGAATATTCAACTGATTAATTAATTCTTTATAACGTACTCTATTTTTTTTTGACAAATAAGCCAGCAGTCGTTGACGTTTTCCCAGAATTTTTCGTAGACCTCTCTGAGATAAATAATCTTTTCTGTGCAATTCCAAATGTGAAGTAAGTCTTCGTATCTTATTAGTGAAACTGACTACTTGAAATTCAACAGATCCCTTGCTTTCTTCTTTTTTTTCTTGAAATGAAATGAATGTATTTTTTATCATAAAAAGAAATCCTTCCCTTTTTAATATGAATTGAAAGATATGAATTTTACTGATCAGTAATAATAATGGTAGTTTTTTTGTACAAGGATCCGAATTTAATTATCAACTTCTTAATTCTTAATTTTATAAAAAAAAAAGTTTAAATTTCGATCTAAAAAAGGAGGATTTTAAAAATTTATTTATGAGTTCGCTCTGAGTGGTATCTATGTCATTAATTCAATGAATCTCATGTATAAAGATTGAATTAAAAAAAATCCCTCACATTTGTGCATCCAATTGTTTTCATATACCGTAACTTAATACTATCTATATATATAGTCAAAATATAGTAAAAAGGATCTACCATTAATGCATTTGAAATCGCGTATACATGTGTATTCTTATCATACTGAAATTATTTCCATTAGTCGTATTAAACCAATAGCGATTCATACAAGCTAAATCTTCTAATCGAAAATTGGGCCAAAGAAAGGATTTTAATTTAATTAGGTTTTTTTTATCCTTATCAAGATCTTTCTTTTTATTCAAAACTGTGGTCAAGTTTTGAATATTTGTATCAAATTTTGAATTTCTATCCCTTGCATTTTTTTTTTTTAAATTGAAACAAATTAGAACTCGAAATTCTTTACGTCGTTTAGGGGATAGAATAGTTTCAGGGACAAAGAAATTTAAACTTTTTTTTTTATAAATATAGCTTTTTTTTTTTGATCTTTTACTTATTTTTTGTTTATTTTTATGAACCAATGAAATCCCGATGGTTCTATATATAATAAGTTGGCCGTCGTTTTTTACAGACAAACGAACAGGTTCAACAATCAATATTCCTTTTTTCATTAATTTTGAAAAAGTTAAATTCTTCTCAATCATTAGAATATCTAAGCTCATCTCTCCTCTTTCAATACAAGATATCGTTATCTCGGTTGGATTTTTTAGTCTAACCAAGAAACAGTATGCTTTTACATTATTGAGGATTTTTTGATTAAAAAAACAATTCCATCGCAATTGAAAACGCGAATACCTTTTGAGAAATAAGTCAAGTTCCGCTTCGGTATTGCTTTTTGGTTGTTTTTTATTTTTACGTTTTTTTATCTTCGATTCTGTATAATTTTCTTCAATATTTTTTTCTTGCTTTGATAGAGCTGATTCCGTATTTATTTTTGTTTCTTTATCTGATTCAAACTCTTCTTGACCTGCTGATTCGTTTTCTTCTTTATTTAGATTAAAAAACCGAAGGAATTTTTTTTCGTTTGATGGTATAAAACCTTTTTTCTTTAGGGTGATCTGTTTATTCACATTTTTTGTTTCATTAAAATTGAAAAGAAGTAATTTAATTGGTATGACCCACGGTTTCATTTTATATGTACTAGAAAATAATAAAAATTCTGGAAAGAAAAAAAAGTCAAAATTTGTTATACGAGGATTGAGTATTTCTTCATTCATTCCCATCCAATCAAAAAAATTTATTTTTTGATTGGCAAGACTCGTCTTAGTTATTTTATAAATTCTTTTATAATTCTTAACTTTAGTCTTAATATATTCTTTTTTACTCCTAGTATCAAGCTCAATATTTACTTTTTTTCTAAACCAAAAGTTGAGAATTCTCCAATCCAAATATTTTCTATGCAAAATTTCCCCTATACTCCGAATATTATATTTTTCTAGAAAACAAGAGATTAAAAAATTTTCTAGGCCTGTAGACATATCAAAAAATTTTTCTGTAGAATGAATAGATTTGTAGCAAAAAAGATTATATATAGAATCCTTTTTGAAATTCTGTTTATGTTTTGGATTGAAAAATAAGTTAGCCTCACAAAAATTTTGTTTTTTGTAATTATCAAAAATTTTTTTTTCGTATGAATCCACTTTGTTTAAACTTGGATTTAGAACTAGAGAGTCTTGGTTTATTTTTTTTTTCCATTTTTGGGTTACTAATCTAGCCCATGCAATCTGAGGTAAATTATATTGAGAATGACTTCGTAACCAGTTTTTCCATTGATTTATTTCGGAATTTAAAAGGGTTTTATCTTTCCATTCATAATGAAAGATTCCTTGTTCTTGAAAAAAATCTTTTATTTGATTCTTAACAAAAAAGGATGTTATGTATATGTTATATTCAAAAAAAGACTTTAATTTAGAAAAGTTACTAACTTGAATTTGTGATAATTTGTAAAATACATATGCTTGTGATAAAGAGCATAAGTCATAACTAAAAAAATTTTTTTTTGATATGAAATTTTTTATAGTCGAAATAAAATAAATGGTATTTTTTTTTATTTTTTTTTCTCCATTTTCCTCATTCTTGTAAATATATACATCAAGAATTTTTTTTGTTGAATCAACAAAAAGTTGTGTAGTAATTCTTGGAATATTAATAATACCTAGAAAAATAGAAATAGACAGTTGTTCAACGCAAAATTTAAAAAAAAAAAAAGATTTACGAATTAATCGAGTTTTTTTTTTTTTTAATGTCTGCCAACTTTTTTTTGATGACTCAATTATTTTAGAATCATAACACAGTTTGTTACAACTATTAGTTAGTTTTTCTTTTTCTTTTGAAATTTTTTTCGTTTGATTTCTGATTGTCTTTATTTTATCAATCACATTTTTTATTTTGTTTTCGCTGAGTGAAGAATTTGGCCACTCCGTCGATTTTTTTTGAACAGATAGTTCATGAATCATCTGATTACTCATTATTGAATCTTTTTTAGTTTCATTTAATTCATATATTTCTCTCGGGCCAACTAATGGAATTCTATTTCGTTTTGAAAGGTTTTTTTTTTTTTCTTTTAGAAAAAGCAAGTTTTTTATAATCCAGTTTTTAATTTCTTTTTGGACTTTTAGGAAAATTGTTGCTCTTTCTTTGAAAATCCTTAAAACCGGAAAAGACTTCGTTTTGGATTTTTTGATTCTTTTTTTTAATTCTTTAAAAATAGGTTTAAAAAAAGAAGGCTTTGGTTTGGTAGAACCAAAAGGTAGGTCAGTTTCCAGCCCCCAAACTGTTAAAAAACGAAAATCATTTTTTTCTCCTTTTGTTTTTTTTAGTCGAGCCTTCTGAGATGATTGAAATTTATATTTATGCCAAGGTTTAAGATAAAACGGAAATAGGATTTTTATCTGAATACCATCCGTTAACCAGTTTCGTGGAAATTCTGTTTCGGATAGTTGAACCCCATTATAAGTACATTTAACATGCATTTCACGTTTCCACTCCTTTAAATCCTCTTCCCACTCGGGAAGTTGAAATAATACGATACGGATGCTATTTTTAATTATTATCAATAAAGGTAATATAATATATTTTCTAAGAATAGATTGAGTTACTAAAATAAAACCTCTTATTATTTGAGCAAATAAGAAGCTATCCCAAGTTTCCGCAATTTCTATACGTCTTTGTTCTTCTTTTTTTGATTGTTCTTCTTCGTTTTTATCAAAAAAAAATTTTTTTTTCCACATGAAATTTCTAAGAATTTTTTTTTTTAGCCCCCATATATCAAACGAAAAAAAAAAAATTTTATCTATTCTATCAAAAAAAAGGGGCGAATGTGCTTTTGCTTGCAACAATTCCCAAATAACAGTTTTACGCCTTTGGGAACGCATGGATCCTTTAATTATCTCTCGACGAAAATCAGATTGTTGTGAATAACGGATCAAAGCCATTTCATCTGTTTGATCAGAATTTTGATTATCTTTGAGATTAGTATAAATCTCGTTATGCGGTTCTTTTAAATCAGTAAAAACCACTACACGTTTTGCTTTTCTTGAACGAATTCCAGGTTCGGTTGGTATATTTTCTTCAGTTTCAGCTTCCAATTCTTCCAATTCACTTGTTAATTTGTATGACCATTGAGGAACTTGTTTATTGATTTCATGGAAATCAATAAAATTTTTTATAAGAGTTTGATCATTATTATAAATTATAACGACATCAAATAAAATTTTGAAAATTTTTATTTCTTCTTCTGACTGAATTTTTTCTTCTTGGGGTTCGGAAAAAAAATAAAGTTTTTTCTCTATTGATAAAGACTTTCTTTTAAATTTTTCTATTGTTTGCTCAAATTTTTGAGAATTAATCTTCAGAAGTATAGCATGAATTTTGTTTATCCAAGATCCTCTTATATTCTTTTTTTTATAGGTTTTGGTTATGATTTGGAACGGAGATAATTTTTTGATTCTTCCGCGCGAAATCCCATGTAAAAATGGATCATAAATTTTAGGTAAATATTCTTTTTGAGTTTCGTTATGACAAAATCGAGTTGTTTTTTCCAGTATATTTTCAATAGACCCTTTTTTATCTAAAGCTTCAATTCTATTTAAAAATGCGTTTTTTAAATTTTCCTTTTTTTCTTCATTGACCAAACTCCAACAAGTATAAACTTGATCCGAGGTTTTTTTTTCTGTTGTAAATGAAGGGATCTTTTTTTGTATCATTTCAAAAAAAGTTGAAAGGTTGGGGGGATATGTAAAAGATATTCGTTCTTTTCCATCACTTTGGCATGTATAAAAAAAATATTGTGACATTTCATTTCTTACAGTATTTTCAATTTTATCATTTTTTATATATCGATTTGGTCTATTCCATCTTTTATAATCGAAAACTAGAGTTACAAAAGGTTTTTCAAACCATAAAAAACGATCCTCTTTTTTTTTTATTTTGAAAAATTCTAAATTAGAATTTTCTTTATTTCCATCTCGATTTTCAGAAACTGTTTTATAGTTATAGTATGTTCGAACGTGGAATTCATCATCCTTATTAATTTTGTCTTTTCCATTCACTCCGATTTCTTCCATTTCATCGAGTTTGTCCAGATCCTCCCTTTCTTCCGAAAAAAGAGAAGGAAAAGGAGCTTCTTCGGTGGATACCTCTTGGTCCTGTTTAGTCCCACCCGTTTCTGAGGTTCCTTTTAGTTTCTTAGTAAAAATGGGTGATGGTATTCTGCCTAAATAGTAGACACAGGTAATAAATAAGATAATACTAAAGATTCGAGTCATAGAATTTCTCAATTCTGACACAAGGAACTTATACTTATTAGATCTAATAAGTACATTAGACCTAATAGAATTATTTTGCTGTATCCAGACTAATACCAATCCAACCCATTTCATGAATAAAATGTGACCAATTAACCAACCAACAAAACTACTTGTTACAAATAACATCTTGTTGTTGCATCGAAACATATAAATGTTGACTAATCTGGCTAACATTGAACTTGGTAAAATGAAATGGTTGAATAATTGAAAAATGAAATTATTCAGGAATACACATTGAATGCGAAGATTACGCATTTCATTTCTGGTAGTAGATCCATAATCAAAAAAGTGTTTGTGATTGTTCCAGAAGAAATGAAACAAAAGATACGGTAGAGCTAGGACAGTTATTGTATGAGGTCTACCCAATGCTAAATGCAGAGGCGCATAATAGATCGATATGAACATCATGAGCTGTCCCGCAATAAAACCAGTTGTTGCTGATACTTTCTTCTCGGTTCCTTCTTCTCCTTCGTCCATAACCCGAGCTCGGAGAAGGAAGAGATAAGAGGGCCCTATGGAGAATGTGGTCAGAAATCCATAATAGAGTCCGACCACAACGACCGAATTGATTATCTTCATGCATAAGGATACTAGATTACCTAGTATAAAAGATTGAAAAACCATCACAAACCTCCCTTTTTTCTTTTCTATTTCAATTTCTGGATTATTATATGATGATTTTGCAACTTTCCATATATAGAAATAGAAAGAGATAGACTAGAAACGACATCTCTTATGTCAATGACACCAAAGGGATATTAAATGAATGGAATTGGGATATGGATGGAATATAATGAAATAGAGCCGCTTTGAGGTTCCCTATGAAATGAGGCATGGAACGGAGCCACTACGAAGAAGTTCCGGGGGTTACGAAGGAAACTTCGAGTTCATATTGGTCATGGGTTGAGAACGGGAATTGAACTCTAGGAGATCTAATCTCCCGTTGTTCCTCAGTAGCTCAGTGGTAGAGCGGTCGGCTGTTAACTGATTGGTCGTAGGTTCGAATCCTACTTGGGGAGATTTGATTCATTCCGAATTCAAGAATTCAGAATGTAAGAGTAGGTAACCCGTTCCCTGTGTCTTTGTTTCTATTGCATTCTATCTCATCGTATCACATTCTGTTCTGTGATATTTGAGAATCACCGTCAATACCTCGGTGTAGGTCCGGGATAATCCTTTATTCCATAGTCCTGGGGCTATTTACAACTAGCCAATTCAGAATTTGCAGGTGTACTAACAAGTGCATCTTTGATGCAGTCATCGATTCTCCCGAGAGTTCACAATTACCGCGCGCAAACATATTAATGACTTAATGATGAGGAACGCATTTTTTCTATGCTACTAATACTTGTACTTGCTCTGCTATTCTGCCCAAGCCTGGCTGAGGAAGAGTTACGGGGCTTCGAAAAATATGCTGATTCGGCCGATAATCATACTATATGTAAAAAAAAAAGCGATAGATATAATAGATATATATATCTAAATTCAAATAAAAAAGAAGGCCACTCCACTCTATTTCGACAAAAGACCCATCCCCAACCAAGTTCCATAGCTTTGAGTCCGCTATCCCGAGCATGATTTTCCTACCCCCCGGAGGGAAAGGTCCTTCCCTTTTGGGCCGGTTGTGGGCGAGGAGGGATTCGAACCCCCGACACCGTGGTTCGTAGCCACGTGCTCTAATCCTCTGAGCTACAAGCCCCACCCCGTCTCCACTGGATCTGTTCCCAGGAGTACCCTACAAAAAAAGGAACCTTTCCTCTCCCCAGCCATTTCGGGTTAAGAAGATGTGAAAGTGCCTTTCTCTCTATAAGAACGGTGCGTTCCGGGGTGTGAAGTGGGATAGAAGGGATTTCATAATTGGGGTTTTGAATAAGACAACCTTTTCATTTTGCATTTTTTTTCAATATGAAAAAGTAATAAGAATGAGAGGTGTTAAGCTTTTCATCATCCTGGCGTCGAGCTATTTTTCCGCAGGACCTCCCCTACAGTATCGTCACCGCAGTAGAGTTTAACCACCAAGTTCGGGATGGATTGGTGTTGTTCCTCTACGCCTAGGACACCAGAATATCGAACCATGAACGAAGAAAGGCATGCGAGAAAAGCATATTGGCTAGTGATTGTGAGGCTCCAATTCTTGACTGGAGTGGACACCAAAGGCCTCCGCCCCTCCATCCTTTGGATAGAAGGGCAGAATTTTTGGTTTTTTCATGTTGTCAAAGAGTTGAACAATGGTTTTTTCGTGTTGTCAAAGAGTTGAACTATGAAAATAGATGGCGAGTGCCTGATCGAATTGATCAGGTCATGTAGGAACAAGGTTCAAGTCTACCGGTCTGTTAGGATGCCTCAGCTGCATACATCACTGCACTTCCACTTGACACCTATCGTAATGATAAACGGCTCGTCTCGCCGTGACCTTCTCTTGAATTCTCAAAACTTCTGTCACTCCATCCCCGCAGGGGCGGAGAACCCGTTGCTGTCTCGGCTGTGCTACCGGAGGCTCTGGGGAAGTCGGAATAGGAGAGCACTCATCTTGGGGTGGGCTTACTACTTAGATGCTTTCAGCAGTTATCCGCTCCGCACTTGGCTACCCAGCGTTTACCGTGGGCACGATAACTGGTACACCAGAGGTGCGTCCTTCCCGGTCCTCTCGTACTAGGGAAAGGTCCTCTCAATGCTCTAACGCCCACACCGGATATGGACCGAACTGTCTCACGACGTTCTGAACCCAGCTCACGTACCGCTTTAATGGGCGAACAGCCCAACCCTTGGAACATACTACAGCCCCAGGTGGCGAAGAGCCGACATCGAGGTGCCAAACCTTCCCGTCGATGTGAGCTCTTGGGGAAGATCAGCCTGTTATCCCTAGAGTAACTTTTATCCGTTGAGCGACGGCCCTTCCACTCGGCACCGTCGGATCACTAAGGCCGACTTTCGTCCCTGCTCGACGGGTGGGTCTTGCAGTCAAGCTCCCTTCTGCCTTTGCACTCGAGGGCCAATCTCCGTCCGGCCCGAGGAAACCTTTGCACGCCTCCGTTACCTTTTGGGAGGCCTACGCCCCATAGAAACTGTCTACCTGAGACTGTCCCTTGGCCCGTAGGTCCTGACACAAGGTTAGAATTCTAGCTCTTCCAGAGTGGTATCTCACTGATGGCTCGGGCCCCCCCGGAAGGAGGCCTTCTTCGCCTTCCACCTAAGCTGCGCAGGAAAAGCCCAAAGCCAATCCCAGGGAACAGTGAAGCTTCATAGGGTCTTTCTGTCCAGGTGCAGGTAGTCCGCATCTTCACAGACATGTCTATTTCACCGAGCCTCTCTCCGAGACAGTGCCCAGATCGTTACGCCTTTCGTGCGGGTCGGAACTTACCCGACAAGGAATTTCGCTACCTTAGGACCGTTATAGTTACGGCCGCCGTTCACCGGGGCTTCGGTCGCCGGCTCCCCTGTCATCAGGTCACCAACTTCCTTGACCTTCCGGCACTGGGCAGGCGTCAGCCCCCATACATGGTCTTACGACTTTGCGGAGACCTGTGTTTTTGGTAAACAGTCGCCCGGGCCTGGTCACTGCGACCCCCTTTGTGAGGAGGCACCCCTTCTCCCGAAGTTACGGGGCTATTTTGCCGAGTTCCTTAGAGAGAGTTGTCTCGCGCCCCTAGGTATTCTCTACCTACCCACCTGTGTCGGTTTCGGGTACAGGTACCCTTTTGTTGAAGGTCGTTCGAGCTTTTCCTGGGAGTATAGCATGGGTTACTTCAGCGCCGTAGCGCCTGGTACTCGAACATTGGCTCGGGGCATTTTCTCTACCCCTTCTTACCCTGAAAAAACAGGGACACCTTGCGTTCTTGAACCGATAACCATCTTTCGGCTAACCTAGCCTCCTCCGTCCCTCGGGACCAACAAGGGGTAGTACAGGAATATTCACCTGTTGTCCATCGACTACGCCTTTCGGCCTGATCTTAGGCCCTGACTCACCCTCCGTGGACGAACCTTGCGGAGGAACCCTTGGGTTTTCGGGGCATTGGATTCTCACCAATGTTTGCGTTACTCAAGCCGACATTCTCGCTTCCGCTTCGTCCACCGCCGCTCGCGCGGTTGCTTCCCCCTAAGGCGGAACGCTCCCCTACCGATGCATTTTGACATCCCACAGCTTCGGCAGATCGCTTAGCCCCGTTCATCTTCGGCGCAAGAGCGCTCGATCAGTGAGCTATTACGCACTCTTTCAAGGGTGGCTGCTTCTAGGCAAACCTCCTGGCTGTCTCTGCACCCCTACCTCCTTTATCACTGAGCGGTCATTTAGGGGCCTTAGCTGGTGATCCGGGCTGTTTCCCTCTCGACGATGAAGCTTATCCCCCACCGTCTCACTGGCCGACCTTGACCCCAGTTATTTTGAGGTCATATCTAGTATTCAGAGTTTGCCTCGATTTGGTACCGCTCTCGCGGCCCGCACCGAAACAGTGCTTTACCCCTAGATGTCCAGTCAACTGCTGCGCCTCAACGCATTTCGGGGAGAACCAGCTAGCTCTGGGTTCGAGTGGCATTTCACCCCTAACCACAACTCATCCGCTGATTTTTCAACATCAGTCGGTTCGGACCTCCACTTAGTTTCACCCAAGCTTCATCCTGGTCATAGATAGATCACCCAGGTTCGGGTCCATAAGCAGTGACAATTGCCCTATGAAGACTCGCTTTCGCTACGGCTCCGGTGGGTTCCCTTAACCAAGCCACTGCCTATGAGTCGCCGGCTCATTCTTCAACAGGCACGCGGTCAGAGCCCAGGGCTCCTCCCACTGCTTGGGAGCTTACGGTTTCATGTTCTATTTCACTCCCCGATGGGGGTTCTTTTCACCCTTCCCTCACGGTACTACTTCGCTATCGGTCACCCAGGAGTATTTAGCCTTGCAAGGTGGTCCTTGCTGATTCACACGGGATTCCACGTGCCCCATGCTACTCGGGTCAGAGCATAAGCTAGTGATGCTTTCGGCTACTGGACTCTCGCCATCTAGGGTGCGGCACTCCACCGCTTCGCCTAGCAGCACGACGCTTTTATTGCTCTCCCACAACCCCGTTTTCACGGTTTAGGCTGCTCCCATTTCGCTCGCCGCTACTACGGGAATCGCTTTTGCTTTCTTTTCCTCTGGCTACTAAGATGTTTCAGTTCGCCAGGTTGTCTCTTGCCTGCCCATGGATTCAGCAGCAGTTCAAAAGGTTAACCTATTCGGGAATCTCCGGATCTACGCTTATTTTCAACTCCCCGAAGCATTTCGTCGCTTACTACGCCCTTCCTCGTCTCTGGGTGCCTAGGTATCCACCGTAAGCCTTTCCTCGTTTGAACCTCGCTCTTAACTTTAAGGCTATGCCATCCTAAGGTGCTGCTAAATGGAAAGATCTTATCAACGTCCATGAATGAAAAATAATAGATCGAACTGCCGAATCGGAAAAATTGGGTGCTATCATATAGCTTTGTATCGGCTAAGTTCACGAGTTGGAGATAAGCGGACTCGAACCGCTGACATCCGCCACAGGGTAAACCACCGCCTATCAGGCCCCTGACTGATTCTACCATAGAGGCCAACGATAGACAAGAACTCCCCCCCGAACACAGCTTACAACTTTCATCGTACTGTGCTCTCCAAAGAGCAACTCTTCTCAAAATCTCAAAAGGTGATGAGTTGGAATCCCATTCCAACTCAGGATTCTTGTGGTTCGAGAGGATCCAGCTACAGGAGAACCAGGAACGGAGAGCTTTCCCCCCTTTTCCGCCCCAACTCTTTCGAATGCTGGTTTTAAGAATGAGTGATTGCCCTTCTCCGACCCTTACTGCCCAACCTGCGAGCGGACAGCTAATGCATTCCACTTATTGAACAGGGTTCTATGGTCGGTCCGCGACCCCTGGATACCGAAGGCGTCCTTGGGGTGATCTCGTAGTTCCTACGGGGTGGAGACGATGGGGTCGGTCCATGGATTTTCCTTCCTTTTGCCGCATTTCGCTCAAAGGGTTGAAGGGAGATAGTGCATCAAGCTGTTCGCAAGGGCCAACTTGATCCTCTTCCCCAGGGATCAATCCCAGACGAGGGAACCCTAGGAGAGCCGCCGACTCCAACTACCGTCCATGTACGATCCATACTAGATCTGACCAACTGACCATCCTACCTCCTCTACGTTCTTGACAGCCCATCTTTGTCTCAGTAGAGTCTTTCAGTGGCATGTTTCGGTCCTTTTCCCCATTACTTAGAAAAAGTGAGCCACCGGTTCAGGTACAAGATACTATCATTACCGCCTGGACAATTAGACACCCAACCCGTAATCGCAACGACCCAATTGCAAGAGCGGAGCTCTACCAACTGAGCTATATCCCCCCGAGCCAAGTGGAGCATGCATGAAGGAGTCAGATCCTTCTTATATTCTTTTCCTTGGCGCAGCTGGGCCATCCTGGATTTGAACCAGAGACCTCGCCCGTGAAGTAAATCATCGCACCTATGGTCCAACCAATTGGGAGAGAATCAATAGATTCCTTTTCGGGAGCGATTCATCCTTCCCGAACGCAGCATACAACTCTCCGTTGTACTGCGCTCTCCAAGTGTGCTTGTTCCCCCCCTCTTCCTTACCATAGGAAGTATTTGTGAAATAACTTCGATGAGAAGAAAAAAGAGGGCGTTAAGAGACCCTACTGGCCTAACCCTAGACACTCTAAGATCCTTTTTCAAACCTGCTCCCATTTCGAGGCGGAAAGGAAAAAGAATTTCACGTTCTTCCTTTCGGGAAGGGAGGATTAGGAAAATCCTATTGATTGCAGCTTTCTCCAGACCCCTGGGAAAAGCATGAAAAAAAGGCTCGAACGGTACGATCCCTCCGTCACCCCAGAATGAAAGGGGTGATCTCGTAGTTCTTGGTCTGTGAAGATACGTTGTTAGGTGCTCCATTTTATTTTCCCATTGAGGCCGAACCTAAACCTGCGCTCGAGAGATAGCTGTCCATACACTGATAAGGGATGTATGGATTCTCGAGAAGAGAGGAGCCGTGGTGGTCCCCTCCGGACCGCCCGGATCCCACGAGTGAATAGAAAGTTGGATCTACATTGGATCTCACCTGAATCGCCCCATCTATCCTCCTGAGGAGAAGTTTGGTTTCAAACCCCGGTTCGAACAGGAGGAGTACGCCATGCTAATGTGCCTTGGATGATCCACATCTCAGGGTCAGGCGCTGATGAGCACATTGAACTATCCATGTGGCTGAGAGCCCTCACAGCCCAGGCACAACGACGCAATTATCAGGGGCGCGCTCTACCACTGAGCTAATAGCCCGTCGTGCGGGCCTCCTGCTGGGGGCCCGCTATGCCAAGCCAAAAGCGAGAGAAACCCCATCCCTCTCTTTCCTTTTTACGCCCCCCTGCCGCCACACGAGAGGGACATGGGGGCGTAAAAGGGGATCCTATCAACTTGTTCCGACCTAGGATAATAAGCTCATGGGCTTTGGGTTTGAAGCTGTGTCAAACCTAAATACCCAAGAAGCATTAGCTCTCCCTGAAAAGGAGGTGATCCAGCCGCACCTTCCAGTACGGCTACCTTGTTACGACTTCACTCCAGTCACTAGCCCTGCCTTCGGCACCCCCCTCCTTGCGGTTAAGGTAACGACTTCGGGCATGGCCAGCTCCCATAGTGTGACGGGCGGTGTGTACAAGGCCCGGGAACGAATTCACCGCCGTATGGCTGACCGGCGATTACTAGCGATTCCGGCTTCATGCAGGCGAGTTGCAGCCTGCAATCCGAACTGAGGACGGGTTTTTGGAGTTAGCTCACCCTCGCGGGATCGCGACCCTTTGTCCCGGCCATTGTAGCACGTGTGTCGCCCAGGGCATAAGGGGCATGATGACTTGACGTCATCCTCACCTTCCTCCGGCTTATCACCGGCAGTCTGTTCAGGGTTCCAAACTCAACGGTGGCAACTAAACACGAGGGTTGCGCTCGTTGCGGGACTTAACCCAACACCTTACGGCACGAGCTGACGACAGCCATGCACCACCTGTGTCCGCGTTCCCGAAGGCACCCCTCTCTTTCAAGAGGATTCGCGGCATGTCAAGCCCTGGTAAGGTTCTTCGCTTTGCATCGAATTAAACCACATGCTCCACCGCTTGTGCGGGCCCCCGTCAATTCCTTTGAGTTTCATTCTTGCGAACGTACTCCCCAGGCGGGATACTTAACGCGTTAGCTACAGCACTGCACGGGTCGATACGCACAGCGCCTAGTATCCATCGTTTACGGCTAGGACTACTGGGGTATCTAATCCCATTCGCTCCCCTAGCTTTCGTCTCTCAGTGTCAGTGTCGGCCCAGCAGAGTGCTTTCGCCGTTGGTGTTCTTTCCGATCTCTACGCATTTCACCGCTCCACCGGAAATTCCCTCTGCCCCTACCGTACTCAAGCTTGGTAGTTTCCACCGCCTGTCCAGGGTTGAGCCCTGGGATTTGACGGCGGACTTAAAAAGCCACCTACAGACGCTTTACGCCCAATCATTCCGGATAACGCTTGCATCCTCTGTATTACCGCGGCTGCTGGCACAGAGTTAGCCGATGCTTATTCCCCAGATACCGTCATTGCTTCTTCTCTGGGAAAAGAAGTTCAGGACCCGTAGGCCTTCTACCTCCACGCGGCATTGCTCCGTCAGGCTTTCGCCCATTGCGGAAAATTCCCCACTGCTGCCTCCCGTAGGAGTCTGGGCCGTGTCTCAGTCCCAGTGTGGCTGATCATCCTCTCGGACCAGCTACTGATCATCGCCTTGGTAAGCTATTGCCTCACCAACTAGCTAATCAGACGCGAGCCCCTCCTCGGGCGGATTCCTCCTTTTGCTCCTCAGCCTACGGGGTATTAGCAGCCGTTTCCAGCTGTTGTTCCCCTCCCAAGGGCAGGTTCTTACGCGTTACTCACCCGTCCGCCACTGGAAACACCACTTCCCGTCCGACTTGCATGTGTTAAGCATGCCGCCAGCGTTCATCCTGAGCCAGGATCGAACTCTCCATGAGATTCATAGTTGCATTACTTATAGCTTCCTTCTTCGTAGACAAAGCTGATTCGGAATTGTCTTTCATTCCAAGTCATAACTTGTATCCATGCGCTTCATATTCGCATGGAGTTCGCTCCCAGAAATATAGCTACCCCTACCCCCTCACGTCAATCCCACGAGCCTCTTATCCATTCTTATTCGATCACAGCGAGGGAGCAAGTCAAAATAGAAAAACTCACATTCATTGGGTTTAGGGATAATCAGGCTCGAACTGATGACTTCCACCACGTCAAGGTGACACTCTACCGCTGAGTTATATCCCTTCCCCCATCAAGAAATAGAACTGACTAATCCTAAGTCAAAGGGTCGAGAAACTCAAGGCCACTATTCTTGAACAACTTGGATTGGAGCCGGGCTTTCCTTTCGCACTATTACGGGTATGAAATGAAAATAATGGAAAAAGTTGGATTCAATTGTCAACTACTCCTATCGGAAATAGGATTGACTACGGATTCGAGCCATAGCACATGGTTTCATAAAACCGTACGATTCTCCCGATCTAAATCAAGCCGGTTTTACATGAAGAAGATTTTACTCAGCATGTTCTATTCGATACGGGTAGGAGAAACGGTATTCTTTTCTTAAACTTCAAAAAATAGAGAAATCAGAACCAAGTCAAGATGATACGGATTAATCCTTTATTCTTGCGCCAAAGATCTTCCTATTTCCAAAGGAACTGGAGTTACATCTCTTTTCCATTTCCATTCAAGAGTTCTTATGTGTTTCCACGCCCCTTTAAGACCCCGAAAAATTAACAAATTCCCTTTTCTTAGGAACACGTGCGAGATAAAAAAAAAAAGAGAGAATGGTAACCCCACGATTAACTATTTCATTTATGAATTTCATAGTAATAGAAATACATGTCCTACCGAAACAGAATTTGTAACTTGCTATCCTATAATCTTGCCTAGCAGGCAAAGATTTCACTCCGCGAAAAAGATGATTCATTCGGATCAACATGAAAGCCCAACTACATTGCATTGCCAGAATTCATGTTATCTATTGGAAAGAGGTTGACCTCCTTGCTTCTATGGTACAATCCTCTTCCCGCTGAGCCTCCTTTCTTCCGTGATTAACTGTTGGCACCAGTCCTACATTTTGTCTCTGTGGACCGAGAAGAAAGGACTCACTGCGCCAAGATCACTAACTAACACTAATCTAATAGAATAGAAAATCCTAATATAATAGAAAAGAACTGTCTTTTCTGTATACTTATGTATACTTTCCCCGGTTCCGTTGCTACTGCGGGCTTTACGCAATCGATCGGATCATCTAGATATCCCTTCAACACAACATAGGTCGTCGAAAGGATCTCGGAGACCCGCCAAAGCACGAAAGCCAGGATCTTTCAGAAAATAAATTCCTATTCGAAGAGTGCATAACCGCATGGATAAGCTCACACTAACCCGTCAATTTGGGATCCAATTCGGGATTTTCCTTGAGGGATATTGGTAAGGAATTGGAATGTAATAATATCGATTCATAATGGATTCATATCGATACAGAAGAAAAGGTTCTCTATCGATTCAACAAGTGCTGTACTTATGGGAAAGCGATAGAGAAAGAGAAAAAAAAAAACGAAGATTTCACATAGTGATTTTTTTTTGATCAAAAAAAAATATGATTGAATTTATTTCGTACCCTTCGCTCAATGAGAACATGGGTCAGATTCTATAGGATCAAACCTATGGGACTTAAGAATGATGGAAGGGAATAAAATCAAAAAAGAAATCAAATAAAGAAAAGAGAGGGAAAATAAAGAAATAATAAGTAAATAAAAATGAAGTAGAAGAACCCAGATTACAAATGAACAAATTCAAACTTGAAAAAGTCTCTTTCTGATTCTCGAAGAATGAGGGGCAAAGAGATTGATCGAGAAAGATCTCTTGTTCTTATTATAAGATCGTGTGATTGGACCCGCAGATGTTTGGTAAAAAGAATAATCTTATCCTTTGAGAATAATCAAAAATAGAAAGTGTTCAATTGGAACATGAAAACGTGACCGAGTTTATCCTAGTTACTCTTCGGGACGGAGGAGATTCGCGAACGAGGAAAGGGACCCAATGACTTCGAAAGAATTGAACGAGGAGCCGTATGAGGTGAAAATCTCATGTCCGGTTCTGTAGAGTGGCAGTAAGGGTGACTTATCTGTCAACTTTTCCACTATCACCCCCAAAAAACCAAACTCTGCCTTACGTAAAGTTGCCAGAGTACGATTAACCTCGGGATTTGAAATCACTGCTTATATACCTGGTATTGGCCATAATTTACAAGAACATTCTGTAGTCTTAGTAAGAGGGGGAAGGGTTAAGGATTTACCCGGTGTGAGATATCACATTGTTCGAGGAACCCTAGATGCTGTCGGAGTAAAGGATCGTCAACAAGGGCGTTCTAGTGCGTTGTAGATTCTTATCCAAGACTTGTATCATTTGATGATGCCATGTGAATCGCTAGAAACATGTGAAGTGTATGGCTAACCCAATAACGAAAGTTTCGTAAGGGGACTGAAGCAGGCTACCATGAGACAAAAGATCTTCTTTCAAAAGAGATTCAATTCGGAACTCTTATATGTCCAAGGTTCAATATTGAAATAATTTCAGAGGTTTTCCCTGACTTTGTCCGTGTCAACAAACAATTCGAAATGCCTCGACTTTTTTAGAACAGGTCCGGGTCAAATAGCAATGATTCGAAGCACTTATTTTTACACTATTTCGGAAACCCAAGGACTCAATCGTATGGATATGTAAAATACAGGATTTCCAATCCTAGCAGGAAAAGGAGGGAAACGGATACTCAATTTAAAAGTGAGTAAACAGAATTCCATACTCGATTTCATAGATACATATAGAATTCTGTGGAAAGCCGTATTCGATGAAAGTCGTATGTACGGTTTGGAGGGAGATCTTTCATATCTTTCGAGATCCACCCTACAATATGGGGTCAAAAAGCCAAAATAAAAGATTTGAGCCCTTATAAAAAGAAAACAGATTCTTGAACCCCTTTCACGCTCATGTCACGTCGAGGTACTGCAGAAGAAAAAACTGCAAAATCCGATCCAATTTATCGTAATCGATTAGTTAACATGTTGGTTAACCGTATTCTGAAACACGGAAAAAAATCATTGGCTTATCAAATTATCTATCGAGCCTTGAAAAAGATTCAACAAAAGACAGAAACAAATCCACTATCTGTTTTACGTCAAGCAATACGTGGAGTAACTCCCGATATAGCAGTAAAAGCAAGACGTGTAGGCGGATCAACTCATCAAGTTCCCATTGAAATAGGATCCACGCAAGGAAAAGCACTTGCCATTCGTTGGTTATTAGGGGCATCCCGAAAACGTCCGGGTCGAAATATGGCTTTCAAATTAAGTTCCGAATTAGTGGATGCTGCCAAAGGGAGTGGCGATGCCATACGCAAAAAGGAAGAGACTCATAGAATGGCAGAGGCAAATAGAGCGTTTGCACATTTTCGTTAATCCATGAACAGGATCTATATAGACACATAGATCCGTGGATCCATACATCTCGATCCGAAAAGAATCAATAGAAAAAGAAAAAATCGGAATTGATCGATCTCTTTCTCGAAACAAACGAAAAGGAAAGAAAAGACGAAACATAAATCATGGATCAACTAAGCCCTCTCGGGGACTTGCTTAAGAATAAGAAAGAGCAATCTCATGTAAATACCATGGAATAAGGTTTTAACCTATTCATGGGGATTCCGTAAATATTCCATTCAAAAAAAAAAAAAATTGGTTTTTTTTTGGAGATTGGATGCAGTTACTAATTCATGATCTGGCATGTACAGAATGAAAATTTCATTCTCGATTCTACGAGAATTTTTATGAAAGCCTTTCATTTGCTTCTCTTCGATGGAAGTTTTATTTTCCCAGAATGTATCCTAATTTTTGGCCTAATCCTTCTTCTGATGATCGATTCAACCTCTGATCAAAAAGATATACCTTGGTTATATTTCATCTCGTCAACAAGTTTCGTAATGAGCATAACGGCCCTATTGTTCCGATGGAGAGAAGAACCTATGATTAGCTTTTCAGGAAATTTCCAAACGAACAATTTCAACGAAATCTTTCAATTTCTTATTTTACTATGTTCAACTCTCTGTATTCCTCTATCCGTAGAGTACATTGAATGTACAGAAATGGCTATAACAGAGTTTCTGTTATTCGTATTAACAGCTACTCTAGGAGGAATGTTTTTATGTGGTGCTAACGATTTAATAACTATCTTTGTAGCTCCAGAATGTTTCAGTTTATGCTCCTACCTATTATCTGGATATACCAAGAAAGATGTACGATCTAATGAAGCTACTATGAAATATTTACTCATGGGTGGGGCAAGCTCTTCTATTCTGGTTCATGGTTTCTCTTGGCTATATGGTTCATCCGGGGGAGAGATTGAGCTTCAAGAAATAGTGAATGGTCTTATCAATACACAAATGTATAACTCCCCAGGAATTTCAATTGCGCTTATATTCATCACTGTAGGAATTGGGTTCAAGCTTTCCCTAGCCCCTTCTCATCAATGGACTCCTGACGTATACGAAGGAGTGCGGTTCGTTTGAGAAATTCCTACCTCTCTATCTATCTCTGAGATGTTTGGATTTTTCAAAACTCCATGGACATGCAGAAGAGAAATGCTATCCCCACGCAGACCAAGACAGAACTTTGACTTGTTCAAATAACAATTAATGTGAAGCAGGGTCAGGAACAACGAATCTCTTTATGATAAACGGATCCATTTTGCAAGTTTGTTATTACGGGTAGTTCCTACAAAGGATCGGACTAATGACGTATACAAGAAAGACTTGAATTCTCGATGTAGATGCTACATAGTTGGTTCTCATCCTTCAGAGACTACGAGTGTAATAGGAGCATCCGTCGACAAAAGGATCACCCTAAGATGATCATCTCATGGCTATTGAGAACGAATCAAATCAGATGGTTCCATTTCTCAATCTTTCGGACGTGCTCCTACGGAACCAAGGTCGAAACGATTGAGAAAAATCAGTCATTCACAACCACTGATGAAGGATTCCTCGAAAAGTTAAGGATTAGTCATCCGTTTTAGAAAGGATTCGATCTTATACATACGCGAGGAAAGTAATCAAAAAAGAAAGAAGATGAGTTCTTCTTTACTTTTATCACTTAGGAGCCGTGCGAGATGAAAGTCTCATGCACGGTTTTGAATGAGAGAAAGAAGTGAGGAATCCTCTTTTCGACTCTGACTCTCCCACTCCAGTCGTTGCTTTTCTTTCTGTTACTTCGAAAGTAGCTGCTTCAGCTTTAGCCACTCGAATTTTCGATATTCCTTTTTATTTCTCATCAAATGAATGGCATCTTCTTCTGGAAATCCTAGCTATTCTTAGCATGATATTGGGGAATCTCATTGCTATTACTCAAACAAGCATGAAACGTATGCTTGCATATTCGTCCATAGGTCAAATCGGATATGTAATTATTGGAATAATTGTTGGAGACTCAAATGGTGGATATGCGAGCATGATAACTTATATGCTGTTCTATATCTCCATGAATCTAGGAACTTTTGCTTGCATTATATTATTTGGTCTACGTACCGGAACTGATAACATTCGAGATTATGCAGGATTATACACAAAAGATCCTTTTTTGGCTCTCTCTTTAGCTCTATGTCTCTTATCCCTAGGAGGTCTTCCTCCACTAGCAGGTTTTTTTGGAAAACTCCATTTATTCTGGTGTGGATGGCGGGCAGGCCTATATTTCTTGGTTTCAATAGGACTCCTTACGAGCGTTCTTTCTATCTACTATTATCTAAAAATAATCAAGTTATTAATGACTGGACGAAACCAAGAAATAACCCCTCACGTGCGAAATTATAGAATATCCCCTTTAAGATCAACCAATTCCATCGAATTGAGTATGATTGTATGTGTGATAGCATCTACTATACCAGGAATATCAATGAACCCGATTATTGCGATTGCTCAGGATACCCTTTTTAGCTTCTAGAATCTATTTCTTAGTTCAAGATCCCTCTTACTAACTGGAATCAAAGAATTAGTAGATCGGTTCCGCCCAAAATGGGAATGGACTAAGGTTATGAACTTATAATCTATAATCTGATGATCGAGTCGATTCCATGATTATAAGTTCATTCCATACCGGACCAGACCGGAATAAGGTTATATACATTCTCATTATGAGAAGGGGTCATTCGAGCGTATCTAAATAGATACTATGTTTACATAGGGATCCCTACGTCGTTACATTCCATTTAGGATTAGGAATAGGCGAAATCTGACCTACTTTTTACATATCTCTCGTTATTTGGGACCCTATTCACCTCTTTGGTTGGACTTCTATTGAATCGAGAAATAGGTTTGATTGTCCATCTTTTTGATATAATATTAATATATATATAAGGCATCCTCCGGATAAGGATAATTAAAATCTAAGCAATTAGATGTCCGACTCGGGCCTATATGACATGACCGATCAATAGAAATACTTCAACACTCCACCTTTGTCATATATTCAATACACCGTACTAGATAGATATCATATTTATGGAATACGATTCACTTTCAAGATGCCTTGGTGGTGAAATGGTAGACACGCGAGACTCAAAATCTCGTGCTAAAAAGCGTGGAGGTTCGAGTCCTCTTCAAGGCATAATATTGAAATGGAATAAGTTCGGCAGCGGATCGCGAAATCTTGGCGATCTTCTCTATCTAATGAATGGGGAGGGGGAGTCCGCTTTGAAATCGTCCGCCCTGCGCCCCGCAGTATATGATTCAACAGGAATCACACAAGGGTAGATTGATACAATCTAAACCTCTGGTAAAATGCCCCCGTAACCCAGCAGATAAAGTACATAGTCCGTTTTAGGGATTGGTGACTTACCCATTCAGTGACTTTGGCACTGGATGGACGTTACCAAAATTGGTACTATCGGGTCGGGTGAATTCAATAATAGACGCCTGGCGGCATTCCAGCCTTCCTTCTCCTTTCAGGACCTATCCTAAACAGAATCCAGTACTTCTTGGTCGTGAATATCTGAATAGGGCGAACCACTCCGTGGATATCTTTACTTCGGAACAAAACAATTAGAATTAGGCTCGGTCAACTGGAATGTGTATTATCCATATAGGGGATCTTCCAATTGAGAAGATCTATCGACCTGAGACGAAGAGAAAGGTCTATCTATTTTATTTAGTTATTCAGTTGATTCGTTATTGGAACAGATAGCAACAACAATTTCATCCGACATGCGTATTTTTGATTTTCCAATGGATTTCCATCCTTCATTAATGGAAATTTTTTTGATGTAGTGAGTAATAGCTCTGGTTGTTCGCTGTTCAAGAATTCTTGTTTAGGCAGTTCGTACCATCCATACATAGTGTTTTGATCTAAGATTTCAATTCTTCCATGTTTCCGTCGTAGCATATTGTTCCATGGAGCTAAGTGGAAGAAACAGGTGTTTCTACAACTCTACCACCCAGTCAATTCCGTTCCACTTAATCCCTATTTCATGGACACATATCTTTCCGGCTAAGTAATGGGAAACCTTTCTCCTGTTACATTACATGAATCCTATTTTCATTTCATCCGGAAAAAGCCATCTTTTTTTCAACAATGTCTTTGTCATTCGATCCACTAGCGTTCCGTTAGATAGGAACAGATTTGATAAATACTGATAACTCTCGGATAGAGTATTAGAACGGAAAAATCCATTAGATAATGAACTATTGGTTCTAAGCCATCTCTGGCGCTGAATCAACAATTCGAAGTGCTTTTCTTGCGTATTCTTGATAAACCAGCGTTTATATATAGATGTAGGAGGATCTGTTTGGGAAGTAAGAAGCCCCTTTGACATCTCTTCATCTGCAAAGAATTCTCGATGTGAAAACACAGAGACAAAGGGCTGATCTTTGAATAGGAAAAAGAGTGGATCTGCGGGGTCCCAAATGAATTGGCTTATTCTAAAAAAGCCTTGTTCTTTGGAAGACCTATCTCGTCTCTGGTACTGCATGGTTCCGCTCTGCAAGAACTCCGAATCATTCTCTTGAAGCTCATACTTTTCATCATAAATGATCCGCTTGCCCCGAAATGACCCGGCCAAATAGGGAAATCCCAATTCATTAGGCCTTTCGATACAATCAAATAGAAAGCCCCGAGGGCGCCATATTCTAGGAGCCCAAACTATGTGATTGAATAAATCCTCCTCTATCTGTTCCGGGTCGAGGACTGCTTCTCCTTCCCCTTCTTCAAACTCCGATTCGTATTTTTCATAGAGAAATCTCTGATCAACGATAGAACAAGATCCATCTTGCATCATATATAAGGGATCCCTTGGTTCGGAGCGAAAAAGCAATGTCACTCGATCATTATCAAACTGACTGCAATCTTTTTCTGTCCGTGAGGATCCCACCAAAGCGCCTTGCACTTCTAATAGGCCATGAAATAGATCCGAATCATTCTCAATGAATCCATAAGAAGTGATCCTATTTTTTTCATCGGGTCCGGGTAGAGACCAAAGGTCTTGAGCGACCGATCCGGCAGAACAACTCAAAAGATAAAGAAGTATCGTGAATTTCTTCATGCTCGTTCCAAGTTCGAAGTACCATTTGTACAAATAAGAATCCCCTTCGTTACATGATTTCTTCTTCATATAGATAGATATAGGATCTATGGGGCAATTACTTATAAGTACATTTTGTGCAACAACCCTTCCTATCTGATAGAAAAGGATCCCATGATCCTGAACCGATCTTACCTGGGATCGCAAATCCCAAGTTTGTCTATGAAGAGCAGATCTAATTGTATTAGTGTCTATAATTGATTTCTTCTGTGTAATACTAATTGATAAGGCCTCATTGGTAAGTGCTACAAGATCTCGTGCACTGGAACCCATGGTTATGGACTCGAATCCATTAGTATGGAACATTTTCTTTTCCAAGTGAAATCCCCTAGTATAGGAAAGAGTGAAAAAGTGCTTTCGTTGTTGTGGAATAAGAAGCCTTCTTATTTTAATGCATGTATTTAATTTATTCGGGGCTATTAGAGCGGGATCCACTTTTTGGGGAATATGAGTCGAAGCAATAACAAGACTATTTCTAGTCGAACATCTTTCACAATCCCTGGAGAGAGAGTTCACCAAGAGACCGAGGGCTAAGTAATTCGACTCATTCACATCAAGATCATGAATGTTTGGAATCCATATTATGCAAGGAGACATTGCTTTTGCTAATTCGAATTGAAGGGTGATATAAAATCGGTCTATTTCCGACATCATATCCATAGTTAGCGCATTCATCATAGTTAGAAGCTCCAGCTCCGTATCAAGTTCACGATCAATATCGTTACTAGCATCAATATCGTCACTATCATCAATATCGATATCATCAAGAAAAAAACCTTTCGGCTTGTTATCCAGGAACTTGTTCAGACATACTGTAATGAAAGGAACATAGGAGTTTGTCGCTAGGTATTTGACCAAATAGGATCGTCCGGTTCCTATAGAACCTATCACTAAAATACTCCTAGAGGGGGATAGGGCTAAGCGGAGCGAAAAGGGTTTTCCATGAGACGGGAAATGAAAACTATTAGCCCCACACGAAGTTTGTGAATAAGTGATTGTCTGATAATGAGCAAGGAATATCCGTCTTTCTGCTAAACAGGATGTATTGAACTCATAATTCATTAGATACTTTTTATGAATGTCAACTAAGTATCGTAAGTAAATTGTTCCCGGTTGTTCAATCATTTGATAACCAGAGTCATTCTTTGATAAATGATCACTATGAGTCAGACTCAATAGAATTTGATCAATCCTTTTTTCTGCCCTTAAGGTGGAGAACTGAACCAAGAATTCTCTTTCTTTATCATCAATCGAATCACTGTTCGCGACCCAGGATTCTATTTTATCATCAATCCAATCACCGCTCACGTTTTTTCTTTTTCTTATCAATGAATAGATGTCTTTACTTGTATGACTTAGATGTCTCGTATTTCTCGAAAAAGTGATTCGATTGATGGGATTTGGTATGATACTTATGAGATCGATGATATCGATGAAGTTTATTTTCAAATCTGTCTTCTTAGAACGTATTGATTTGACCCCATAAGCGGGATCACCACCCCATAGCATGTTGCCGCCAGAACCCCGTATTTCTTCTAGACAATCTCCTAATTGTTCCAGAGCAACTAGAAAAAGATTCTTTAACCAGAAAGAATTCTGTTCAGATGTAGGATACCTATCCAGAAGTTTTCGCAACTCAATCATGTATGATGGAATCATCAAAGATTTGATCTTTTCGAACTCTGTCTGTAACTCACTATAGGCTCGGGAAACAAAGAGAAGATGTGTACGAACGATATATCCAGCAACAAGAAGAAGGAAAAGGATTGAATAGAGGACCTCACGAACATTTGGCGATCTCAGATGTGTCGATATCAACGATGACTCATTATTTCGATGAATCATTTCTTCGGACAGAAGAAGATTATGTAAAGACTTACTCGAAATCTCACTTATCAGATTCCTTTGTGGAAGACACAATTTTTTCTGAAGAATTCGCCATGATATATCTAATCCATACATAATATCATGAAAAATGGATACAAATTTTTGACTGCTACTTAGTATCCGCAATAGGTCTGAAAAAATATCTAAAAATATCAAATTTAGATATTTGTACCCTGTCGAAGTAAAGAACCATGGCATATATGTTTGGAATAGATTCCATTTTGAGAGAGTTGAAAAAGCACTATCTCGTTGAAAGGTTCTATCCATCTGCCCTTTGTCAACGCATTTTTTTAGGCAAAGACTCCGTTTTTTCCTCTGTAAATATTTCTCAGAACATGGAGTGTGAATCAAACCCACGTTTGAATTGAAATTGAGATACTGATGCAAGCTCTTCTCTTCTGAATCGGATAGATTCATATCTGAAAGAGTTTGACAATACGTTCTTTCCAAATTTACTCTTTGTCCCTCTATTAGAGGTGTTCCAGAAATGTCTGCAATCGAGTAAATAGCTCTACGAACTAATGGATCGGATCGAATTGGAAAATGGAAAGATTTGTACAAGTTATACCTTTCGTCACCACTTTGTGGAAAATCGTTAGATATGAATATGTTAGATACCTGTGACTCGATTGACGAAGGTGAAATAGTATCTCTCTCCAAAAAAGCATGTTTTTTTTTACCACCACACGAAGAAAATATTTTGTTGTGAATGAACAAGATAGTGAGGAATTGTCCATACGTAAAATCAGAATTATTGAGACGGGCCTTTTCCACATAAAAAGGGAATCTTTTGTTACAATAGAAGCAGAAGTGATGTGGATTATTCAAGAATCGAAGTCGATTTGCTTTAGAAAAAGAAGATATCAATGAACTTCTCTGAAATGGTTTCACGGGATTCAGCCAATTGTCTTGATCGTGGGATACGATTGAGAAATAGGAATCCGTGTTATCAAAAGATTTCCTGCGATTCTTTCTAGTATGGAATGAGTCAATCATCCACTTTGGTATCTTATTGAACAAAAATGGTGATATTGTTCCTCCATTGATCAAGAATTTCGATTTTTGAGAAGTATTATGATCATCCAATAAAAAGGGTTTCAATTTTTTAAAATGAACGATTTGAAGACCTATTGATTCTAACAACTGATTGCAGGGTTGATCGTTCGGACCTTTCAATTCATAGATGTGGATCTCAGACCTATGAATGGGGATATTCTCGAAACTCACAAAGAAAAAAGGAAGTGAATTAGACAAAAAGAGAAGTAACTTGGACAAAAAACGAAGTAACTTGGACAAAAAGAAACGAAGTGACTTAGACAAATCTTTTTTATCAATAACCTCAGACCAATCAATCGAATATTGATTAATACATAATCGATCGAACACTACTTGAAAACGGCTCTTCCGCTCAGAAACGAAATGTTTCAAATGCTCCTGGAAATTCTTGCTCCCATTGGACCATTTGTATCTATATGCATTAGGATCCCGATTTATGGATCTCTCGGTTCGAGAAAGAAAAATAAGAGGATCGAACCATTTCTTCTGACTCTTTTTCAAATTCGATAAATGTTGGTTGATCGTATCTTTCATTATAGTTCTATGATTCAGAGTATCATTTCCTATTAGATCCCTTTGAATTCCATATTCGAAGTTGCGATCAGGTCTCTTCATTAAAAAGAATCGATTCAATACATTTCTTATGTACCCATAGGGACTATATTGGAATTGGATTTGAATCAGATTTCGGATCAATCTATATTGATTGACTGCCTCCATTATGTTGTTGCTAGCAAATACCACTCTTTTTGGTTTTGGATCTTCAAAAAAATTCCCGCAGGAGATCCGGACCCAATTTTTTCTGATCCTTCGATAAAAAGATTCATTTTCTTCATAAAAAATAGGAGGTAGAACCAATAAAGATTTCTTTTTCAATTCATCCCTGGAGTTGAAAACCTCCTTCAAGAATTGTCTTTGATCCAATCCGTAGGAATCAATAGAAAAGGCAAATCCCGTATGATACACCAGATCCGGCTCGGTTATTGATAGAGTGAATAGATCTGCCATTTCTTGAAATCTCTCTTCTGACTCAAAATCGTGGCGTAACGTGTATCCCCCCCTCTTCCGTTCATGGAATAGATGAAATAAATAAAAAAATGGATTTTTGTTCAAGAATGAAATCTTATTGGAACTGTCCATATCCAGTTCATCCTTCGGAACCGTATCACATCCCAGATCTGATGAAATAGGATGAATTGAGACGGTATTTTGTAAATACGTAATTATCTTGAATATATTAACTATTTCTTTATTTTCCGATCGCCTGGAAGGGACAAAAGAAACATCTTGTTCTTTCTTCAACAATTTCTGATCCCTAGTGGACCTCTCAGTAGGATTCGAACCCAGATGAAGTTCTGACCATCTGTCAGAGAAAAAAGAACGAATGGCTCTTGTAGAATTCCAAAAAAATTCTTCGCTTTCTTCCGGAAGCAGATGATTATTCATTCGCTTTTCACGTTCCGTGAATAGCCGGGGCATTGAGGAATATCCAGAAAGGTATTTAGGGAATCGGTCTGATTCTATCTCTCTTCCTTCCGTTTGAATAAAGGAAGGATCCCAAAGAATCGATCTTTCTTTTAGTTGTTGAATCTCTCTTTGATTGATCAATGTGTGATAGTGATATTCCGAATCCTCATTACTAATGGAATCGAAAGGATCTATGAATTGATCAGAAGATCCGTTCAATTGGCTAGAATCCGTTACTTGAACGAAACTAGATCTTGTAGAATCATATTGAATATTTGACGATACATTTCGTACCTTGCTAAAAAATCTATCCTTGTTTACCAACCACACATTGTCTAACCAAATCCAATTCTCTCTCGATATTTTCCTCAAAAAATCCGATTCGTGCGGATTCTTCCCCCAACTAACGAAGAGATCTTGGTGGAATTGCCACATATGAAATTGAGCACAATTTTGCAAAGAAATAGCCCGCTTGTTTCTCGAGAAGAGATGGGAAACATGCTCAATATCATTTGATTGAATAGTTGACCCAGCTCCTTGTTGTTTGAAGAAACCCTCCACTTCAATTGGTATTTTTTCACGAAAAGCAAACATGAGATAACAAATCCAGTCTTTCACTAAGATTTCGAATAGCTGTCCCGAATTCAAGTTGATTATGTTTCGCCTCTTATTCGGAGAAAGACGATCAAACAATTCCCAATCATGGCCCTTGCGGATCGGATCATCCATATAATATACAAAAAGAAACTCCAGATATTTGATATCTTTCTCTTTAAATGAGATATCAATTCCAGCGACGGTTTCATTAGATATCTTACAACAAAAATCCCTCTTTTTTCCGATCCAGTTCCTCCACCACCGCGAACTCCAGTTAGATTCAGGCATGATACACTTTTTAGTTATTGGGAGAACCCGAGTACTCTCTTTCGGATCCCGGAAACAGCTCTCAGAGATCTTTTTTCCTTTTGTAAAATACAGGAGCGAAACAATCAACCTATTGATATTGGAAGACCCAAAAGATTCTTCCGATGTATCATTTCTGGGTCCAATGGAATTCATAGGTATAGGAAGAAGCCCTTTCAAATAGAGATTTTTGCTTTCGACCATATTTCGATTGTTAATACGATATAGAAGGGCCGCTACTACAAATAGTACTACACCCTTGATCGTGAAATATCGATTGCTTGTTGAACCCTGTGAATTGCGCAAAAGTAGGATACTAAAAATTCGAGGGTCCAAGAGTTTTCTAAAACGTTCTTGGTGGAAAAAAATATGAATGAAAGATCCCACTGAATTGATTTGGGTCCATGAATCTAAGAAATAGTGAGAATTCTTGATCTCTCTCACTATTTCTCTCAATTCGAAAATCCAGGATTTGAATTGATGTCCTTTCATTGATTCCTCCTAAATTGCATTGATTTATCCTAAAGATTTCATTTCAATTGGAATTTGGTTATTCACCATGTACGAGGATCCCCACTAAGCATCCATGGCTGAATGGTTAAAGCGCCCAACTCATAATTGGCGAATTCGTAGGTTCAATTCCTACTGGATGCACGCCAATGGGACCCTCCAATAAGTCTATTGGAATTGGCTCTGTATCAATGGAATCTTCTCATCATCTATACATAACGAATTGGTGTGGTATATTCATATCATAACATAACATATGAACAGTAAGAACTAGCATTCTTATTGAGACTAGAACTCATAGGGAAGAAAATCGATTTATGGATGGAATCAAATATGCAGTATTTACAGACAAAAGTATTCGGTTATTGGGGAAAAATCAATATACTTTTAATGTCGAATCAGGATCAACTAGGACAGAAATAAAGCATTGGGTCGAACTCTTCTTTGGTGTCAAGGTAATAGCTATGAATAGTCATCGACTCCCCGGAAAGGTTAAAAGAATGGGACCTATTCTGGGACATACAATGCATTACAGACGTATGATCATTACGCTTCAACCGGGTTATTCTATTCCACCTCTTAGAAAGAAAAGAACTTAAATCAAAATACTTAATAGCATGGCGATACATTTATACAAAACTTCTACCCCGAGCACACGCAATGGAGCCGTAGACAGTCAAGTGAAATCCAATCCACGAAATAATTTGATCTATGGGCAGCATCATTGTGGTAAAGGTCGTAATGCCAGAGGAATCATTACCGTAAGGCATAGAGGGGGAGGTCATAAGCGTCTATACCGTAAAATAGATTTTCGACGAAATACAAAAGACATATATGGTAGAATCGTAACCATAGAATACGACCCTAATCGAAATGCATACATTTGTCTCATACACTATGGGGATGGTGAGAAGAGATATATTTTACATCCCAGAGGGGCTATAATTGGAGATACCATTGTTTCTGGTACAGAAGTTCCTATAAAAATGGGAAATGCCCTACCTTTGAGTGCGGTTTGAACTATTTGATTTACGTAATTGGAAGTAACCAATTAGGTTTACGACAAAACCTAGAAATCGATCACTGATCCAATTTGAGTACCTCTGCAGGATAGACCTCAACAGAAAACTGAAGAGTAACGGCAGCAAGTGATTGAGTTCAGTAGTTCCTCATATAAAATTATTGACTCTAGAGATATAGTAATATGGAGAAGACAAAATTGTTTCAAGCACCGACAGAACCATAAGCGCCCCTTGTTTCAAAGAGAGGAGGACGGGTTATTCACATTTCATTTGATGGTCAGAGGCGAATTGAAAGCTAAGCAGTGGTAATTCTAAAGATTCCCCCGGGGAAAAATAGAGATGTCTCCTACGTTACCCATAATATGTGGAAGTATCGACGTAATTTCATAGAGTCATTCGGTCTGAATGCTACATGAAGAACATAAGCCAGATGACGGAACGGGAAGACCTAGGATGTAGAAGATCATAACATAAGTTATTCGGCAGATTTTGATTCCTATATATCCACTCGTGTGGTACTTCTACCATATATAGAAGAATTCTACGATATATATAAGATAAGATCCATCCGTATAGATATCATCATCTACATTCAGAAAGCCGTATGCTTTGGAAGAAGCTTGTACAGTTTGGGAAGGGGTTTTGATTGATCAAAAAGAAGAATCTACTTCAACCGATATGCCCTTAGGCACGGCCATACATAATATAGAAATCACACTTGGAAAGGGTGGACAATTAGCTAGAGCAGCGGGTGCTGTAGCGAAACTGATTGCAAAAGAGGGGAAATCGGCCACATTAAAATTACCTTCTGGAGAGGTCCGTTTGATATCCAAAAACTGCTCAGCAACAGTCGGACAAGTGGGAAATGTTGGGGTAAACCAGAAAAGTTTGGGTAGAGCCGGATCGAAATGTTGGCTAGGTAAACGTCCTGTAGTAAGAGGAGTAGTTATGAACCCTGTCGACCACCCCCATGGAGGTGGTGAAGGGAGGGCTCCAATTGGTAGAAAAAAACCCGTAACCCCCTGGGGTTATCCTGCGCTTGGAAGAAGAACTAGAAAAAGGAAAAAATATAGTGAGACTTTGATTCTTCGTCGCCGTAGTAAATAGGAGAGAAAATCGAATTTCTTTCTTCGTCTTAAAAAAAATAGGAGTTAATTAACTGTGACACGTTCACTAAAAAAAAATCCTTTTGTAGCAAAGCATTTATTAAGAAAA

# A83 chloroplast genome
[truncated: 10,745,322 more chars]
